# Photochemical Cobalt-Catalyzed Formal Hydroacylation via Radical 1,2-Carbonyl Migration

**DOI:** 10.1021/acs.orglett.6c04309

**Published:** 2026-10-23

**Authors:** He Chen, Ilaria Marilungo, Christopher J. Teskey

**Affiliations:** Institute of Organic Chemistry, TU Braunschweig, Hagenring 30, 38106 Braunschweig, Germany

## Abstract

Radical rearrangements constitute a powerful strategy for constructing complex molecular scaffolds by exploiting C–C bond cleavage and reconstruction. Despite significant advances, many such transformations require preinstalled, non-native functional groups or are limited in substrate diversity by the identity of the radical initiating the reaction. Herein, we report a photocatalytic 1,2-carbonyl migration of β,γ-unsaturated ketones via cobalt MHAT under mild reductive conditions, enabling direct access to structurally rearranged ketone products from readily prepared starting materials.

## Graphical abstract

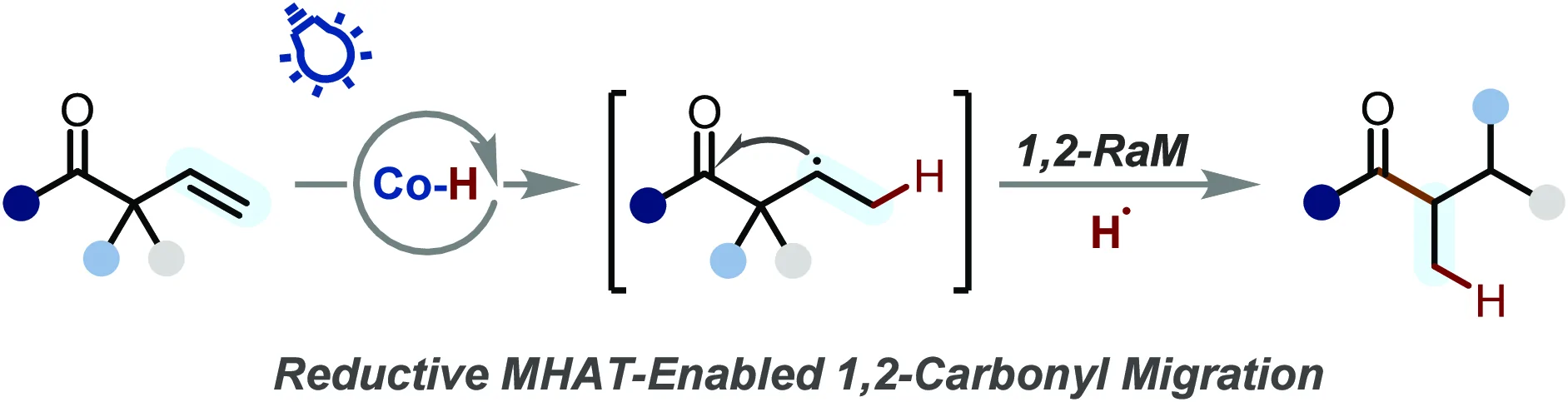

Metal hydride hydrogen atom transfer (MHAT) has emerged as a powerful platform for alkene hydrofunctionalization, enabling selective radical generation under mild conditions. In this process, Markovnikov-selective hydrogen atom transfer from a high-valent metal hydride species (most commonly cobalt, manganese, or iron) leads to the formation of the more stabilized carbon-centered radical (Scheme 1a).^1,2^ Traditional MHAT methods, however, commonly rely on stoichiometric oxidants and silanes, which can limit functional group compatibility and promote undesired side reactions.^3,4^ Reductive MHAT catalysis has recently emerged as an attractive orthogonal strategy, bypassing the need for external oxidants and hydride donors while enabling efficient access to carbon-centered radicals under mild conditions.^5–8^ Beyond classic hydrofunctionalization, these radical intermediates can offer opportunities for cascade processes and skeletal reorganization reactions.^9,10^

**Scheme 1:**
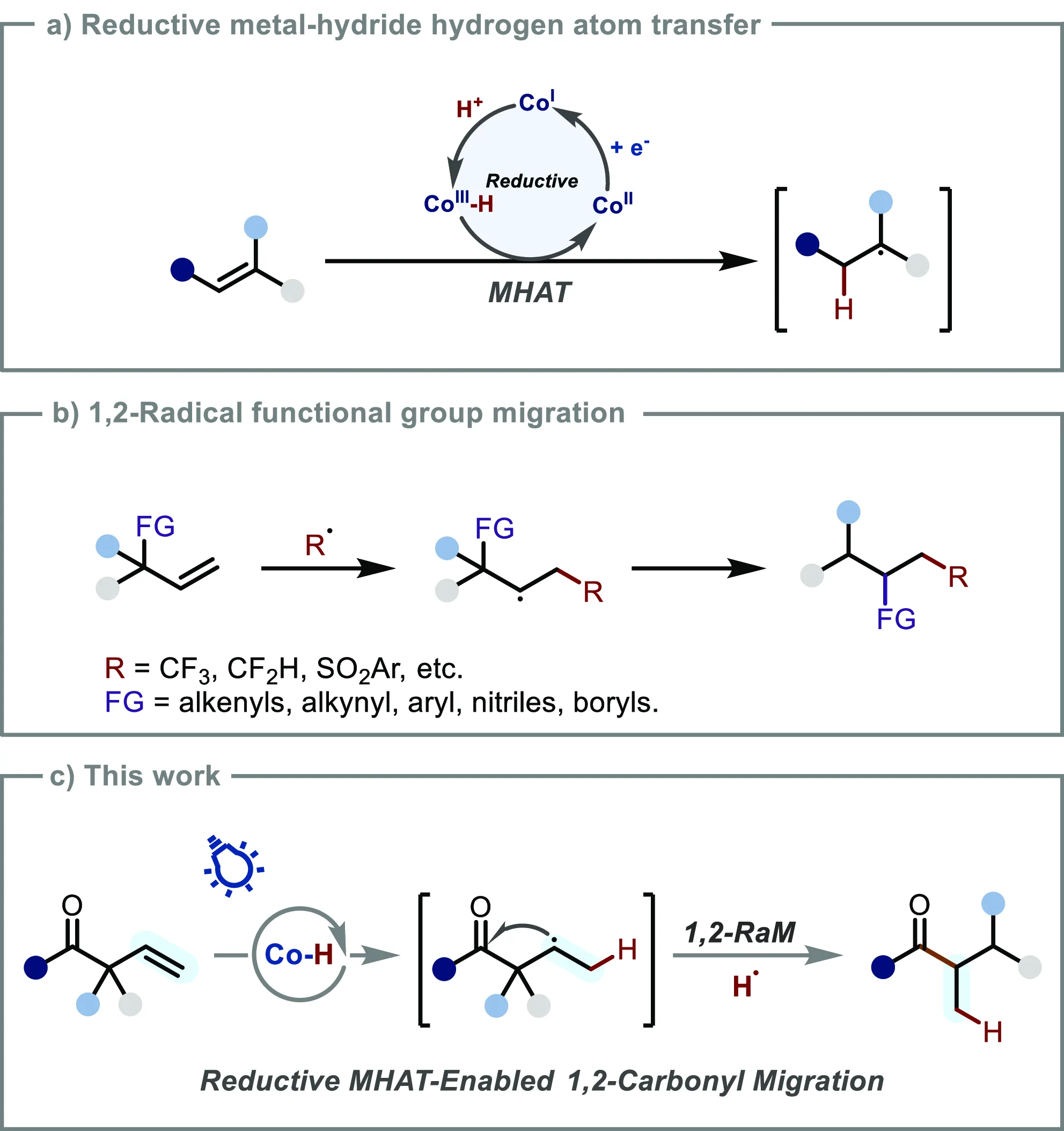
Context of This Work

Radical rearrangements constitute a valuable strategy for molecular editing through selective C–C bond cleavage and reconstruction.^11^ In particular, radical-mediated 1,2-migrations (1,2-RaM) provide direct access to structurally diversified frameworks and have been explored for the migration of alkenyl,^12^ alkynyl,^13^ aryl,^14^ boryl,^15^ and cyano groups (Scheme 1b).^16^ More specifically, 1,2-carbonyl migrations of β,γ-unsaturated ketones have been initiated by N-centered,^17^ fluoroalkyl,^18^ alkyl,^19^ and sulfur-centered radicals, including sulfonyl,^20,21^ SF_5_, and CF_3_SF_4_ species.^22^ As these cascades are initiated by radical addition, the installation of an external functional group derived from the radical precursor is required, thereby precluding access to simple carbo-skeleton frameworks.

Our group^9,23–28^ and others^29–31^ have developed cooperative photocatalysis/cobalt catalysis platforms for the mild generation of Co­(III)–H species. These systems have enabled a range of radical rearrangements, including 1,2-aryl migrations^24,32,33^ and Truce–Smiles rearrangements.^9,26^ More recently, the Ngai group reported an MHAT-enabled 1,2-radical acyloxy migration with a broad substrate scope and good functional group tolerance, although the reaction requires increased temperatures.^34^ Inspired by these advances, we questioned whether MHAT catalysis could be extended to other functional group migrations. Developing such transformations would expand the repertoire of MHAT-enabled radical rearrangements and provide alternative disconnections for the synthesis of structurally diverse molecular architectures.

Herein, we report a photocatalytic 1,2-carbonyl migration of β,γ-unsaturated ketones enabled by reductive MHAT catalysis (Scheme 1c). The reaction proceeds under mild conditions and provides efficient access to structurally rearranged ketones, establishing an approach to radical-mediated skeletal reorganization through synergistic cobalt and photoredox catalysis.

We began our investigation of the 1,2-carbonyl migration using 1-mesityl-2,2-dimethylbut-3-en-1-one (**1a**) as the model substrate under reductive MHAT conditions.^23^ Our previous studies have demonstrated that Hantzsch ester (HEH) can serve as both a sacrificial reductant and a proton source for the cobalt cycle.^25^ Preliminary screening revealed that cobalt catalysts efficiently promoted the transformation, with low catalyst loadings sufficient to afford the desired product in high yields. Initial experiments conducted in the presence of 2.0 equiv of pyridine as an additive furnished the desired product in an excellent 96% NMR yield (Table 1, entry 2). However, subsequent control experiments demonstrated that pyridine was not required for efficient product formation (see the Supporting Information for details).

**Table 1:**
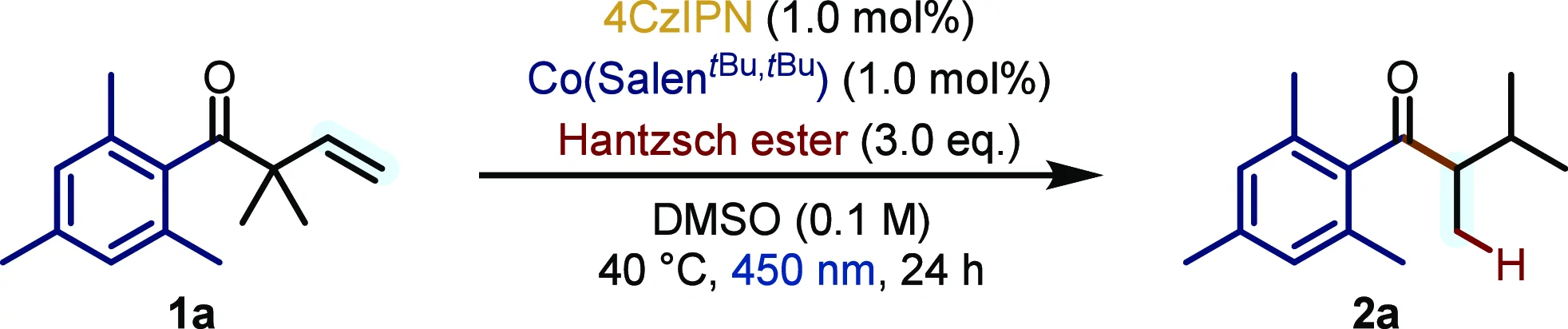
Reaction Discovery and Selected Optimization

entry	deviation from the standard conditions	yield (%)^a^
1	none	99 (91)^b^
2	pyridine (2.0 equiv) as an additive^c^	96
3	MeCN, DCE, or EtOAc as the solvent	60–69
4	MesAcrBF_4_/[Ru(bpy)_3_]Cl_2_ instead of 4CzIPN	<5/27
5	without Co(salen^*t*Bu,*t*Bu^)	0
6	without Hantzsch ester	0
7	without light	0
8	without 4CzIPN	34

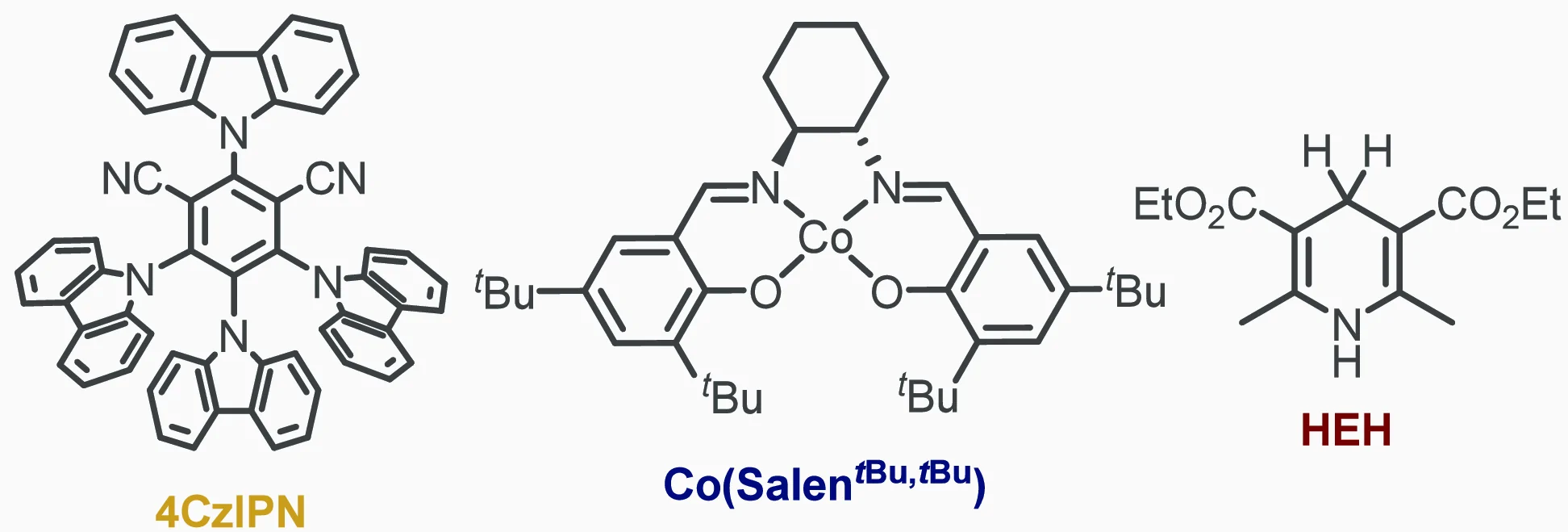

a Yield determined by ^1^H NMR using CH_2_Br_2_ as an internal standard.

b Isolated yields.

c Reaction time of 18 h.

Product formation was observed across a range of solvents, with dimethyl sulfoxide (DMSO) proving to be optimal for the transformation (Table 1, entry 3). Screening of various photocatalysts identified 4CzIPN as an effective catalyst for promoting the reaction (Table 1, entry 4). Control experiments further established that both the cobalt catalyst and Hantzsch ester were essential for efficient conversion, while no product formation was observed in the absence of light (Table 1, entries 5–7). Interestingly, the desired product was still obtained in moderate yield in the absence of the photocatalyst (Table 1, entry 8). This observation likely results from direct photoexcitation of the Hantzsch ester under blue light,^25^ allowing the transformation to proceed through a less efficient pathway and consequently leading to a diminished yield.

With the optimized conditions in hand, we evaluated the substrate scope using a range of β,γ-unsaturated ketones (Scheme 2a). Tri- and dimethyl-substituted substrates were first tested, affording products **2a** and **2b** in 91% and 72% yields, respectively, while the unsubstituted substrate gave desired product **2c** in 55% yield. Substrates bearing either electron-donating or electron-withdrawing substituents on the aryl ring were well tolerated, providing desired products **2d**–**2o** in moderate to good yields. In contrast, the *ortho*-substituted substrate (**2d**) afforded a lower yield, likely due to the altered steric positioning of the aryl ring, which brings the rearranged radical closer to the *ortho* position, thereby favoring a competing cyclization pathway. Fluorinated substrates, which are of broad interest because fluorine substitution can modulate metabolic stability and electronic properties,^35,36^ were also well tolerated. Both mono- and polyfluorinated substrates furnished products **2k** and **2l** in moderate yields, with *ortho*-, *meta*-, and *para*-substituted examples (**2m**–**o**, respectively) giving comparable results.

**Scheme 2:**
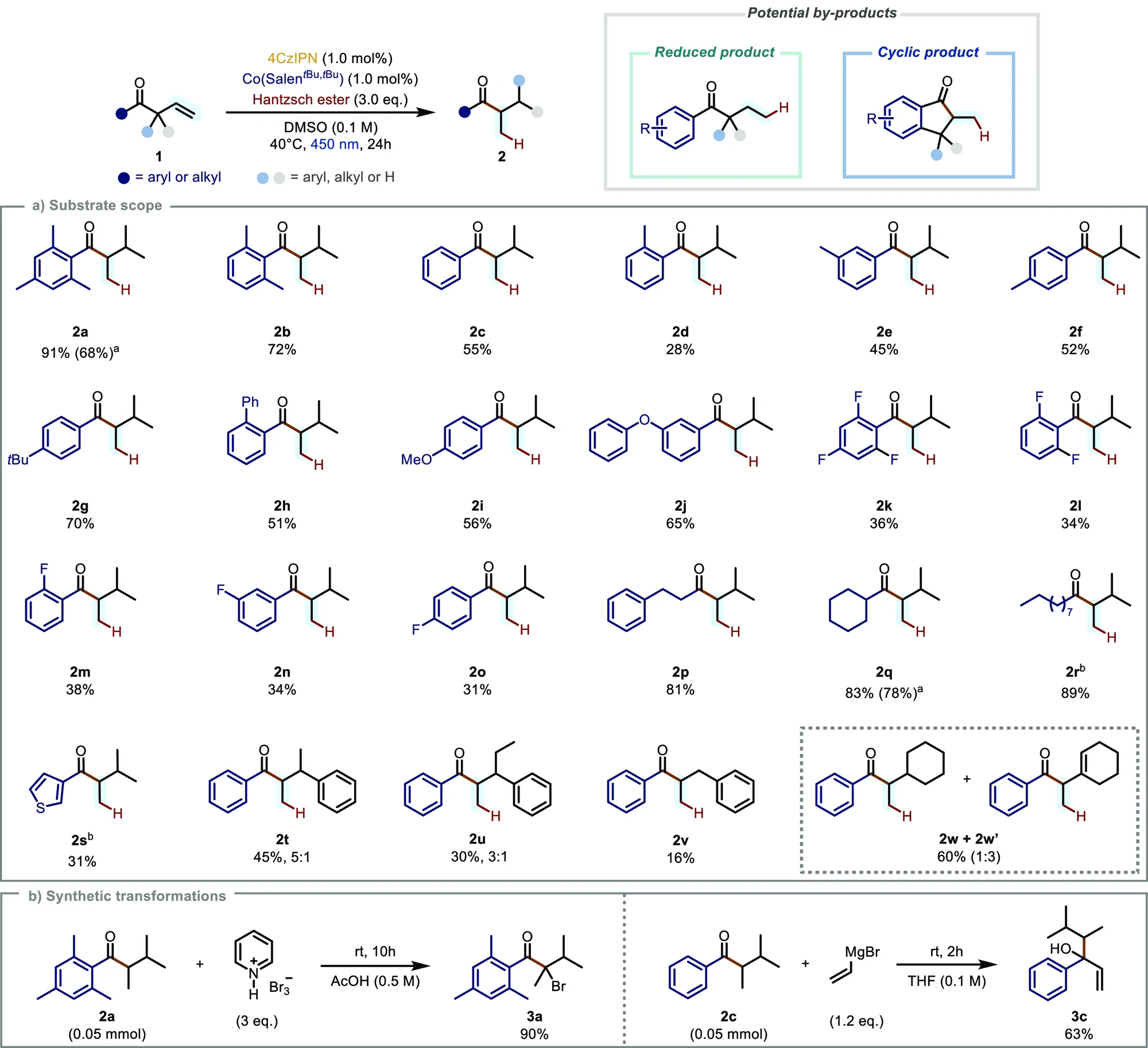
Substrate Scope^a^

a On a 1 mmol scale; reaction extended to 36 h.
b Using 2.0 equiv of pyridine as an additive.
c Unless otherwise stated, reactions were carried out on a 0.1 mmol scale, and all reported yields are isolated yields.

Aliphatic ketone substrates were also well tolerated under the standard conditions, affording desired products **2p**–**r** in high yields. However, long-chain aliphatic substrate **2r** required the addition of pyridine to achieve efficient conversion. Likewise, the thiophene-containing substrate afforded product **2s** in 31% yield only in the presence of pyridine. Allyl ketones bearing different α-substituents on the carbonyl group, including ethyl and phenyl groups, were also compatible with the reaction conditions, delivering products **2t**–**v** in moderate to good yields. For the mono-α-substituted substrate, desired product **2v** was obtained in only 16% yield because of competing reduction pathways and the challenging separation of the rearranged product from the corresponding reduced byproducts, which exhibited similar polarity. For the cyclohexyl-substituted substrate, a mixture of **2w** and **2w′** was obtained. We propose that alkene product **2w′** is formed via abstraction of the adjacent hydrogen atom from the rearranged radical intermediate. Furthermore, the α-unsubstituted substrate afforded no rearranged product, yielding only the competing reduction product. This result suggests that α,α-disubstitution plays an important role in promoting the transformation. The geminal substituents likely facilitate radical addition to the carbonyl through the Thorpe–Ingold effect and stabilize the radical formed upon ring opening, thereby shifting the equilibrium toward the rearranged product.

To demonstrate the preparative practicality of the method, the reactions furnishing **2a** and **2q** were conducted on a 1.0 mmol scale (Scheme 2a), affording the corresponding products in 68% and 78% isolated yields, respectively. Further transformations demonstrate the synthetic versatility of the products (Scheme 2b). Bromination of **2a** with pyridinium tribromide (3.0 equiv) in AcOH proceeded efficiently at the sterically congested α-position, providing tertiary α-bromoketone **3a** in 90% yield. Addition of vinylmagnesium bromide to **2c** afforded tertiary alcohol **3c** in 63% yield, introducing hydroxyl and terminal alkene functionalities as complementary handles for further elaboration.

Based on our previous studies and related reports,^25,28,37^ we propose the following mechanism (Scheme 3a). Upon irradiation with 450 nm light, 4CzIPN is excited to 4CzIPN*, which undergoes single-electron transfer (SET) with HEH to generate 4CzIPN^•–^ and HEH^•+^. Direct SET from 4CzIPN^•–^ (*E*_ox_ = −1.21 V vs SCE) to the Co­(II) species (*E*_red_ = −1.60 V vs SCE)^29^ is thermodynamically unfavorable. However, previous studies have shown that under visible-light irradiation, 4CzIPN can be converted into more reducing photocatalyst 4CzMeBN when using DMSO as the methyl source,^38^ consistent with our reaction conditions. Supporting this possibility, the *in situ* formation of 4CzMeBN was detected by ^1^H NMR and HRMS analyses (see the Supporting Information for details).

**Scheme 3:**
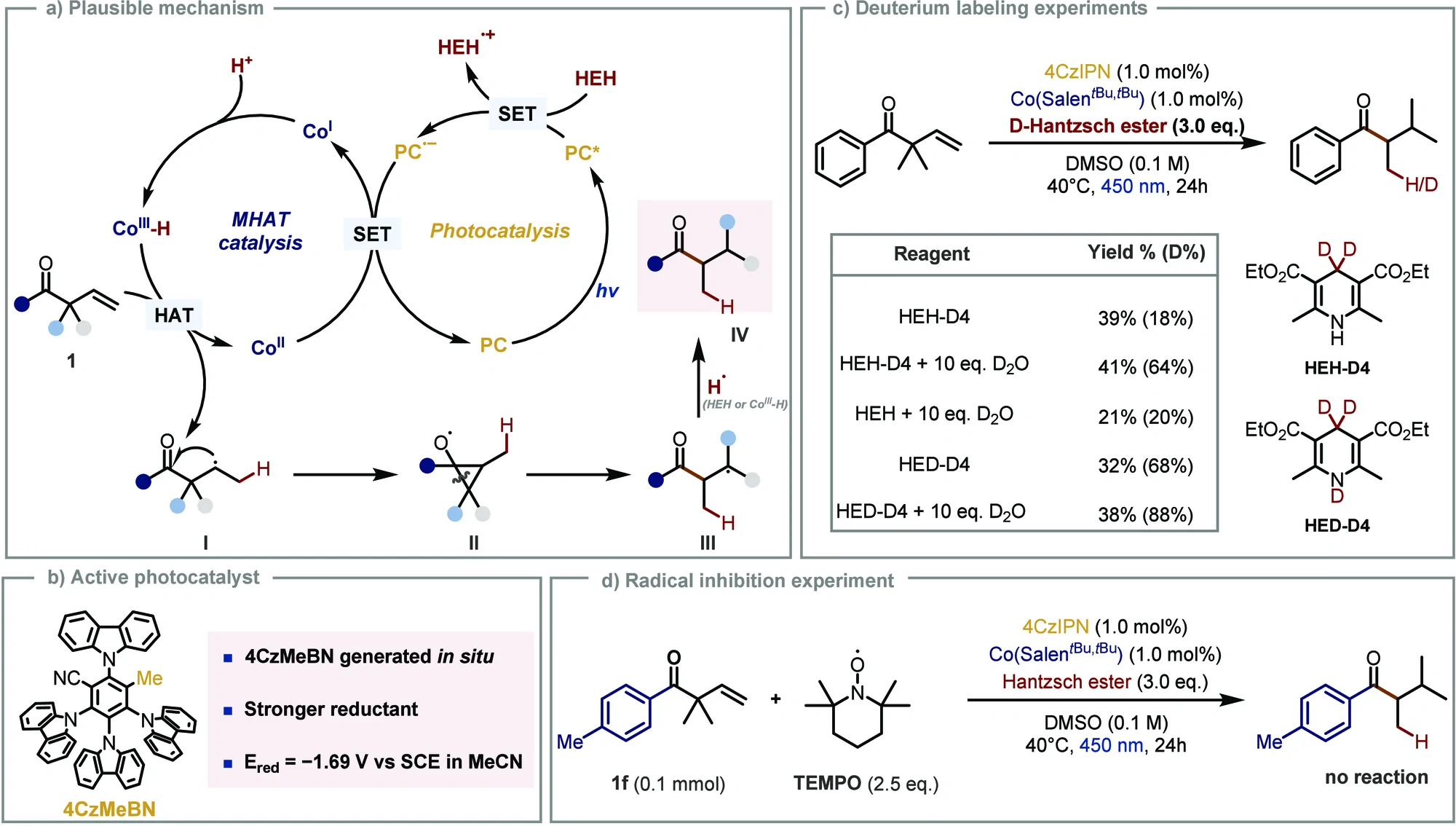
Plausible Mechanism and Mechanistic Studies

We therefore propose that 4CzMeBN is generated *in situ* to serve as the active photocatalyst (Scheme 3b). With an estimated reduction potential (*E*_red_) of −1.69 V vs SCE,^39^ this species is sufficiently reducing to convert Co­(II) into Co­(I) via SET. Subsequent protonation of the Co­(I) species by the reaction medium furnishes the key Co­(III)–H intermediate.

From this intermediate, hydrogen atom transfer (HAT) to unactivated alkene **1** generates alkyl radical intermediate **I**, together with Co­(II), thereby closing the MHAT catalytic cycle. Resulting radical **I** subsequently adds to the carbonyl group to afford cyclic intermediate **II**, which undergoes ring opening to furnish more stable radical intermediate **III**. Finally, termination via hydrogen atom transfer delivers rearranged product **IV**, with the hydrogen atom potentially originating from either HEH or a Co­(III)–H species. To probe the reaction mechanism, the reaction was performed in the presence of 2.5 equiv of TEMPO (Scheme 3d). Under these conditions, the reaction was completely suppressed, which supported the involvement of a radical pathway.

Next, we performed a series of deuterium-labeling experiments to elucidate the origin of the hydrogen atom transferred from cobalt in the initiation step of the reaction (Scheme 3c). Deuteration at both position C4 and the nitrogen atom of the Hantzsch ester resulted in partial deuteration of the product. The addition of D_2_O significantly increased the extent of deuterium incorporation. Collectively, these results are consistent with previous reports and suggest that proton exchange occurs within the reaction mixture, which provides several possible proton sources.^9,24,28^

In conclusion, we have developed a mild, reductive MHAT-enabled 1,2-carbonyl migration of β,γ-unsaturated ketones through the merger of cobalt catalysis and photocatalysis. The dual catalytic platform proceeds under mild conditions and supports a range of migrating carbonyl groups, providing practical and efficient access to simple branched ketone products without being bound to inclusion of specific functional groups.

## Supplementary Material



## Data Availability

The data underlying this study are available in the published article and its Supporting Information.

## Notes

Experimental procedures, NMR spectra, and analytical data of the products (PDF)
