# Supplementary material for: Photochemical Cobalt-Catalyzed Formal Hydroacylation via Radical 1,2-Carbonyl Migration
Source: Org Lett. 2026 Oct 23;28(44):13900–4. doi: 10.1021/acs.orglett.6c04309 (PMC13750077; doi:10.1021/acs.orglett.6c04309)
Supplement: Supplementary file 1 [file ol6c04309_si_001.pdf]

Supporting Information

Photochemical Cobalt-Catalyzed Formal Hydroacylation *via* Radical 1,2-Carbonyl Migration

He Chen, Ilaria Marilungo and Christopher J. Teskey*

Institute of Organic Chemistry, TU Braunschweig, Hagenring 30, 38106 Braunschweig, Germany

*Email: christopher.teskey@tu-braunschweig.de

Table of Contents

1. Materials and Methods 1
2. Selected Optimisation Reactions 2
2.1 Reaction time screening 2
2.2 Hantzsch ester loading screening 2
2.3 Cobalt catalyst loading screening 3
2.4 Additive loading screening 3
2.5 Photocatalyst screening 3
2.6 Solvent screening 4
2.7 Control experiments 4
3. Synthesis of Starting Materials: 5
3.1 General procedure for the synthesis of β,γ-unsaturated ketone (1)[1,2] 5
3.2 General procedure for the synthesis of β,γ-unsaturated ketone (2)[3] 5
3.3 Characterisation data: 7
4. General procedure and characterisation data of products 12
4.1 General procedure for the 1,2-Carbonyl Migration 12
4.2 Characterisation data 13
4.3 1 mmol-Scale examples of 1,2-Carbonyl Migration 19
5. Synthetic transformation 20
5.1 Characterisation data 21
6. Deuteration experiments 22
7. Radical inhibition experiment 25
8. Mechanistic investigation 25
8.1 Synthesis of 4CzMeBN 25
8.2 Control Reaction with 4CzMeBN 26
8.3 Investigation of the *In Situ* Formation of 4CzMeBN 27
9. References 29

1. Materials and Methods

Unless otherwise stated, all reactions were performed using standard Schlenk techniques. All reagents and starting materials were purchased at reagent grade and used as received unless otherwise stated. Anhydrous solvents were dried using an Innovative Technology PS-MD-5 solvent purification system. Thin layer chromatography (TLC) was performed on Merck Kieselgel 60 F254 aluminum plates with unmodified silica and visualized either under UV light or stained with potassium permanganate. Column chromatography was performed with Merck silica gel 60 (35 – 70 mesh). All noncommercially available reagents were prepared using reported literature procedure.

All ^{1}H, ^{13}C and ^{19}F NMR spectra were recorded at ambient temperature on either Bruker AVNeo600, Bruker AVIII400, Bruker AVIIIHD500 or Bruker AVII300 spectrometers. Chemical shifts (δ/ppm) were referenced to the residual solvent peak in ^{1}H (7.26 ppm for Chloroform-d) and ^{13}C spectra (77.16 ppm for Chloroform-d). Coupling constants (J) are given in Hz. Signals are described as br = broad, s = singlet, d = doublet, dd = doublet of doublets, t = triplet, q = quartet, p = quintet, h = sextet and m = multiplet, *app* = apparent.

High-resolution mass spectrometry (HRMS) was performed using Thermo Scientific LTQ Orbitrap XL spectrometer. Electrospray ionization (ESI) was performed using ThermoFisher Scientific LTQ Orbitrap Velos. Electrospray measurements were performed in direct infusion mode using a custom made microspray-device mounted on a Proxeon nanospray ion source. The microspray-device allows for the sample infusion through a stainless-steel capillary (90 µm ID). Accurate mass measurements in the orbitrap were performed using the lock mass option of the instrument control software using the cation of tetradecyltrimethylammonium bromide (256.29988 amu) as internal mass reference. GC/MS were measured on a Shimadzu Nexis GC-2030 as the gas chromatograph part and with a GCMS-QP2020 NX as the mass spectrometer part.

Unless otherwise stated, all photochemical reactions were carried out in 4 mL borosilicate-glass vials using an EvoluChem™ PhotoRedOx Box Duo (HepatoChem Inc.) equipped with two EvoluChem™ 450PF LED lamps (HCK1012-01-002; CREE XPE LEDs; λ = 450-455 nm, λ_{max} ≈ 450 nm; 18 W electrical power per lamp). The manufacturer reports an average irradiance of 34 mW cm^{-2} for the 450PF LED measured in the PhotoRedOx Box. The reaction vials were held in the manufacturer supplied vial holder at a distance of approximately 80 mm from the LED source. No optical filters were used.

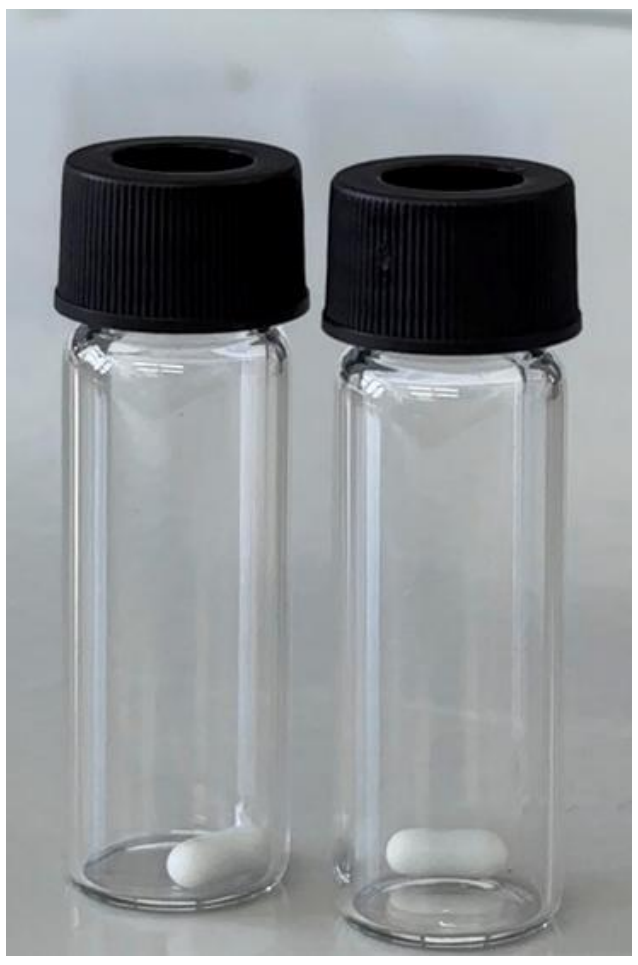

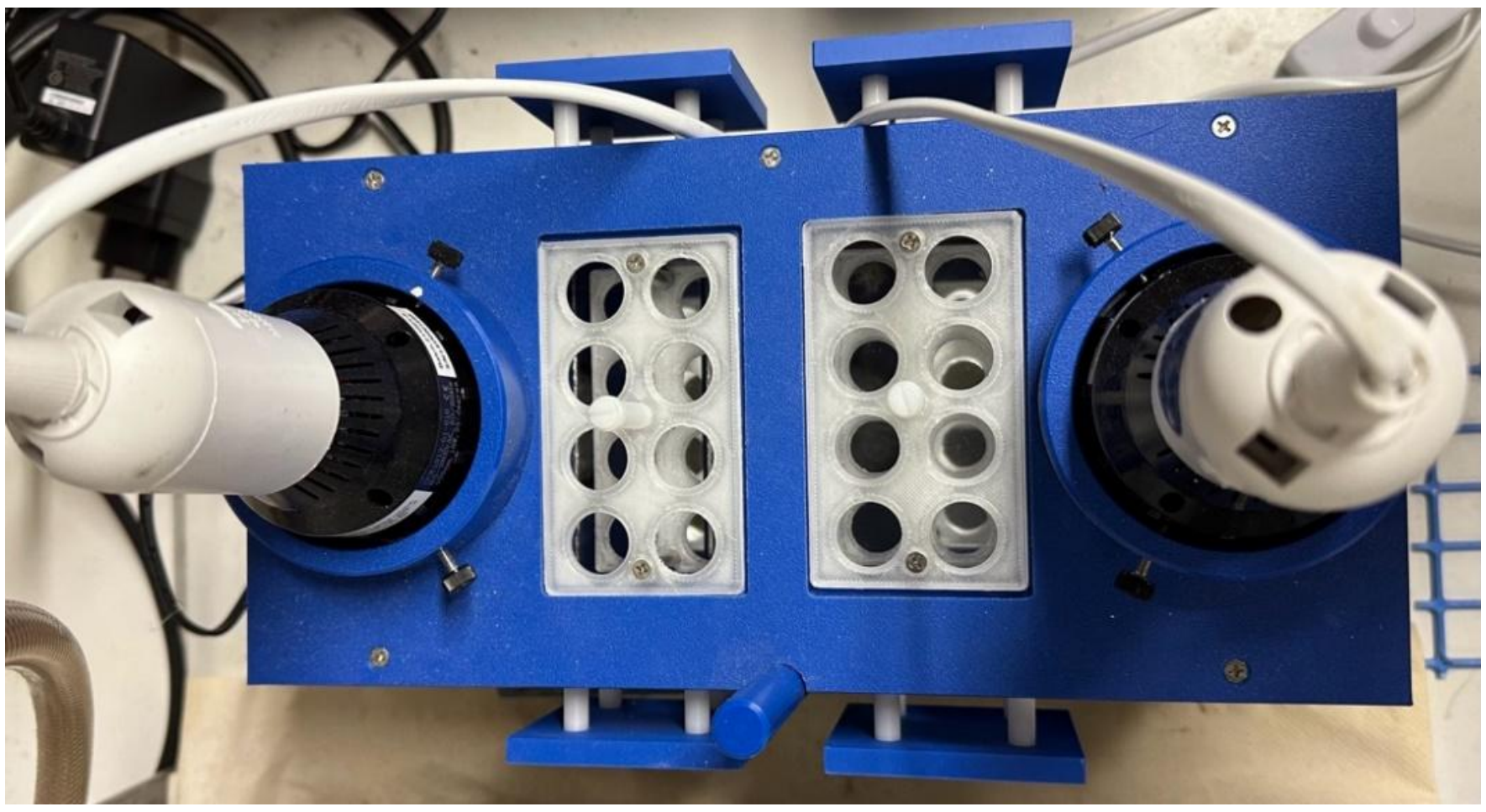

2. Selected Optimisation Reactions

Unless otherwise stated, the optimization has been carried out on a 0.1 mmol scale.

2.1 Reaction time screening

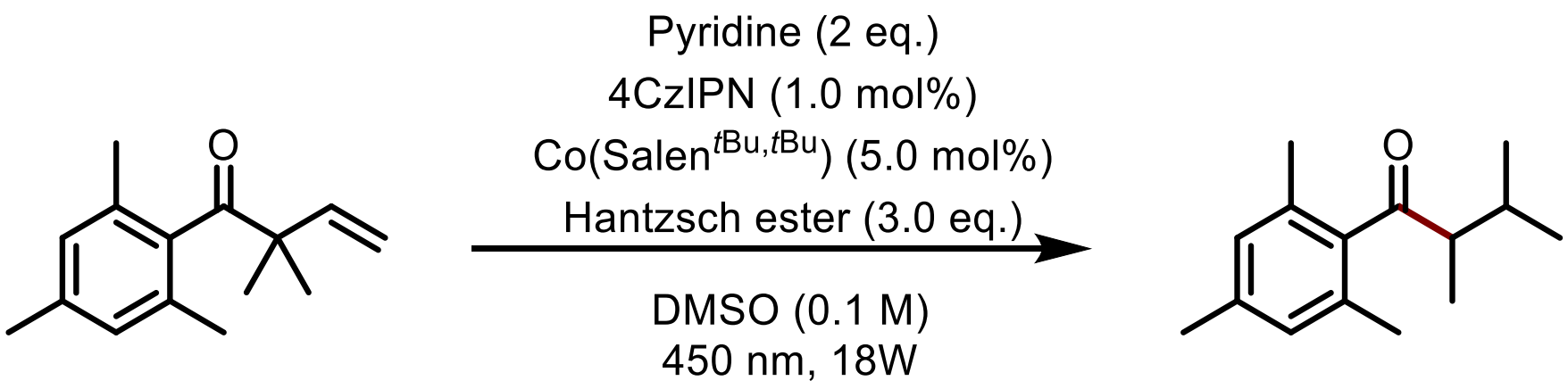

Entry	*Variations*	*Product (%)*[a]
1	18h	97
2	24h	98
3	36h	85
4	48h	79

[a]yield determined by ^{1}H-NMR analysis with CH_2Br_2 as an internal standard.

2.2 Hantzsch ester loading screening

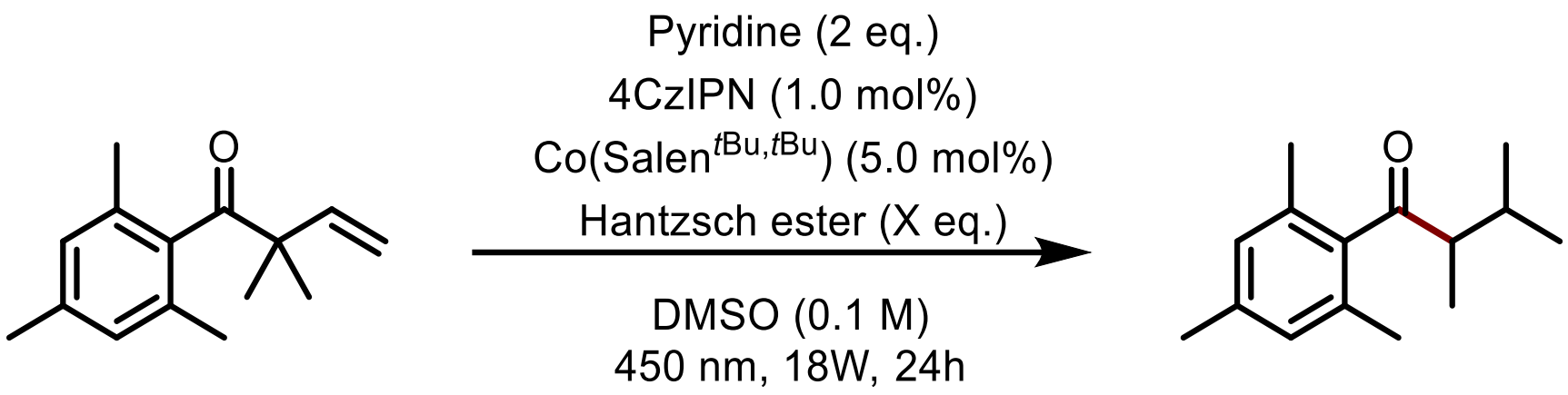

Entry	*Variations*	*Product (%)*[a]
1	HEH (5 eq)	87
2	HEH (4 eq)	97
3	HEH (3 eq)	99
4	HEH (2 eq)	56
5	HEH (1 eq)	31

[a]yield determined by ^{1}H-NMR analysis with CH_2Br_2 as an internal standard.

2.3 Cobalt catalyst loading screening

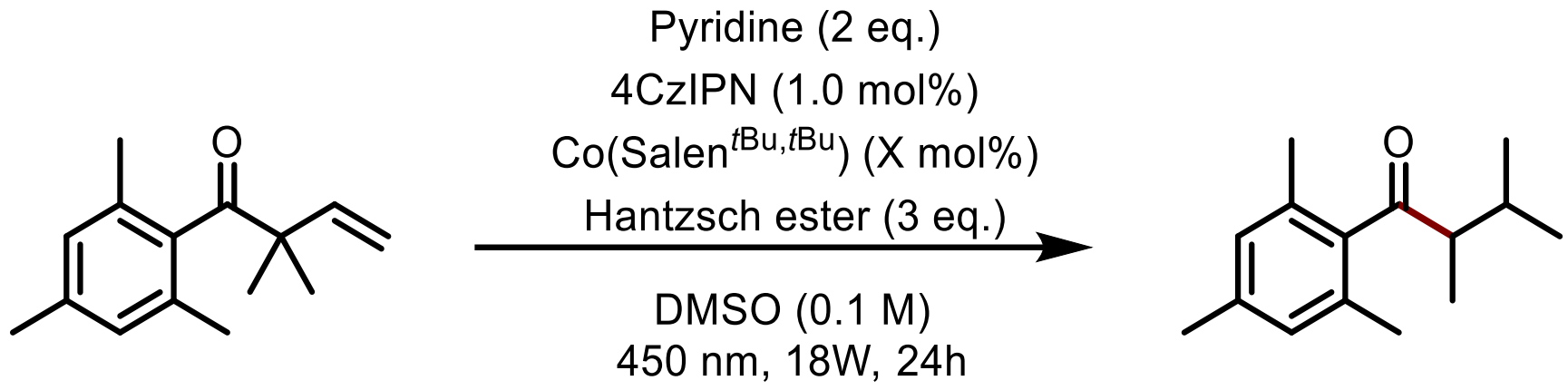

Entry	*Variations*	*Product (%)*[a]
1	[Co] (3 mol%)	95
2	[Co] (2 mol%)	99
3	[Co] (1 mol%)	99

[a]yield determined by ^{1}H-NMR analysis with CH_2Br_2 as an internal standard.

2.4 Additive loading screening

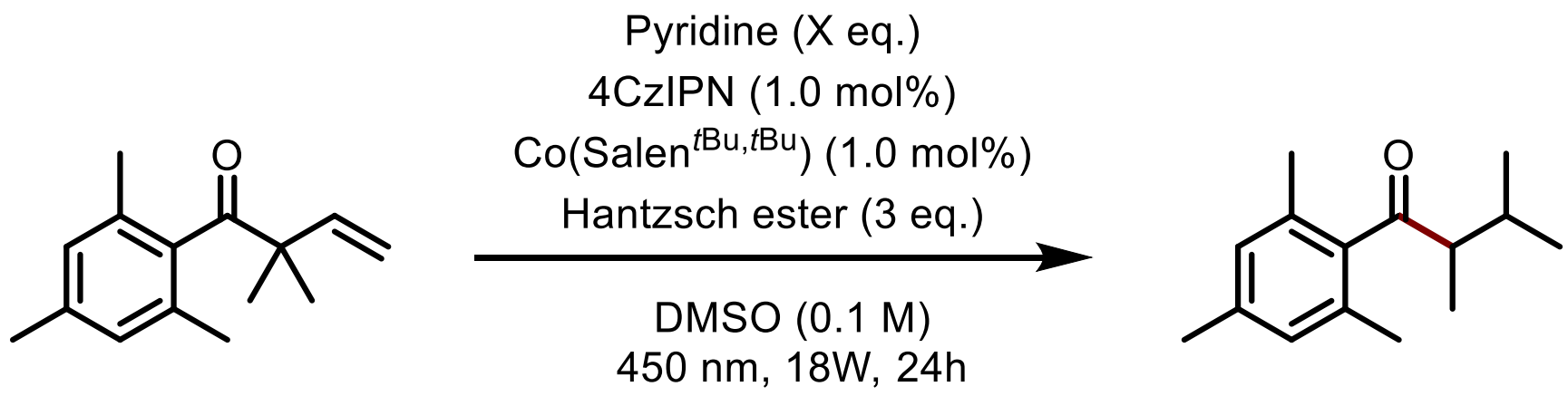

Entry	*Variations*	*Product (%)*[a]
1	Pyridine (2 eq.)	96
2	Pyridine (1 eq.)	94
3	w/o pyridine	99% (91%)[b]

[a]yield determined by ^{1}H-NMR analysis with CH_2Br_2 as an internal standard. [b]isolated yield.

2.5 Photocatalyst screening

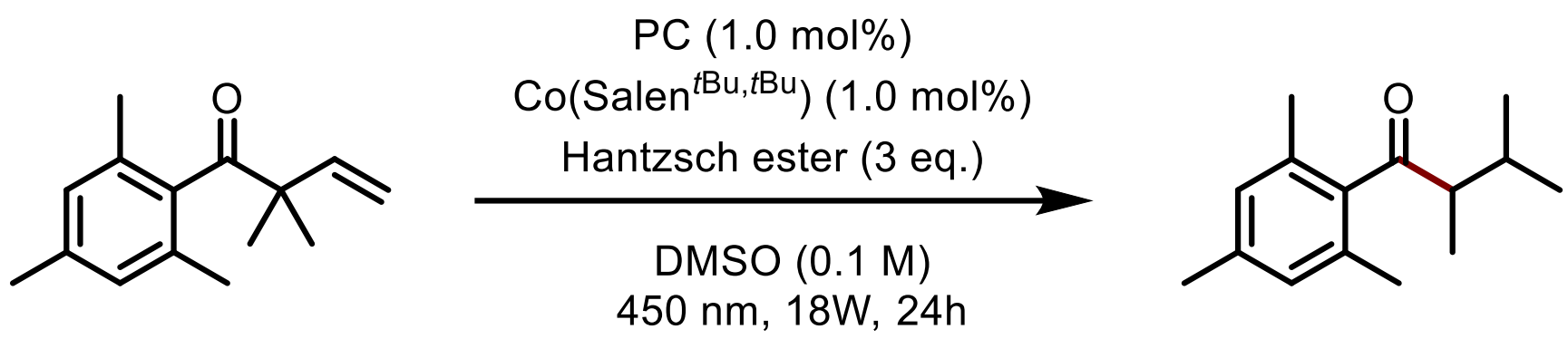

Entry	*Variations*	*Product (%)*[a]
1	$Ir(dtbbpy)(ppy)_2PF_6$ (1 mol%)	70
2	$[Ru(bpy)_3]Cl_2$ (1 mol%)	27
3	$MesAcrBF_4$ (1 mol%)	Traces

[a]yield determined by ^{1}H-NMR analysis with CH_2Br_2 as an internal standard.

2.6 Solvent screening

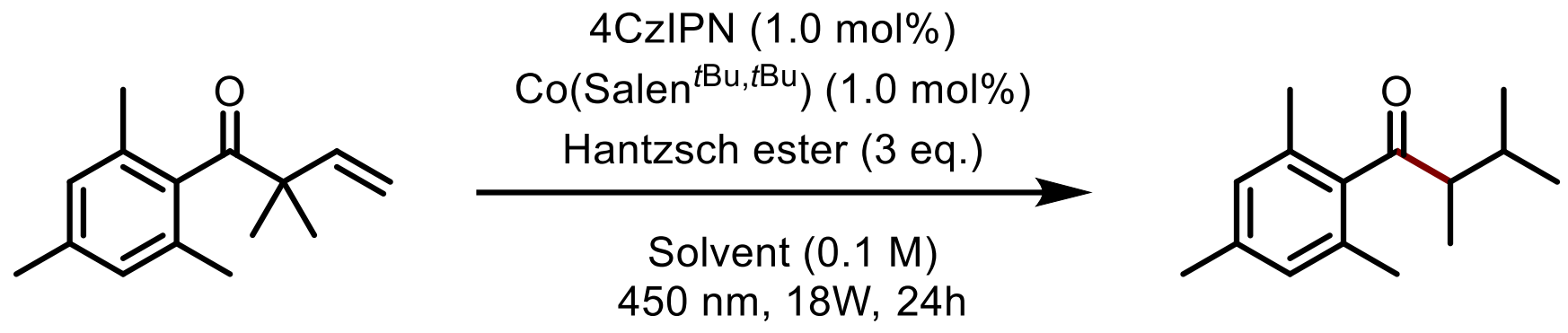

Entry	*Variations*	*Product (%)[a]*
1	MeCN (0.1 M)	67
2	DCE (0.1 M)	60
3	EtOAc (0.1 M)	69

[a]yield determined by ^{1}H-NMR analysis with CH_2Br_2 as an internal standard.

2.7 Control experiments

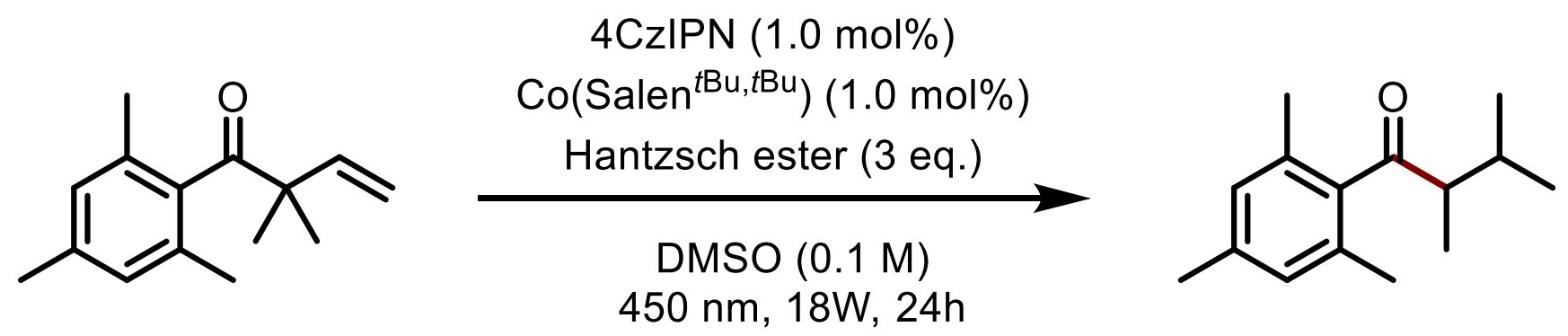

Entry	*Variations*	*Product (%)[a]*
1	w/o [Co]	0
2	w/o PC	34
3	w/o HEH	0
4	w/o light	0

[a]yield determined by ^{1}H-NMR analysis with CH_2Br_2 as an internal standard.

3. Synthesis of Starting Materials:

3.1 General procedure for the synthesis of β,γ-unsaturated ketone (1)[1,2]

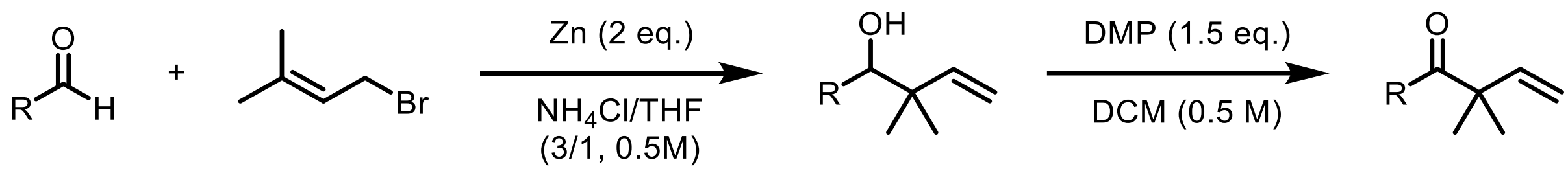

To a suspension of the aldehyde (10 mmol, 1.0 equiv) in THF/saturated NH_4Cl (v/v = 1:3, 0.5 M) at 0 °C were added 3,3-dimethylallyl bromide (15 mmol, 1.5 equiv) and zinc powder (1.31 g, 20 mmol, 2.0 equiv) portionwise. The reaction mixture was stirred at room temperature overnight. The organic layer was separated, and the aqueous layer was extracted with EtOAc (×3). The combined organic layers were washed with brine, dried over $MgSO_4$, filtered, and concentrated *in vacuo*. The crude product was carried for the second step without further purification.

A solution of the homoallylic alcohol (1.0 equiv) in DCM (0.5 M) was added to a stirred solution of Dess–Martin periodinane (1.5 equiv) in DCM (0.5 M). The reaction mixture was stirred for 2 h and monitored by TLC until complete consumption of the starting material. The solvent was then removed *in vacuo*, and the resulting residue was purified by silica gel column chromatography to afford the corresponding *β,γ*-unsaturated ketones **1**.

3.2 General procedure for the synthesis of β,γ-unsaturated ketone (2)[3]

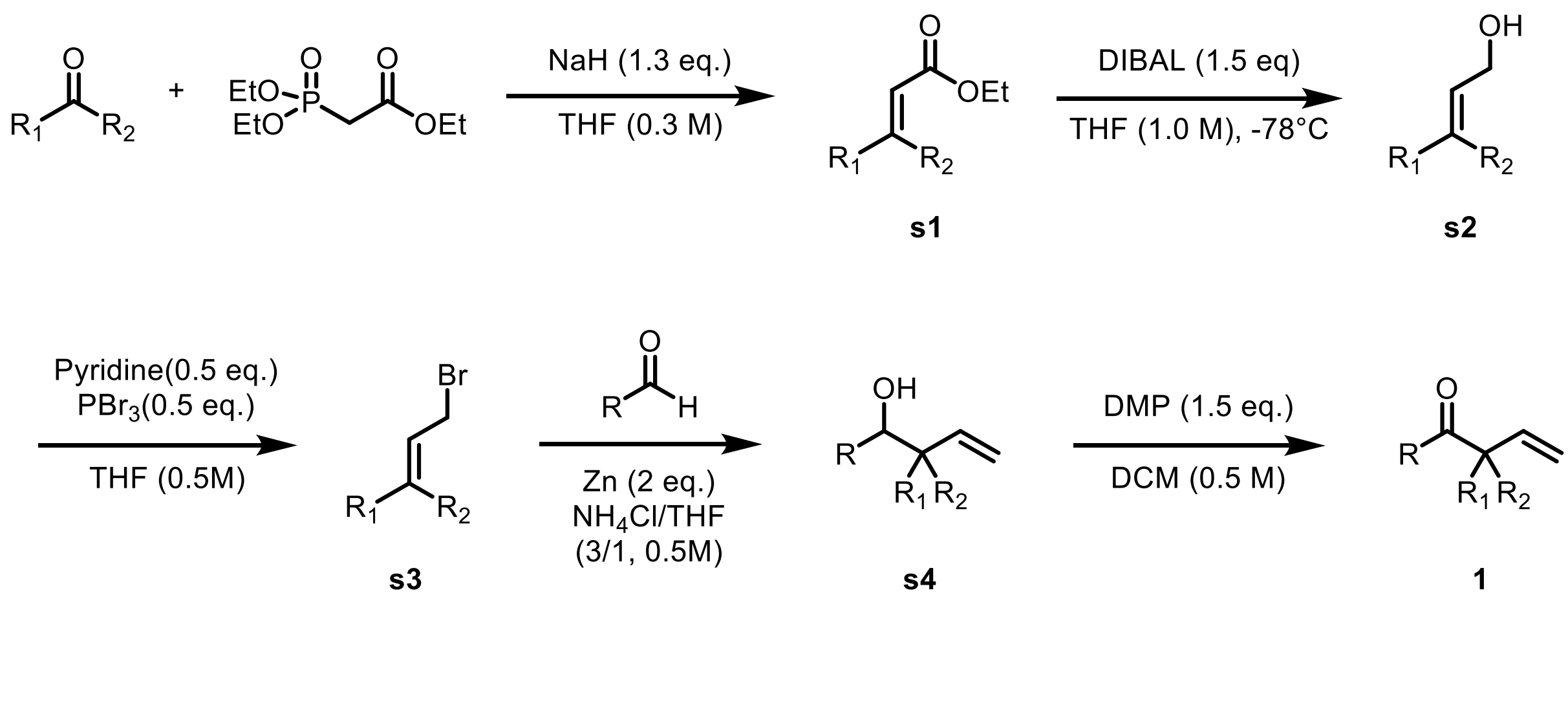

To a suspension of sodium hydride (1.3 equiv) in anhydrous THF (0.3 M) at 0 °C was added triethyl phosphonoacetate (1.5 equiv). The mixture was stirred at room temperature for 1 h, after which the ketone (1.0 equiv) was added. The reaction mixture was stirred for an additional 5 h, and reaction progress was monitored by TLC. Upon completion, the reaction mixture was cooled to 0 °C, quenched with water, and extracted with EtOAc (3 ×). The combined organic layers were washed with saturated

brine, dried over $MgSO_4$, filtered, and concentrated in vacuo. The crude product was purified by silica gel column chromatography to afford the corresponding product **s1** for the next step.

The resulting product **s1** in anhydrous THF (1.0 M) was cooled to −78 °C. DIBAL (1.5 equiv) was added dropwise, and the reaction mixture was stirred at −78 °C for 2 h. After completion, the reaction mixture was allowed to warm to room temperature and then quenched. The mixture was extracted with EtOAc, and the combined organic layers were washed with saturated brine, dried over $MgSO_4$, filtered, and concentrated in vacuo. The crude product **s2** was used directly in the next step without further purification.

To a solution of resulting product **s2** in THF (0.5 M) at 0 °C were added pyridine (0.5 equiv) and phosphorus tribromide (0.5 equiv) dropwise. The reaction mixture was allowed to warm to room temperature and stirred for 5 h. Upon completion, the mixture was cooled to 0 °C and quenched with ice–water. The reaction mixture was extracted with EtOAc (3 ×), and the combined organic layers were washed with saturated brine, dried over $MgSO_4$, filtered, and concentrated in vacuo. The crude product **s3** was used directly in the next step without further purification.

To a suspension of the aldehyde (1.0 equiv) in THF/saturated NH_4Cl (v/v = 1:3, 0.5 M) at 0 °C were added resulting crude product **s3** (1.5 equiv) and zinc powder (2.0 equiv) portionwise. The reaction mixture was stirred at room temperature overnight. The organic layer was separated, and the aqueous layer was extracted with EtOAc (3 ×). The combined organic layers were washed with brine, dried over $MgSO_4$, filtered, and concentrated in vacuo. The crude product **s4** was carried directly to the next step without further purification.

A solution of the homoallylic alcohol **s4** (1.0 equiv) in DCM (0.5 M) was added to a stirred solution of Dess–Martin periodinane (1.5 equiv) in DCM (0.5 M). The reaction mixture was stirred for 2 h and monitored by TLC until complete consumption of the starting material. The solvent was removed in vacuo, and the residue was purified by silica gel column chromatography to afford the corresponding *β,γ*-unsaturated ketones **1**.

3.3 Characterisation data:

1-mesityl-2,2-dimethylbut-3-en-1-one (1a)

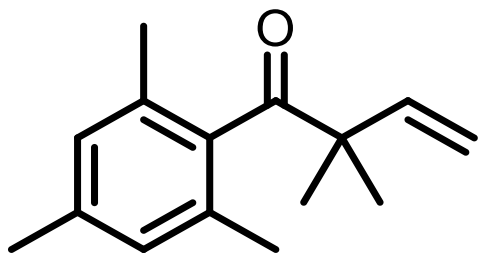

Prepared following general procedure (1). **1a** was obtained after purification by column chromatography (pentane : EtOAc 100 :1) as a colorless oil in 63% yield (1.36 g, 6.30 mmol). **^{1}H NMR** (300 MHz, $CDCl_3$) δ 6.80 (s, 2H), 6.08 (dd, *J* = 17.4, 10.6 Hz, 1H), 5.12 (dd, *J* = 17.4, 0.9 Hz, 1H), 5.05 (dd, *J* = 10.6, 0.9 Hz, 1H), 2.27 (s, 3H), 2.13 (s, 6H), 1.33 (s, 6H). **^{13}C NMR** (76 MHz, $CDCl_3$) δ 216.6, 143.8, 139.5, 137.8, 132.3, 128.4, 112.9, 51.4, 25.3, 21.10, 20.5. **HRMS(ESI):** calculated for $C_{15}H_{20}ONa$ $[M+Na]^+$: 239.1406, found: 239.1407. These data are in accordance with those reported previously.[1]

1-(2,6-dimethylphenyl)-2,2-dimethylbut-3-en-1-one (1b)

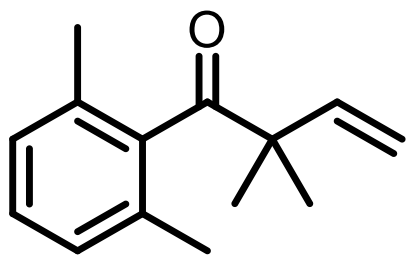

Prepared following general procedure (1). **1b** was obtained after purification by column chromatography (pentane : EtOAc 100 :1) as a pale yellow oil in 58% yield (1.17 g, 5.80 mmol). **^{1}H NMR** (400 MHz, $CDCl_3$) δ 7.14 – 7.10 (m, 1H), 7.00 – 6.96 (m, 2H), 6.09 (dd, *J* = 17.4, 10.6 Hz, 1H), 5.13 (dd, *J* = 17.4, 0.9 Hz, 1H), 5.05 (dd, *J* = 10.6, 0.9 Hz, 1H), 2.17 (s, 6H), 1.34 (s, 6H). **^{13}C NMR** (101 MHz, $CDCl_3$) δ 216.3, 143.7, 142.2, 132.4, 128.1, 127.7, 113.0, 51.4, 25.3, 20.6. **HRMS(ESI):** calculated for $C_{14}H_{18}ONa$ $[M+Na]^+$: 225.1250, found: 225.1250.

2,2-dimethyl-1-phenylbut-3-en-1-one (1c)

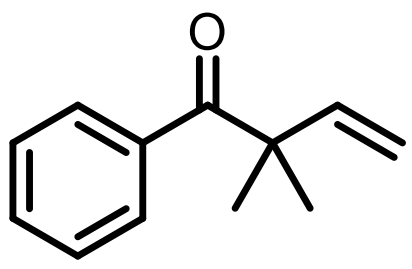

Prepared following general procedure (1). **1c** was obtained after purification by column chromatography (pentane : EtOAc 100 :1) as a colorless oil in 44% yield (765.0 mg, 4.40 mmol). **^{1}H NMR** (400 MHz, $CDCl_3$) δ 7.90 – 7.85 (m, 2H), 7.47 – 7.42 (m, 1H), 7.39 – 7.33 (m, 2H), 6.18 (dd, *J* = 17.5, 10.6 Hz, 1H), 5.28 – 5.16 (m, 2H), 1.39 (s, 6H). **^{13}C NMR** (101 MHz, $CDCl_3$) δ 204.7, 144.0, 137.2, 131.7, 129.4, 128.0, 114.1, 50.2, 26.1. **HRMS(ESI):** calculated for $C_{12}H_{14}ONa$ $[M+Na]^+$: 197.0937, found: 197.0938. These data are in accordance with those reported previously.[1]

2,2-dimethyl-1-(o-tolyl)but-3-en-1-one (1d)

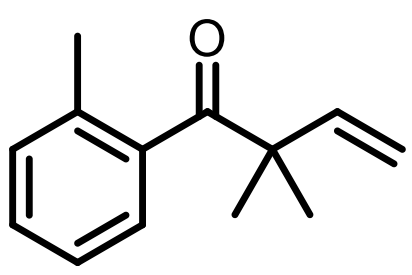

Prepared following general procedure (1). **1d** was obtained after purification by column chromatography (pentane : EtOAc 100 :1) as a colorless oil in 47% yield (883.0 mg, 4.70 mmol). **^{1}H NMR** (300 MHz, $CDCl_3$) δ 7.29 – 7.19 (m, 3H), 7.19 – 7.10 (m, 1H), 6.03 (dd, *J* = 17.4, 10.6 Hz, 1H), 5.24 – 5.10 (m, 2H), 2.23 (s, 3H), 1.33 (s, 6H). **^{13}C NMR** (76 MHz, $CDCl_3$) δ 211.1, 142.8, 140.6, 134.6, 130.9, 129.1, 125.6, 124.8, 114.3, 51.5, 24.7, 20.1. **HRMS(ESI):** calculated for $C_{13}H_{16}ONa$ $[M+Na]^+$: 211.1093, found: 211.1094. These data are in accordance with those reported previously.[4]

2,2-dimethyl-1-(m-tolyl)but-3-en-1-one (1e)

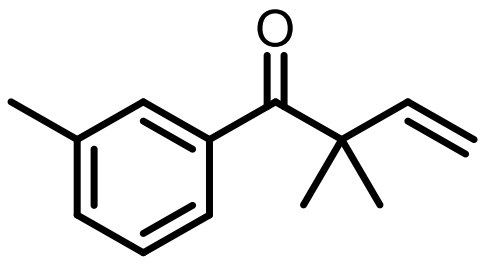

Prepared following general procedure (1). **1e** was obtained after purification by column chromatography (pentane : EtOAc 100 :1) as a colorless oil in 60% yield (1.13 g, 6.00 mmol). **^{1}H NMR** (300 MHz, $CDCl_3$) δ 7.69 – 7.62 (m, 2H), 7.29 – 7.21 (m, 2H), 6.18 (dd, *J* = 17.6, 10.6 Hz, 1H), 5.29 – 5.14 (m, 2H), 2.37 (s, 3H), 1.39 (s, 6H). **^{13}C NMR** (76 MHz, $CDCl_3$) δ 205.1, 144.1, 137.9, 137.4, 132.5, 129.8, 127.8, 126.5, 114.1, 50.3, 26.2, 21.6. **HRMS(ESI):** calculated for $C_{13}H_{16}ONa$ $[M+Na]^+$: 211.1093, found: 211.1094. These data are in accordance with those reported previously.[4]

2,2-dimethyl-1-(p-tolyl)but-3-en-1-one (1f)

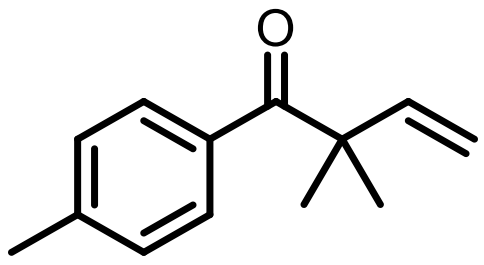

Prepared following general procedure (1). **1f** was obtained after purification by column chromatography (pentane : EtOAc 100 :1) as a colorless oil in 60% yield (1.13 g, 6.00 mmol). **^{1}H NMR** (400 MHz, $CDCl_3$) δ 7.86 – 7.78 (m, 2H), 7.21 – 7.13 (m, 2H), 6.19 (dd, *J* = 17.5, 10.6 Hz, 1H), 5.26 – 5.16 (m, 2H), 2.40 – 2.34 (m, 3H), 1.39 (s, 6H). **^{13}C NMR** (101 MHz, $CDCl_3$) δ 204.0, 144.3, 142.4, 134.2, 129.7, 128.7, 113.9, 50.1, 26.3, 21.6. **HRMS(ESI):** calculated for $C_{13}H_{16}ONa$ $[M+Na]^+$: 211.1093, found: 211.1094. These data are in accordance with those reported previously.[2]

1-(4-(tert-butyl)phenyl)-2,2-dimethylbut-3-en-1-one (1g)

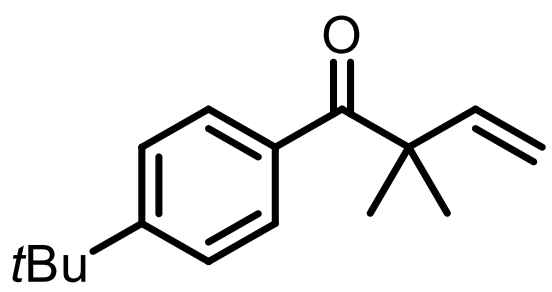

Prepared following general procedure (1). **1g** was obtained after purification by column chromatography (pentane : EtOAc 100 :1) as a colorless oil in 58% yield (1.33 g, 5.80 mmol). **^{1}H NMR** (300 MHz, $CDCl_3$) δ 7.92 – 7.82 (m, 2H), 7.44 – 7.34 (m, 2H), 6.20 (dd, *J* = 17.5, 10.6 Hz, 1H), 5.32 – 5.15 (m, 2H), 1.40 (s, 6H), 1.32 (s, 9H). **^{13}C NMR** (76 MHz, $CDCl_3$) δ 204.0, 155.4, 144.3, 134.1, 129.7, 125.0, 113.9, 50.17, 35.1, 31.2, 26.3. **HRMS(ESI):** calculated for $C_{16}H_{22}ONa$ $[M+Na]^+$: 253.1563, found: 253.1564. These data are in accordance with those reported previously.[4]

1-([1,1'-biphenyl]-2-yl)-2,2-dimethylbut-3-en-1-one (1h)

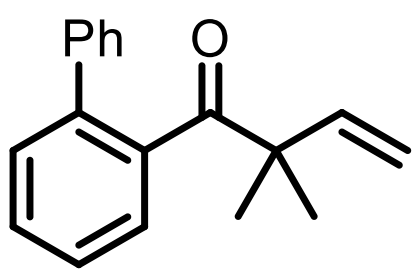

Prepared following general procedure (1). **1h** was obtained after purification by column chromatography (pentane : EA 100 :1) as a colorless oil in 31% yield (775.0 mg, 3.10 mmol). **^{1}H NMR** (400 MHz, $CDCl_3$) δ 7.47 – 7.30 (m, 8H), 7.20 – 7.13 (m, 1H), 5.68 (dd, *J* = 17.4, 10.6 Hz, 1H), 4.92 – 4.80 (m, 2H), 1.00 (s, 6H). **^{13}C NMR** (151 MHz, $CDCl_3$) δ 213.2, 142.6, 141.0, 140.9, 138.6, 129.9, 129.7, 129.0, 128.5, 127.7, 126.7, 126.2, 113.6, 51.4, 24.5. **HRMS(ESI):** calculated for $C_{18}H_{18}ONa$ $[M+Na]^+$: 273.1250, found: 273.1252.

1-(4-methoxyphenyl)-2,2-dimethylbut-3-en-1-one (1i)

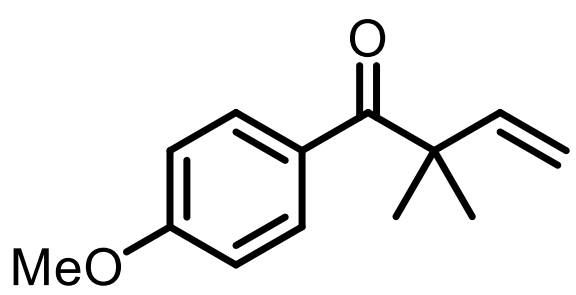

Prepared following general procedure (1). **1i** was obtained after purification by column chromatography (pentane : EtOAc 20 :1) as a colorless oil in 45% yield (918.0 mg, 4.50 mmol). **^{1}H NMR** (300 MHz, $CDCl_3$) δ 7.96 (d, *J* = 9.0 Hz, 2H), 6.86 (d, *J* = 9.0 Hz, 2H), 6.20 (dd, *J* = 17.6, 10.6 Hz, 1H), 5.28 – 5.13 (m, 2H), 3.84 (s, 3H), 1.39 (s, 6H). **^{13}C NMR** (76 MHz, $CDCl_3$) δ 202.5, 162.6, 144.6, 132.2, 129.3, 113.7,

113.2, 55.5, 50.0, 29.8, 26.5. **HRMS(ESI):** calculated for $C_{13}H_{16}O_2Na$ $[M+Na]^+$: 227.1042, found: 227.1043. These data are in accordance with those reported previously.[5]

2,2-dimethyl-1-(3-phenoxyphenyl)but-3-en-1-one (1j)

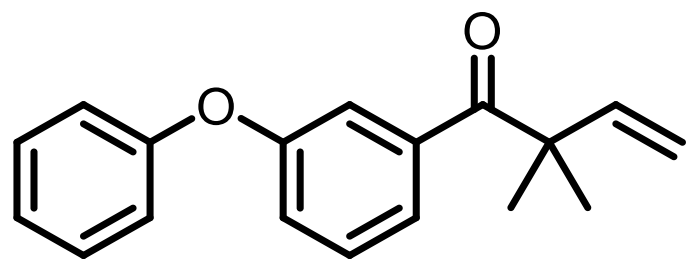

Prepared following general procedure (1). **1j** was obtained after purification by column chromatography (pentane : EtOAc 20 :1) as a colorless oil in 43% yield (1.14 g, 4.30 mmol). **^{1}H NMR** (400 MHz, $CDCl_3$) δ 7.60 – 7.57 (m, 1H), 7.52 – 7.50 (m, 1H), 7.38 – 7.31 (m, 3H), 7.18 – 7.08 (m, 2H), 7.06 – 6.97 (m, 2H), 6.12 (dd, *J* = 17.6, 10.7 Hz, 1H), 5.21 – 5.10 (m, 2H), 1.37 (s, 6H). **^{13}C NMR** (101 MHz, $CDCl_3$) δ 204.0, 157.3, 156.7, 143.6, 138.8, 130.0, 129.4, 123.9, 123.9, 121.8, 119.4, 119.2, 114.3, 50.3, 26.0. **HRMS(ESI):** calculated for $C_{18}H_{18}O_2Na$ $[M+Na]^+$: 289.1199, found: 289.1201.

2,2-dimethyl-1-(2,4,6-trifluorophenyl)but-3-en-1-one (1k)

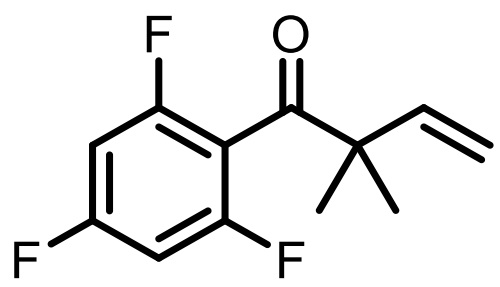

Prepared following general procedure (1). **1k** was obtained after purification by column chromatography (pentane : EtOAc 50 :1) as a colorless oil in 34% yield (775.2 mg, 3.40 mmol). **^{1}H NMR** (300 MHz, $CDCl_3$) δ 6.74 – 6.59 (m, 2H), 5.95 (ddt, *J* = 17.6, 10.4, 0.8 Hz, 1H), 5.16 (q, *J* = 0.8 Hz, 1H), 5.12 (dd, *J* = 5.2, 0.8 Hz, 1H), 1.33 (t, *J* = 0.8 Hz, 6H). **^{13}C NMR** (76 MHz, $CDCl_3$) δ 202.5, 164.7, 161.4, 160.9 – 155.5 (m), 140.7, 115.2, 101.1 – 100.2 (m), 52.0, 23.4. **^{19}F NMR** (376 MHz, $CDCl_3$) δ -106.4 (tt, *J* = 8.7, 6.8 Hz, 1F), -108.2 (dd, *J* = 8.1, 6.5 Hz, 2F). **HRMS(ESI):** calculated for $C_{12}H_{11}F_3ONa$ $[M+Na]^+$: 251.0654, found: 251.0655.

1-(2,6-difluorophenyl)-2,2-dimethylbut-3-en-1-one (1l)

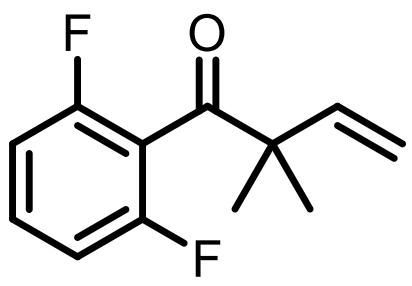

Prepared following general procedure (1). **1l** was obtained after purification by column chromatography (pentane : EtOAc 50 :1) as a colorless oil in 58% yield (1.20 g, 5.80 mmol). **^{1}H NMR** (300 MHz, $CDCl_3$) δ 7.39 – 7.23 (m, 1H), 6.96 – 6.82 (m, 2H), 5.98 (dd, *J* = 17.5, 10.5 Hz, 1H), 5.21 – 5.07 (m, 2H), 1.35 (t, *J* = 0.9 Hz, 6H). **^{13}C NMR** (76 MHz, $CDCl_3$) δ 203.6, 159.9 (d, *J* = 8.7 Hz), 156.6 (d, *J* = 8.7 Hz), 140.9, 130.9 (t, *J* = 9.6 Hz), 114.9, 111.6 (dd, *J* = 23.1, 2.5 Hz), 51.8, 23.4. **^{19}F NMR** (282 MHz, $CDCl_3$) δ -111.2 (t, *J* = 6.7 Hz, 2F). **HRMS(ESI):** calculated for $C_{12}H_{12}F_2ONa$ $[M+Na]^+$: 233.0748, found: 233.0749.

1-(2-fluorophenyl)-2,2-dimethylbut-3-en-1-one (1m)

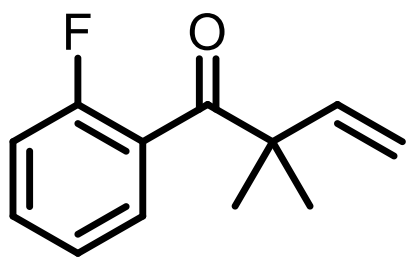

Prepared following general procedure (1). **1m** was obtained after purification by column chromatography (pentane : EtOAc 50 :1) as a colorless oil in 25% yield (480.8 mg, 2.50 mmol). **^{1}H NMR** (300 MHz, $CDCl_3$) δ 7.40 – 7.33 (m, 1H), 7.28 – 7.22 (m, 1H), 7.16 – 7.02 (m, 2H), 6.01 (ddd, *J* = 17.6, 10.3, 0.8 Hz, 1H), 5.18 (q, *J* = 0.8 Hz, 1H), 5.13 (dd, *J* = 5.6, 0.8 Hz, 1H),1.34 (d, *J* = 0.8 Hz, 6H). **^{13}C NMR** (76 MHz, $CDCl_3$) δ 206.4, 158.4 (d, *J* = 248.3 Hz), 141.9 (d, *J* = 1.3 Hz), 131.4 (d, *J* = 8.0 Hz), 128.4 (d, *J* = 4.1 Hz), 123.7 (d, *J* = 3.4 Hz), 116.2, 115.9, 114.9, 51.4, 24.2. **^{19}F NMR** (282 MHz, $CDCl_3$) δ -107.3 – -115.2 (m, 1F). **HRMS(ESI):** calculated for $C_{12}H_{13}FONa$ $[M+Na]^+$: 215.0842, found: 215.0843. These data are in accordance with those reported previously.[2]

1-(3-fluorophenyl)-2,2-dimethylbut-3-en-1-one (1n)

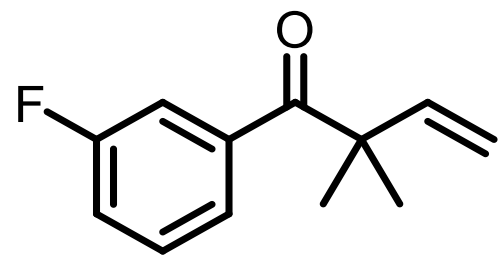

Prepared following general procedure (1). **1n** was obtained after purification by column chromatography (pentane : EtOAc 50 :1) as a colorless oil in 40% yield (768.6 mg, 4.00 mmol). **^{1}H NMR** (300 MHz, $CDCl_3$) δ 7.67 – 7.63 (m, 1H), 7.60 – 7.53 (m, 1H), 7.40 – 7.30 (m, 1H), 7.19 – 7.12 (m, 1H), 6.16 (dd, *J* = 17.5, 10.4Hz, 1H), 5.31 – 5.17 (m, 2H), 1.38 (s, 6H). **^{13}C NMR** (76 MHz, $CDCl_3$) δ 203.4 (d, *J* = 1.9 Hz), 162.3 (d, *J* = 246.8 Hz), 143.6, 139.3, 129.7 (d, *J* = 7.7 Hz), 125.1 (d, *J* = 3.1 Hz), 118.8 (d, *J* = 21.3 Hz), 116.3 (d, *J* = 22.8 Hz), 114.7, 50.4, 26.0. **^{19}F NMR** (282 MHz, $CDCl_3$) δ -112.6 – -112.7 (m, 1F). **HRMS(ESI):** calculated for $C_{12}H_{13}FONa$ $[M+Na]^+$: 215.0842, found: 215.0843. These data are in accordance with those reported previously.[2]

1-(4-fluorophenyl)-2,2-dimethylbut-3-en-1-one (1o)

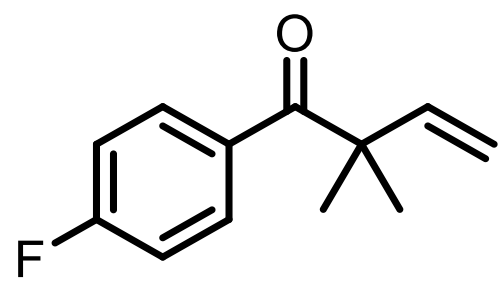

Prepared following general procedure (1). **1o** was obtained after purification by column chromatography (pentane : EtOAc 50 :1) as a colorless oil in 39% yield (750.3 mg, 3.90 mmol). **^{1}H NMR** (300 MHz, $CDCl_3$) δ 7.98 – 7.92 (m, 2H), 7.11 – 6.97 (m, 2H), 6.17 (dd, *J* = 17.6, 10.4 Hz, 1H), 5.29 – 5.16 (m, 2H), 1.38 (s, 6H). **^{13}C NMR** (76 MHz, $CDCl_3$) δ 202.8, 164.9 (d, *J* = 253.4 Hz), 144.0, 133.1 (d, *J* = 3.3 Hz), 132.3 (d, *J* = 8.9 Hz), 115.1 (d, *J* = 21.6 Hz), 114.3, 50.2, 26.2. **^{19}F NMR** (282 MHz, $CDCl_3$) δ -107.4 – -107.5 (m, 1F). **HRMS(ESI):** calculated for $C_{12}H_{13}FONa$ $[M+Na]^+$: 215.0842, found: 215.0843. These data are in accordance with those reported previously.[2]

4,4-dimethyl-1-phenylhex-5-en-3-one (1p)

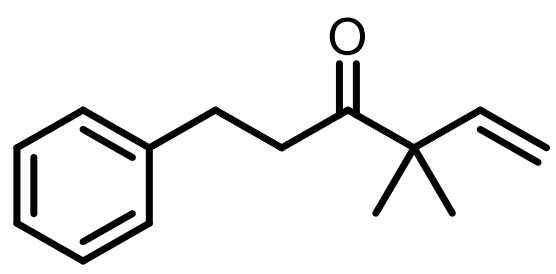

Prepared following general procedure (1). **1p** was obtained after purification by column chromatography (pentane : EtOAc 100 :1) as a pale yellow oil in 31% yield (313.1 mg, 1.55 mmol). **^{1}H NMR** (300 MHz, $CDCl_3$) δ 7.30 – 7.23 (m, 2H), 7.22 – 7.13 (m, 3H), 5.86 (dd, *J* = 17.7, 10.3 Hz, 1H), 5.17 – 5.04 (m, 2H), 2.89 – 2.81 (m, 2H), 2.80 – 2.73 (m, 2H), 1.19 (s, 6H). **^{13}C NMR** (76 MHz, $CDCl_3$) δ 212.2, 142.5, 141.6, 128.5, 128.5, 126.1, 114.5, 50.9, 39.5, 30.3, 23.6. **HRMS(ESI):** calculated for $C_{14}H_{18}ONa$ $[M+Na]^+$: 225.1250, found: 225.1251. These data are in accordance with those reported previously.[6]

1-cyclohexyl-2,2-dimethylbut-3-en-1-one (1q)

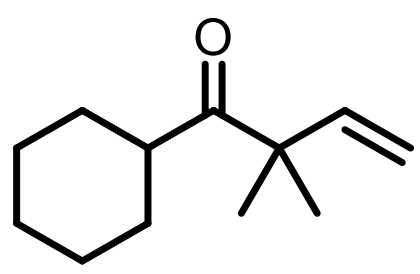

Prepared following general procedure (1). **1q** was obtained after purification by column chromatography (pentane : EtOAc 100 :1) as a pale yellow oil in 63% yield (567.3 mg, 3.15 mmol). **^{1}H NMR** (300 MHz, $CDCl_3$) δ 5.92 (dd, *J* = 17.4, 10.6 Hz, 1H), 5.21 – 5.12 (m, 2H), 2.81 – 2.71 (m, 1H), 1.77 – 1.57 (m, 5H), 1.44 – 1.22 (m, 5H), 1.21 (s, 6H). **^{13}C NMR** (76 MHz, $CDCl_3$) δ 213.6, 142.8, 114.9, 52.3, 40.0, 39.4, 27.8, 24.0, 21.8. **HRMS(ESI):** calculated for $C_{12}H_{20}ONa$ $[M+Na]^+$: 203.1406, found: 203.1407. These data are in accordance with those reported previously.[2]

3,3-dimethyltridec-1-en-4-one (1r)

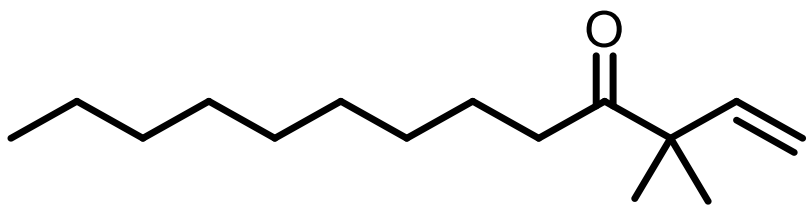

Prepared following general procedure (1). **1r** was obtained after purification by column chromatography (pentane : EtOAc 100 :1) as a yellow oil in 55% yield (1.23 g, 5.50 mmol). **^{1}H NMR** (300 MHz, $CDCl_3$) δ 5.91 (dd, *J* = 17.6, 10.6 Hz, 1H), 5.19 – 5.06 (m, 2H), 2.42 (t, *J* = 7.3Hz, 2H), 1.58 – 1.41 (m, 2H), 1.28 – 1.22 (m, 12H), 1.21 (s, 6H), 0.96 – 0.80 (m, 3H). **^{13}C NMR** (76 MHz, $CDCl_3$) δ 213.4, 142.8, 114.2, 51.0, 37.5, 32.0, 29.6, 29.41, 29.39, 24.2, 23.6, 22.8, 14.2. **HRMS(ESI):** calculated for $C_{15}H_{28}ONa$ $[M+Na]^+$: 247.2032, found: 247.2033.

2,2-dimethyl-1-(thiophen-3-yl)but-3-en-1-one (1s)

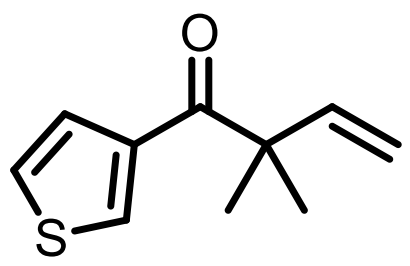

Prepared following general procedure (1). **1s** was obtained after purification by column chromatography (pentane : EtOAc 50 :1) as a pale yellow oil in 49% yield (882.1 mg, 4.90 mmol). **^{1}H NMR** (300 MHz, $CDCl_3$) δ 8.12 (dd, *J* = 2.9, 1.3 Hz, 1H), 7.56 (dd, *J* = 5.1, 1.3 Hz, 1H), 7.23 (dd, *J* = 5.1, 2.9 Hz, 1H), 6.18 (dd, *J* = 17.5, 10.6 Hz, 1H), 5.23 (dd, *J* = 17.5, 10.6 Hz, 2H), 1.39 (s, 6H). **^{13}C NMR** (76 MHz, $CDCl_3$) δ 197.9, 144.1, 140.1, 133.0, 129.0, 125.0, 114.2, 50.3, 25.6. **HRMS(ESI):** calculated for $C_{10}H_{12}OSNa$ $[M+Na]^+$: 203.0501, found: 203.0501. These data are in accordance with those reported previously.[2]

2-methyl-1,2-diphenylbut-3-en-1-one (1t)

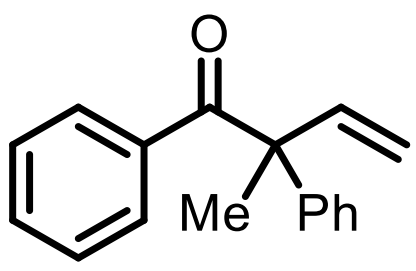

Prepared following general procedure (2). **1t** was obtained after purification by column chromatography (pentane : EtOAc 50 :1) as a pale yellow solid in 21% yield (495.6 mg, 2.10 mmol). **^{1}H NMR** (400 MHz, $CDCl_3$) δ 7.56 – 7.52 (m, 2H), 7.40 – 7.32 (m, 3H), 7.29 – 7.26 (m, 3H), 7.26 – 7.21 (m, 2H), 6.62 (dd, *J* = 17.4, 10.8 Hz, 1H), 5.30 (dd, *J* = 10.8, 0.8 Hz, 1H), 5.17 (dd, *J* = 17.4, 0.8 Hz, 1H), 1.67 (s, 3H). **^{13}C NMR** (151 MHz, $CDCl_3$) δ 201.0, 144.6, 141.1, 136.3, 132.0, 130.2, 129.2, 128.1, 127.1, 126.6, 116.7, 58.7, 26.1. **HRMS(ESI):** calculated for $C_{17}H_{16}ONa$ $[M+Na]^+$: 259.1093, found: 259.1094. These data are in accordance with those reported previously.[2]

2-ethyl-1,2-diphenylbut-3-en-1-one (1u)

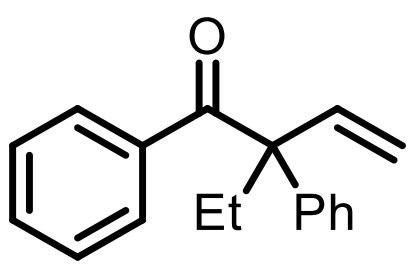

Prepared following general procedure (2). **1u** was obtained after purification by column chromatography (pentane : EtOAc 50 :1) as a colorless solid in 13% yield (325.6 mg, 1.30 mmol). **^{1}H NMR** (600 MHz, $CDCl_3$) δ 7.55 – 7.52 (m, 2H), 7.36 – 7.31 (m, 3H), 7.27 – 7.19 (m, 5H), 6.69 (dd, *J* = 17.6, 10.9 Hz, 1H), 5.33 (dd, *J* = 10.9, 0.7 Hz, 1H), 5.02 (dd, *J* = 17.6, 0.7 Hz, 1H), 2.32 – 2.15 (m, 2H), 0.71 (t, *J* = 7.4 Hz, 3H). **^{13}C NMR** (151 MHz, $CDCl_3$) δ 200.7, 142.5, 139.3, 136.5, 131.9, 130.2, 128.9, 128.0, 127.4, 127.0, 117.7, 62.0, 31.7, 8.7. **HRMS(ESI):** calculated for $C_{18}H_{18}ONa$ $[M+Na]^+$: 273.1250, found: 273.1252. These data are in accordance with those reported previously.[7]

1,2-diphenylbut-3-en-1-one (1v)

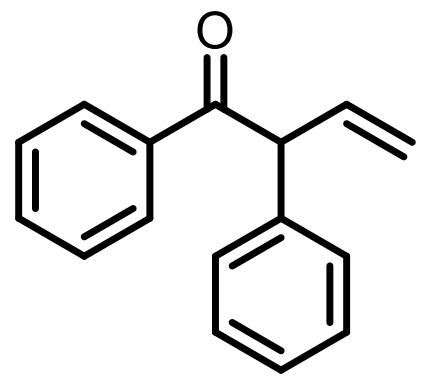

Prepared following general procedure (1). **1v** was obtained after purification by column chromatography (pentane : EtOAc 50 :1) as a colorless solid in 59% yield (2.62 g, 11.8 mmol). **^{1}H NMR** (400 MHz, $CDCl_3$) δ 7.95 – 7.90 (m, 2H), 7.48 – 7.43 (m, 1H), 7.38 – 7.33 (m, 2H), 7.30 – 7.24 (m, 4H), 7.22 – 7.16 (m, 1H), 6.32 (ddd, *J* = 17.2, 10.2, 7.7 Hz, 1H), 5.24 (dt, *J* = 7.7, 1.1 Hz, 1H), 5.18 (dt, *J* = 10.2, 1.1 Hz, 1H), 5.05 (dt, *J* = 17.2, 1.1 Hz, 1H). **^{13}C NMR** (101 MHz, $CDCl_3$) δ 198.7, 138.5, 137.3, 136.5, 133.2, 129.1, 129.0, 128.7, 128.5, 127.4, 117.3, 58.1. **HRMS(ESI):** calculated for $C_{16}H_{14}ONa$ $[M+Na]^+$: 245.0937, found: 245.0937. These data are in accordance with those reported previously.[8]

phenyl(1-vinylcyclohexyl)methanone (1w)

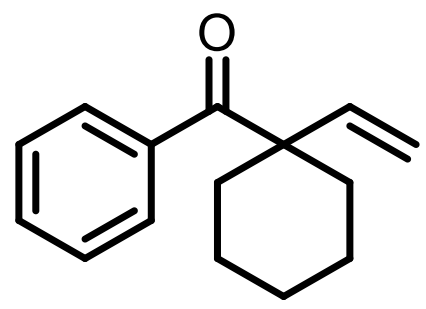

Prepared following general procedure (2). **1w** was obtained after purification by column chromatography (pentane : EtOAc 100 :1) as a colorless oil in 23% yield (492.5 mg, 2.30 mmol). **^{1}H NMR** (400 MHz, $CDCl_3$) δ 7.78 – 7.73 (m, 2H), 7.47 – 7.41 (m, 1H), 7.39 – 7.33 (m, 2H), 6.12 (dd, *J* = 17.7, 10.7 Hz, 1H), 5.26 (dd, *J* = 10.7, 0.7 Hz, 1H), 5.20 (dd, *J* = 17.7, 0.7 Hz, 1H), 2.19 – 2.13 (m, 2H), 1.66 – 1.60 (m, 2H), 1.55 – 1.41 (m, 3H), 1.40 – 1.27 (m, 3H). **^{13}C NMR** (101 MHz, $CDCl_3$) δ 205.6, 142.9, 138.4, 131.3, 128.8, 128.0, 116.2, 54.5, 35.2, 25.9, 22.9. **HRMS(ESI):** calculated for $C_{15}H_{18}ONa$ $[M+Na]^+$: 237.1250, found: 237.1251. These data are in accordance with those reported previously.[2]

4. General procedure and characterisation data of products

4.1 General procedure for the 1,2-Carbonyl Migration

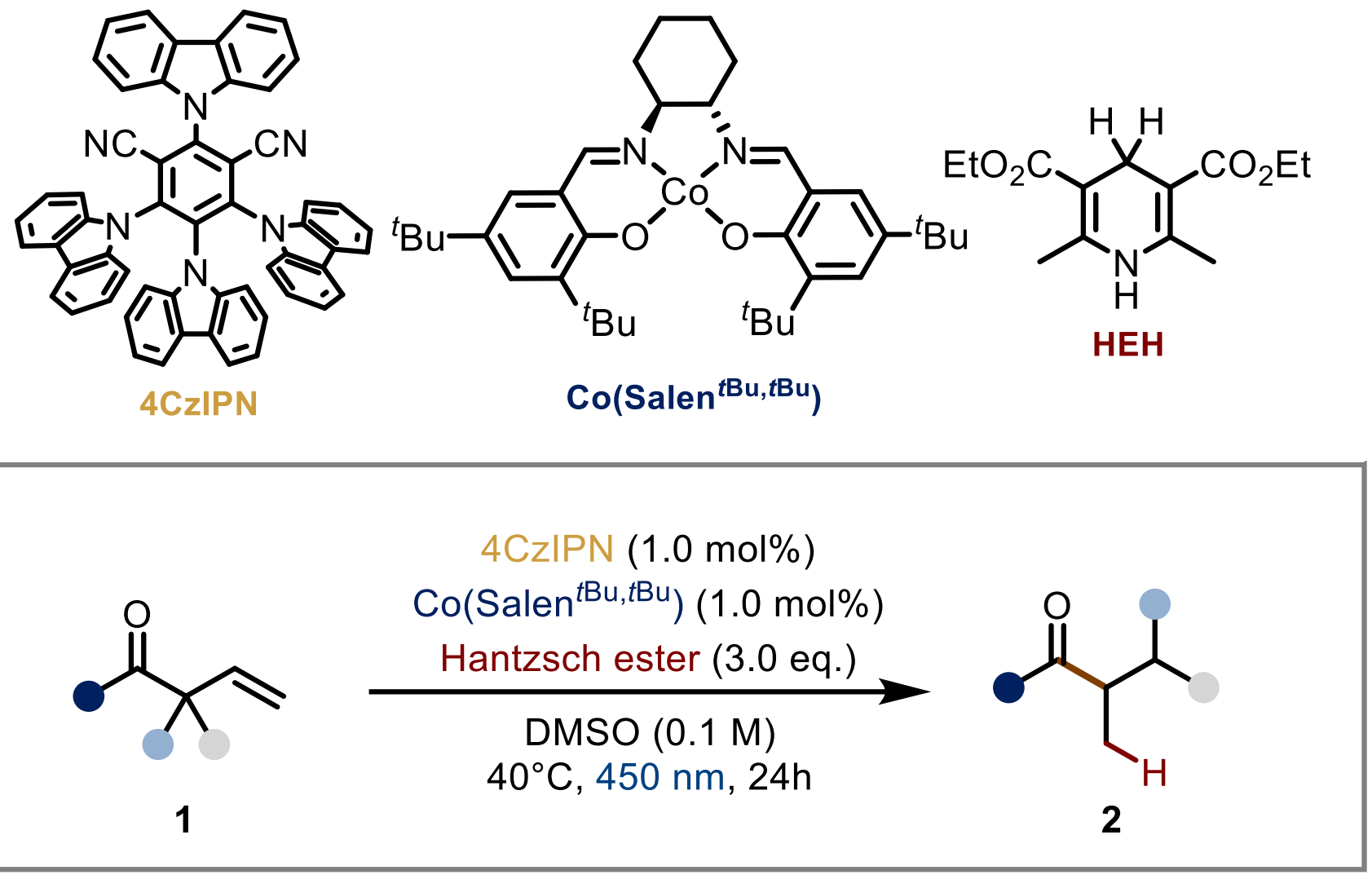

In an oven-dried 4 mL vial equipped with a magnetic stir bar were placed *β,γ*-unsaturated ketone **1** (0.10 mmol), 4CzIPN (0.001 mmol, 1.0 mol%, 0.8 mg), Co(Salen^{tBu,tBu}) (0.001 mmol, 1.0 mol%, 0.6 mg), and Hantzsch ester (0.30 mmol, 3.0 equiv, 75.9 mg). The vial was sealed with a plastic cap fitted with a rubber septum, and the reaction mixture was degassed by purging with argon for 15 min. DMSO (1.0 mL) was then added. The vial was placed in the PhotoRedOx Box (see Materials and Methods for details of the photochemical setup) and irradiated with 450 nm light for 24 h without cooling fan; under these conditions, the reaction temperature was approximately 40 °C. Upon completion, the reaction mixture was extracted with EtOAc and the organic phase was washed with water (3 ×) to remove residual DMSO. The organic layer was dried over $MgSO_4$, filtered, and concentrated under reduced pressure. Purification of the residue by silica gel column chromatography (Pentane : EtOAc) afforded the corresponding product.

4.2 Characterisation data

1-mesityl-2,3-dimethylbutan-1-one (2a)

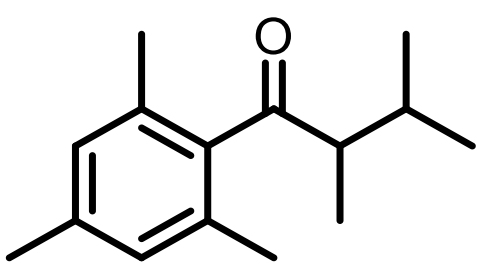

Prepared following general procedure for the 1,2-Carbonyl Migration, **2a** was obtained after purification by column chromatography (pentane : EtOAc 100 :1) as a colorless oil (19.8 mg, 91 µmol, 91%). **^{1}H NMR** (300 MHz, $CDCl_3$) δ 6.86 – 6.80 (m, 2H), 2.82 (dq, *J* = 7.2 Hz, 4.3 Hz, 1H), 2.27 (s, 3H), 2.21 (s, 6H), 2.19 -2.10 (m, 1H), 1.07 (d, *J* = 7.2 Hz, 3H), 0.99 (d, *J* = 6.8 Hz, 3H), 0.91 (d, *J* = 6.8 Hz, 3H). **^{13}C NMR** (76 MHz, $CDCl_3$) δ 213.1, 139.3, 138.4, 133.8, 128.9, 53.2, 28.0, 22.2, 21.2, 19.9, 17.6, 10.3. **HRMS(ESI):** calculated for $C_{15}H_{22}ONa$ $[M+Na]^+$: 241.1563, found: 241.1564.

1-(2,6-dimethylphenyl)-2,3-dimethylbutan-1-one (2b)

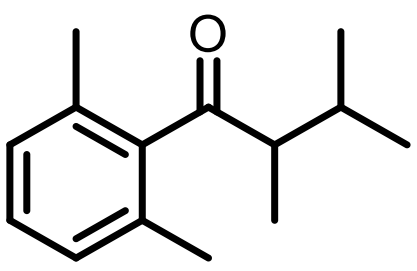

Prepared following general procedure for the 1,2-Carbonyl Migration, **2b** was obtained after purification by column chromatography (pentane : EtOAc 100 :1) as a pale yellow solid (14.7 mg, 72 µmol, 72%). **^{1}H NMR** (600 MHz, $CDCl_3$) δ 7.15 (dd, *J* = 8.0, 7.3 Hz, 1H), 7.03 – 6.99 (m, 2H), 2.83 (dq, *J* = 7.2, 4.2 Hz, 1H), 2.24 (s, 6H), 2.22 – 2.15 (m, 1H), 1.08 (d, *J* = 7.2 Hz, 3H), 1.00 (d, *J* = 6.8 Hz, 3H), 0.93 (d, *J* = 6.8 Hz, 3H). **^{13}C NMR** (151 MHz, $CDCl_3$) δ 212.9, 142.0, 133.7, 128.6, 128.2, 53.2, 27.8, 22.1, 19.9, 17.6, 10.2. **HRMS(ESI):** calculated for $C_{14}H_{20}ONa$ $[M+Na]^+$: 227.1406, found: 227.1407.

2,3-dimethyl-1-phenylbutan-1-one (2c)

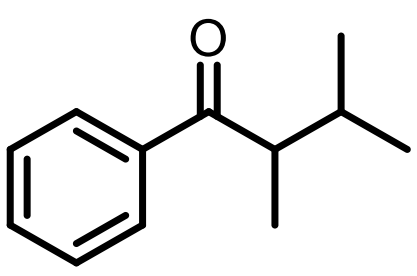

Prepared following general procedure for the 1,2-Carbonyl Migration, **2c** was obtained after purification by column chromatography (pentane : EtOAc 100 :1) as a pale yellow oil (9.7 mg, 55 µmol, 55%). **^{1}H NMR** (500 MHz, $CDCl_3$) δ 7.96 – 7.92 (m, 2H), 7.58 – 7.52 (m, 1H), 7.47 – 7.44 (m, 2H), 3.29 (*app* p, *J* = 6.8 Hz, 1H), 2.15 – 2.04 (m, 1H), 1.14 (d, *J* = 6.8 Hz, 3H), 0.95 (d, *J* = 6.8 Hz, 3H), 0.90 (d, *J* = 6.8 Hz, 3H). **^{13}C NMR** (126 MHz, $CDCl_3$) δ 204.9, 137.5, 132.9, 128.7, 128.4, 47.0, 30.9, 21.7, 18.8, 13.5. **HRMS(ESI):** calculated for $C_{12}H_{16}ONa$ $[M+Na]^+$: 199.1093, found: 199.1094.

2,3-dimethyl-1-(o-tolyl)butan-1-one (2d)

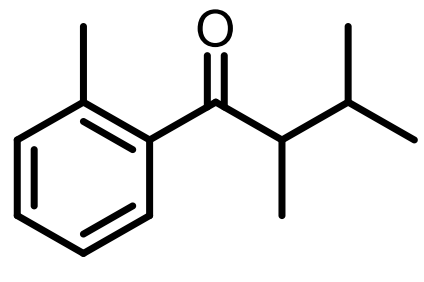

Prepared following general procedure for the 1,2-Carbonyl Migration, **2d** was obtained after purification by column chromatography (pentane : EtOAc 100 :1) as a pale yellow oil (5.3 mg, 28 µmol, 28%). **^{1}H NMR** (600 MHz, $CDCl_3$) δ 7.55 – 7.52 (m, 1H), 7.37 – 7.32 (m, 1H), 7.29 – 7.21 (m, 2H), 3.11 (*app* p, *J* = 6.8 Hz, 1H), 2.45 (s, 3H), 2.06 (dq, *J* = 13.5, 6.7 Hz, 1H), 1.11 (d, *J* = 6.8 Hz, 3H), 0.96 (d, *J* = 6.7 Hz, 3H), 0.88 (d, *J* = 6.8 Hz, 3H). **^{13}C NMR** (151 MHz, $CDCl_3$) δ 209.2, 139.3, 137.8, 131.9, 130.8, 127.9, 125.7, 50.4, 30.4, 21.8, 20.9, 18.7, 12.8. **HRMS(ESI):** calculated for $C_{13}H_{18}ONa$ $[M+Na]^+$: 213.1250, found: 213.1250.

2,3-dimethyl-1-(m-tolyl)butan-1-one (2e)

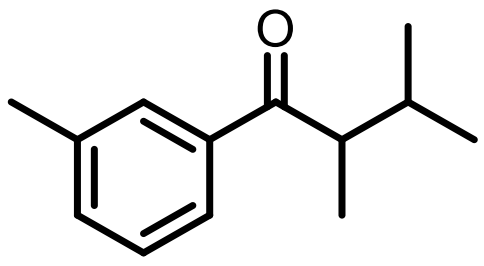

Prepared following general procedure for the 1,2-Carbonyl Migration, **2e** was obtained after purification by column chromatography (pentane : EtOAc 100 :1) as a pale yellow oil (8.6 mg, 45 µmol, 45%). **^{1}H NMR** (300 MHz, $CDCl_3$) δ 7.77 – 7.69 (m, 2H), 7.38 – 7.31 (m, 2H), 3.27 (*app* p, *J* = 6.9 Hz, 1H), 2.42 (s, 3H), 2.08 (dq, *J* = 13.6, 6.9 Hz, 1H), 1.13 (d, *J* = 6.9 Hz, 3H), 0.94 (d, *J* = 6.7 Hz, 3H), 0.90 (d, *J* = 6.7 Hz, 3H). **^{13}C NMR** (151 MHz, $CDCl_3$) δ 205.2, 138.5, 137.6, 133.6, 128.9, 128.4, 125.6, 47.1, 30.9, 21.8, 21.6, 18.8, 13.7. **HRMS(ESI):** calculated for $C_{13}H_{18}ONa$ $[M+Na]^+$: 213.1250, found: 213.1250.

2,3-dimethyl-1-(p-tolyl)butan-1-one (2f)

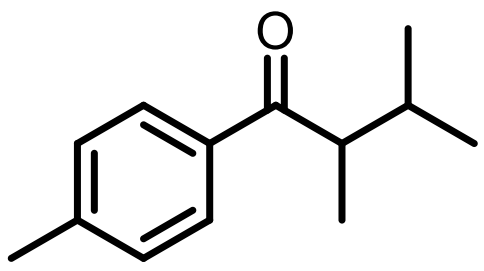

Prepared following general procedure for the 1,2-Carbonyl Migration, **2f** was obtained after purification by column chromatography (pentane : EtOAc 100 :1) as a pale yellow oil (9.9 mg, 52 µmol, 52%). **^{1}H NMR** (600 MHz, $CDCl_3$) δ 7.85 (d, *J* = 8.2 Hz, 2H), 7.27 – 7.26 (m, 1H), 7.26 – 7.24 (m, 1H), 3.25 (*app* p, *J* = 6.9 Hz, 1H), 2.41 (s, 3H), 2.08 (*app* h, *J* = 6.8 Hz, 1H), 1.12 (d, *J* = 6.9 Hz, 3H), 0.93 (d, *J* = 6.8 Hz, 3H), 0.90 (d, *J* = 6.8 Hz, 3H). **^{13}C NMR** (151 MHz, $CDCl_3$) δ 204.6, 143.6, 134.9, 129.4, 128.5, 46.9, 30.9, 21.8, 21.7, 18.9, 13.7. **HRMS(ESI):** calculated for $C_{13}H_{18}ONa$ $[M+Na]^+$: 213.1250, found: 213.1250.

1-(4-(tert-butyl)phenyl)-2,3-dimethylbutan-1-one (2g)

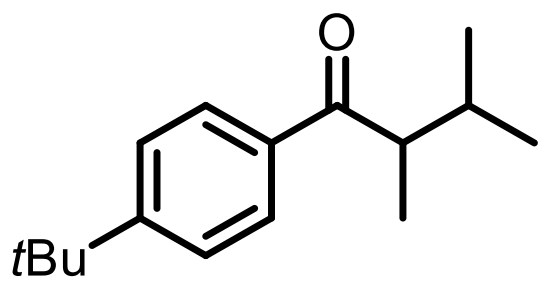

Prepared following general procedure for the 1,2-Carbonyl Migration, **2g** was obtained after purification by column chromatography (pentane : EtOAc 100 :1) as a colorless oil (16.2 mg, 70 µmol, 70%). **^{1}H NMR** (600 MHz, $CDCl_3$) δ 7.91 – 7.88 (m, 2H), 7.49 – 7.46 (m, 2H), 3.27 (*app* p, *J* = 6.9 Hz, 1H), 2.09 (dq, *J* = 13.6, 6.8 Hz, 1H), 1.34 (s, 9H), 1.13 (d, *J* = 6.9 Hz, 3H), 0.94 (d, *J* = 6.8 Hz, 3H), 0.90 (d, *J* = 6.8 Hz, 3H). **^{13}C NMR** (151 MHz, $CDCl_3$) δ 204.5, 156.5, 134.8, 128.3, 125.7, 46.9, 35.2, 31.3, 30.9, 21.8, 18.8, 13.7. **HRMS(ESI):** calculated for $C_{16}H_{24}ONa$ $[M+Na]^+$: 255.1719, found: 255.1719.

1-([1,1'-biphenyl]-2-yl)-2,3-dimethylbutan-1-one (2h)

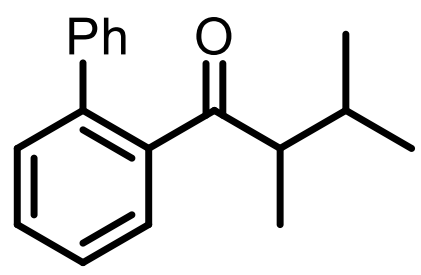

Prepared following general procedure for the 1,2-Carbonyl Migration, **2h** was obtained after purification by column chromatography (pentane : EtOAc 100 :1) as a colorless oil (12.9 mg, 51 µmol, 51%). **^{1}H NMR** (400 MHz, $CDCl_3$) δ 7.51 – 7.47 (m, 1H), 7.46 – 7.35 (m, 6H), 7.36 – 7.29 (m, 2H), 2.26 (qd, *J* = 6.9, 4.4 Hz, 1H), 1.74 (pd,

J = 6.9, 4.4 Hz, 1H), 0.77 (d, *J* = 6.8 Hz, 3H), 0.68 (d, *J* = 6.8 Hz, 3H), 0.65 (d, *J* = 6.8 Hz, 3H). **^{13}C NMR** (126 MHz, $CDCl_3$) δ 211.3, 141.4, 141.0, 140.0, 130.3, 130.2, 129.0, 128.7, 128.3, 127.9, 127.5, 51.0, 29.7, 21.3, 17.7, 10.8. **HRMS(ESI):** calculated for $C_{18}H_{20}ONa$ $[M+Na]^+$: 275.1406, found: 275.1408.

1-(4-methoxyphenyl)-2,3-dimethylbutan-1-one (2i)

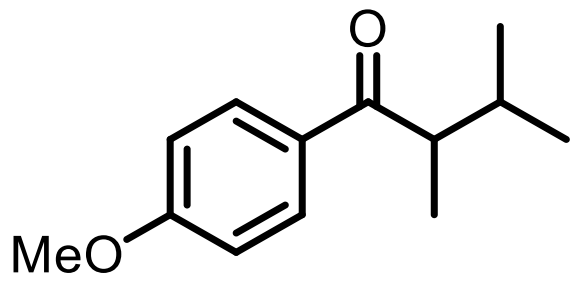

Prepared following general procedure for the 1,2-Carbonyl Migration, **2i** was obtained after purification by column chromatography (pentane : EtOAc 20 :1) as a pale yellow oil (11.5 mg, 56 µmol, 56%). **^{1}H NMR** (600 MHz, $CDCl_3$) δ 7.97 – 7.92 (m, 2H), 6.97 – 6.91 (m, 2H), 3.87 (s, 3H), 3.23 (*app* p, *J* = 6.9 Hz, 1H), 2.07 (dq, *J* = 13.6, 6.8 Hz, 1H), 1.12 (d, *J* = 6.9 Hz, 3H), 0.93 (d, *J* = 6.8 Hz, 3H), 0.90 (d, *J* = 6.8 Hz, 3H). **^{13}C NMR** (151 MHz, $CDCl_3$) δ 203.5, 163.4, 130.6, 130.4, 113.9, 55.6, 46.6, 31.0, 21.8, 19.0, 13.9. **HRMS(ESI):** calculated for $C_{13}H_{18}O_2Na$ $[M+Na]^+$: 229.1199, found: 229.1200.

2,3-dimethyl-1-(3-phenoxyphenyl)butan-1-one (2j)

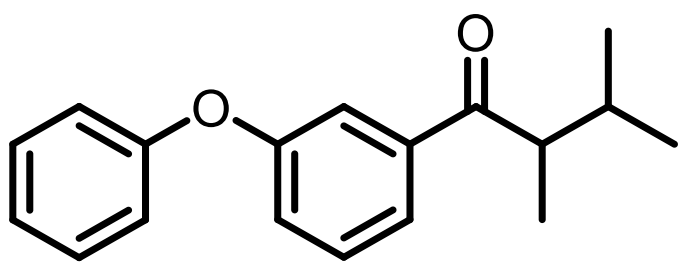

Prepared following general procedure for the 1,2-Carbonyl Migration, **2j** was obtained after purification by column chromatography (pentane : EtOAc 20 :1) as a pale yellow oil (17.4 mg, 65 µmol, 65%). **^{1}H NMR** (300 MHz, $CDCl_3$) δ 7.68 -7.64 (m, 1H), 7.58 – 7.56 (m, 1H), 7.45 – 7.32 (m, 3H), 7.21 – 7.10 (m, 2H), 7.05 – 6.99 (m, 2H), 3.21 (*app* p, *J* = 6.8 Hz, 1H), 2.14 – 1.99 (m, 1H), 1.12 (d, *J* = 6.8 Hz, 3H), 0.93 (d, *J* = 6.8 Hz, 3H), 0.88 (d, *J* = 6.8 Hz, 3H). **^{13}C NMR** (151 MHz, $CDCl_3$) δ 204.2, 157.9, 156.8, 139.2, 130.1, 123.9, 123.0, 123.0, 119.3, 118.3, 47.2, 30.8, 21.7, 18.7, 13.4. **HRMS(ESI):** calculated for $C_{18}H_{20}O_2Na$ $[M+Na]^+$: 291.1355, found: 291.1356.

2,3-dimethyl-1-(2,4,6-trifluorophenyl)butan-1-one (2k)

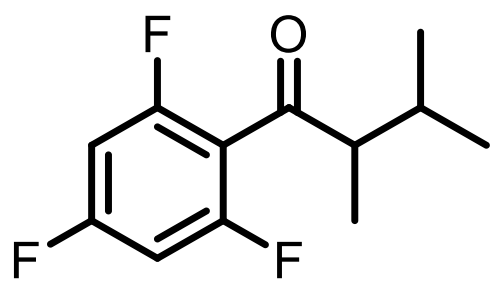

Prepared following general procedure for the 1,2-Carbonyl Migration, **2k** was obtained after purification by column chromatography (pentane : EtOAc 50 :1) as a pale yellow oil (8.3 mg, 36 µmol, 36%). **^{1}H NMR** (300 MHz, $CDCl_3$) δ 6.75 – 6.64 (m, 2H), 2.89 (ddt, *J* = 6.9, 5.7, 1.2 Hz, 1H), 2.17 – 2.01 (m, 1H), 1.12 (d, *J* = 6.9 Hz, 3H), 0.98 (d, *J* = 6.8 Hz, 3H), 0.87 (d, *J* = 6.8 Hz, 3H). **^{13}C NMR** (151 MHz, $CDCl_3$) δ 200.9, 163.6 (dt, *J* = 253.6, 15.4 Hz), 160.5 (ddd, *J* = 253.3, 15.0, 10.6 Hz), 115.8 (td, *J* = 20.6, 4.8 Hz), 101.6 – 100.5 (m), 53.8, 30.1, 21.4, 18.2, 11.3. **^{19}F NMR** (282 MHz, $CDCl_3$) δ -104.2 (t, *J* = 8.2 Hz, 1F), -109.7 (d, *J* = 7.8 Hz, 2F). **HRMS(ESI):** calculated for $C_{12}H_{13}F_3ONa$ $[M+Na]^+$: 253.0810, found: 253.0812.

1-(2,6-difluorophenyl)-2,3-dimethylbutan-1-one (2l)

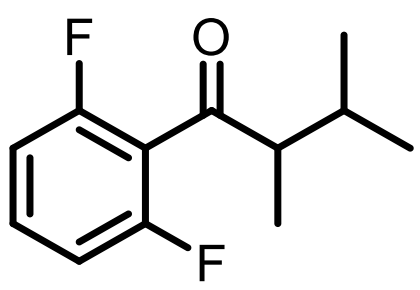

Prepared following general procedure for the 1,2-Carbonyl Migration, **2l** was obtained after purification by column chromatography (pentane : EtOAc 50 :1) as a pale yellow oil (7.2 mg, 34 µmol, 34%). **^{1}H NMR** (600 MHz, $CDCl_3$) δ 7.35 (tt, *J* = 8.4, 6.3 Hz, 1H), 6.98 – 6.89 (m, 2H), 2.96 – 2.89 (m, 1H), 2.14 – 2.07 (m, 1H), 1.12 (d, *J* = 6.8 Hz, 3H), 0.99 (d, *J* = 6.8 Hz, 3H), 0.87 (d, *J* = 6.8 Hz, 3H). **^{13}C NMR** (151 MHz, $CDCl_3$) δ 202.1, 159.8 (dd, *J* = 251.9, 7.9 Hz), 131.8 (t, *J* = 10.2 Hz), 119.2 (t, *J* = 20.6 Hz), 112.1 (dd, *J* = 21.5, 4.5 Hz), 53.8,

29.9, 21.4, 18.2, 11.1. **^{19}F NMR** (565 MHz, $CDCl_3$) δ -113.2 (t, *J* = 7.3 Hz, 2F). **HRMS(ESI):** calculated for $C_{12}H_{14}F_2ONa$ $[M+Na]^+$: 235.0905, found: 235.0905.

1-(2-fluorophenyl)-2,3-dimethylbutan-1-one (2m)

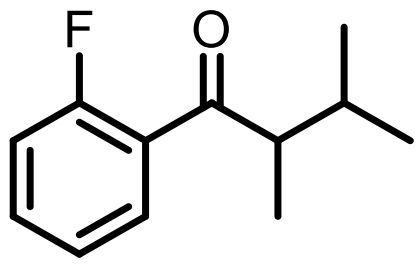

Prepared following general procedure for the 1,2-Carbonyl Migration, **2m** was obtained after purification by column chromatography (pentane : EtOAc 50 :1) as a pale yellow oil (7.4 mg, 38 µmol, 38%). **^{1}H NMR** (300 MHz, $CDCl_3$) δ 7.73 (td, *J* = 7.6, 1.9 Hz, 1H), 7.52 – 7.44 (m, 1H), 7.25 – 7.18 (m, 1H), 7.11 (ddd, *J* = 11.2, 8.4, 1.1 Hz, 1H), 3.20 (qdd, *J* = 6.8, 6.1, 1.1 Hz, 1H), 2.16 – 2.02 (m, 1H), 1.13 (dd, *J* = 6.8, 0.9 Hz, 3H), 0.97 (d, *J* = 6.8 Hz, 3H), 0.84 (d, *J* = 6.8 Hz, 3H). **^{13}C NMR** (151 MHz, $CDCl_3$) δ 203.9 (d, *J* = 4.0 Hz), 161.2 (d, *J* = 253.0 Hz), 133.9 (d, *J* = 8.9 Hz), 130.8 (d, *J* = 3.0 Hz), 127.2 (d, *J* = 13.3 Hz), 124.6 (d, *J* = 3.4 Hz), 116.7 (d, *J* = 23.9 Hz), 51.7 (d, *J* = 6.0 Hz), 30.3, 21.7, 18.3, 12.1. **^{19}F NMR** (565 MHz, $CDCl_3$) δ -112.0 (dt, *J* = 11.9, 6.1 Hz, 1F). **HRMS(ESI):** calculated for $C_{12}H_{15}FONa$ $[M+Na]^+$: 217.0999, found: 217.1000.

1-(3-fluorophenyl)-2,3-dimethylbutan-1-one (2n)

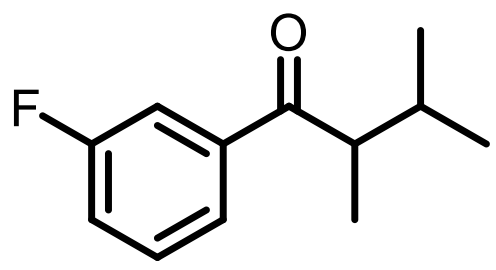

Prepared following general procedure for the 1,2-Carbonyl Migration, **2n** was obtained after purification by column chromatography (pentane : EtOAc 50 :1) as a pale yellow oil (6.6 mg, 34 µmol, 34%). **^{1}H NMR** (300 MHz, $CDCl_3$) δ 7.73 – 7.68 (m, 1H), 7.64 – 7.59 (m, 1H), 7.48 – 7.40 (m, 1H), 7.29 – 7.21 (m, 1H), 3.22 (*app* p, *J* = 6.8 Hz, 1H), 2.08 (h, *J* = 6.8 Hz, 1H), 1.14 (d, *J* = 6.8 Hz, 3H), 0.95 (d, *J* = 6.8 Hz, 3H), 0.90 (d, *J* = 6.8 Hz, 3H). **^{13}C NMR** (151 MHz, $CDCl_3$) δ 203.6 (d, *J* = 2.0 Hz), 163.1 (d, *J* = 247.7 Hz), 130.4 (d, *J* = 7.6 Hz), 124.0 (d, *J* = 3.0 Hz), 119.9 (d, *J* = 21.5 Hz), 115.2 (d, *J* = 22.2 Hz), 47.3, 30.8, 21.7, 18.7, 13.4. **^{19}F NMR** (565 MHz, $CDCl_3$) δ -111.9 – -112.0 (m, 1F). **HRMS(ESI):** calculated for $C_{12}H_{15}FONa$ $[M+Na]^+$: 217.0999, found: 217.1000.

1-(4-fluorophenyl)-2,3-dimethylbutan-1-one (2o)

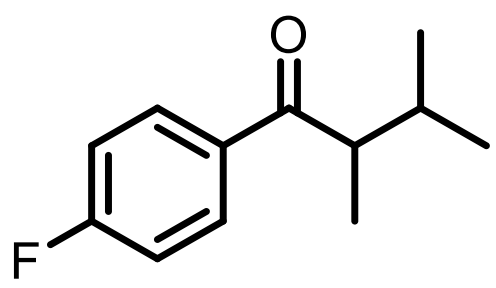

Prepared following general procedure for the 1,2-Carbonyl Migration, **2o** was obtained after purification by column chromatography (pentane : EtOAc 50 :1) as a pale yellow oil (6.0 mg, 31 µmol, 31%). **^{1}H NMR** (500 MHz, $CDCl_3$) δ 8.02 – 7.93 (m, 2H), 7.15 – 7.10 (m, 2H), 3.23 (*app* p, *J* = 6.8 Hz, 1H), 2.13 – 2.00 (m, 1H), 1.13 (d, *J* = 6.8 Hz, 3H), 0.93 (d, *J* = 6.8 Hz, 3H), 0.90 (d, *J* = 6.8 Hz, 3H). **^{13}C NMR** (126 MHz, $CDCl_3$) δ 203.3, 165.7 (d, *J* = 254.2 Hz), 133.8 (d, *J* = 3.0 Hz), 131.0 (d, *J* = 9.1 Hz), 115.8 (d, *J* = 21.9 Hz), 47.0, 30.9, 21.7, 18.8. **^{19}F NMR** (471 MHz, $CDCl_3$) δ -106.4 – -106.5 (m, 1F). **HRMS(ESI):** calculated for $C_{12}H_{15}FONa$ $[M+Na]^+$: 217.0999, found: 217.0999.

4,5-dimethyl-1-phenylhexan-3-one (2p)

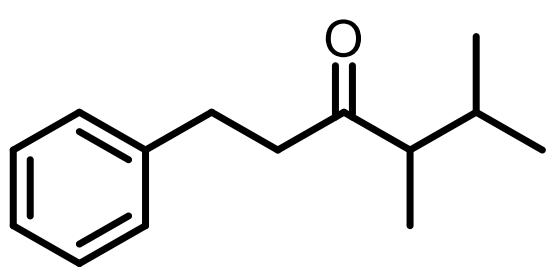

Prepared following general procedure for the 1,2-Carbonyl Migration, **2p** was obtained after purification by column chromatography (pentane : EtOAc 50 :1) as a pale yellow oil (16.5 mg, 81 µmol, 81%). **^{1}H NMR** (300 MHz, $CDCl_3$) δ 7.31 – 7.27 (m, 1H), 7.26 – 7.24 (m, 1H), 7.21 – 7.19 (m, 2H), 7.19 – 7.15 (m, 1H), 2.93 – 2.84 (m, 2H), 2.79 – 2.71 (m, 2H), 2.29 (*app* p, *J* = 7.0 Hz, 1H), 1.92 (dq, *J* = 13.7, 6.8 Hz, 1H), 0.98

(d, J = 7.0 Hz, 3H), 0.87 (d, J = 6.8 Hz, 3H), 0.83 (d, J = 6.8 Hz, 3H). **^{13}C NMR** (151 MHz, $CDCl_3$) δ 214.0, 141.5, 128.6, 128.5, 126.2, 53.2, 43.7, 30.3, 29.8, 21.5, 12.9. **HRMS(ESI):** calculated for $C_{14}H_{20}ONa$ $[M+Na]^+$: 227.1406, found: 227.1407.

1-cyclohexyl-2,3-dimethylbutan-1-one (2q)

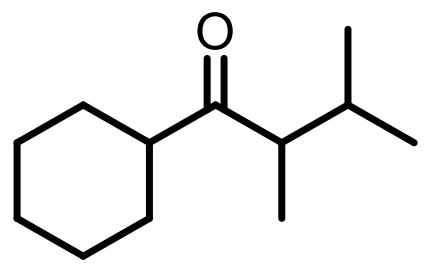

Prepared following general procedure for the 1,2-Carbonyl Migration, **2q** was obtained after purification by column chromatography (pentane : EtOAc 50 :1) as a pale yellow oil (15.1 mg, 83 µmol, 83%). **^{1}H NMR** (600 MHz, $CDCl_3$) δ 2.46 – 2.38 (m, 2H), 1.90 (dp, J = 7.0, 6.7 Hz, 1H), 1.82 – 1.72 (m, 4H), 1.37 – 1.12 (m, 6H), 0.97 (d, J = 7.0 Hz, 3H), 0.86 (d, J = 6.7 Hz, 3H), 0.84 (d, J = 6.7 Hz, 3H). **^{13}C NMR** (151 MHz, $CDCl_3$) δ 218.1, 51.3, 50.8, 30.2, 28.9, 28.2, 26.0, 25.8, 21.8, 19.1, 13.7. **HRMS(ESI):** calculated for $C_{12}H_{22}ONa$ $[M+Na]^+$: 205.1563, found: 205.1563.

2,3-dimethyltridecan-4-one (2r)

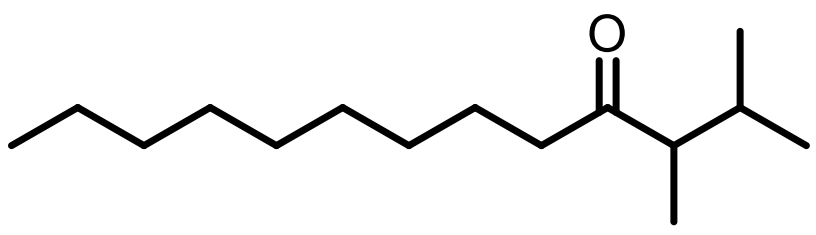

Prepared following general procedure for the 1,2-Carbonyl Migration with an addition of pyridine (16 µl, 0.2 mmol, 2 eq.), **2r** was obtained after purification by column chromatography (pentane : EtOAc 100 :1) as a pale yellow oil (20.1 mg, 89 µmol, 89%). **^{1}H NMR** (600 MHz, $CDCl_3$) δ 2.44 – 2.38 (m, 2H), 2.29 (p, J = 6.9 Hz, 1H), 1.93 (h, J = 6.7 Hz, 1H), 1.58 – 1.51 (m, 2H), 1.34 – 1.19 (m, 12H), 1.00 (d, J = 6.9 Hz, 3H), 0.90 – 0.84 (m, 9H). **^{13}C NMR** (151 MHz, $CDCl_3$) δ 215.4, 53.1, 42.1, 32.0, 30.3, 29.6, 29.5, 29.4, 24.0, 23.8, 22.8, 21.6, 19.0, 14.3, 9.2. **HRMS(ESI):** calculated for $C_{15}H_{30}ONa$ $[M+Na]^+$: 249.2189, found: 249.2191.

2,3-dimethyl-1-(thiophen-3-yl)butan-1-one (2s)

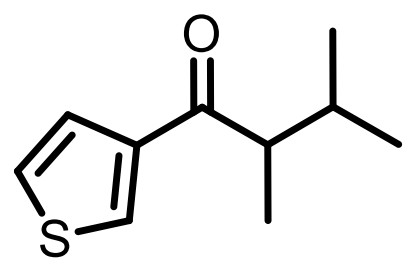

Prepared following general procedure for the 1,2-Carbonyl Migration with an addition of pyridine (16 µl, 0.2 mmol, 2 eq.), **2s** was obtained after purification by column chromatography (pentane : EtOAc 50 :1) as a pale yellow oil (5.6 mg, 31 µmol, 31%). **^{1}H NMR** (600 MHz, $CDCl_3$) δ 8.03 (dd, J = 2.9, 1.3 Hz, 1H), 7.55 (dd, J = 5.1, 1.3 Hz, 1H), 7.31 (dd, J = 5.1, 2.9 Hz, 1H), 3.04 (*app* p, J = 7.0 Hz, 1H), 2.07 (dq, J = 13.6, 6.8 Hz, 1H), 1.14 (d, J = 7.0 Hz, 3H), 0.94 (d, J = 6.8 Hz, 3H), 0.92 (d, J = 6.8 Hz, 3H). **^{13}C NMR** (151 MHz, $CDCl_3$) δ 199.2, 142.8, 131.9, 127.5, 126.4, 49.3, 31.0, 21.7, 19.1, 13.9. **HRMS(ESI):** calculated for $C_{10}H_{14}OSNa$ $[M+Na]^+$: 205.0657, found: 205.0658.

2-methyl-1,3-diphenylbutan-1-one (2t)

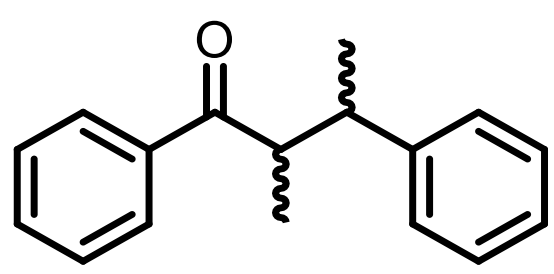

Prepared following general procedure for the 1,2-Carbonyl Migration, **2t** was obtained after purification by column chromatography (pentane : EtOAc 50 :1) as a colorless oil (10.7 mg, 45 µmol, 45%). ***Major anti product***: **^{1}H NMR** (600 MHz, $CDCl_3$) δ 8.03 – 8.01 (m, 2H), 7.60 – 7.57 (m, 1H), 7.51 – 7.48 (m, 2H), 7.42 – 7.30 (m, 2H), 7.26 – 7.20 (m, 3H), 3.69 – 3.64 (m, 1H), 3.20 – 3.15 (m, 1H), 1.21 (d, J = 6.9 Hz, 3H), 0.94 (d, J = 6.9 Hz, 3H). **^{13}C NMR** (151 MHz, $CDCl_3$) δ 204.9, 145.2, 137.6, 133.2, 128.8, 128.6, 128.4, 127.9, 126.6, 47.4, 43.4, 21.1, 17.5. ***Minor syn product:*** **^{1}H NMR** (600 MHz, $CDCl_3$) δ 7.83 – 7.77

(m, 2H), 7.51 – 7.48 (m, 1H), 7.42 – 7.30 (m, 2H), 7.26 – 7.20 (m, 3H), 7.13 – 7.07 (m, 2H), 3.76 – 3.71 (m, 1H), 3.30 – 3.24 (m, 1H), 1.29 (d, *J* = 7.0 Hz, 3H), 1.21 (d, *J* = 6.9 Hz, 3H). **^{13}C NMR** (151 MHz, $CDCl_3$) δ 204.2, 145.8, 137.1, 132.8, 128.6, 128.4, 128.2, 127.5, 126.3, 47.5, 41.7, 17.4, 14.3. **HRMS(ESI):** calculated for $C_{17}H_{18}ONa$ $[M+Na]^+$: 261.1250, found: 261.1251. These data are in accordance with those reported previously.[9]

2-methyl-1,3-diphenylpentan-1-one (2u)

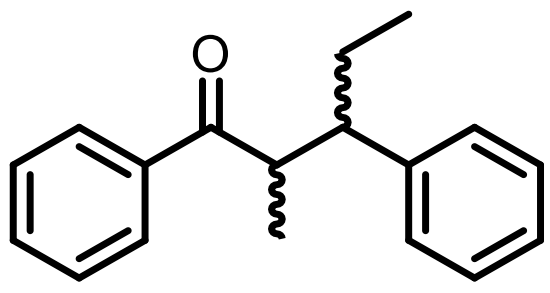

Prepared following general procedure for the 1,2-Carbonyl Migration, **2u** was obtained after purification by column chromatography (pentane : EtOAc 50 :1) as a colorless oil (7.6 mg, 30 µmol, 30%). ***Major anti product:*** **^{1}H NMR** (400 MHz, $CDCl_3$) δ 8.06 – 8.01 (m, 2H), 7.61 – 7.56 (m, 1H), 7.52 – 7.44 (m, 2H), 7.42 – 7.29 (m, 2H), 7.25 – 7.18 (m, 3H), 3.79 – 3.68 (m, 1H), 2.99 – 2.91 (m, 1H), 1.71 – 1.45 (m, 2H), 0.91 (d, *J* = 6.9 Hz, 3H), 0.66 (t, *J* = 7.3 Hz, 3H). **^{13}C NMR** (151 MHz, $CDCl_3$) δ 205.1, 142.8, 137.6, 133.2, 128.9, 128.7, 128.5, 128.4, 126.6, 51.1, 46.5, 28.0, 17.4, 12.5. ***Minor syn product***: **^{1}H NMR** (400 MHz, $CDCl_3$) δ 7.79 – 7.76 (m, 2H), 7.49 – 7.45 (m, 1H), 7.40 – 7.30 (m, 2H), 7.20 – 7.14 (m, 4H), 7.10 – 7.06 (m, 1H), 3.79 – 3.68 (m, 1H), 2.99 – 2.91 (m, 1H), 1.90 – 1.84 (m, 1H), 1.69 – 1.58 (m, 1H), 1.26 (d, *J* = 6.9 Hz, 3H), 0.71 (t, *J* = 7.3 Hz, 3H). **^{13}C NMR** (151 MHz, $CDCl_3$) δ 204.3, 143.5, 137.3, 132.7, 128.6, 128.4, 128.3, 128.2, 126.3, 49.7, 46.8, 24.2, 15.1, 12.0. **HRMS(ESI):** calculated for $C_{18}H_{20}ONa$ $[M+Na]^+$: 275.1406, found: 275.1409. These data are in accordance with those reported previously.[10]

2-methyl-1,3-diphenylpropan-1-one (2v)

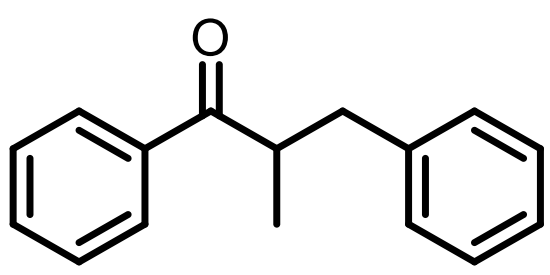

Prepared following general procedure for the 1,2-Carbonyl Migration, **2v** was obtained after purification by column chromatography (pentane : EtOAc 200 :1) as a colorless oil (3.6 mg, 16 µmol, 16%). **^{1}H NMR** (300 MHz, $CDCl_3$) δ 7.96 – 7.89 (m, 2H), 7.58 – 7.51 (m, 1H), 7.48 – 7.40 (m, 2H), 7.30 – 7.26 (m, 1H), 7.25 – 7.14 (m, 4H), 3.78 – 3.72 (m, 1H), 3.17 (dd, *J* = 13.6, 6.3 Hz, 1H), 2.69 (dd, *J* = 13.6, 8.0 Hz, 1H), 1.20 (d, *J* = 6.9 Hz, 3H). **^{13}C NMR** (151 MHz, $CDCl_3$) δ 203.9, 140.1, 136.6, 133.1, 129.2, 128.8, 128.5, 126.3, 42.9, 39.5, 17.5. **HRMS(ESI):** calculated for $C_{16}H_{16}ONa$ $[M+Na]^+$: 247.1093, found: 247.1094. These data are in accordance with those reported previously.[11]

2-cyclohexyl-1-phenylpropan-1-one (2w) - *Minor product*

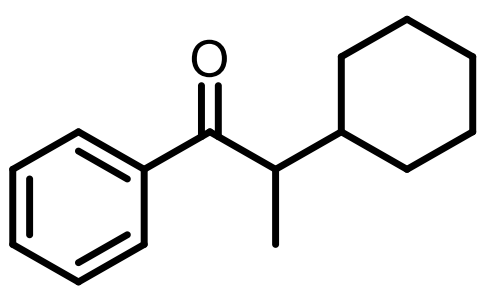

Prepared following general procedure for the 1,2-Carbonyl Migration, **2w and 2w'** were obtained after purification by column chromatography (pentane : EtOAc 100 :1) as a colorless oil (13.0 mg, 60 µmol, 60%). **^{1}H NMR** (600 MHz, $CDCl_3$) δ 7.99 – 7.93 (m, 2H), 7.57 – 7.50 (m, 1H), 7.48 – 7.40 (m, 2H), 3.32 (*app* p, *J* = 6.8 Hz, 1H), 1.77 – 1.71 (m, 3H), 1.68 – 1.46 (m, 4H), 1.27 – 1.16 (m, 2H), 1.14 (d, *J* = 6.9 Hz, 3H), 1.11 – 0.80 (m, 2H). **^{13}C NMR** (151 MHz, $CDCl_3$) δ 205.2, 137.6, 132.9, 128.7, 128.4, 46.2, 40.9, 32.2, 29.4, 26.6, 26.6, 26.5, 14.1. These data are in accordance with those reported previously.[12]

2-(cyclohex-1-en-1-yl)-1-phenylpropan-1-one (2w') - *Major product*

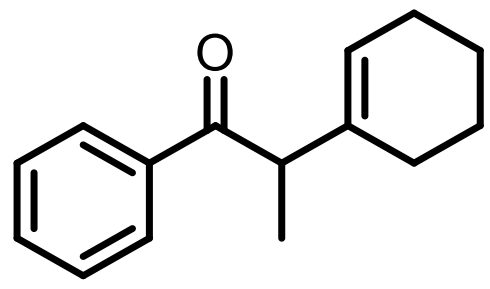

^{1}**H NMR** (600 MHz, $CDCl_3$) δ 7.98 – 7.93 (m, 2H), 7.57 – 7.50 (m, 1H), 7.48 – 7.40 (m, 2H), 5.59 – 5.57 (m, 1H), 3.99 (q, *J* = 6.9 Hz, 1H), 2.09 – 1.85 (m, 4H), 1.67 – 1.53 (m, 2H), 1.53 – 1.46 (m, 2H), 1.29 (d, *J* = 6.8 Hz, 3H). ^{13}**C NMR** (151 MHz, $CDCl_3$) δ 201.5, 137.9, 137.1, 132.7, 128.5, 128.5, 124.9, 49.4, 26.6, 25.5, 23.1, 22.3, 16.2. These data are in accordance with those reported previously.[3]

2,3,3,7-tetramethyl-2,3-dihydro-1H-inden-1-one (2d'') – *Cyclic byproduct*

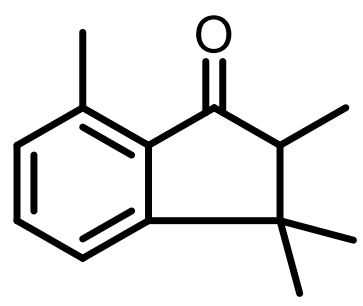

Prepared following general procedure for the 1,2-Carbonyl Migration, **2d''** was isolated as cyclic byproduct after purification by column chromatography (pentane : EtOAc 100 :1) as a colorless oil (8.4 mg, 45 µmol, 45%). ^{1}**H NMR** (400 MHz, $CDCl_3$) δ 7.44 (t, *J* = 7.5 Hz, 1H), 7.32 (dt, *J* = 7.7, 0.9 Hz, 1H), 7.09 (dp, *J* = 7.5, 0.9 Hz, 1H), 2.63 (s, 3H), 2.44 (q, *J* = 7.3 Hz, 1H), 1.43 (s, 3H), 1.19 (d, *J* = 7.3 Hz, 3H), 1.12 (s, 3H). ^{13}**C NMR** (101 MHz, $CDCl_3$) δ 209.0, 163.6, 138.5, 134.0, 132.2, 129.3, 120.7, 55.1, 41.3, 27.9, 27.2, 18.5, 9.7. **HRMS(ESI):** calculated for $C_{13}H_{16}ONa$ $[M+Na]^+$: 211.1093, found: 211.1094.

4.3 1 mmol-Scale examples of 1,2-Carbonyl Migration

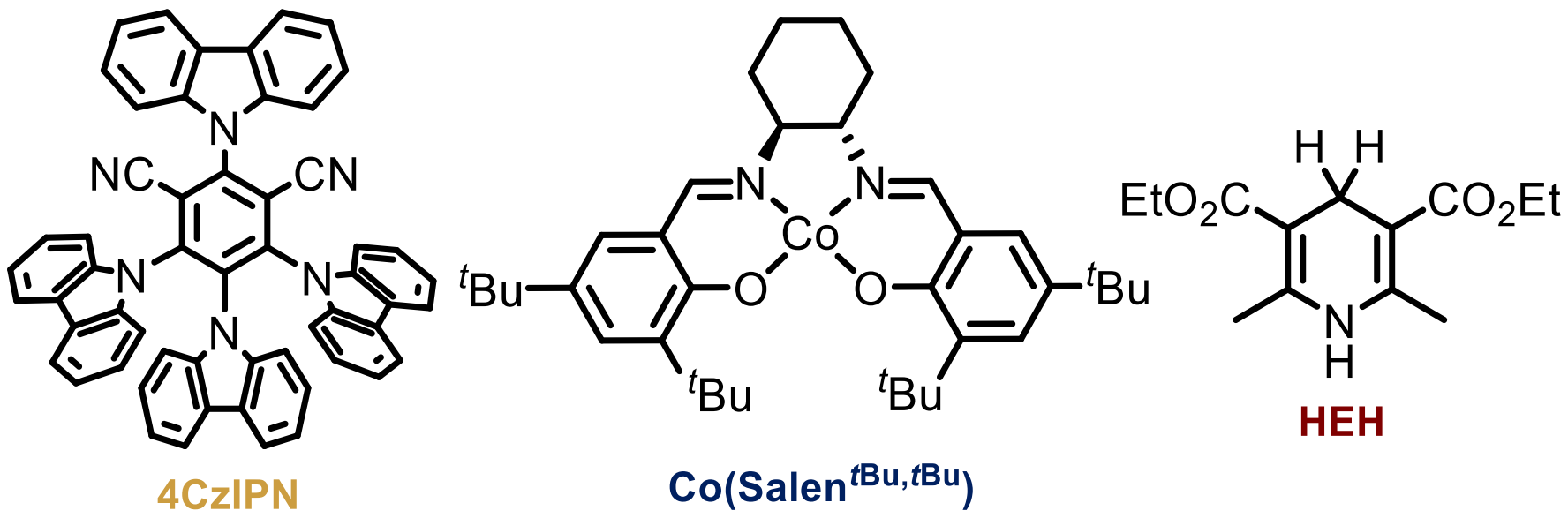

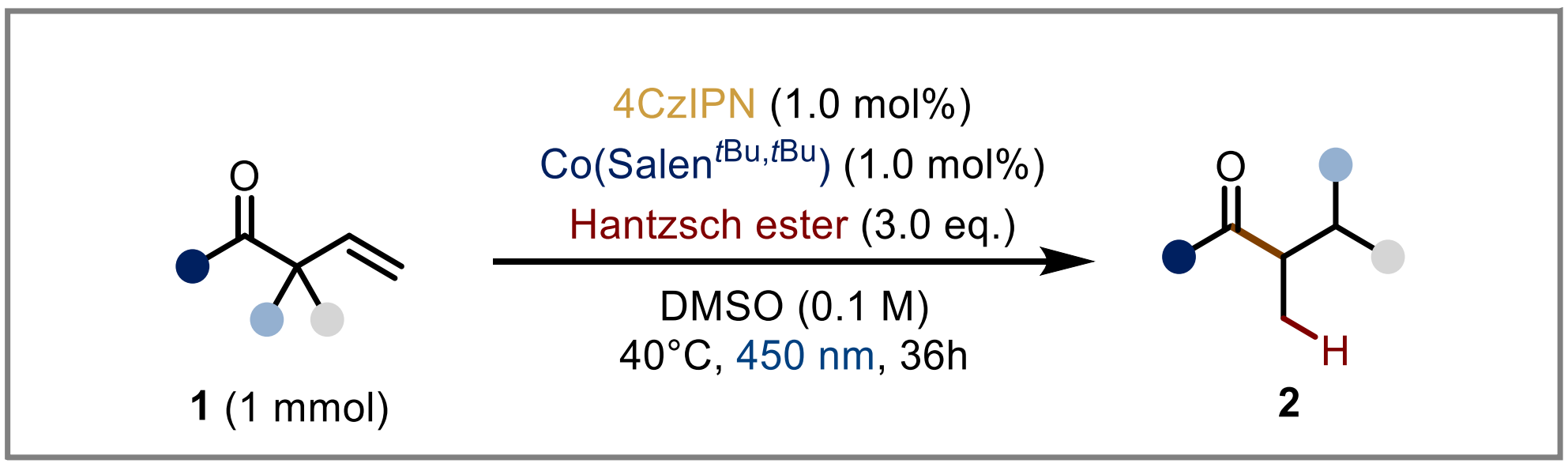

In an oven-dried 15 mL vial equipped with a magnetic stir bar were placed *β,γ*-unsaturated ketone **1** (1.0 mmol), 4CzIPN (0.01 mmol, 1.0 mol%, 8.0 mg), Co(Salen^{tBu,tBu}) (0.01 mmol, 1.0 mol%, 6.0 mg), and Hantzsch ester (3.0 mmol, 3.0 equiv, 759.0 mg). The vial was sealed with a rubber septum, and the reaction mixture was degassed by purging with argon for 15 min. DMSO (10.0 mL) was then added. The vial was placed in the PhotoRedOx Box (see Materials and Methods for details of the photochemical setup) and irradiated with 450 nm light for 36 h without cooling fan; under these conditions, the reaction temperature was approximately 40 °C. Upon completion, the reaction mixture was extracted with EtOAc and the organic phase was washed with water (3 ×) to remove residual DMSO. The organic layer was dried over $MgSO_4$, filtered, and concentrated under reduced pressure.

Purification of the residue by silica gel column chromatography (pentane : EtOAc) afforded the corresponding product.

1-mesityl-2,3-dimethylbutan-1-one (2a)

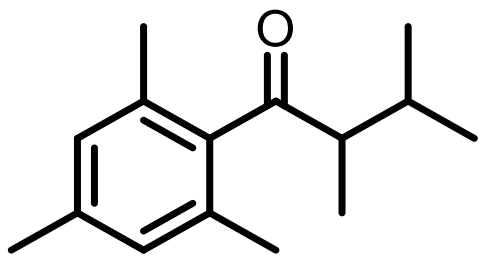

Prepared following general procedure for 1 mmol-scale 1,2-Carbonyl Migration, **2a** was obtained after purification by column chromatography (pentane : EtOAc 100 :1) as a colorless oil (148.3 mg, 0.68 mmol, 68%). The characterization data were consistent with those reported above.

1-cyclohexyl-2,3-dimethylbutan-1-one (2q)

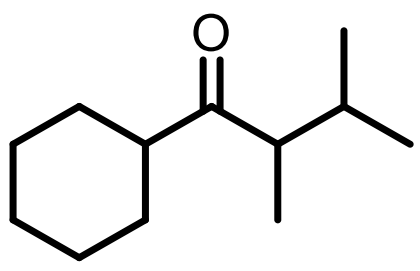

Prepared following general procedure for the 1,2-Carbonyl Migration, **2q** was obtained after purification by column chromatography (pentane : EtOAc 50 :1) as a pale yellow oil (141.8 mg, 0.78 mmol, 78%). The characterization data were consistent with those reported above.

5. Synthetic transformation

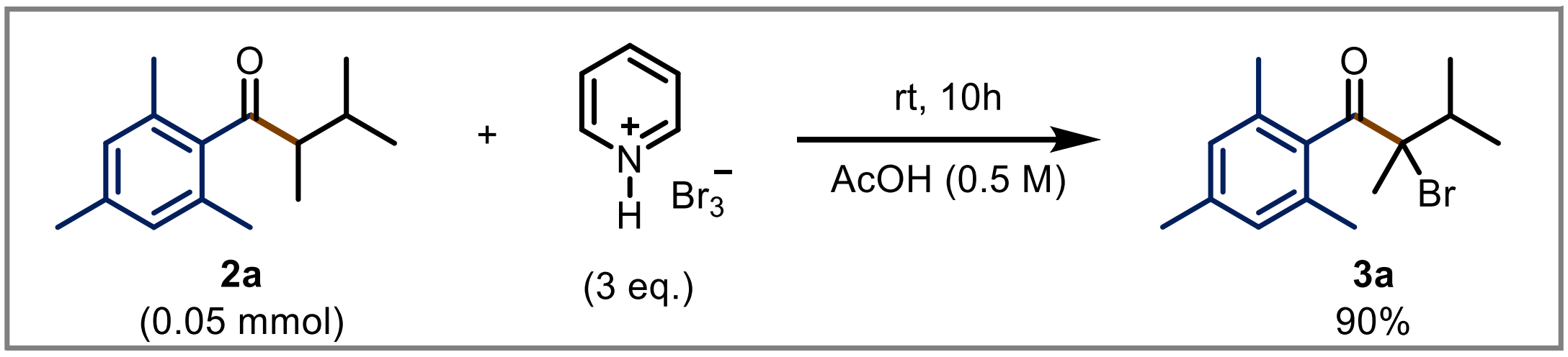

Ketone 2a (0.05 mmol, 1.0 equiv) and pyridinium tribromide (48.0 mg, 0.15 mmol, 3.0 equiv) were dissolved in AcOH (0.10 mL, 0.50 M). The reaction mixture was stirred at room temperature for 10 h. Upon completion, the mixture was diluted with water and extracted with dichloromethane. The combined organic layers were washed sequentially with saturated aqueous $NaHCO_3$ solution and brine, dried over $MgSO_4$, filtered, and concentrated under reduced pressure. The resulting residue was purified by flash column chromatography (pentane: EtOAc, 50:1) to afford 3a as a pale yellow oil (13.4 mg, 90%).

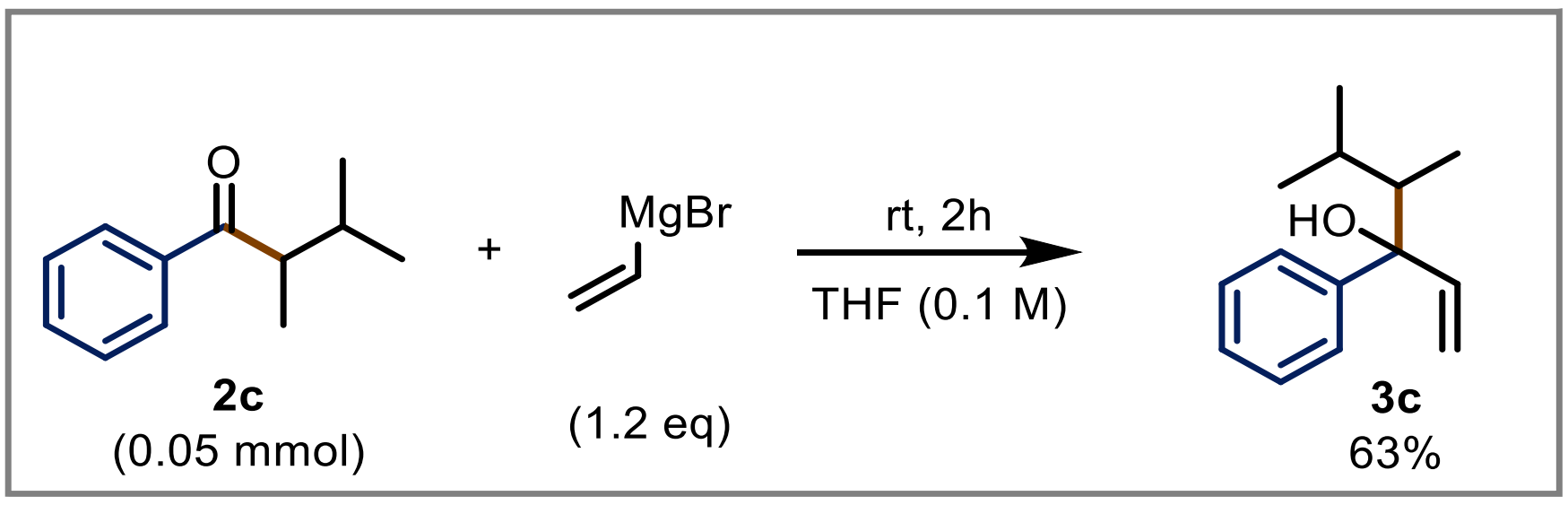

Ketone 2c (0.050 mmol, 1.0 equiv) was placed in a 4 mL vial and dissolved in anhydrous THF (0.50 mL, 0.10 M). The solution was cooled to 0 °C and stirred for 5 min, after which vinylmagnesium bromide (1.0 M in THF, 60 µL, 0.060 mmol, 1.2 equiv) was added. The reaction mixture was allowed to warm to room temperature and stirred for 2 h. The reaction was quenched with saturated aqueous NH_4Cl, and the mixture was extracted with ethyl acetate. The combined organic layers were washed with brine, dried over anhydrous $MgSO_4$, filtered, and concentrated under reduced pressure. The residue was purified by flash column chromatography (pentane : EtOAc, 20 :1) to afford 3c as a colorless oil (6.4 mg, 63%).

5.1 Characterisation data

2-bromo-1-mesityl-2,3-dimethylbutan-1-one (3a)

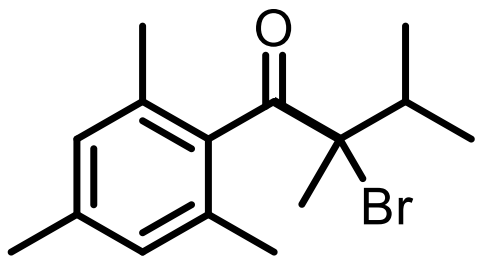

^{1}H NMR (400 MHz, $CDCl_3$) δ 6.84 (d, J = 12.8 Hz, 2H), 2.51 – 2.41 (m, 1H), 2.33 (s, 3H), 2.28 (s, 3H), 2.20 (s, 3H), 1.85 (s, 3H), 1.20 (d, J = 6.6 Hz, 3H), 1.09 (d, J = 6.6 Hz, 3H). **^{13}C NMR** (101 MHz, $CDCl_3$) δ 207.6, 138.3, 137.5, 134.4, 133.2, 128.8, 128.6, 77.6, 36.3, 26.2, 21.3, 21.1, 20.8, 20.3, 18.9. **HRMS(ESI):** calculated for $C_{15}H_{21}BrONa$ $[M+Na]^+$: 319.0668, found: 319.0668.

4,5-dimethyl-3-phenylhex-1-en-3-ol (3c)

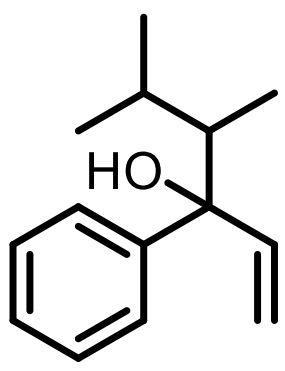

^{1}H NMR (400 MHz, $CDCl_3$) δ 7.46 – 7.41 (m, 2H), 7.36 – 7.31 (m, 2H), 7.25 – 7.19 (m, 1H), 6.24 (dd, *J* = 17.2, 10.7 Hz, 1H), 5.25 (dd, *J* = 17.2, 1.0 Hz, 1H), 5.11 (dd, *J* = 10.7, 1.0 Hz, 1H), 1.96 (qd, *J* = 7.2, 2.2 Hz, 1H), 1.65 (pd, *J* = 6.9, 2.2 Hz, 1H), 0.88 (d, *J* = 7.2 Hz, 3H), 0.80 (d, *J* = 6.9 Hz, 6H). **^{13}C NMR** (101 MHz, $CDCl_3$) δ 146.3, 145.2, 128.3, 126.5, 111.7, 80.8, 46.6, 26.9, 23.8, 17.4, 7.7. **HRMS(ESI):** calculated for $C_{14}H_{20}ONa$ $[M+Na]^+$: 227.1406, found: 227.1405.

6. Deuteration experiments

All reactions were conducted according to the general procedure for 1,2-Carbonyl Migration of *β,γ*-unsaturated ketone, using the different deuterated Hantzsch esters, which were synthesized by following reported procedures.[13]

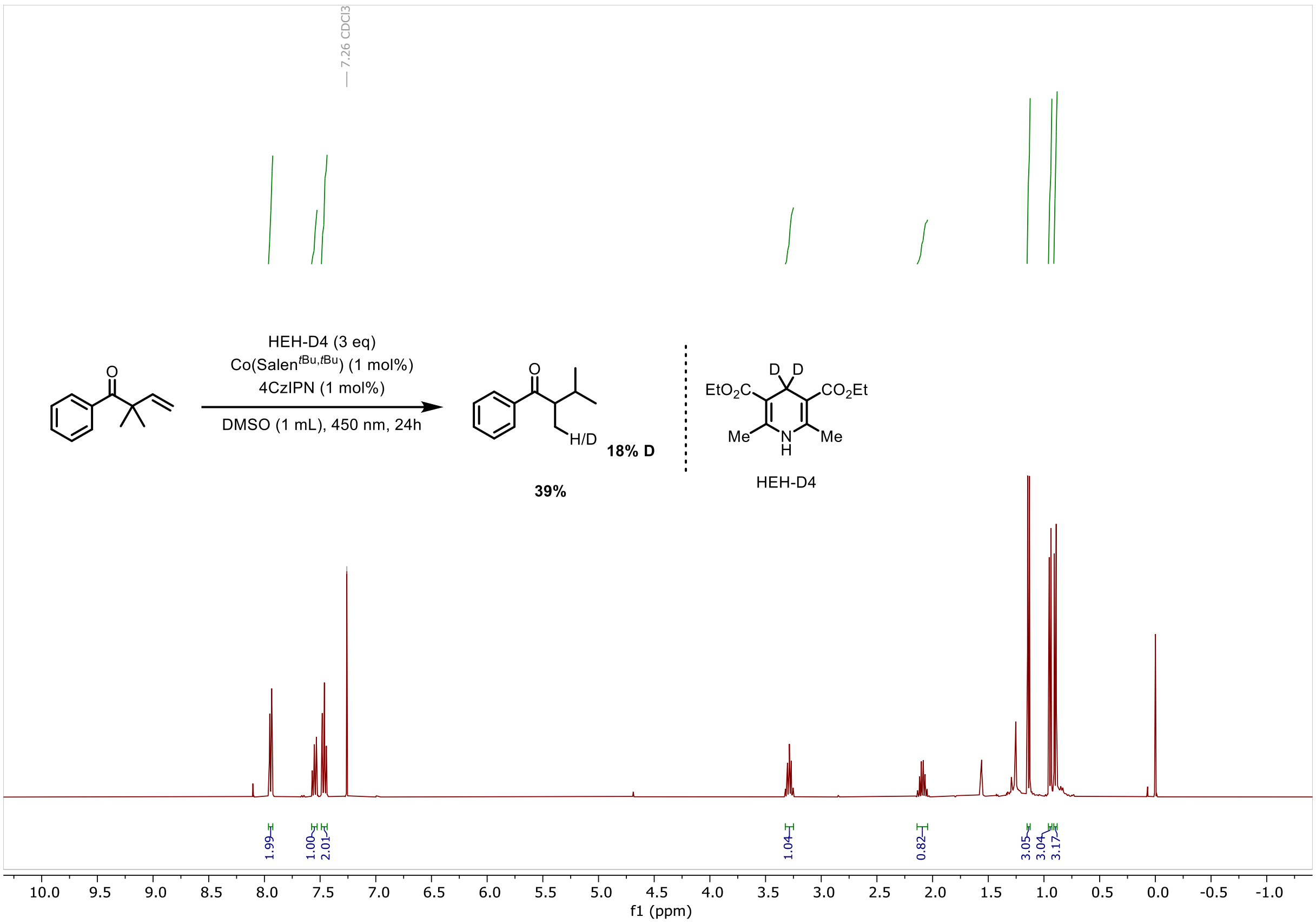

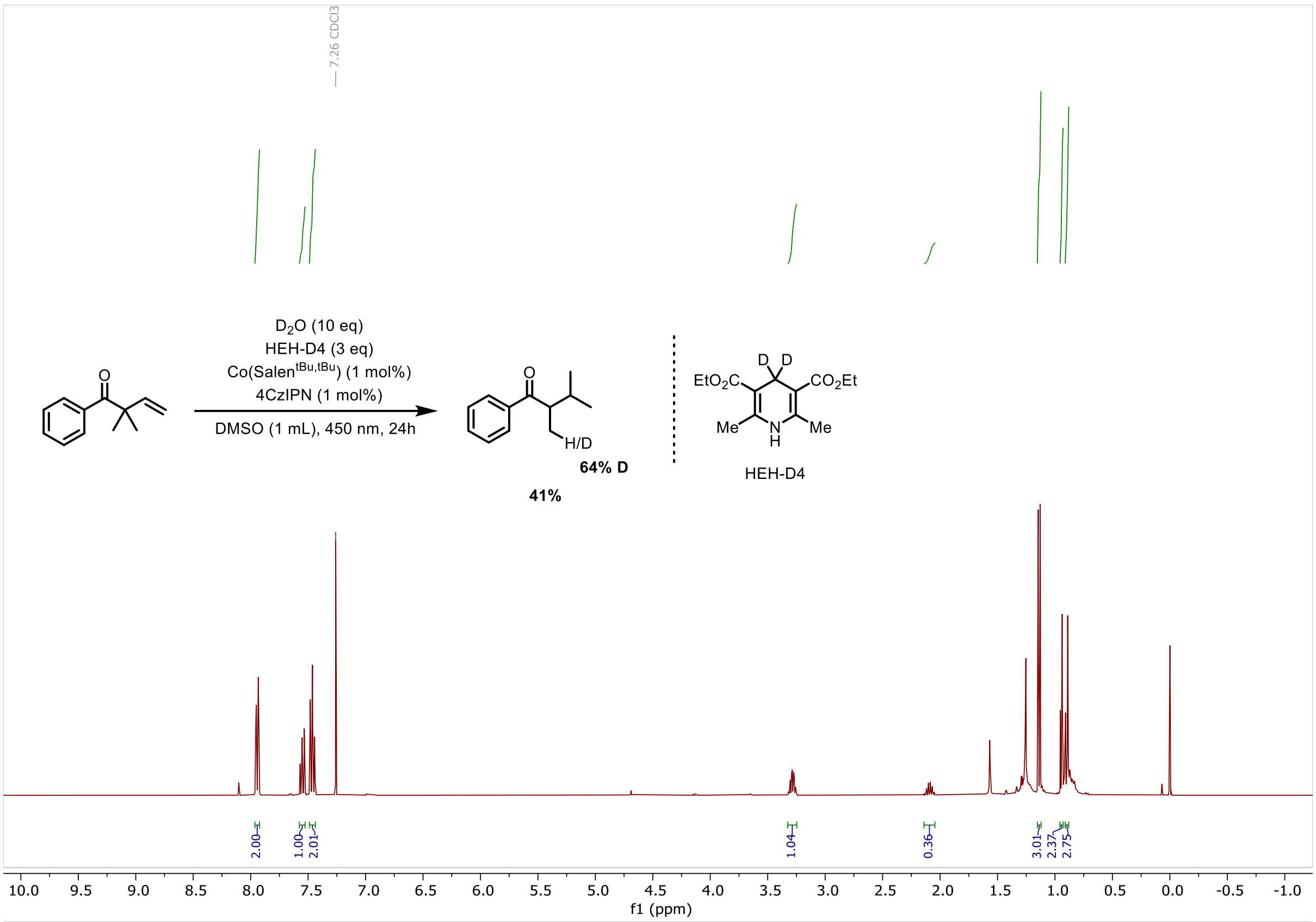

7.26 CDCl3
D2O (10 eq)
HEH-D4 (3 eq)
Co(Salen^tBu,tBu) (1 mol%)
4CzIPN (1 mol%)
DMSO (1 mL), 450 nm, 24h
H/D
64% D
41%
EtO2C
CO2Et
Me
Me
HEH-D4
2.00
1.00
2.01
1.04
0.36
3.01
2.37
2.75
f1 (ppm)

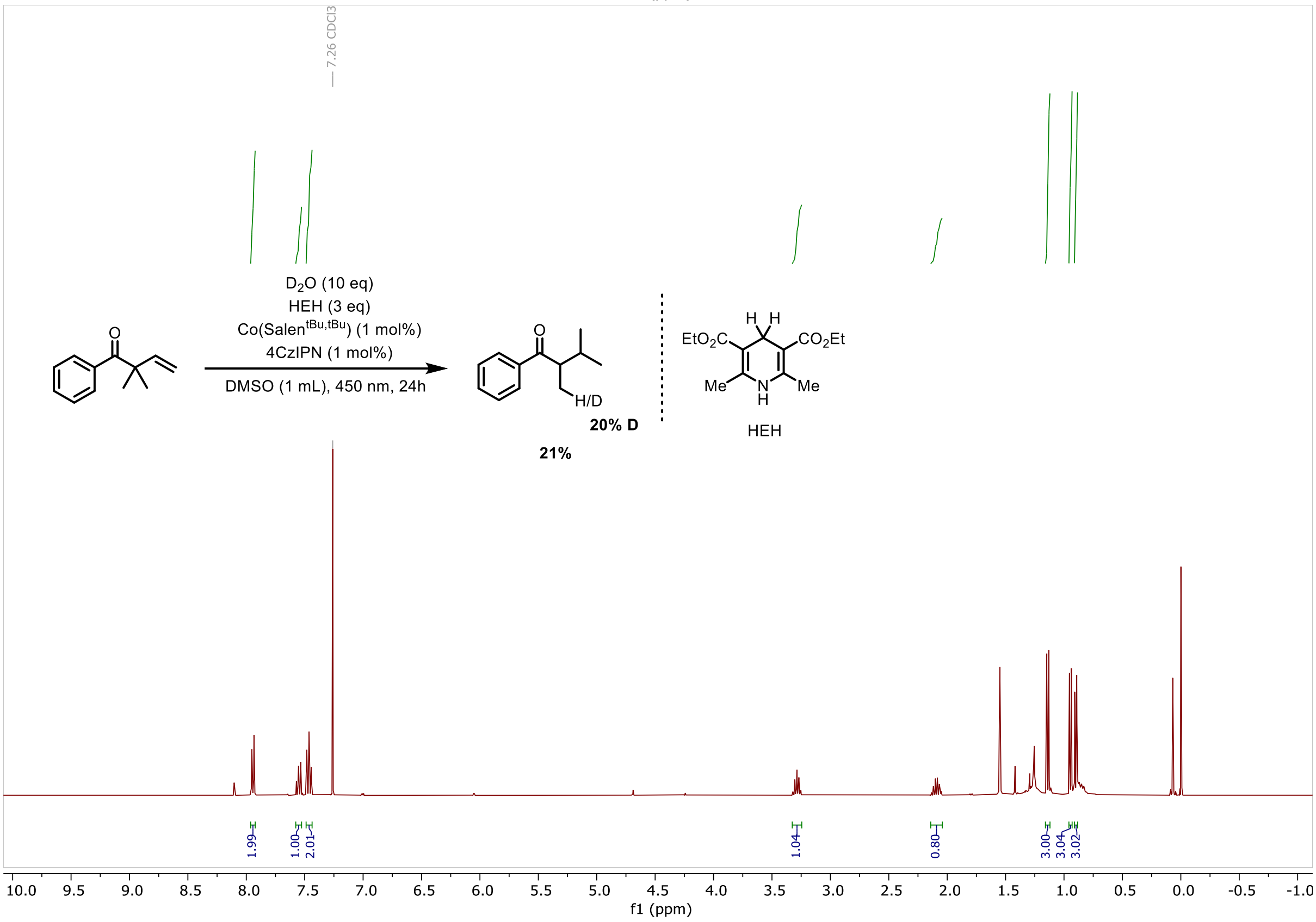

7.26 CDCl3
D2O (10 eq)
HEH (3 eq)
Co(Salen^tBu,tBu) (1 mol%)
4CzIPN (1 mol%)
DMSO (1 mL), 450 nm, 24h
H/D
20% D
21%
EtO2C
CO2Et
Me
Me
HEH
1.99
1.00
2.01
1.04
0.80
3.00
3.04
3.02
f1 (ppm)

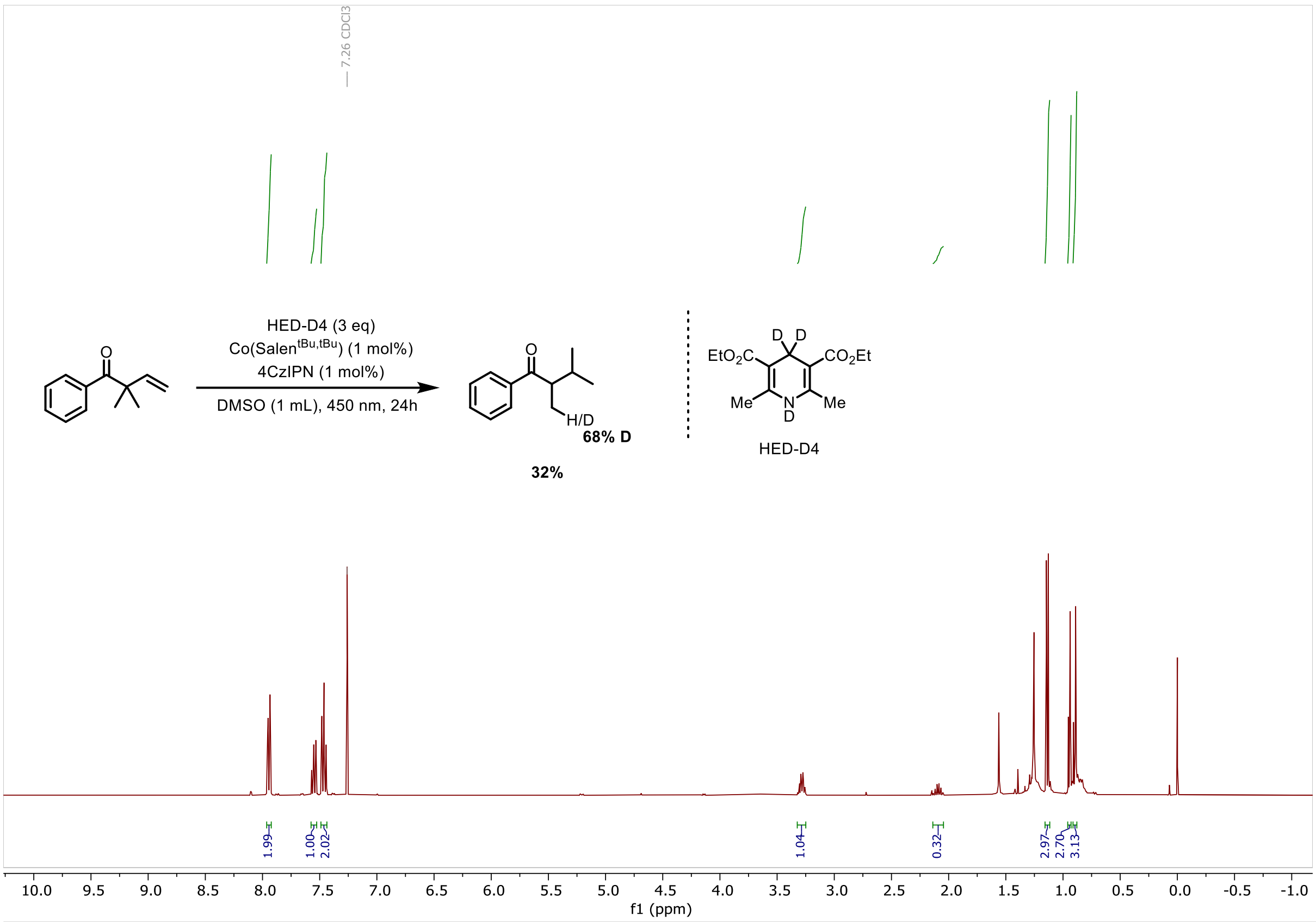
HED-D4 (3 eq)
Co(Salen^tBu,tBu) (1 mol%)
4CzIPN (1 mol%)
DMSO (1 mL), 450 nm, 24h
H/D
68% D
32%
EtO2C
CO2Et
Me
Me
HED-D4
7.26 CDCl3
1.99
1.00
2.02
1.04
0.32
2.97
2.70
3.13
f1 (ppm)

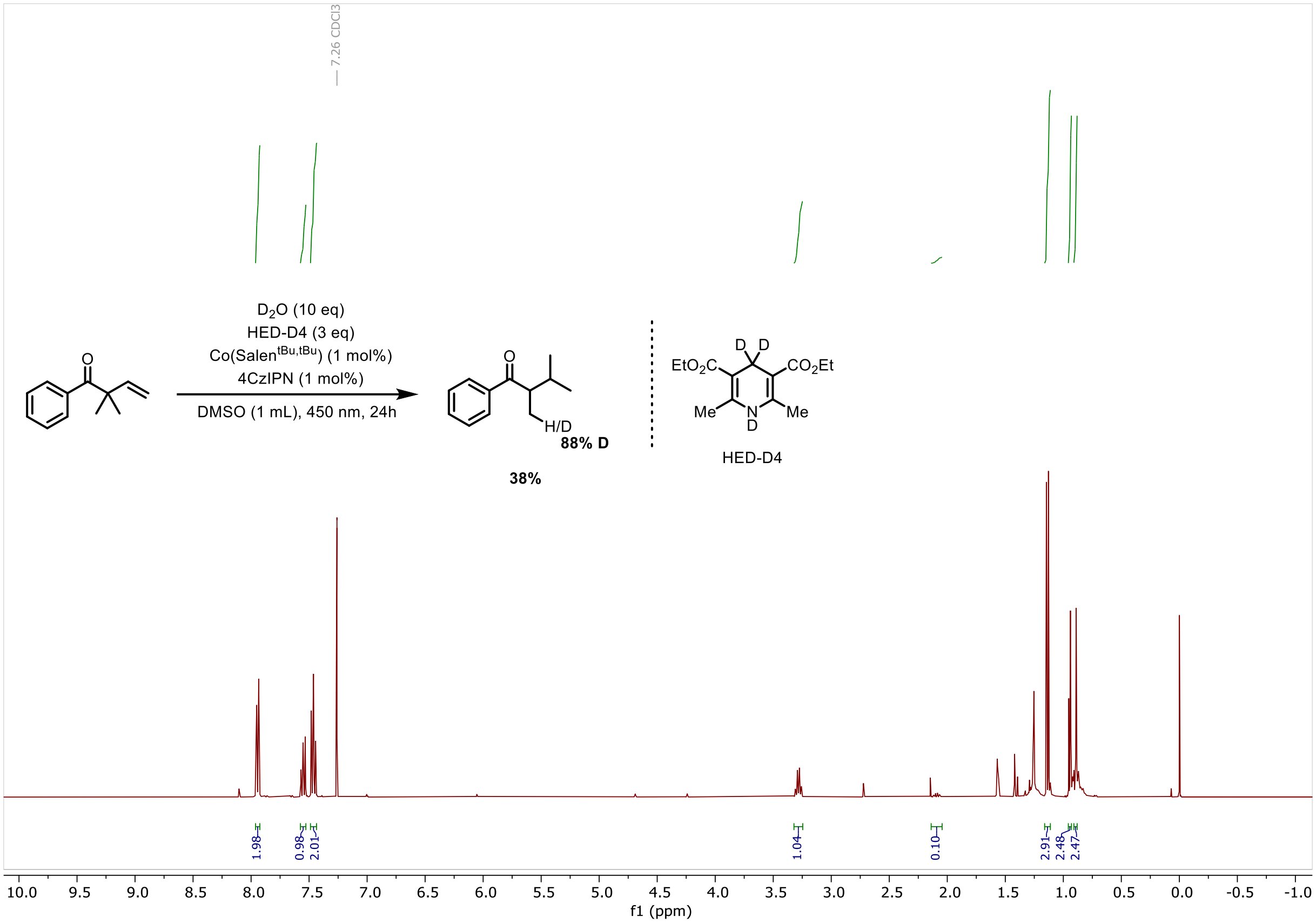
D2O (10 eq)
HED-D4 (3 eq)
Co(Salen^tBu,tBu) (1 mol%)
4CzIPN (1 mol%)
DMSO (1 mL), 450 nm, 24h
H/D
88% D
38%
EtO2C
CO2Et
Me
Me
HED-D4
7.26 CDCl3
1.98
0.98
2.01
1.04
0.10
2.91
2.48
2.47
f1 (ppm)

7. Radical inhibition experiment

The reaction of **1f** was conducted according to the general procedure for 1,2-carbonyl migration in the presence of TEMPO (0.25 mmol, 39.0 mg, 2.5 equiv.). The reaction was completely suppressed, suggesting a radical pathway is involved.

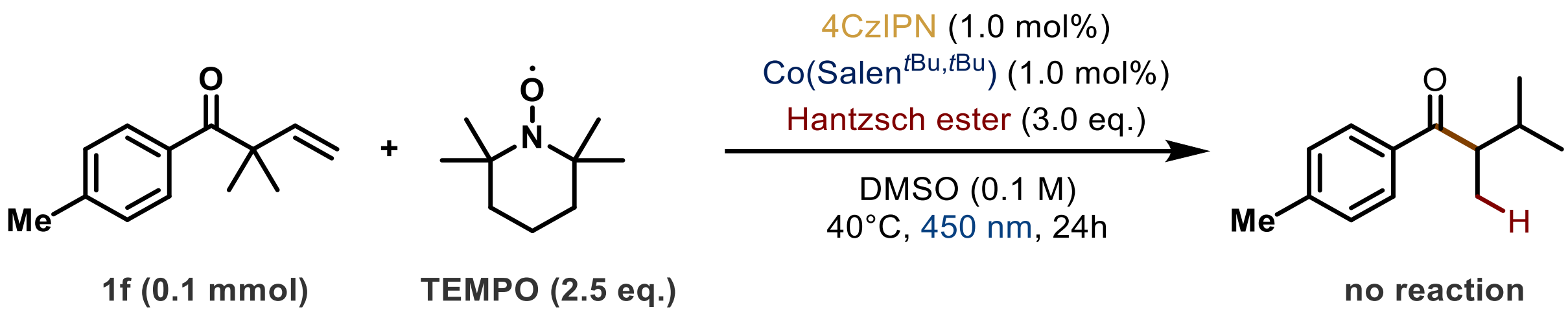

8. Mechanistic investigation

8.1 Synthesis of 4CzMeBN

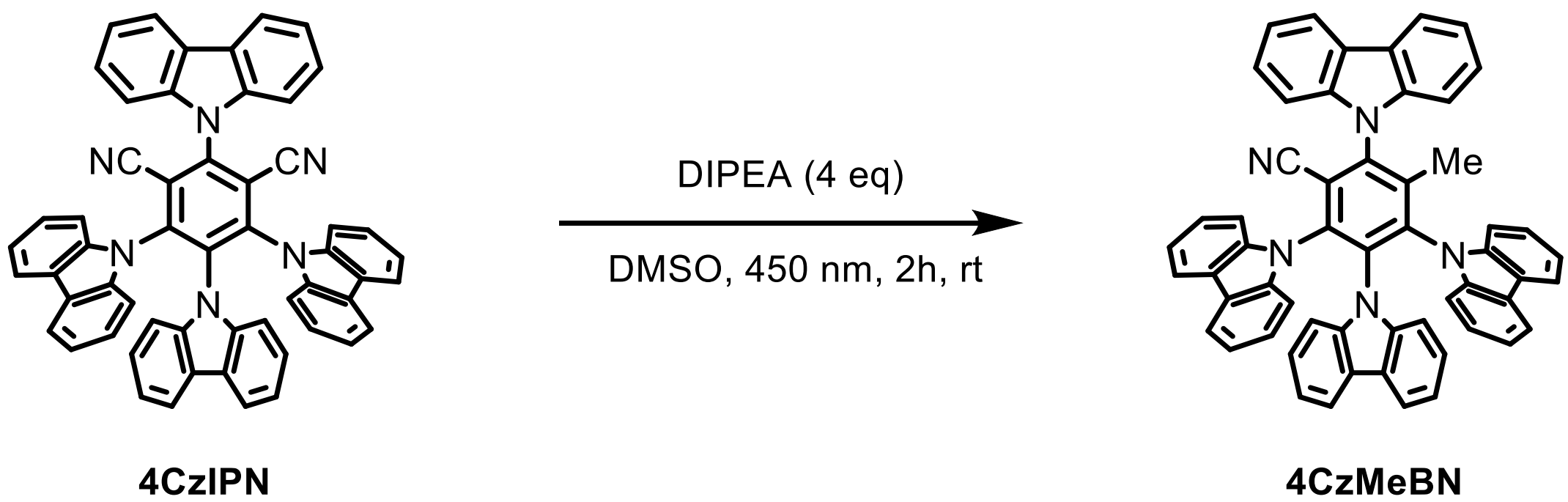

4CzMeBN was prepared using a procedure adapted from Grotjahn and König.[14] 4CzIPN (284 mg, 0.360 mmol, 1.0 equiv) and DIPEA (251 µL, 1.44 mmol, 4.0 equiv) were placed in a single vial and dissolved in DMSO (10.0 mL, 0.036 M). The solution was irradiated under air for 2 h. After irradiation, the reaction mixture was diluted with ethyl acetate, and the organic layer was washed with water (3×) to remove DMSO and then with brine. The organic layer was dried over anhydrous $MgSO_4$, filtered, and concentrated under reduced pressure. The residue was purified by flash column chromatography (pentane : EtOAc, 5:1) to afford 4CzMeBN as a yellow solid (118 mg, 0.152 mmol, 42%).

2,3,4,6-Tetra(9H-carbazol-9-yl)-5-methylbenzonitrile–4CzMeBN

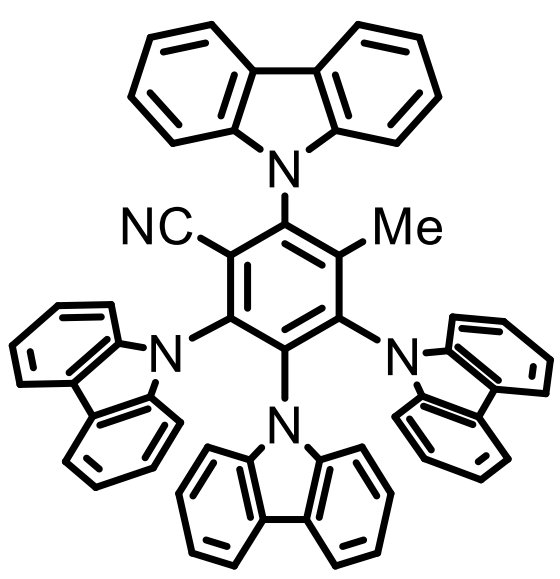

4CzMeBN

^{1}H NMR (400 MHz, $CDCl_3$) δ 8.24 (d, *J* = 7.6 Hz, 2H), 7.76 – 7.65 (m, 6H), 7.52 (d, *J* = 8.1 Hz, 2H), 7.47 – 7.40 (m, 2H), 7.33 – 7.25 (m, 4H), 7.11 – 7.02 (m, 10H), 6.94 (d, *J* = 8.2 Hz, 2H), 6.77 (td, *J* = 7.5, 0.9 Hz, 2H), 6.64 (ddd, *J* = 8.3, 7.2, 1.2 Hz, 2H), 1.77 (s, 3H). **HRMS(ESI):** calculated for $C_{56}H_{35}N_5Na$ $[M+Na]^+$: 800.2784, found: 800.2785. These data are in accordance with those reported previously.[14,15]

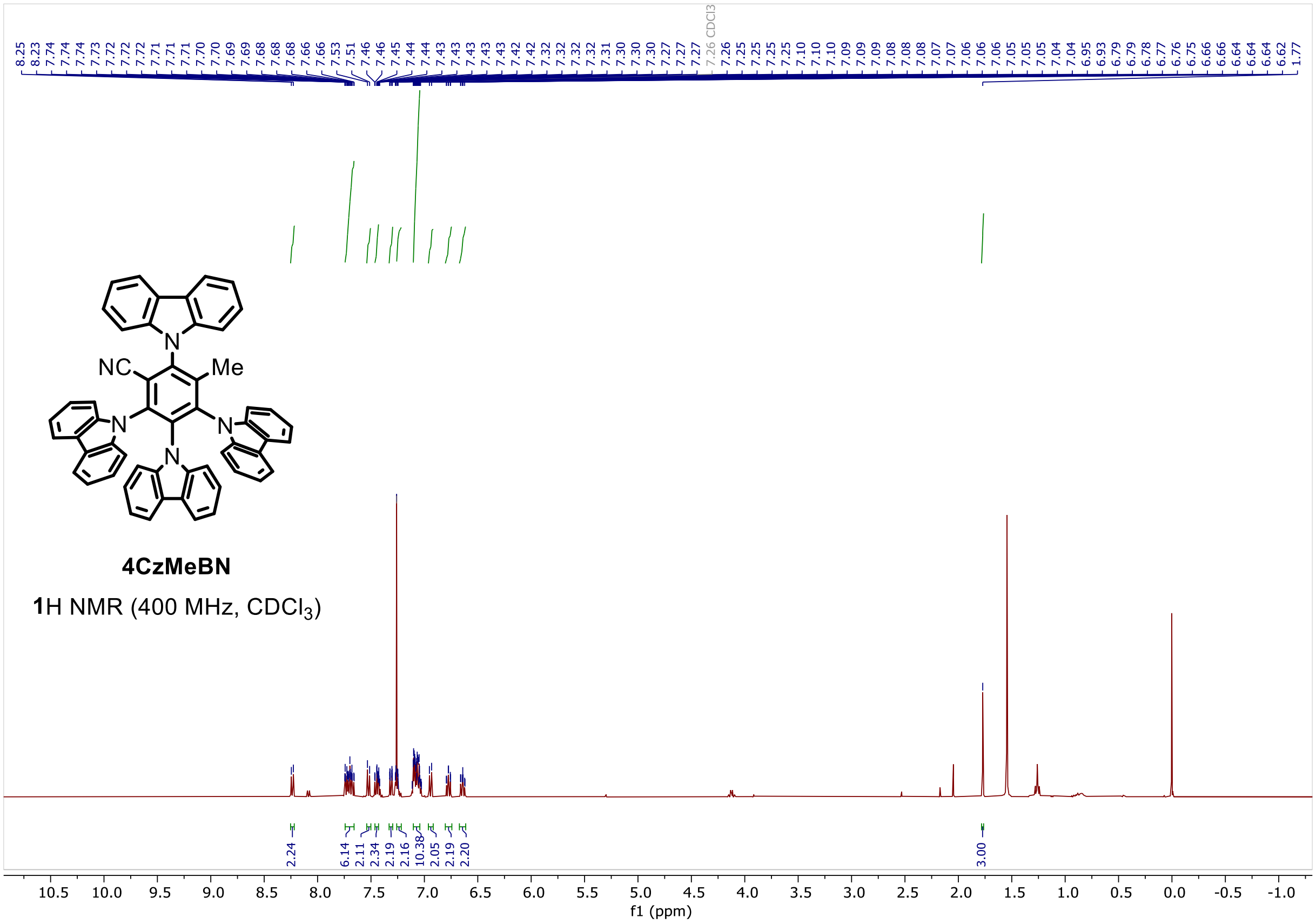

8.2 Control Reaction with 4CzMeBN

We then performed a control experiment using independently prepared 4CzMeBN as the photocatalyst under the standard reaction conditions. Full conversion was observed, and the desired product was obtained in quantitative yield, as determined by ^{1}H NMR using CH_2Br_2 as an internal standard.

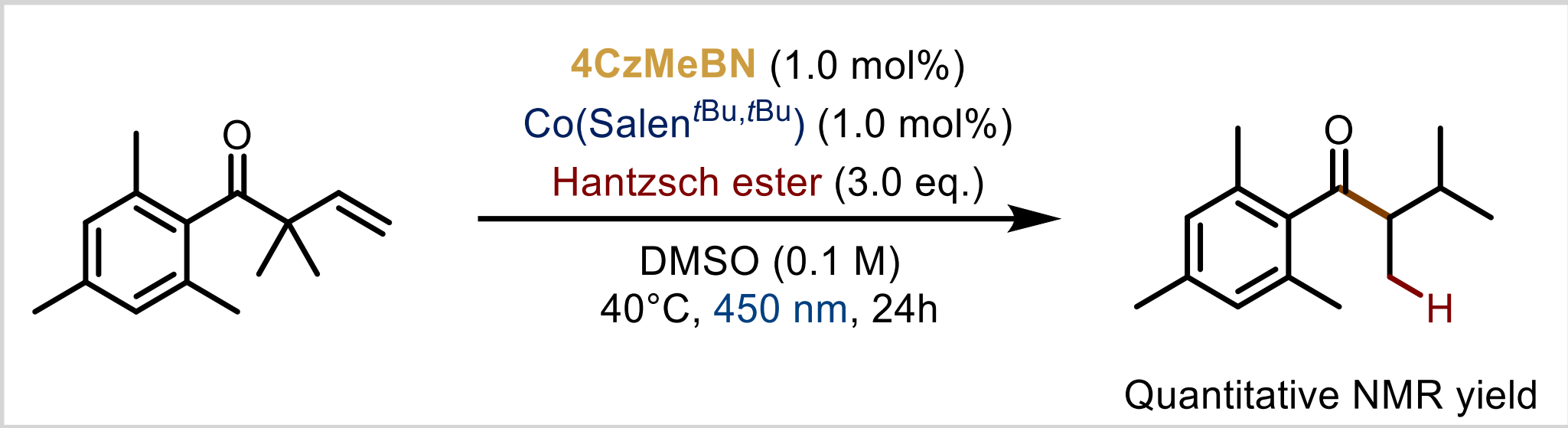

8.3 Investigation of the *In Situ* Formation of 4CzMeBN

To assess the *in situ* formation of 4CzMeBN, the reaction was conducted using 10 mol% 4CzIPN to facilitate its detection. Isolation of pure 4CzMeBN was prevented by coelution with the oxidized Hantzsch ester and its low concentration. Nevertheless, ^{1}H NMR analysis of the partially purified fraction revealed signals that closely matched those of independently prepared 4CzMeBN (Red: independently prepared 4CzMeBN; blue: partially purified fraction obtained from the reaction mixture.)

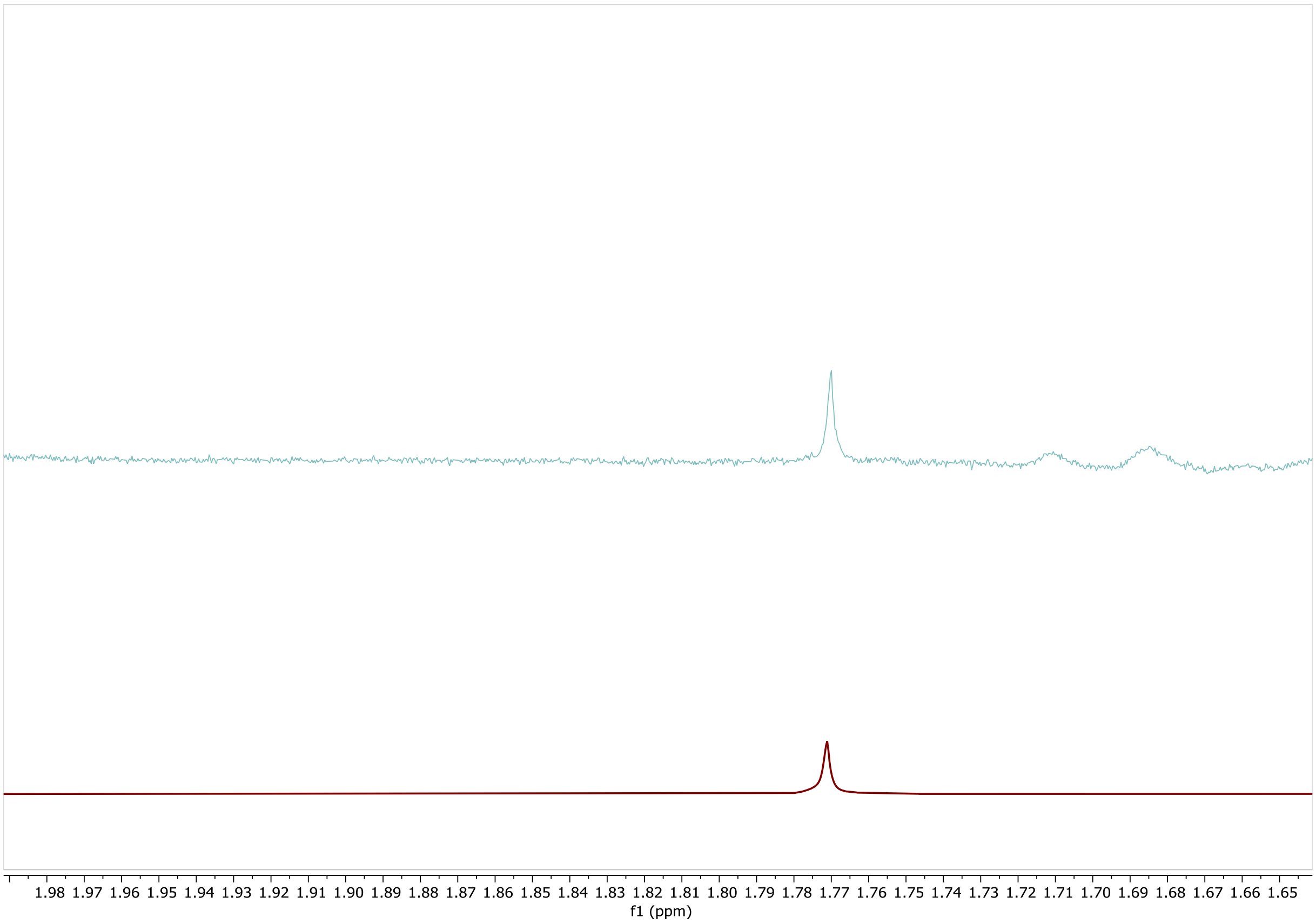

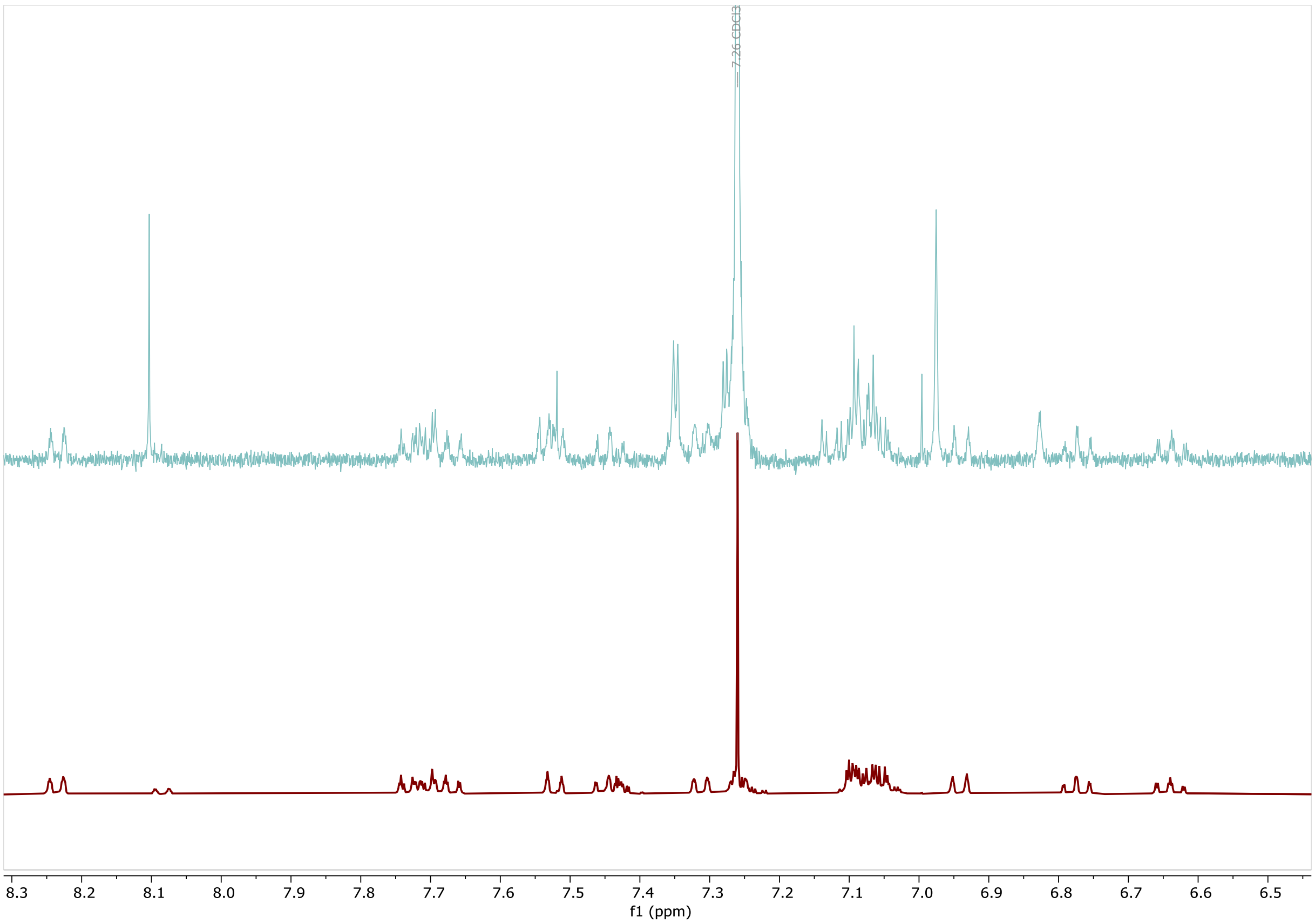

Additionally, the reaction mixture was analyzed by HRMS, confirming the presence of 4CzMeBN. **HRMS(ESI):** calculated for $C_{56}H_{35}N_5Na$ $[M+Na]^+$: 800.2784, found: 800.2785.

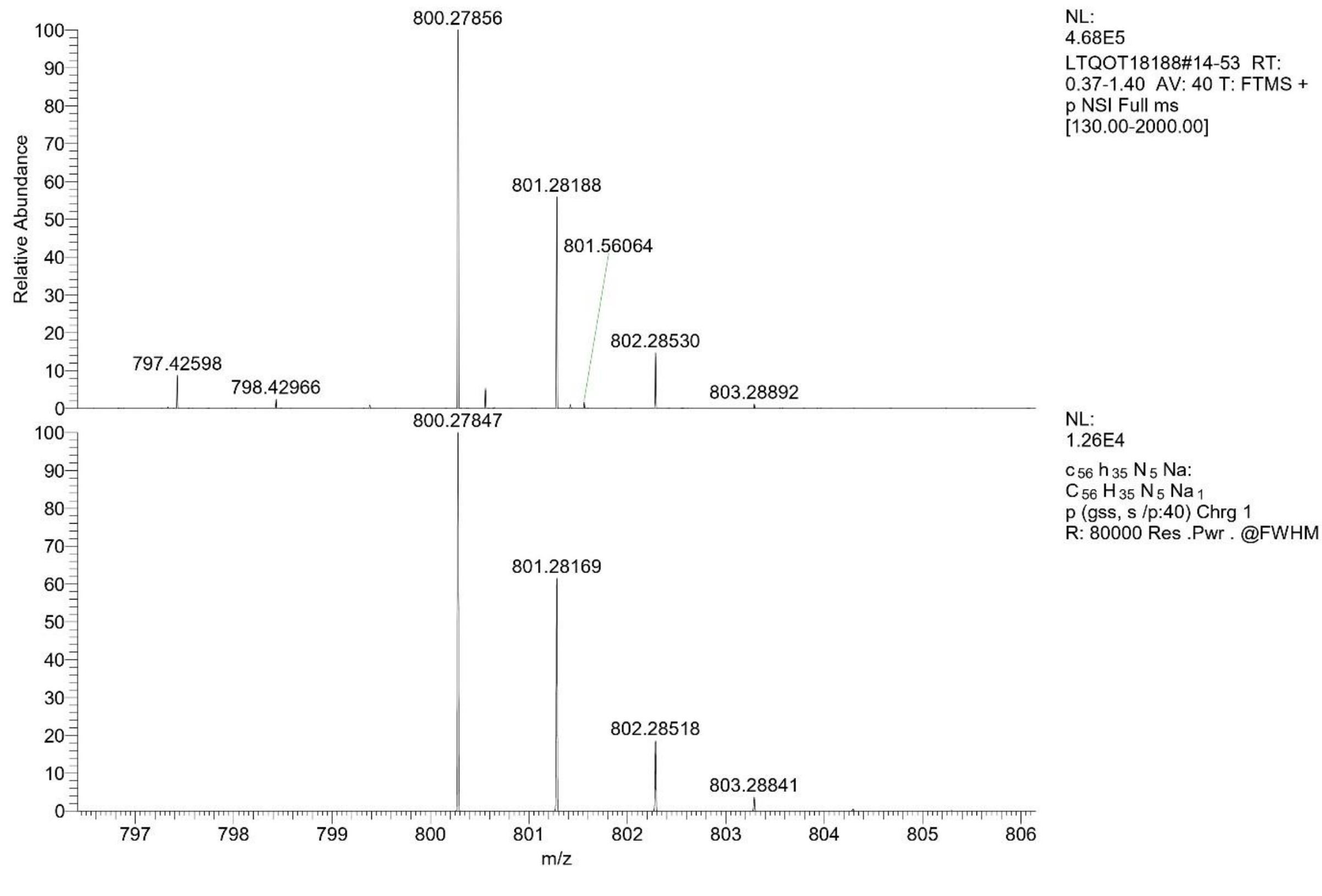

9. References

(1) Li, J.-Y.; Liu, Y.; Sun, D.; Wu, G.; Yue, S.-S.; Yang, H.-Y.; Zhang, H.; Zhang, W.; Zheng, M.-M.; Xue, X.-S.; Guo, S. Radical 1,3-Difunctionalization of β,γ-Unsaturated Ketones via Concomitant 1,2-Carbonyl Migration: An Entrance to β-SF_5 -/β-CF_3 SF_4 Ketones. *J. Am. Chem. Soc.* **2025**, *147* (30), 26124–26132. https://doi.org/10.1021/jacs.5c09085

(2) Liu, R.; Tian, Y.; Wang, J.; Wang, Z.; Li, X.; Zhao, C.; Yao, R.; Li, S.; Yuan, L.; Yang, J.; Shi, D. Visible Light–Initiated Radical 1,3-Difunctionalization of β,γ-Unsaturated Ketones. *Science Advances* **2022**, *8* (49), eabq8596. https://doi.org/10.1126/sciadv.abq8596

(3) Jiang, C.; Lu, H.; Xu, W.-H.; Wu, J.; Yu, T.-Y.; Xu, P.-F.; Wei, H. Ni-Catalyzed 1,2-Acyl Migration Reactions Triggered by C–C Bond Activation of Ketones. *ACS Catal.* **2020**, *10* (3), 1947–1953. https://doi.org/10.1021/acscatal.9b04112

(4) Yan, S.-G.; Li, Y.; Wu, S.; Hu, M.; Wang, P.; Li, J.-H. Photocatalytic 1,2,3-Trifunctionalization of β,γ-Unsaturated Ketones via 1,2-Carbonyl Migration to Access Functionalized Indanones. *Org. Lett.* **2026**, *28* (9), 3084–3089. https://doi.org/10.1021/acs.orglett.6c00457

(5) Zheng, X.; Li, X.; Han, X.; Zhang, X.; Zhang, Q.; Yao, T.; Zhang, G. Visible-Light-Induced Radical 1,3-Hydrosulfonylation of Allylketones with Sulfonyl Chlorides. *Chem. Commun.* **2025**, *61* (54), 9916–9919. https://doi.org/10.1039/d5cc01285f

(6) Luo, M.; Zhu, S.; Yin, J.; Yang, C.; Guo, L.; Xia, W. Photoinduced Difunctionalizations of Unactivated Olefins Enabled by Ligand-to-Iron Charge Transfer and Functional Group Migration Strategies. *Org. Chem. Front.* **2024**, *11* (17), 4748–4756. https://doi.org/10.1039/D4QO01025F

(7) Yuan, Y.; Zhang, X.; Qian, H.; Ma, S. Catalytic Enantioselective Allene–Anhydride Approach to β,γ-Unsaturated Enones Bearing an α-All-Carbon-Quarternary Center. *Chem. Sci.* **2020**, *11* (34), 9115–9121. https://doi.org/10.1039/d0sc03227a

(8) Shi, C.; Liu, R.; Wang, Z.; Li, X.; Qin, H.; Yuan, L.; Shan, W.; Zhuang, W.; Li, X.; Shi, D. Radical Addition-Enabled C–C σ-Bond Cleavage/Reconstruction to Access Functional Indanones: Total Synthesis of Carexane L. *Org. Lett.* **2024**, *26* (15), 2913–2917. https://doi.org/10.1021/acs.orglett.4c00348

(9) Chan, L. K. M.; Poole, D. L.; Shen, D.; Healy, M. P.; Donohoe, T. J. Rhodium-Catalyzed Ketone Methylation Using Methanol Under Mild Conditions: Formation of α-Branched Products. *Angewandte Chemie International Edition* **2014**, *53* (3), 761–765. https://doi.org/10.1002/anie.201307950

(10) Singhal, N.; Koner, A. L.; Mal, P.; Venugopalan, P.; Nau, W. M.; Moorthy, J. N. Diastereomer-Differentiating Photochemistry of β-Arylbutyrophenones: Yang Cyclization versus Type II Elimination. *J. Am. Chem. Soc.* **2005**, *127* (41), 14375–14382. https://doi.org/10.1021/ja0523643

(11) Ganguli, K.; Mandal, A.; Kundu, S. Well-Defined Bis(NHC)Mn(I) Complex Catalyzed Tandem Transformation of α,β-Unsaturated Ketones to α-Methylated Ketones Using Methanol. *ACS Catal.* **2022**, *12* (19), 12444–12457. https://doi.org/10.1021/acscatal.2c04131

(12) Wang, Q.-Q.; Wang, Z.-X.; Zhang, X.-Y.; Fan, X.-S. Microwave-Promoted Metal-Free α-Alkylation of Ketones with Cycloalkanes through Cross-Coupling of C(Sp3)−H Bonds. *Asian Journal of Organic Chemistry* **2017**, *6* (10), 1445–1450. https://doi.org/10.1002/ajoc.201700256

(13) Bergamaschi, E.; Mayerhofer, V. J.; Teskey, C. J. Light-Driven Cobalt Hydride Catalyzed Hydroarylation of Styrenes. *ACS Catal.* **2022**, *12* (24), 14806–14811. https://doi.org/10.1021/acscatal.2c05109

(14) Grotjahn, S.; König, B. Photosubstitution in Dicyanobenzene-Based Photocatalysts. *Org. Lett.* **2021**, *23* (8), 3146–3150. https://doi.org/10.1021/acs.orglett.1c00836

(15) Jana, S.; Kusza, D. A.; Vystavkin, N.; Lunic, D.; Teskey, C. J. Inverting Polarity in a Cobalt MHAT Reaction via Reductive Catalytic Turnover. *Chem. Sci.* **2026**, *17* (2), 1098–1104. https://doi.org/10.1039/D5SC06119A

8. NMR Spectra

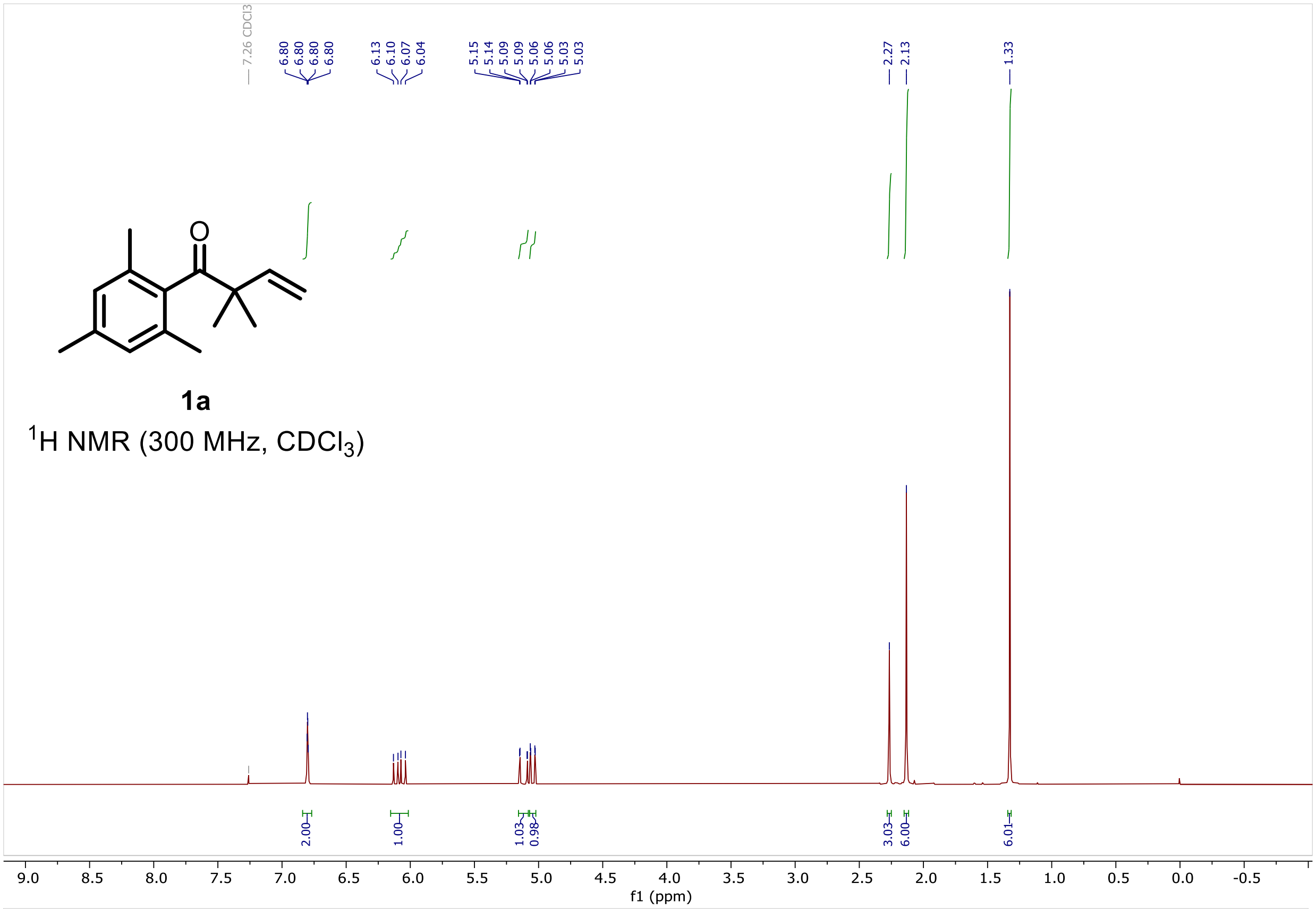

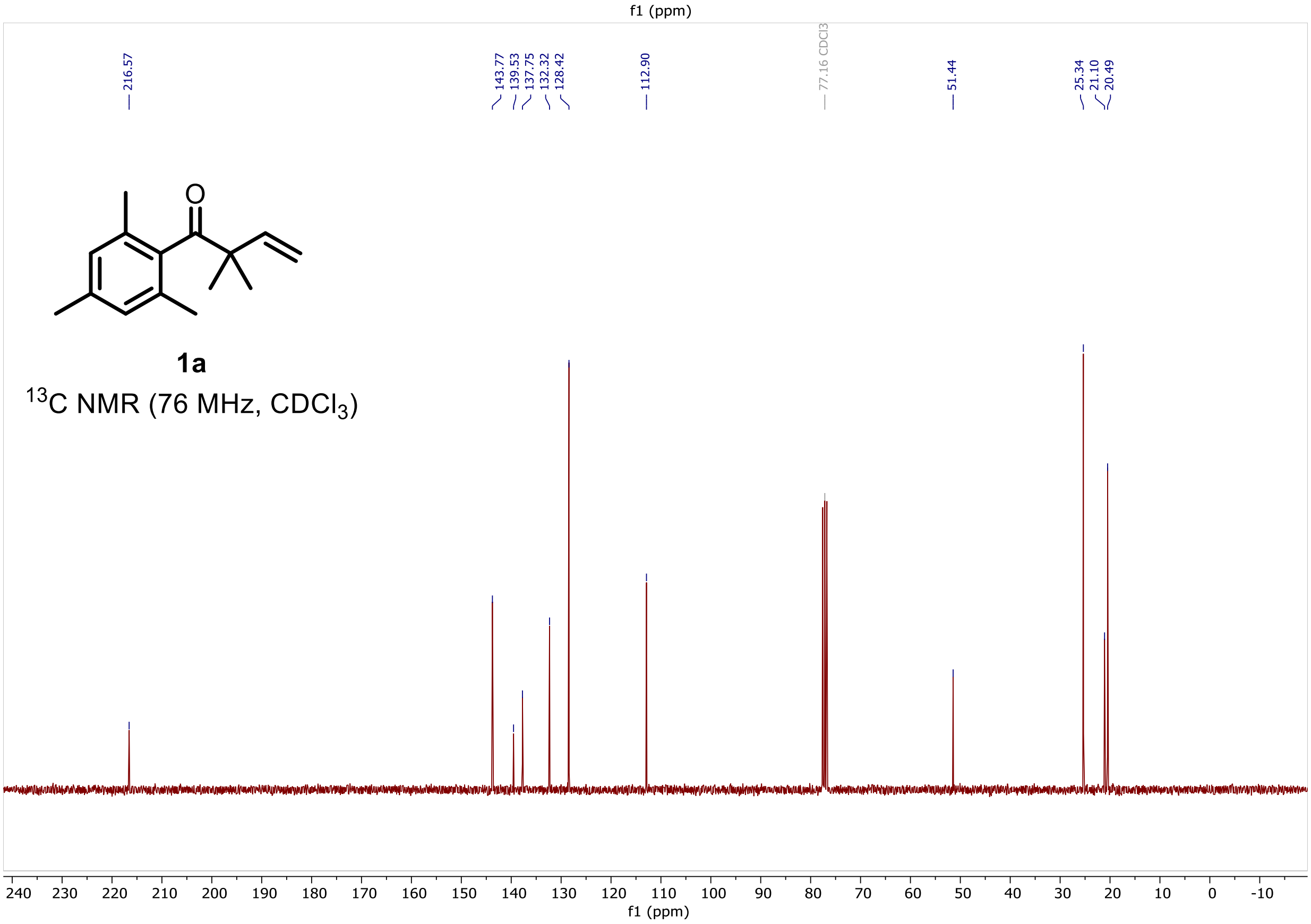

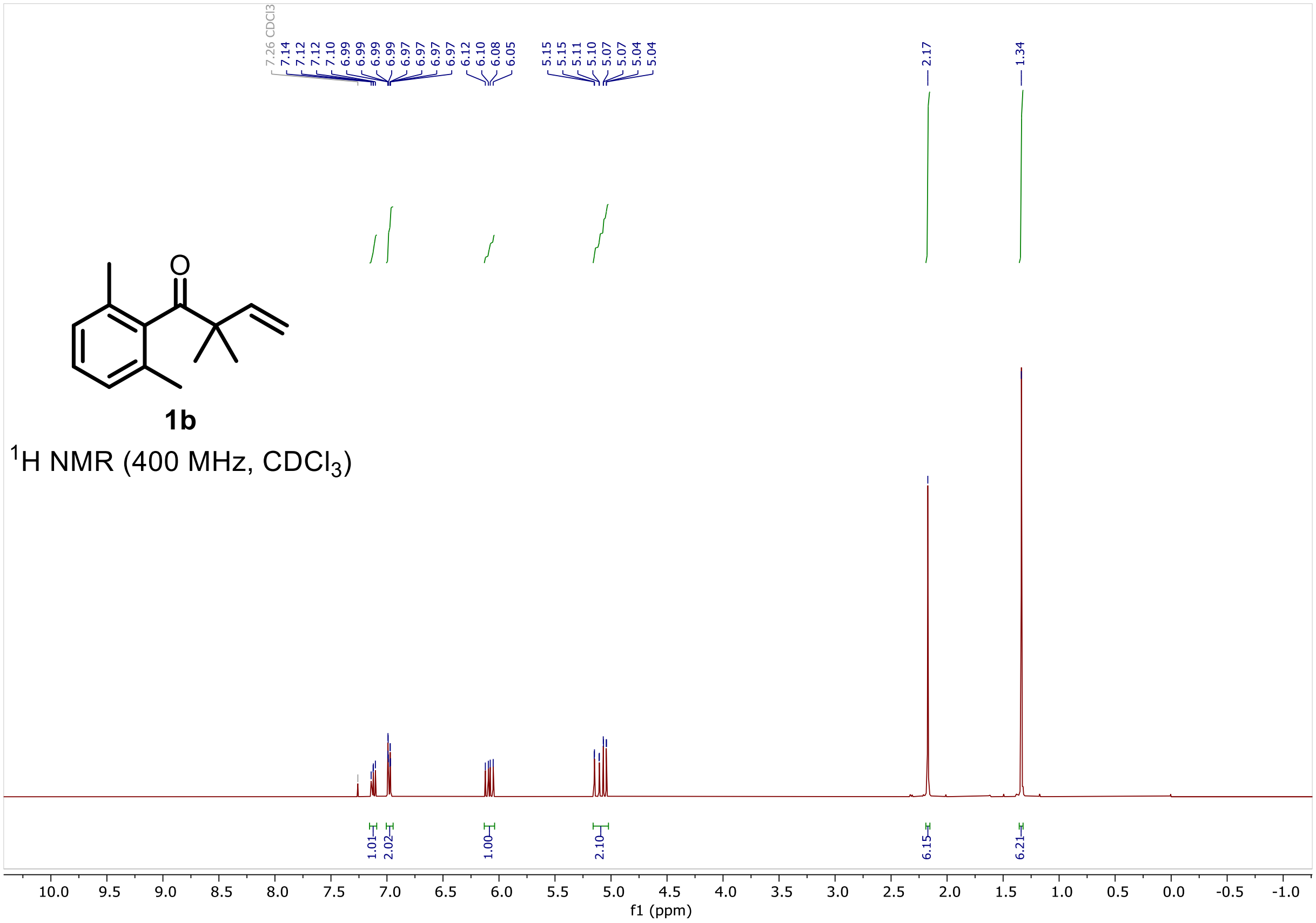

1b

^{1}H NMR (400 MHz, $CDCl_3$)

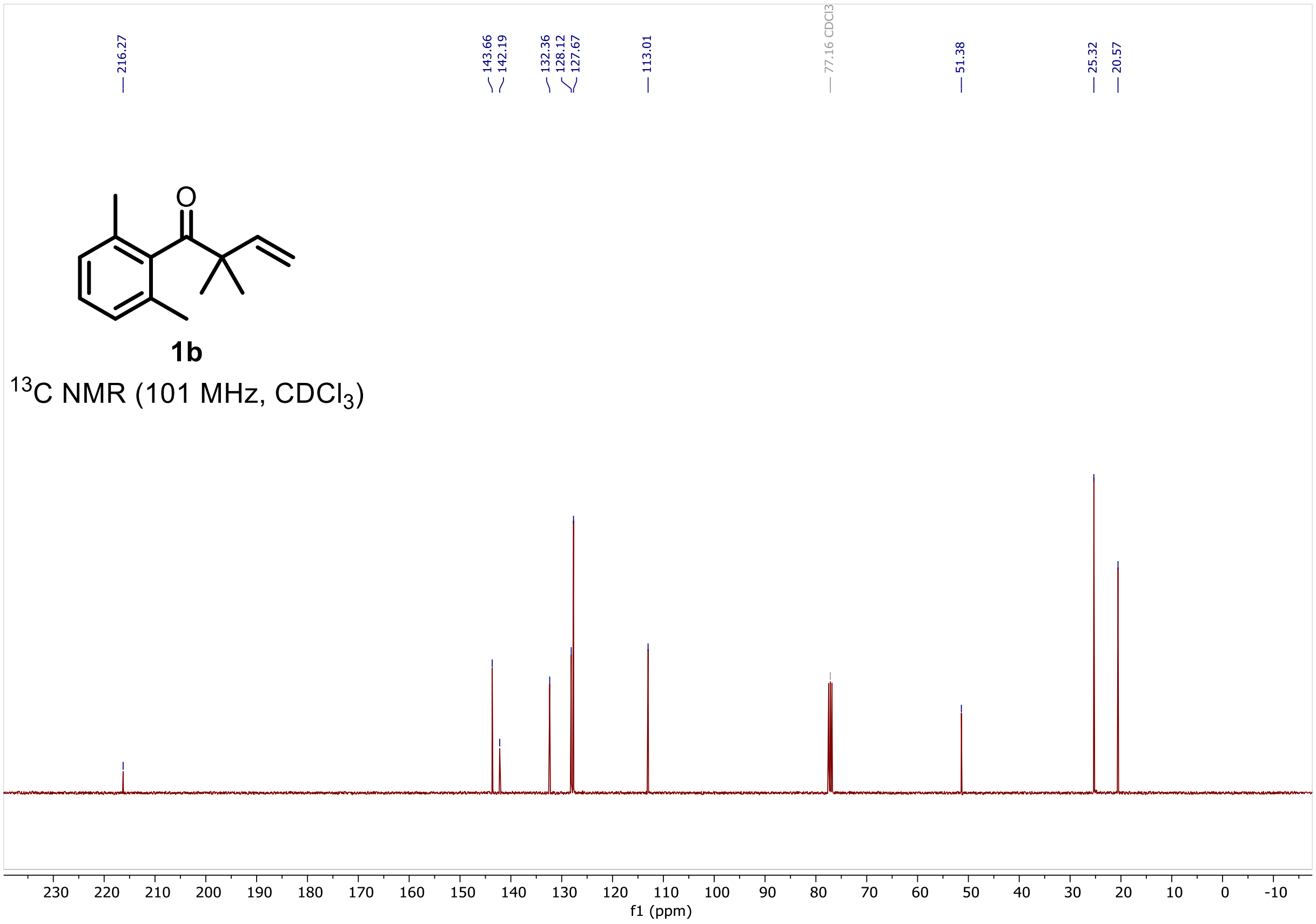

1b

^{13}C NMR (101 MHz, $CDCl_3$)

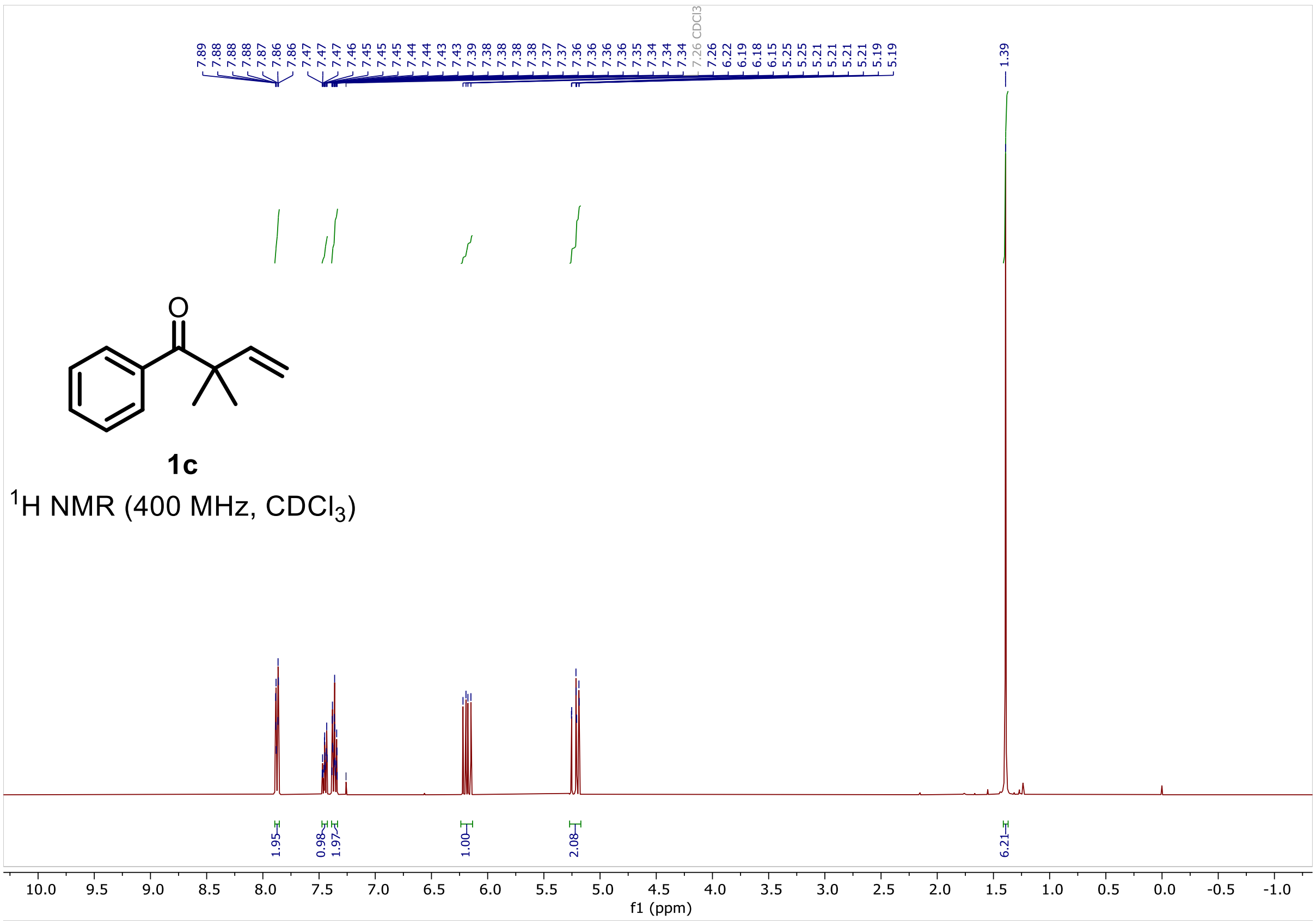
1c
^{1}H NMR (400 MHz, CDCl$_3$)
f1 (ppm)

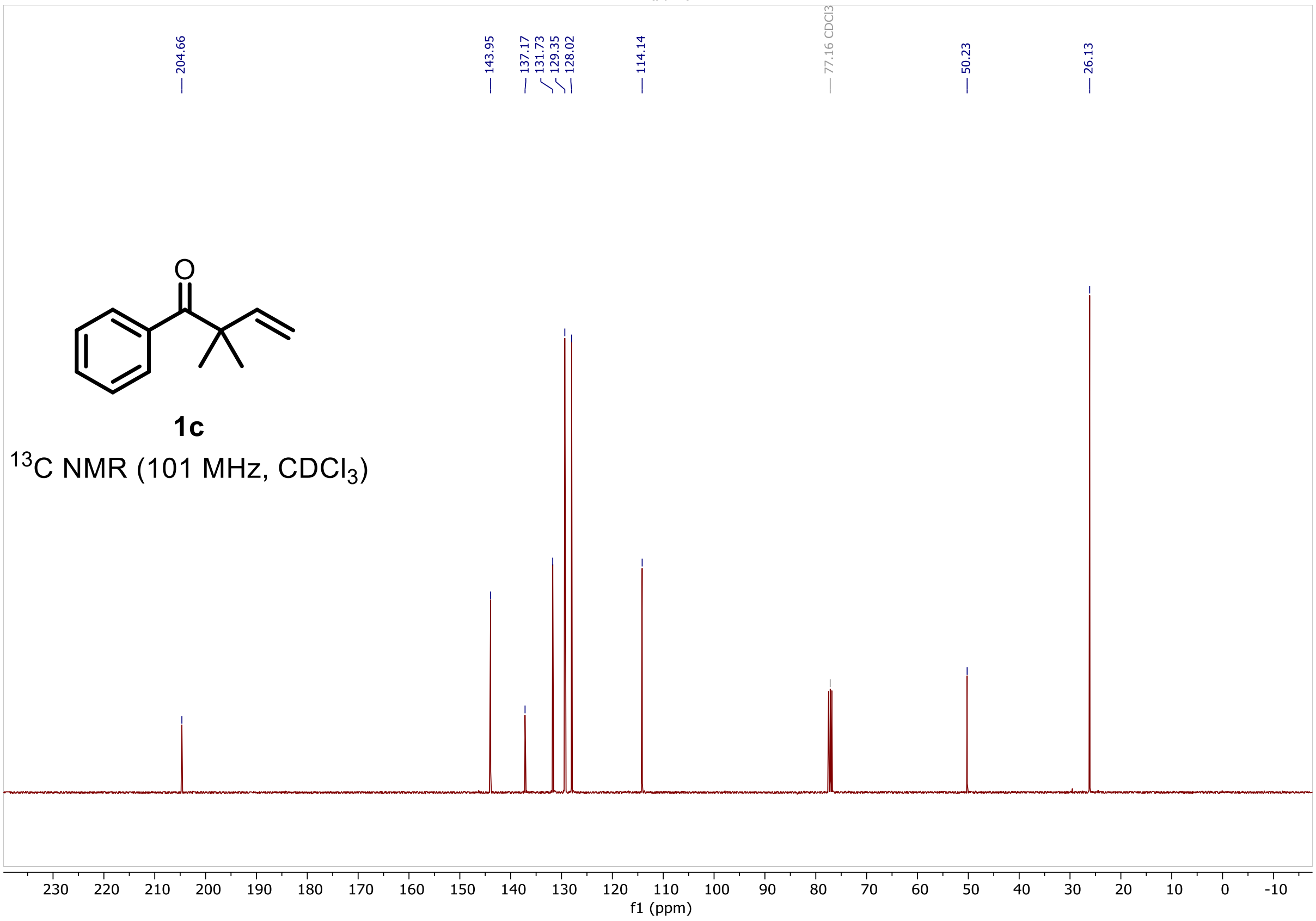
1c
^{13}C NMR (101 MHz, CDCl$_3$)
204.66
143.95
137.17
131.73
129.35
128.02
114.14
77.16 CDCl3
50.23
26.13
f1 (ppm)

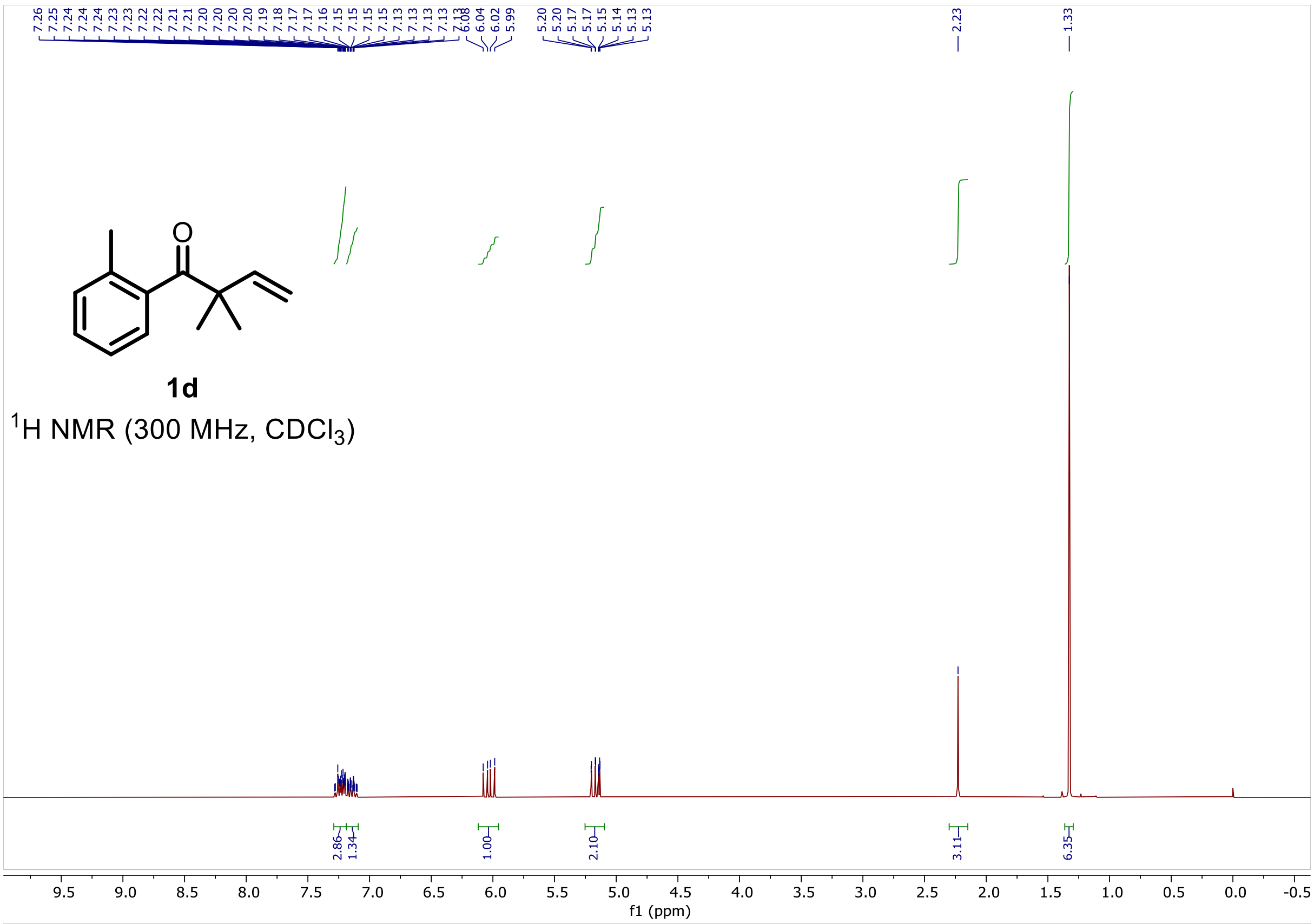
7.26 7.25 7.24 7.24 7.24 7.23 7.23 7.22 7.22 7.21 7.21 7.20 7.20 7.20 7.20 7.19 7.18 7.17 7.17 7.16 7.15 7.15 7.15 7.15 7.13 7.13 7.13 7.13 7.13 6.08 6.04 6.02 5.99
5.20 5.20 5.17 5.17 5.15 5.14 5.13 5.13
2.23
1.33
1d
1H NMR (300 MHz, CDCl3)
2.86 1.34 1.00 2.10 3.11 6.35
f1 (ppm)

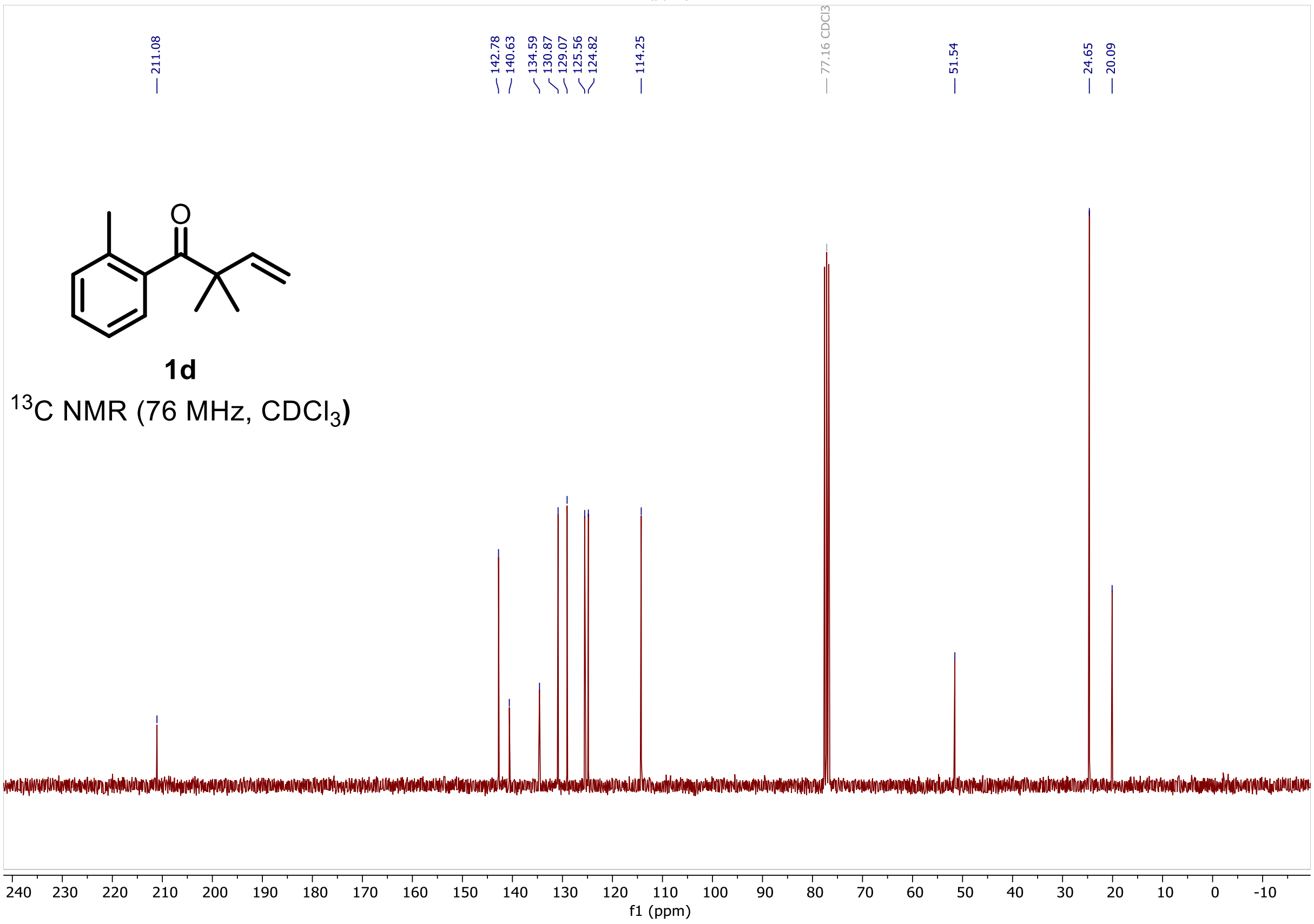
211.08
142.78 140.63 134.59 130.87 129.07 125.56 124.82
114.25
77.16 CDCl3
51.54
24.65 20.09
1d
13C NMR (76 MHz, CDCl3)
f1 (ppm)

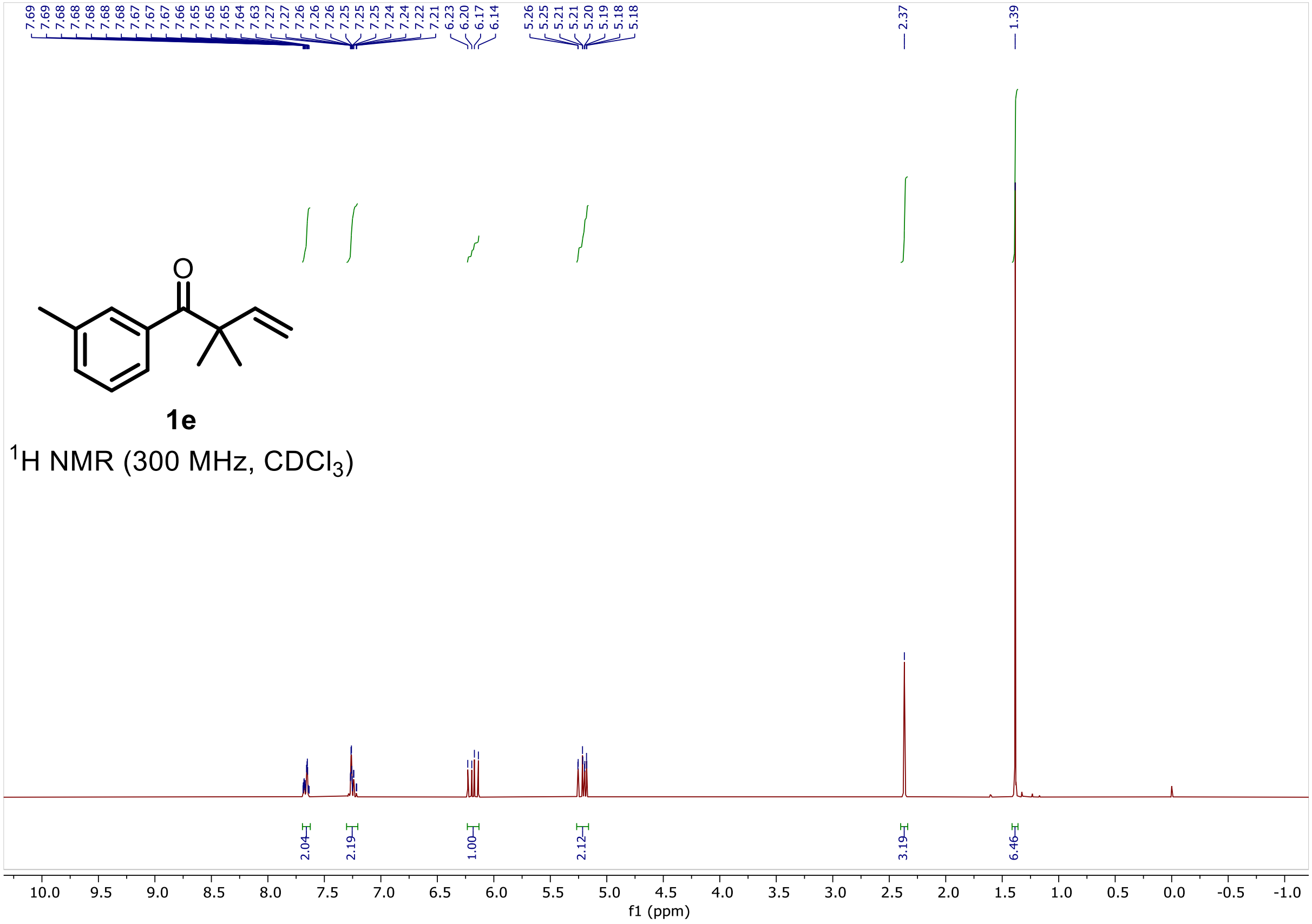
7.69 7.69 7.68 7.68 7.68 7.68 7.67 7.67 7.66 7.65 7.65 7.65 7.64 7.63 7.27 7.27 7.26 7.26 7.26 7.25 7.25 7.25 7.24 7.24 7.22 7.21 6.23 6.20 6.17 6.14 5.26 5.25 5.21 5.21 5.20 5.19 5.18 5.18 2.37 1.39
1e
^{1}H NMR (300 MHz, $CDCl_3$)
2.04 2.19 1.00 2.12 3.19 6.46
10.0 9.5 9.0 8.5 8.0 7.5 7.0 6.5 6.0 5.5 5.0 4.5 4.0 3.5 3.0 2.5 2.0 1.5 1.0 0.5 0.0 -0.5 -1.0
f1 (ppm)

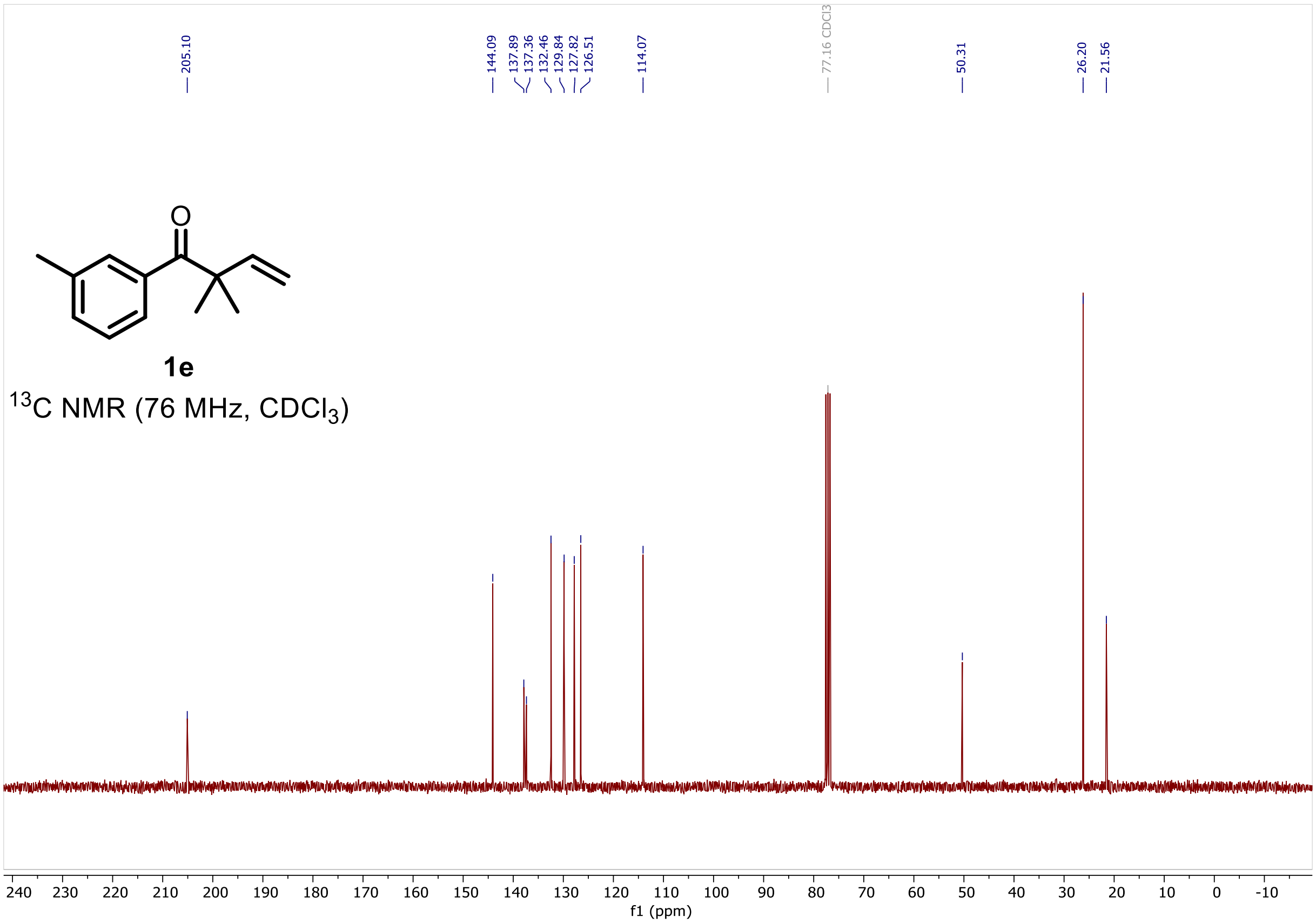
205.10 144.09 137.89 137.36 132.46 129.84 127.82 126.51 114.07 77.16 CDCl3 50.31 26.20 21.56
1e
^{13}C NMR (76 MHz, $CDCl_3$)
240 230 220 210 200 190 180 170 160 150 140 130 120 110 100 90 80 70 60 50 40 30 20 10 0 -10
f1 (ppm)

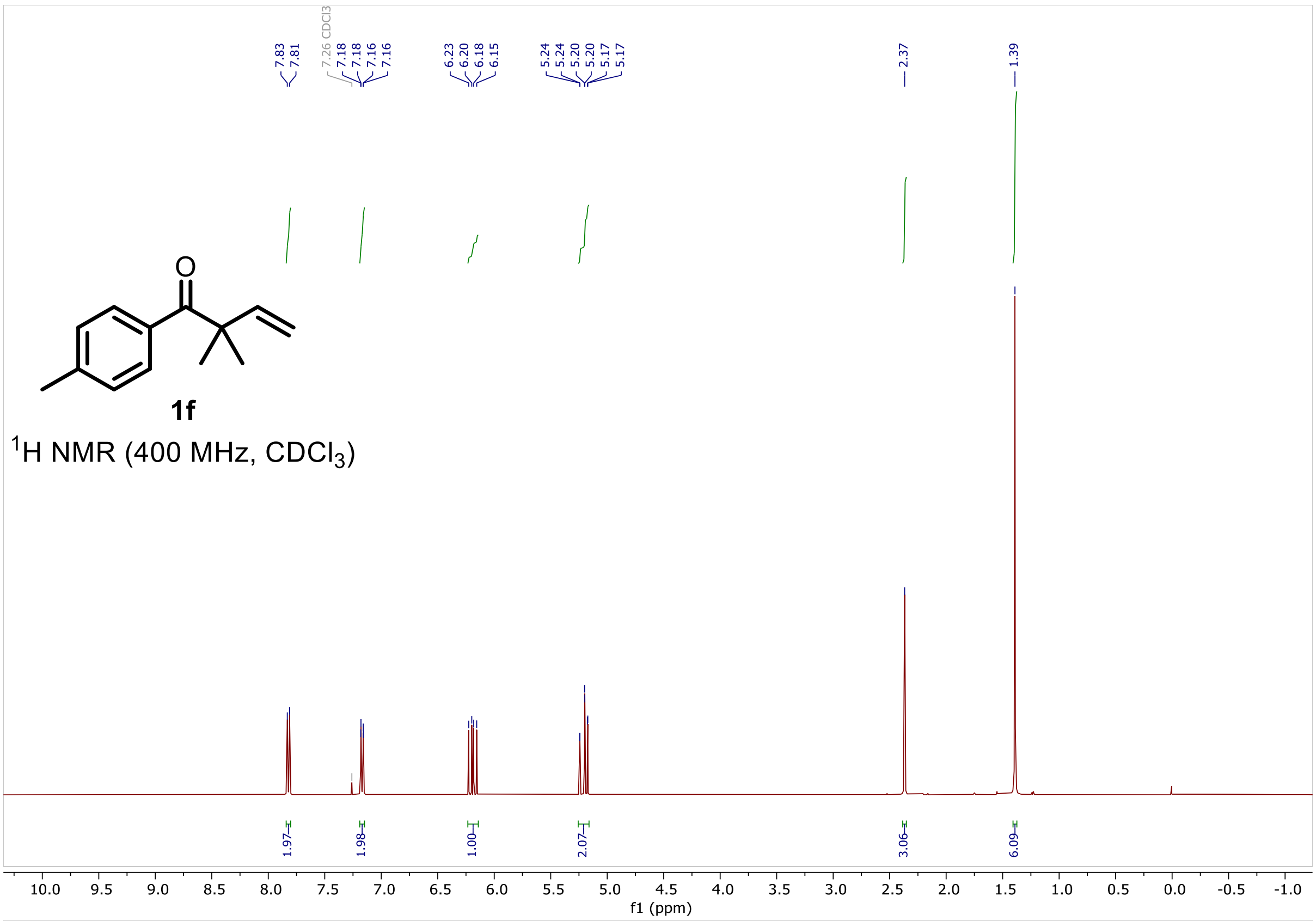

1f
^{1}H NMR (400 MHz, CDCl$_3$)
f1 (ppm)

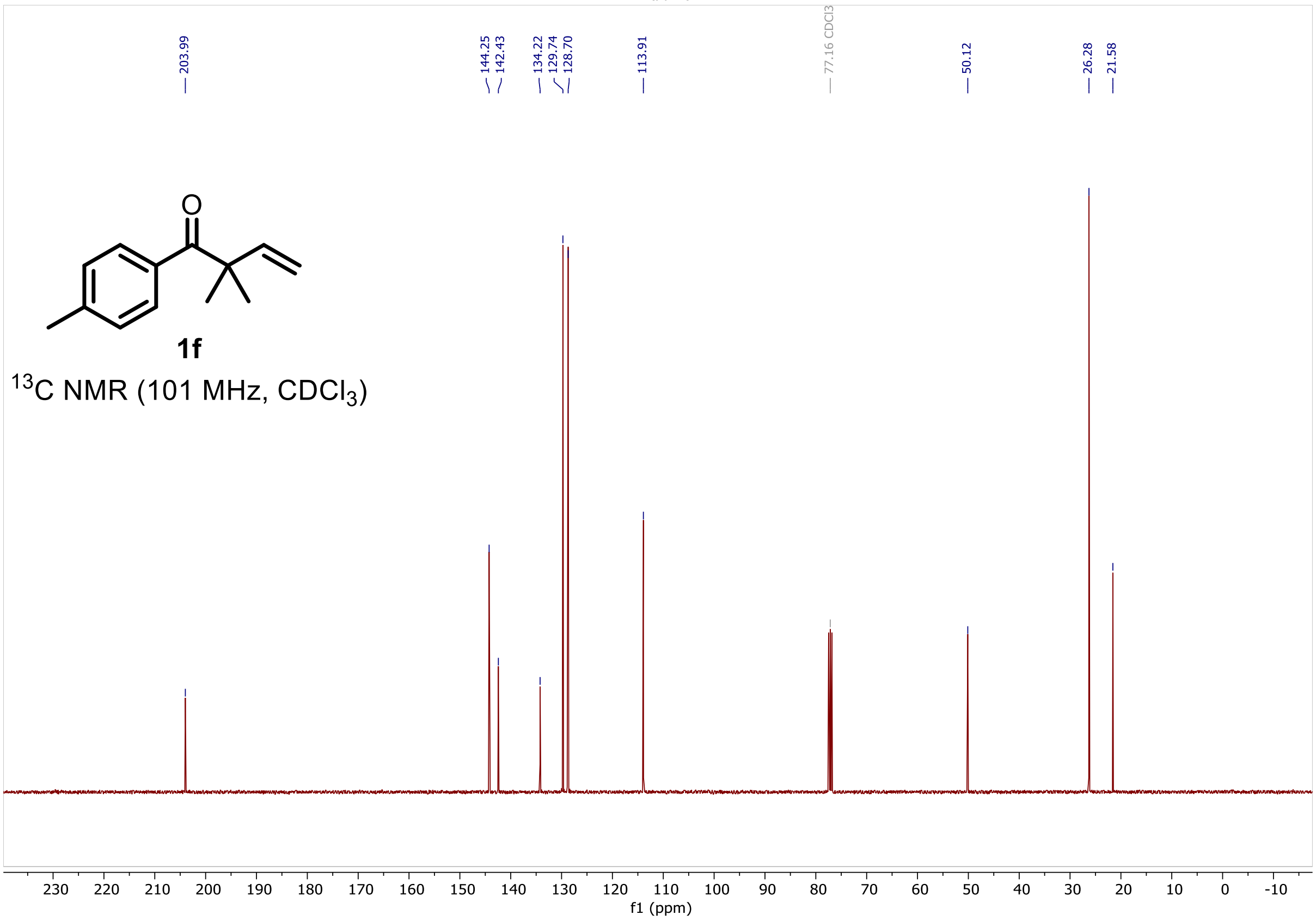

1f
^{13}C NMR (101 MHz, CDCl$_3$)
f1 (ppm)

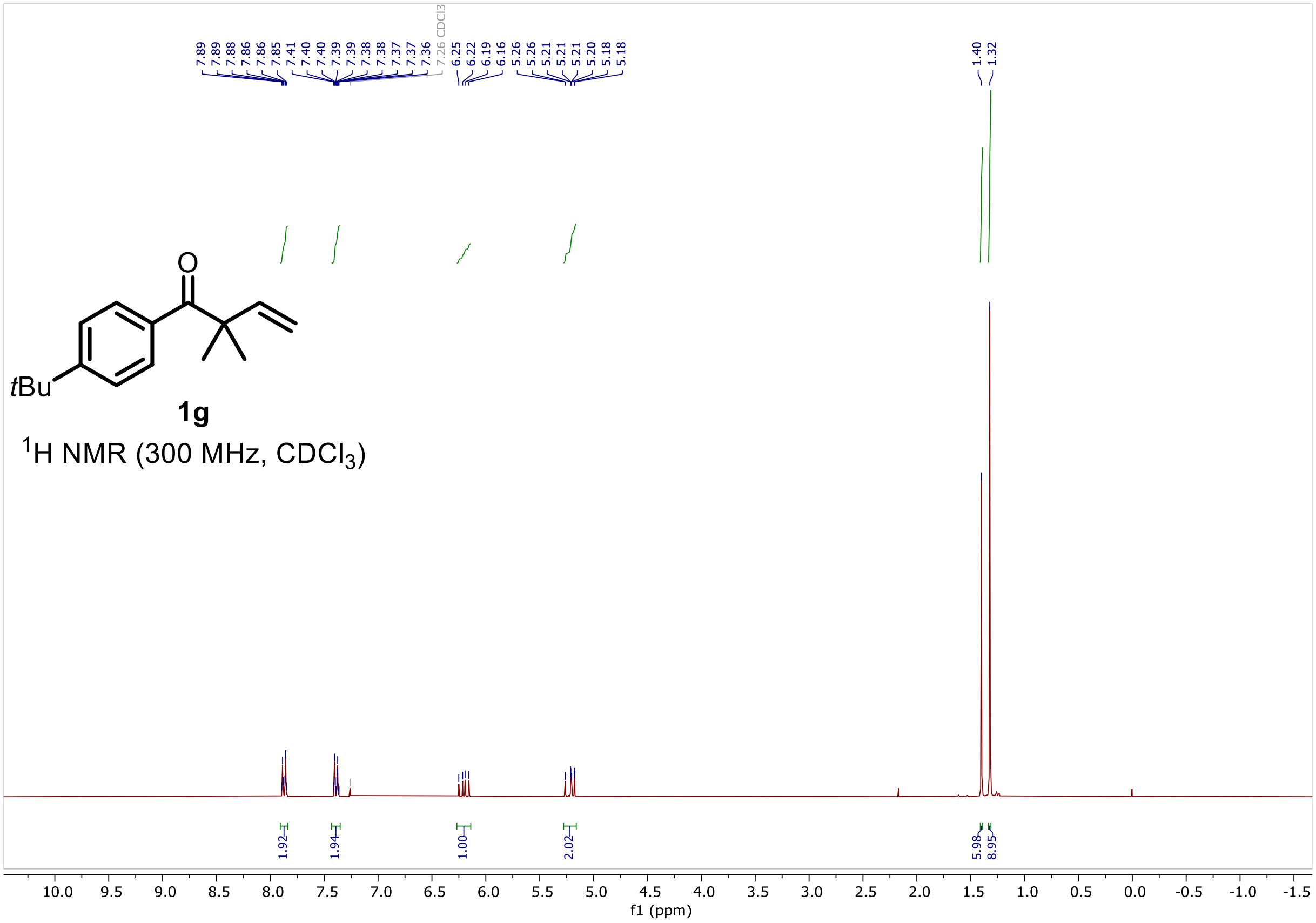
tBu
O
1g
1H NMR (300 MHz, CDCl3)
7.89 7.89 7.88 7.86 7.86 7.85 7.41 7.40 7.40 7.39 7.39 7.38 7.38 7.37 7.37 7.36 7.26 CDCl3 6.25 6.22 6.19 6.16 5.26 5.26 5.21 5.21 5.21 5.20 5.18 5.18
1.40 1.32
1.92 1.94 1.00 2.02 5.98 8.95
f1 (ppm)

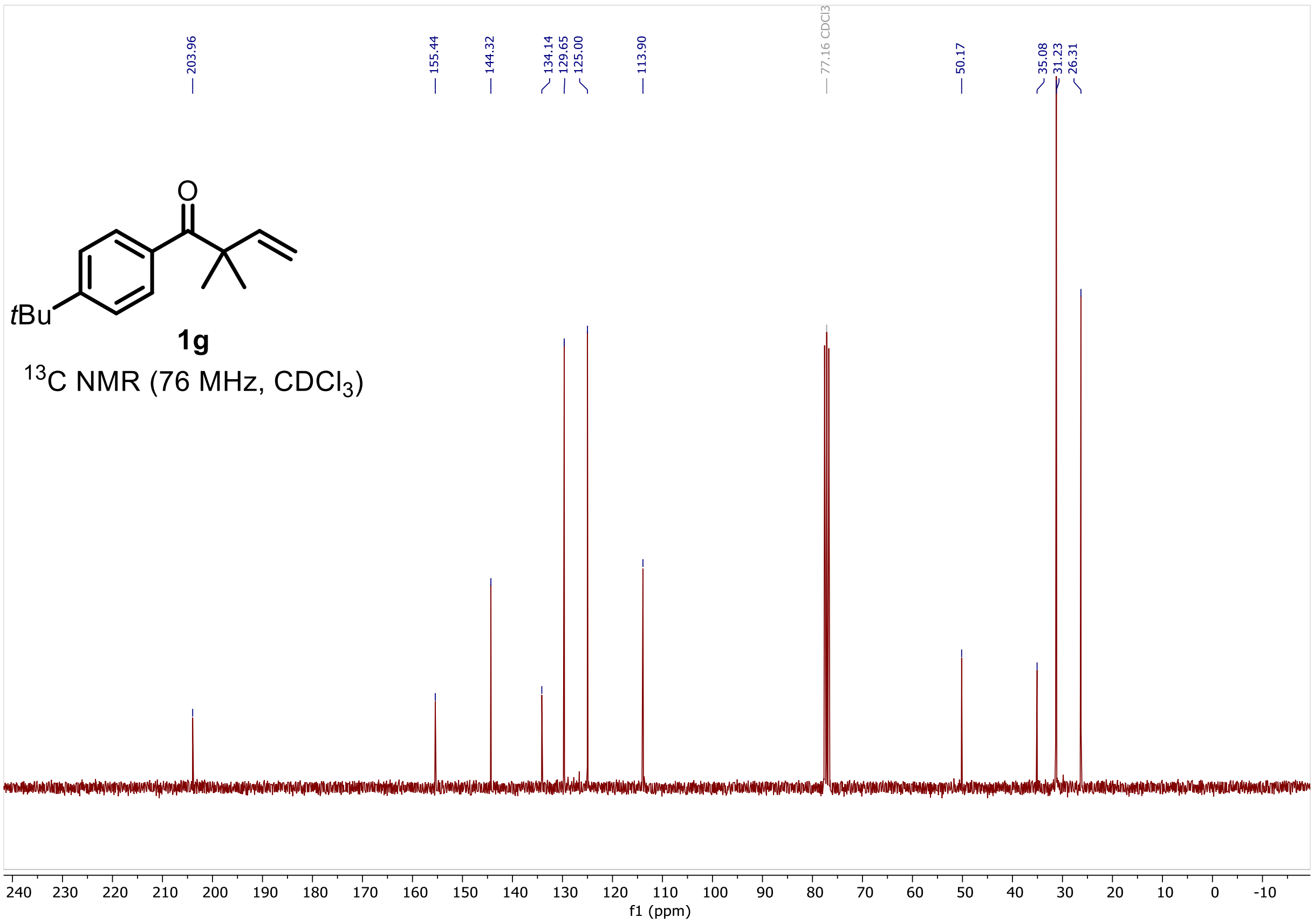
tBu
O
1g
13C NMR (76 MHz, CDCl3)
203.96 155.44 144.32 134.14 129.65 125.00 113.90 77.16 CDCl3 50.17 35.08 31.23 26.31
f1 (ppm)

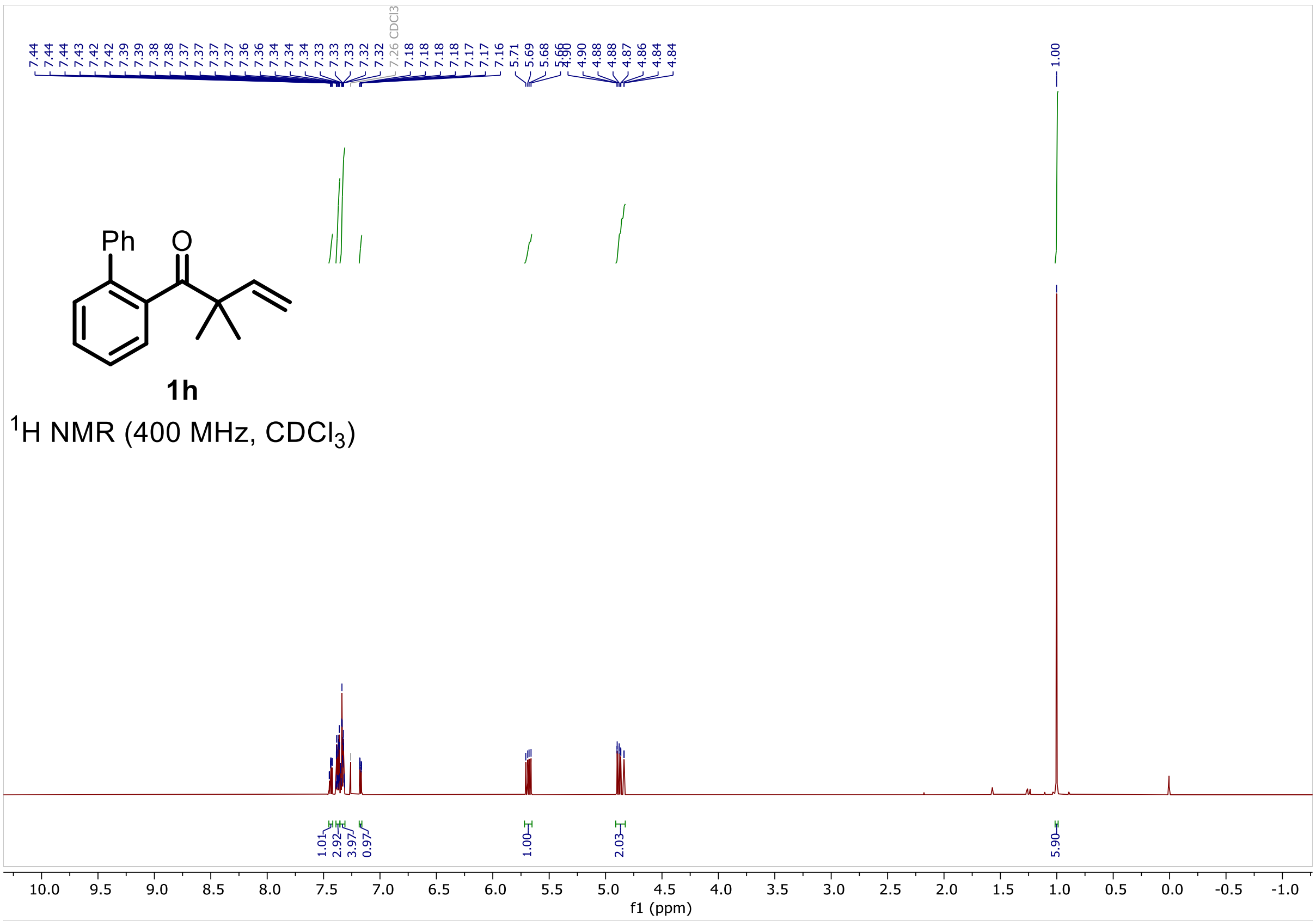

1h

^{1}H NMR (400 MHz, $CDCl_3$)

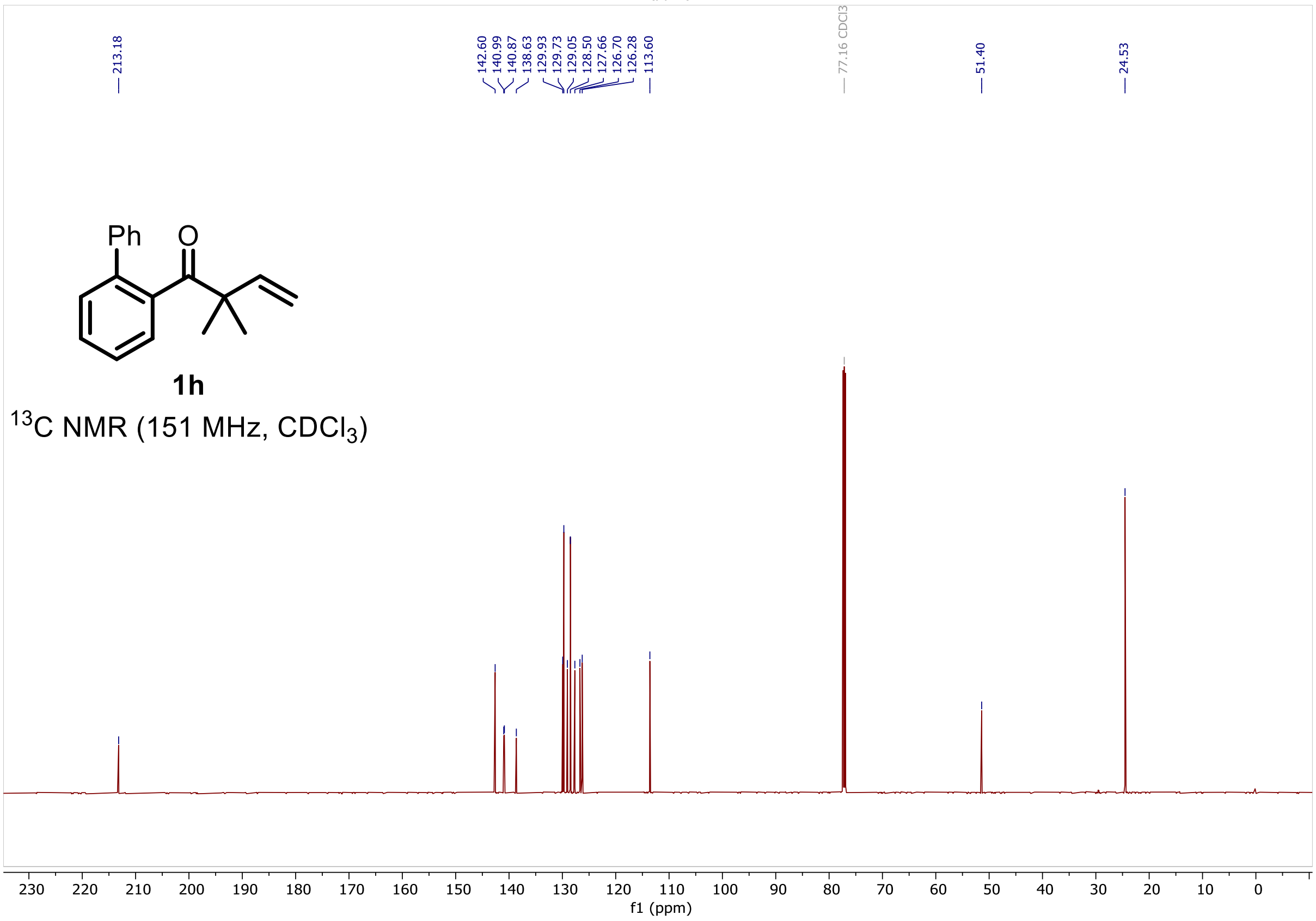

1h

^{13}C NMR (151 MHz, $CDCl_3$)

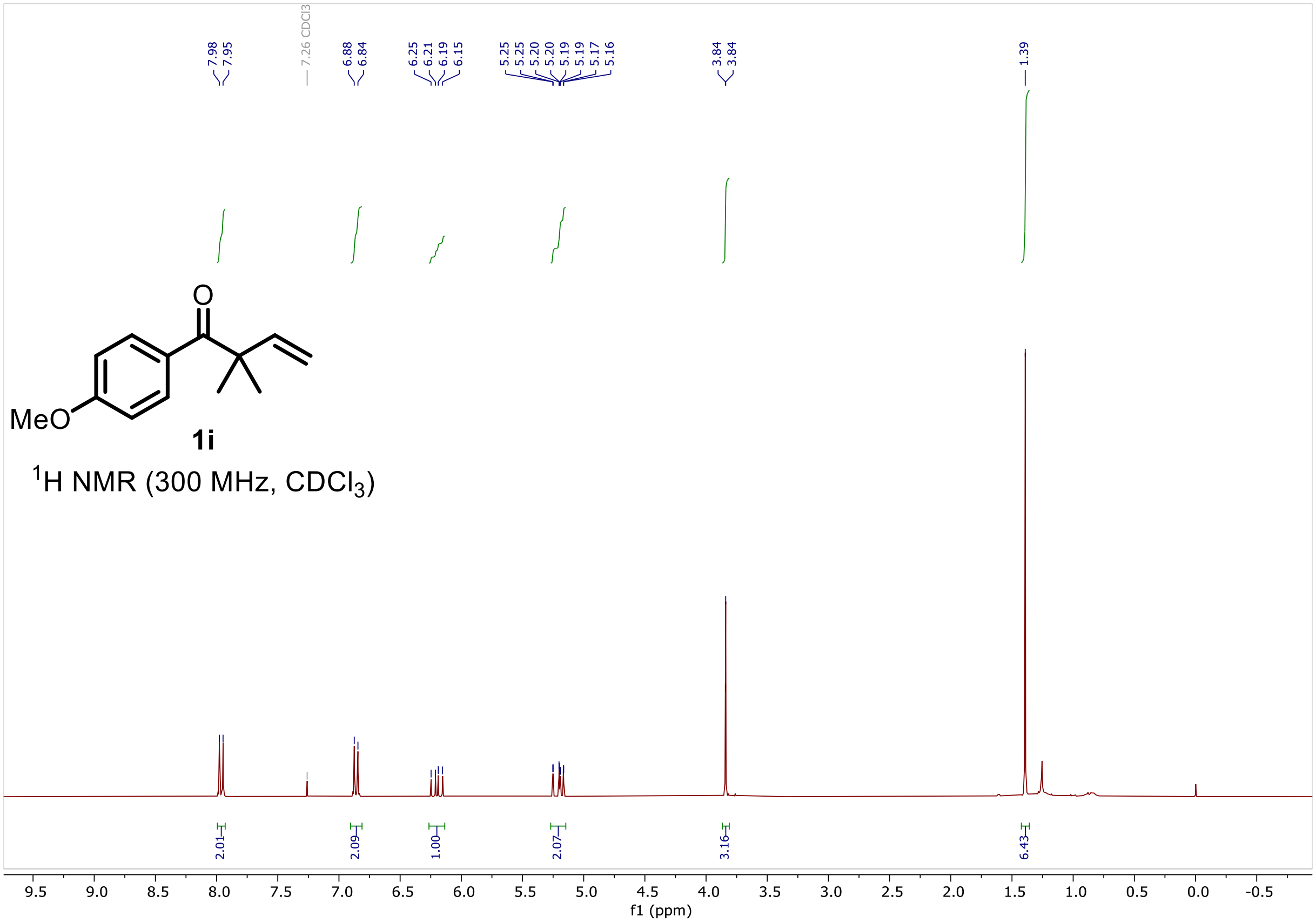

7.98
7.95
7.26 CDCl3
6.88
6.84
6.25
6.21
6.19
6.15
5.25
5.25
5.20
5.20
5.19
5.19
5.17
5.16
3.84
3.84
1.39
MeO
O
1i
^{1}H NMR (300 MHz, $CDCl_3$)
2.01
2.09
1.00
2.07
3.16
6.43
9.5 9.0 8.5 8.0 7.5 7.0 6.5 6.0 5.5 5.0 4.5 4.0 3.5 3.0 2.5 2.0 1.5 1.0 0.5 0.0 -0.5
f1 (ppm)

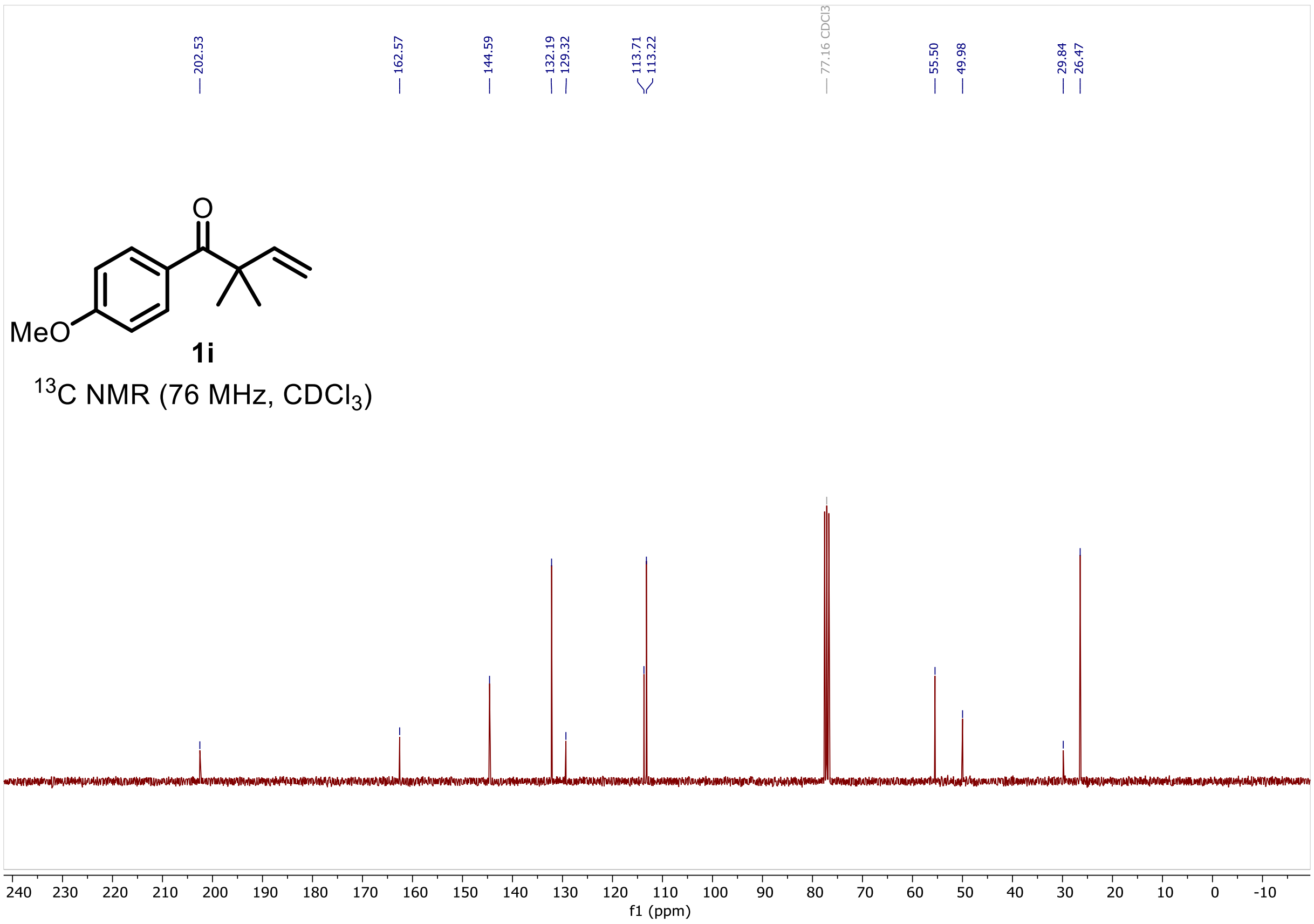

202.53
162.57
144.59
132.19
129.32
113.71
113.22
77.16 CDCl3
55.50
49.98
29.84
26.47
MeO
O
1i
^{13}C NMR (76 MHz, $CDCl_3$)
240 230 220 210 200 190 180 170 160 150 140 130 120 110 100 90 80 70 60 50 40 30 20 10 0 -10
f1 (ppm)

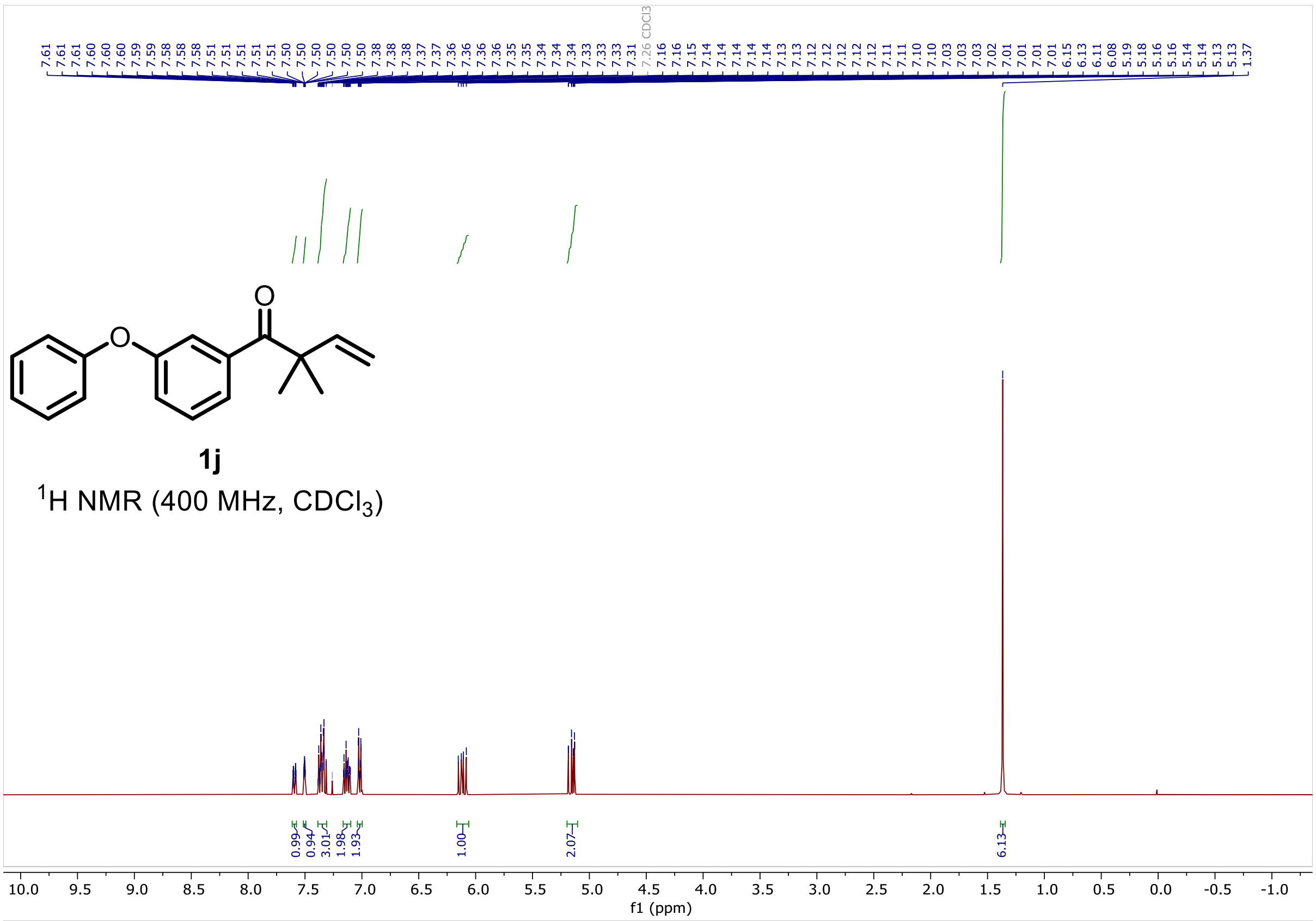
1j
¹H NMR (400 MHz, CDCl3)
7.26 CDCl3
0.99
0.94
3.01
1.98
1.93
1.00
2.07
6.13
f1 (ppm)

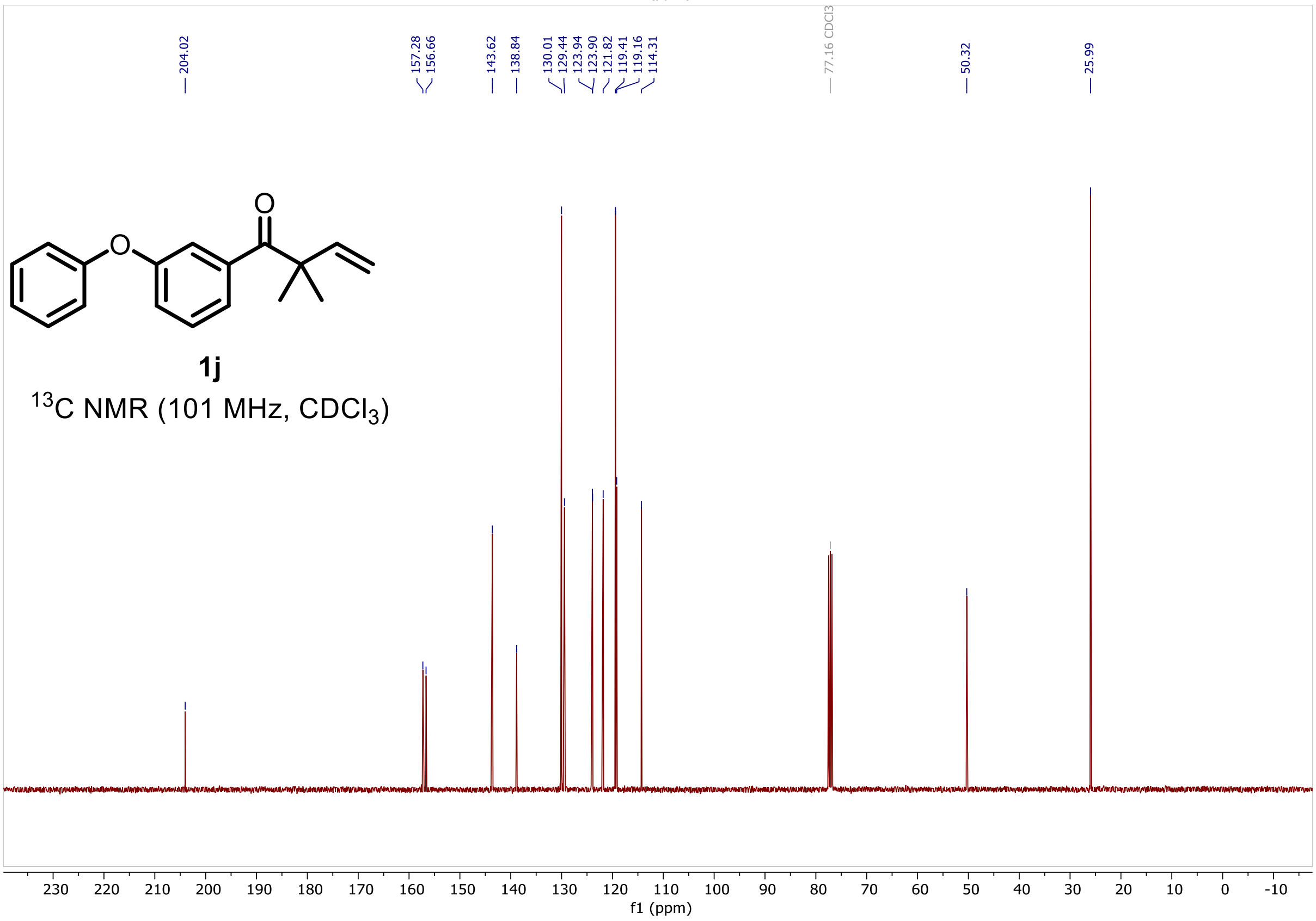
1j
¹³C NMR (101 MHz, CDCl3)
204.02
157.28
156.66
143.62
138.84
130.01
129.44
123.94
123.90
121.82
119.41
119.16
114.31
77.16 CDCl3
50.32
25.99
f1 (ppm)

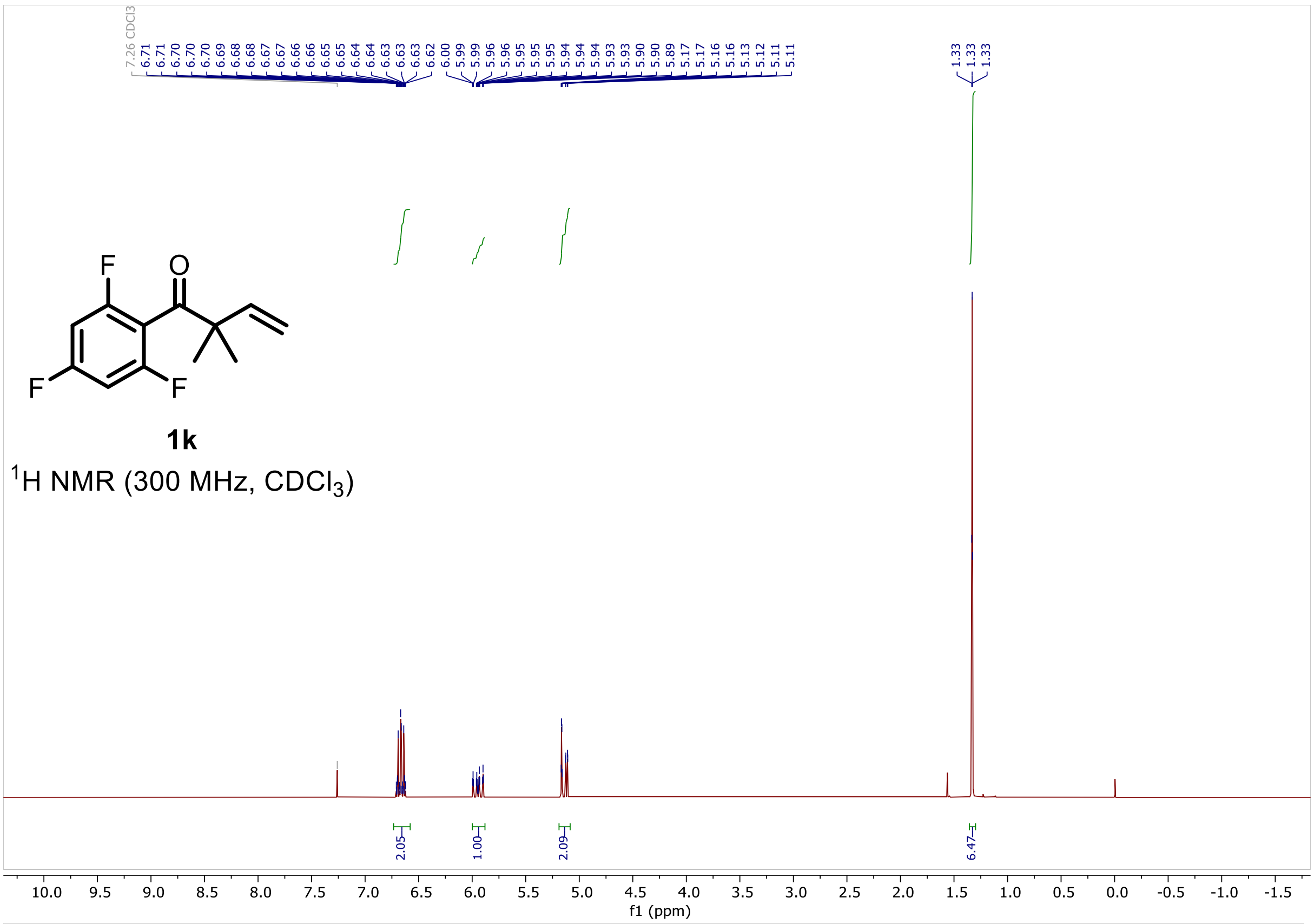
F
O
F
F
1k
1H NMR (300 MHz, CDCl3)
7.26 CDCl3
6.71
6.71
6.70
6.70
6.69
6.68
6.67
6.67
6.66
6.66
6.65
6.65
6.64
6.64
6.63
6.63
6.63
6.62
6.00
5.99
5.99
5.96
5.96
5.95
5.95
5.95
5.94
5.94
5.94
5.93
5.93
5.90
5.90
5.89
5.17
5.17
5.16
5.16
5.13
5.12
5.11
5.11
1.33
1.33
1.33
2.05
1.00
2.09
6.47
f1 (ppm)

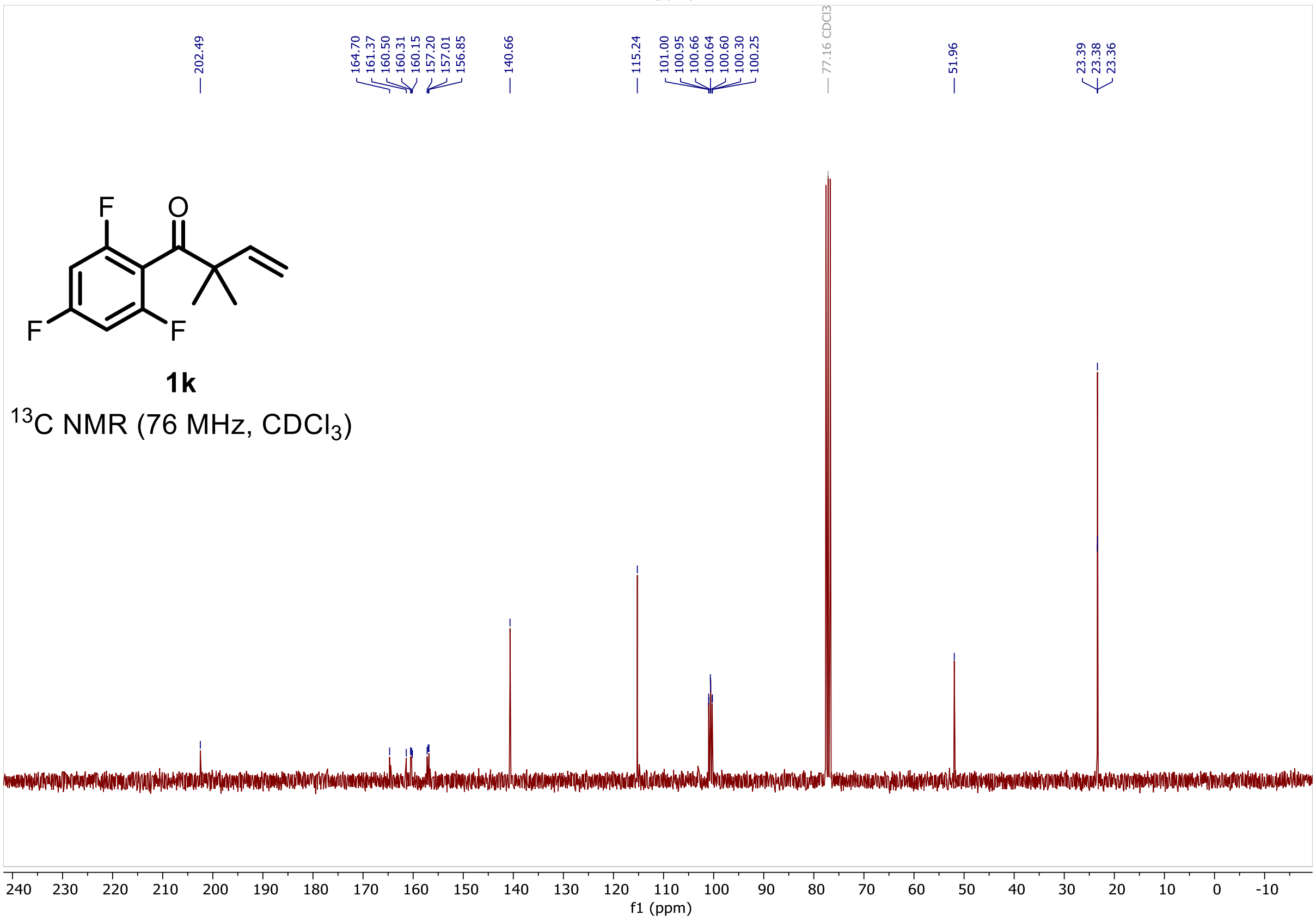
F
O
F
F
1k
13C NMR (76 MHz, CDCl3)
202.49
164.70
161.37
160.50
160.31
160.15
157.20
157.01
156.85
140.66
115.24
101.00
100.95
100.66
100.64
100.60
100.30
100.25
77.16 CDCl3
51.96
23.39
23.38
23.36
f1 (ppm)

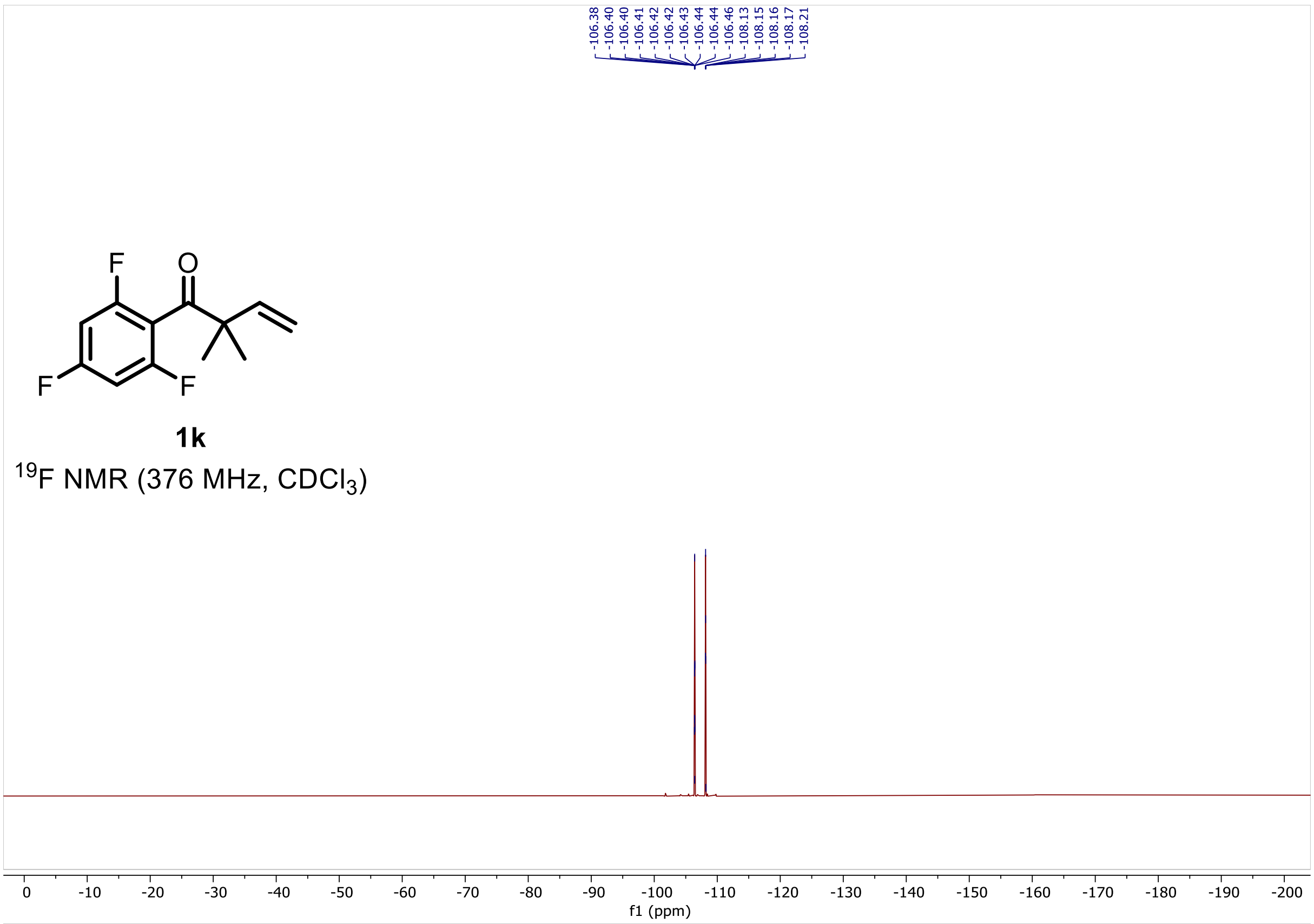
-106.38
-106.40
-106.40
-106.41
-106.42
-106.42
-106.43
-106.44
-106.44
-106.46
-108.13
-108.15
-108.16
-108.17
-108.21
F
O
F
F
1k
19F NMR (376 MHz, CDCl3)
0 -10 -20 -30 -40 -50 -60 -70 -80 -90 -100 -110 -120 -130 -140 -150 -160 -170 -180 -190 -200
f1 (ppm)

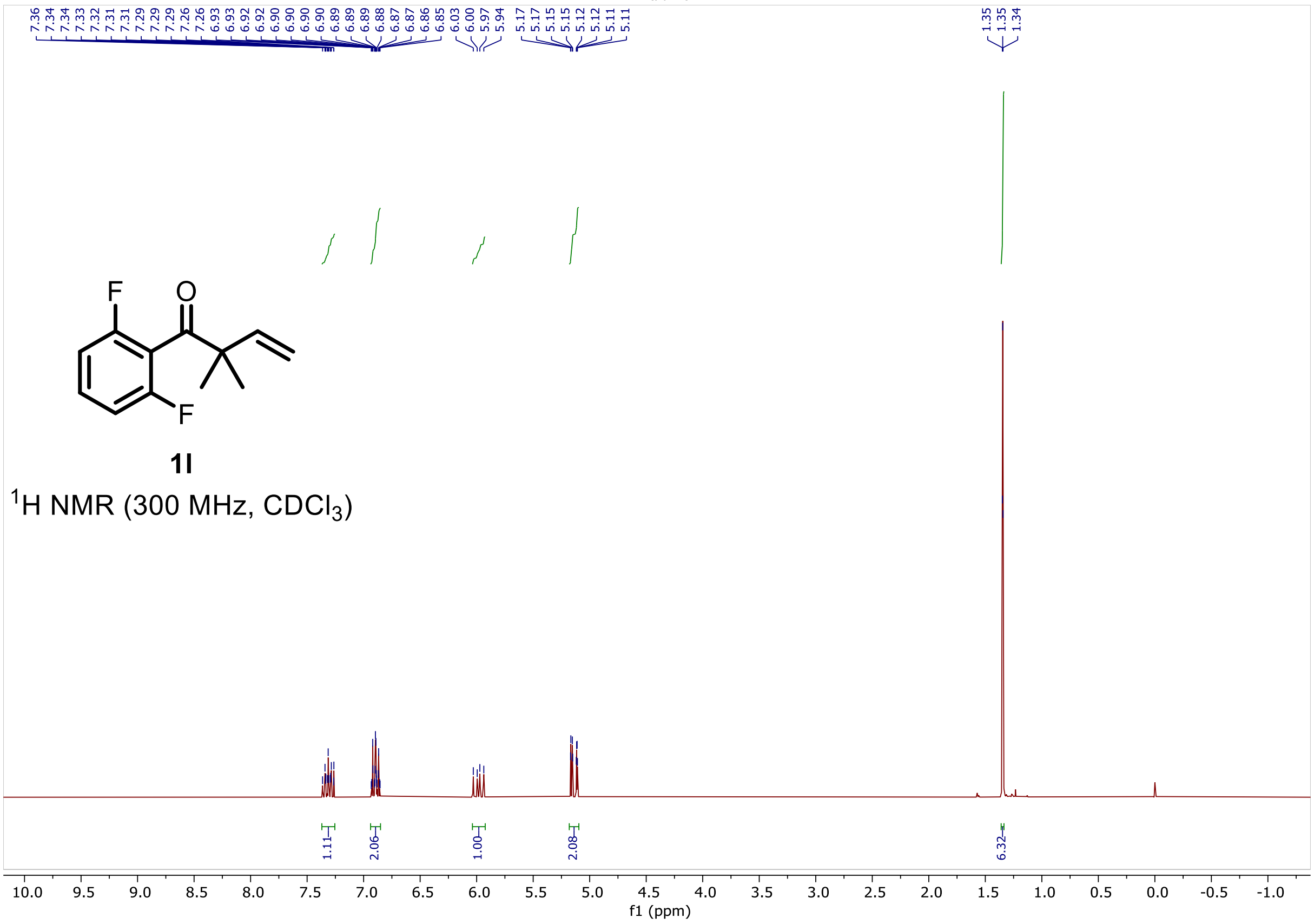
7.36
7.34
7.34
7.33
7.32
7.31
7.31
7.29
7.29
7.29
7.26
6.93
6.93
6.92
6.92
6.90
6.90
6.90
6.90
6.89
6.89
6.89
6.88
6.87
6.87
6.86
6.85
6.03
6.00
5.97
5.94
5.17
5.17
5.15
5.15
5.12
5.12
5.11
5.11
1.35
1.35
1.34
F
O
F
1l
1H NMR (300 MHz, CDCl3)
1.11
2.06
1.00
2.08
6.32
10.0 9.5 9.0 8.5 8.0 7.5 7.0 6.5 6.0 5.5 5.0 4.5 4.0 3.5 3.0 2.5 2.0 1.5 1.0 0.5 0.0 -0.5 -1.0
f1 (ppm)

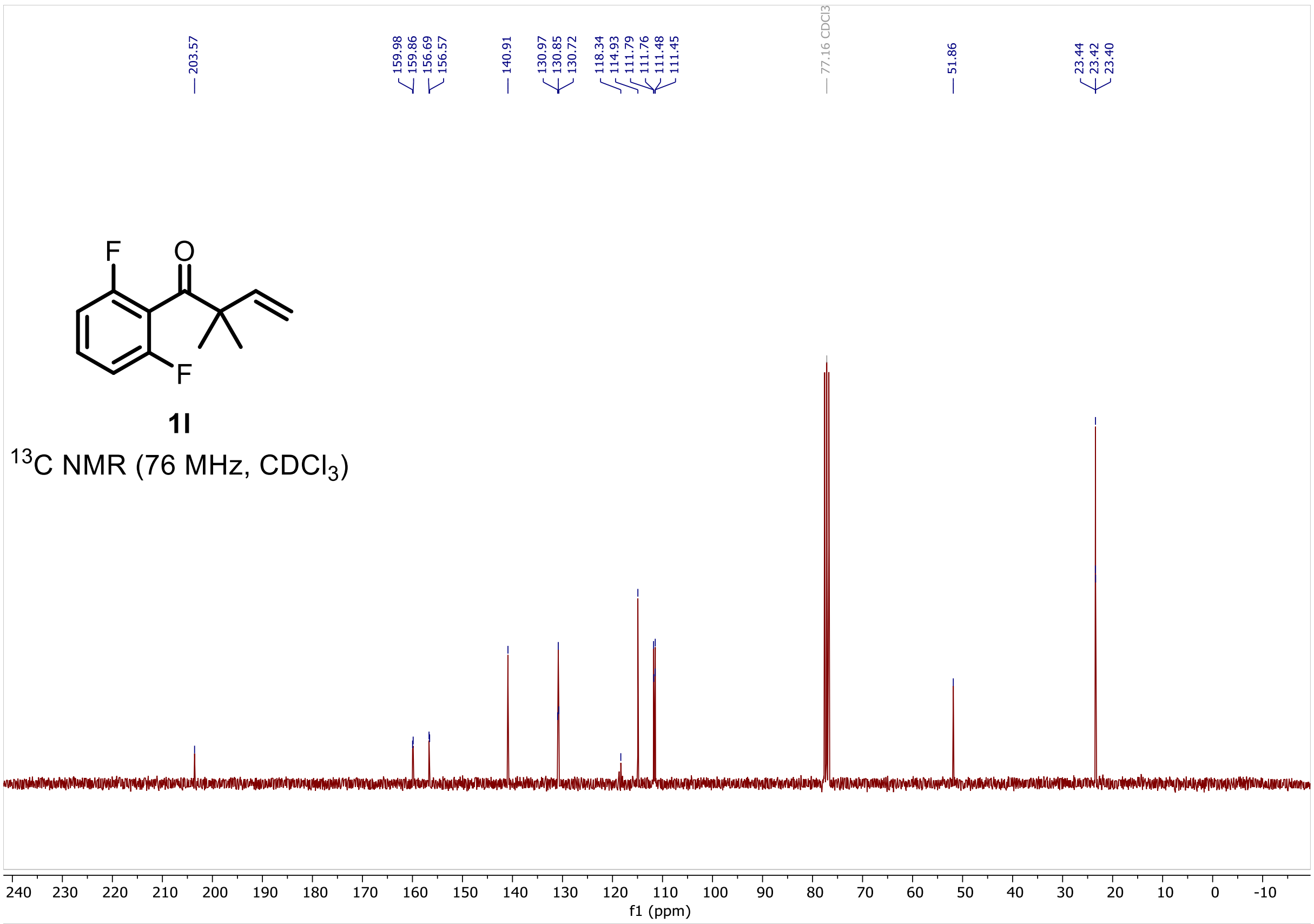
203.57
159.98
159.86
156.69
156.57
140.91
130.97
130.85
130.72
118.34
114.93
111.79
111.76
111.48
111.45
77.16 CDCl3
51.86
23.44
23.42
23.40
F
O
F
1l
13C NMR (76 MHz, CDCl3)
240 230 220 210 200 190 180 170 160 150 140 130 120 110 100 90 80 70 60 50 40 30 20 10 0 -10
f1 (ppm)

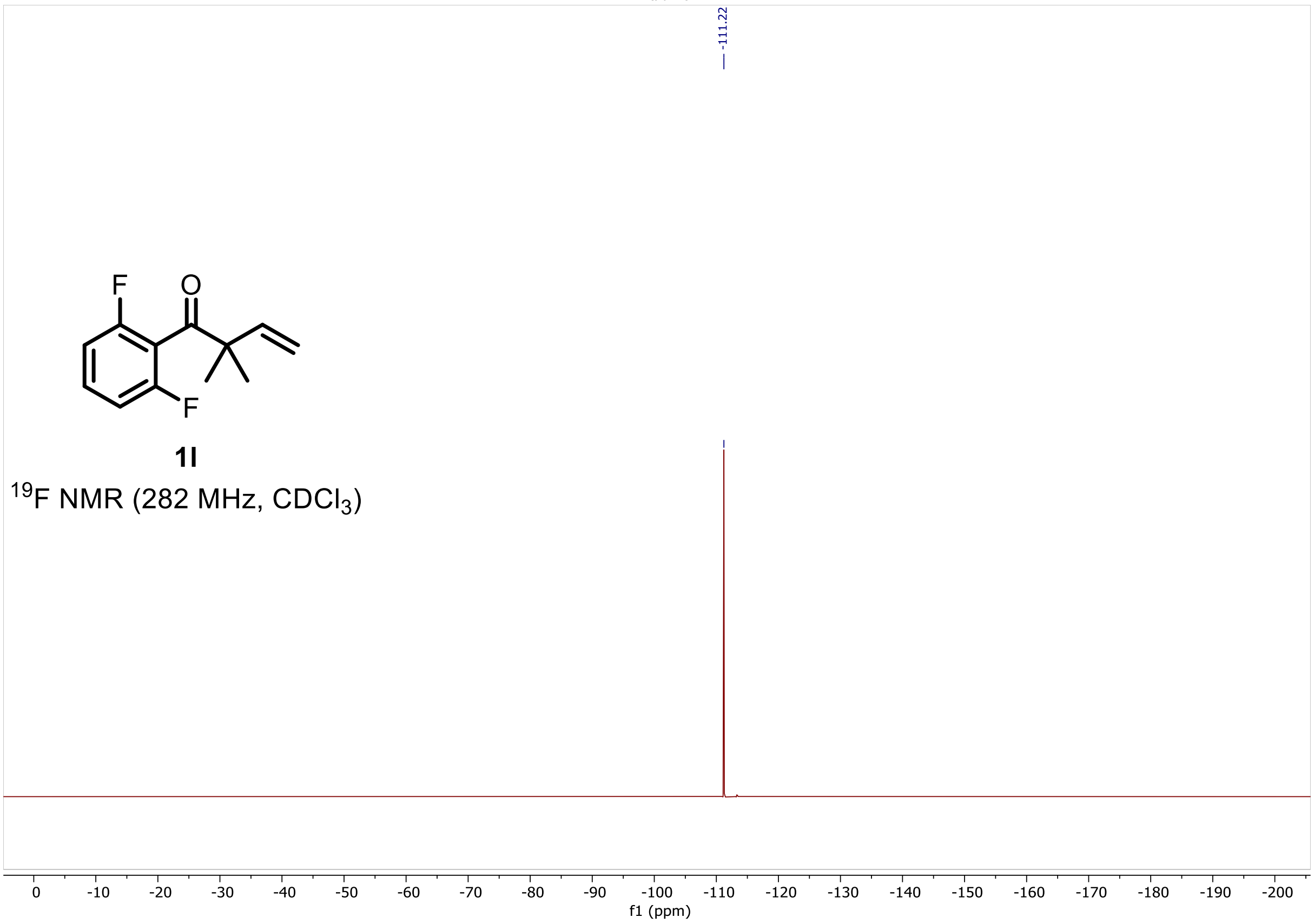
-111.22
F
O
F
1l
19F NMR (282 MHz, CDCl3)
0 -10 -20 -30 -40 -50 -60 -70 -80 -90 -100 -110 -120 -130 -140 -150 -160 -170 -180 -190 -200
f1 (ppm)

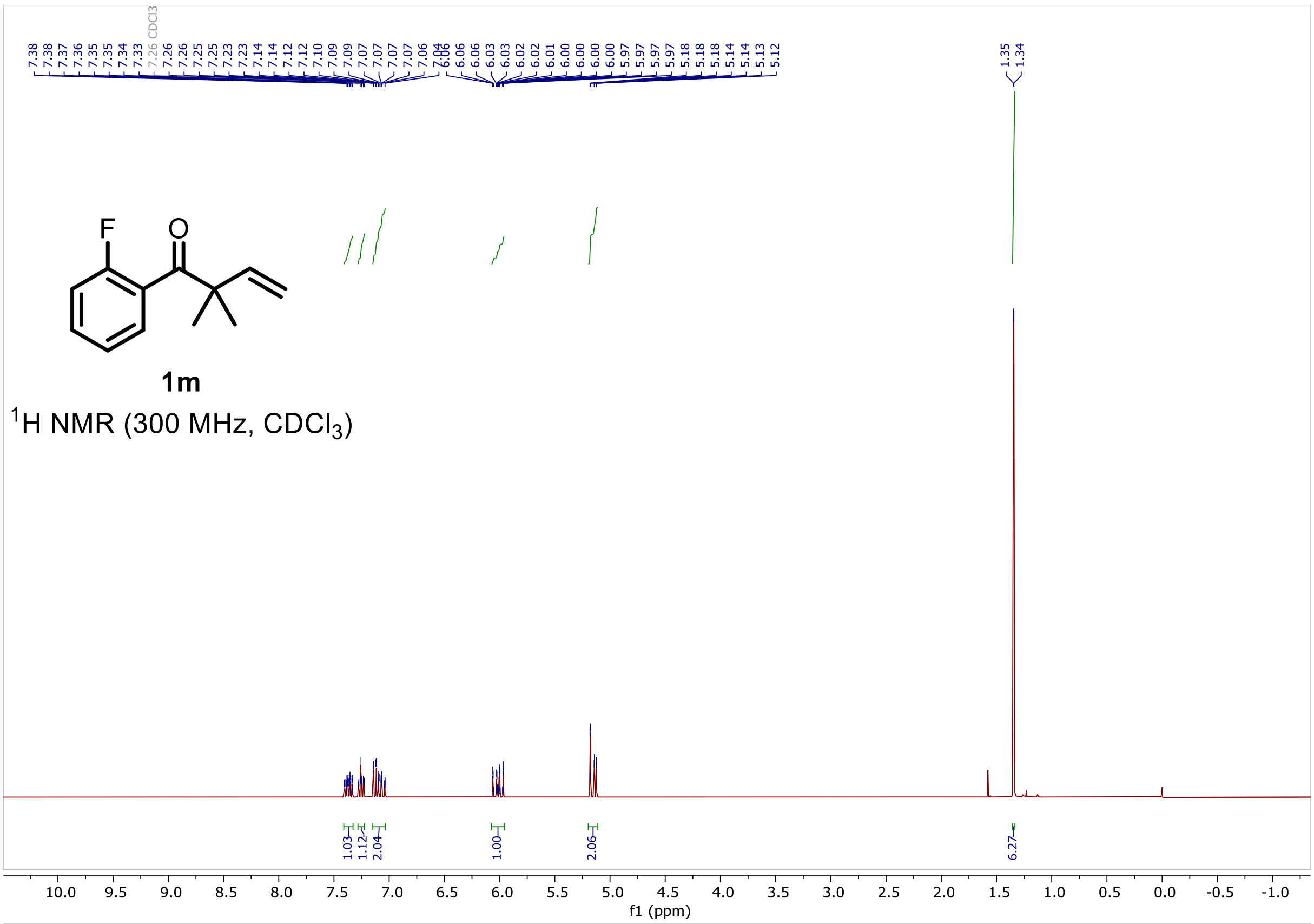
F
O
1m
^{1}H NMR (300 MHz, $CDCl_3$)
f1 (ppm)

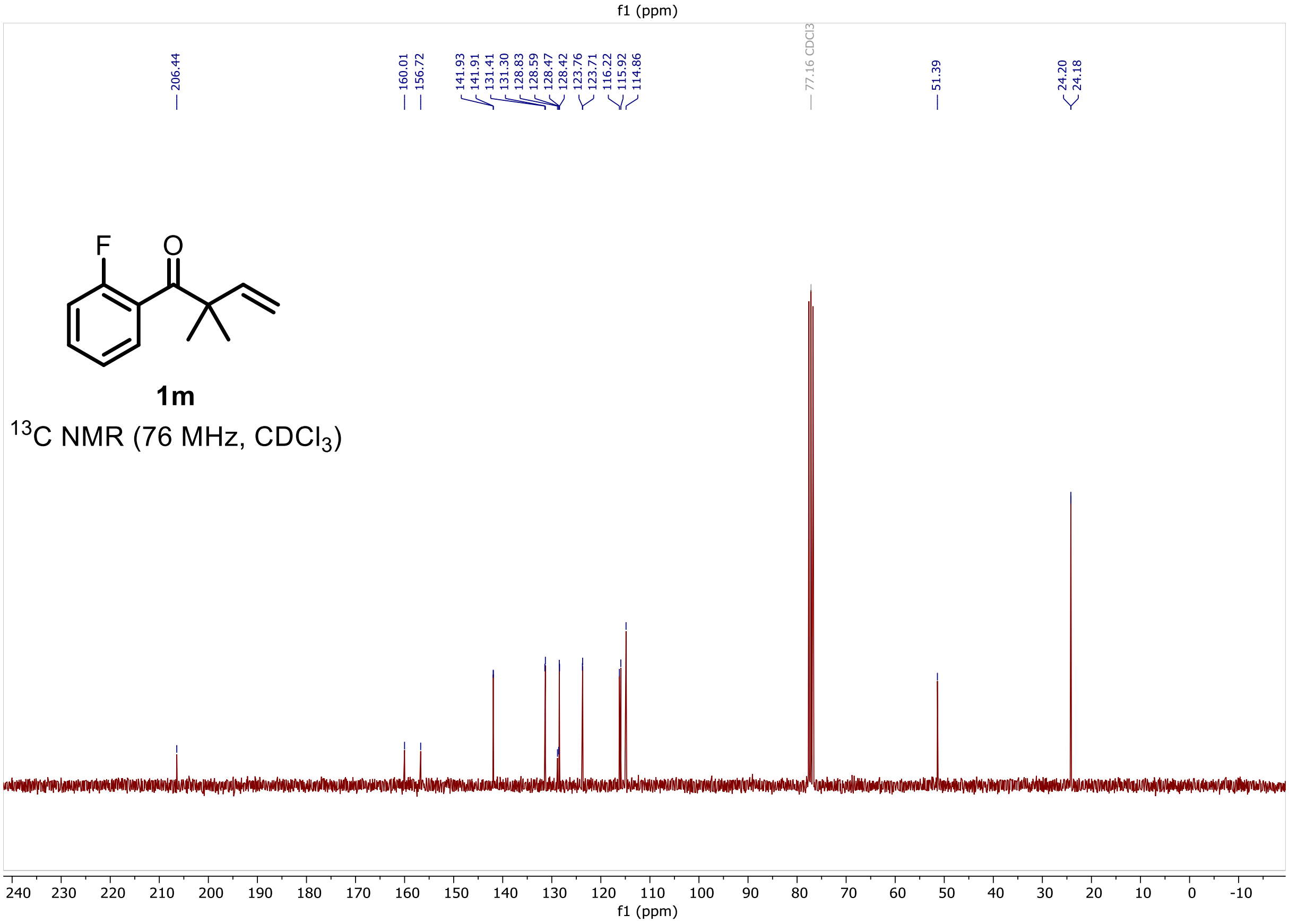
F
O
1m
^{13}C NMR (76 MHz, $CDCl_3$)
206.44
160.01
156.72
141.93
141.91
131.41
131.30
128.83
128.59
128.47
128.42
123.76
123.71
116.22
115.92
114.86
77.16 CDCl3
51.39
24.20
24.18
f1 (ppm)

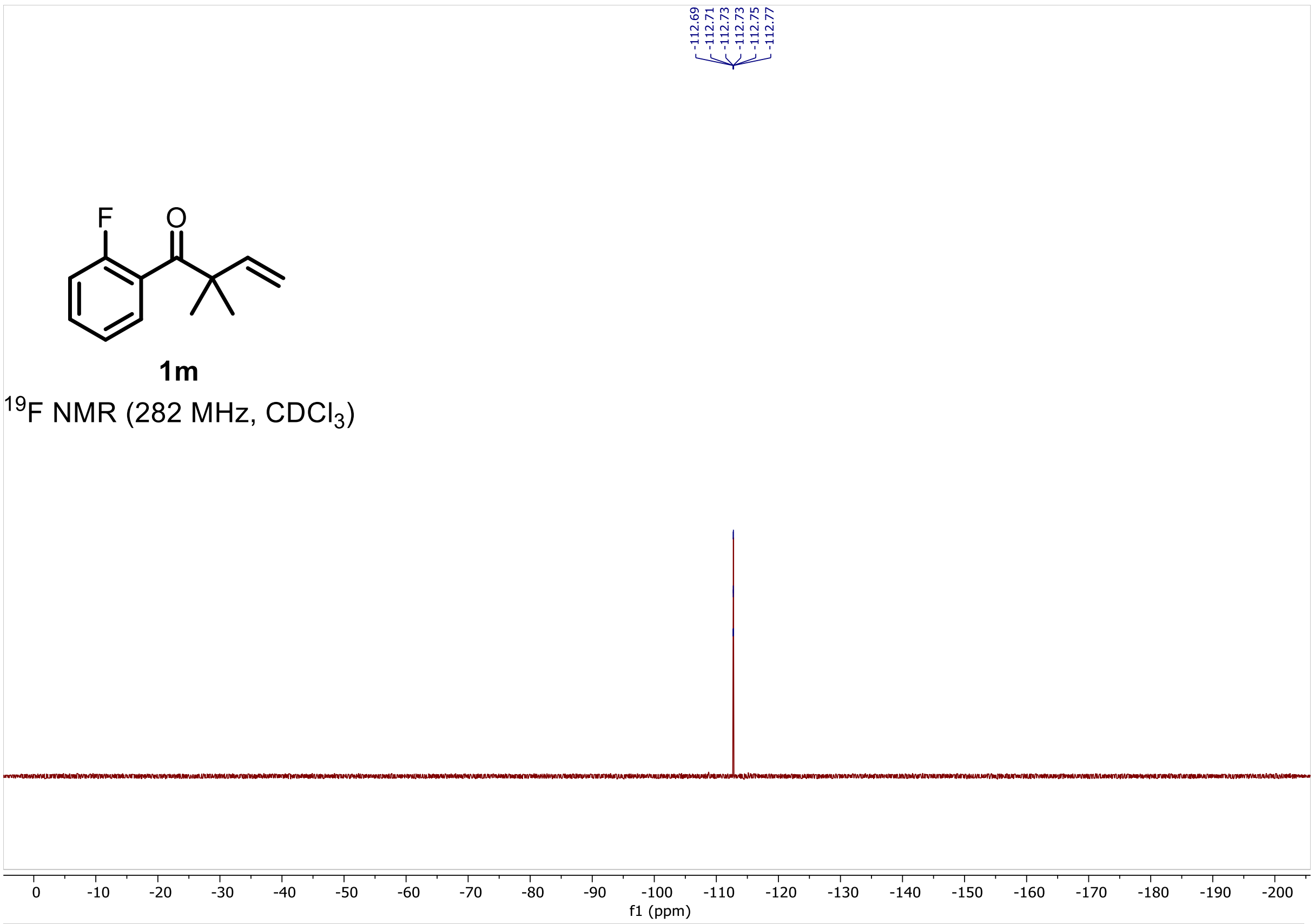

1m

^{19}F NMR (282 MHz, $CDCl_3$)

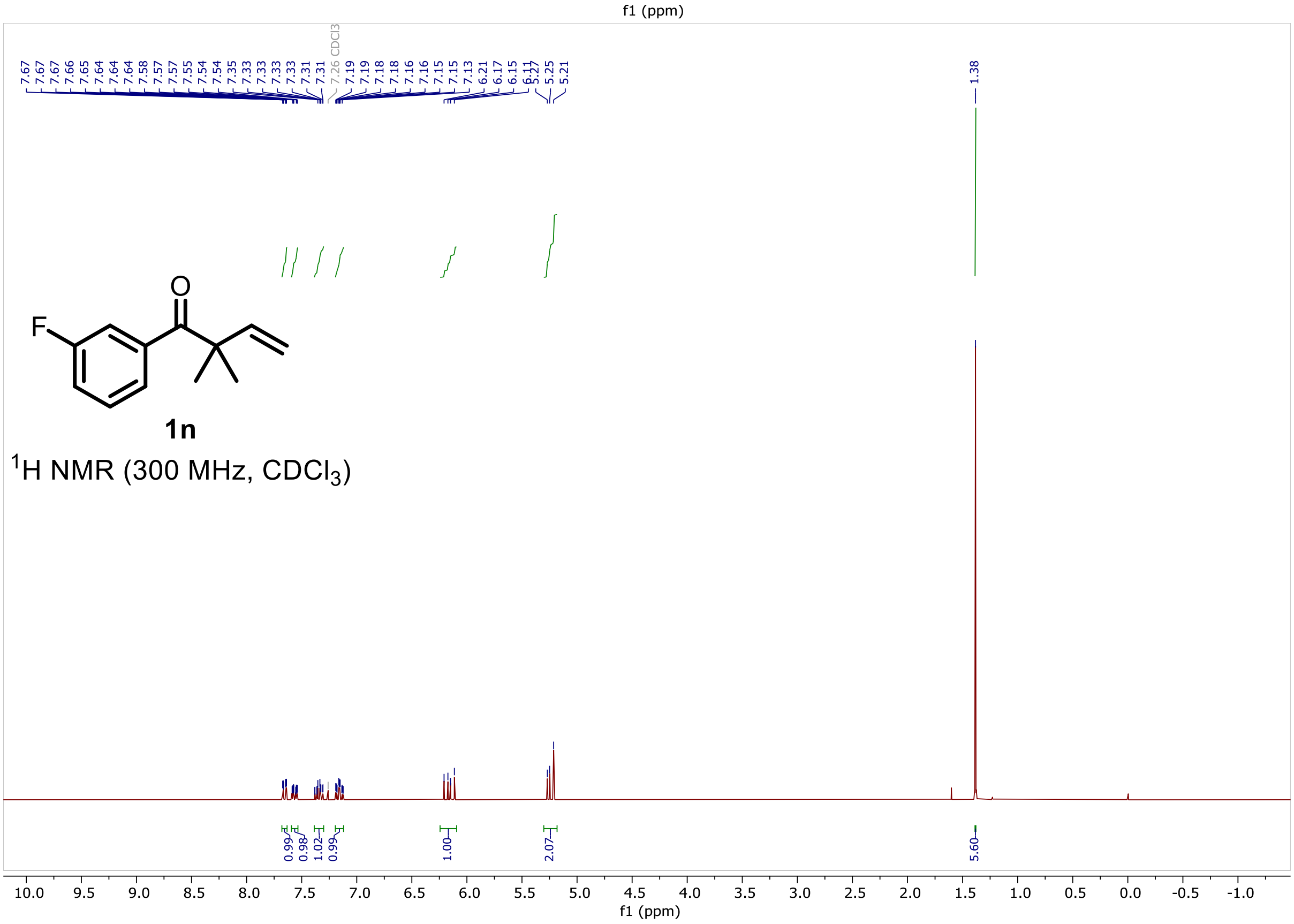

1n

^{1}H NMR (300 MHz, $CDCl_3$)

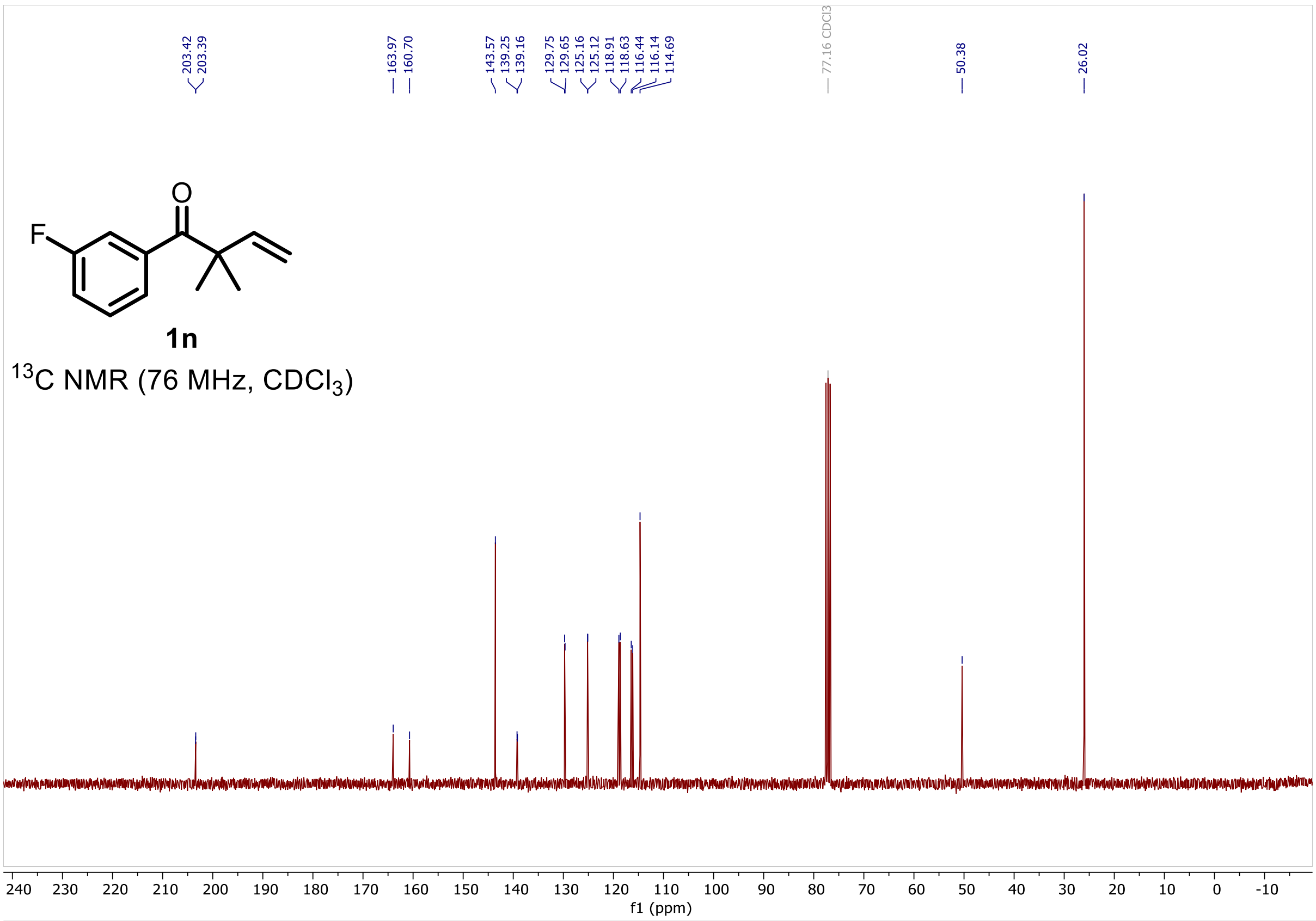
203.42
203.39
163.97
160.70
143.57
139.25
139.16
129.75
129.65
125.16
125.12
118.91
118.63
116.44
116.14
114.69
77.16 CDCl3
50.38
26.02
F
O
1n
^{13}C NMR (76 MHz, CDCl$_3$)
f1 (ppm)

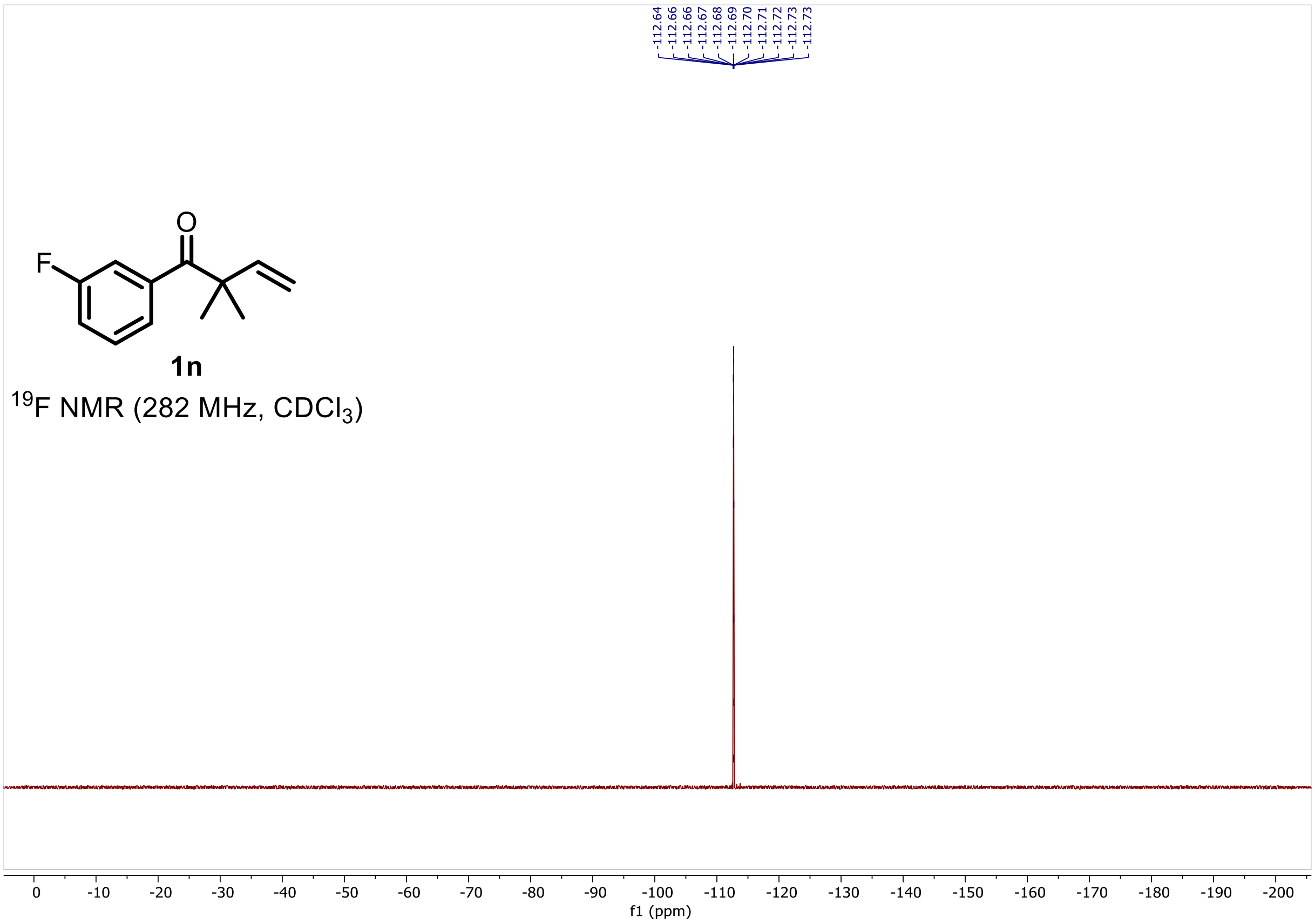
-112.64
-112.66
-112.66
-112.67
-112.68
-112.69
-112.70
-112.71
-112.72
-112.73
-112.73
F
O
1n
^{19}F NMR (282 MHz, CDCl$_3$)
f1 (ppm)

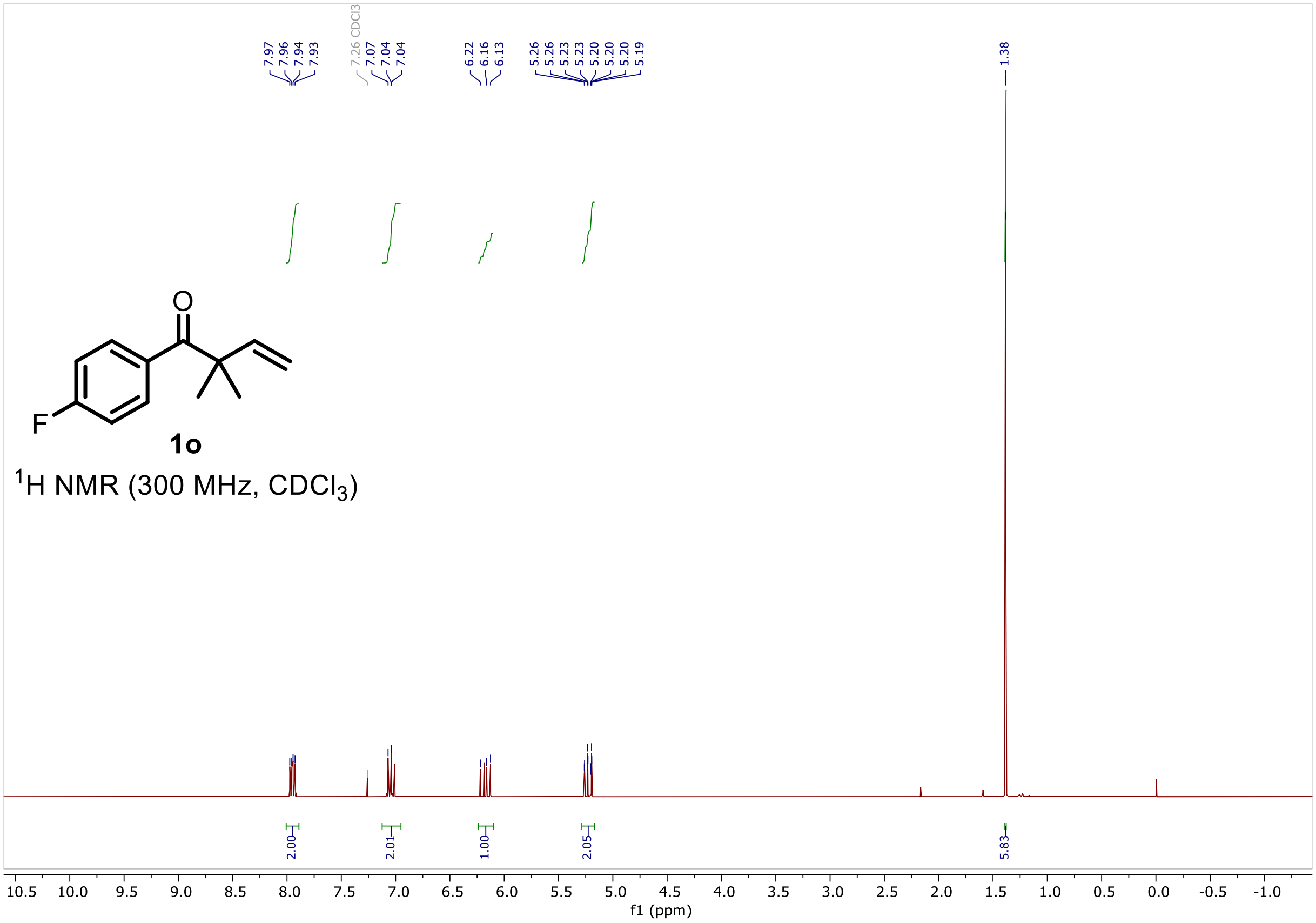
7.97
7.96
7.94
7.93
7.26 CDCl3
7.07
7.04
7.04
6.22
6.16
6.13
5.26
5.26
5.23
5.23
5.20
5.20
5.20
5.19
1.38
O
F
1o
^{1}H NMR (300 MHz, CDCl$_3$)
2.00
2.01
1.00
2.05
5.83
f1 (ppm)

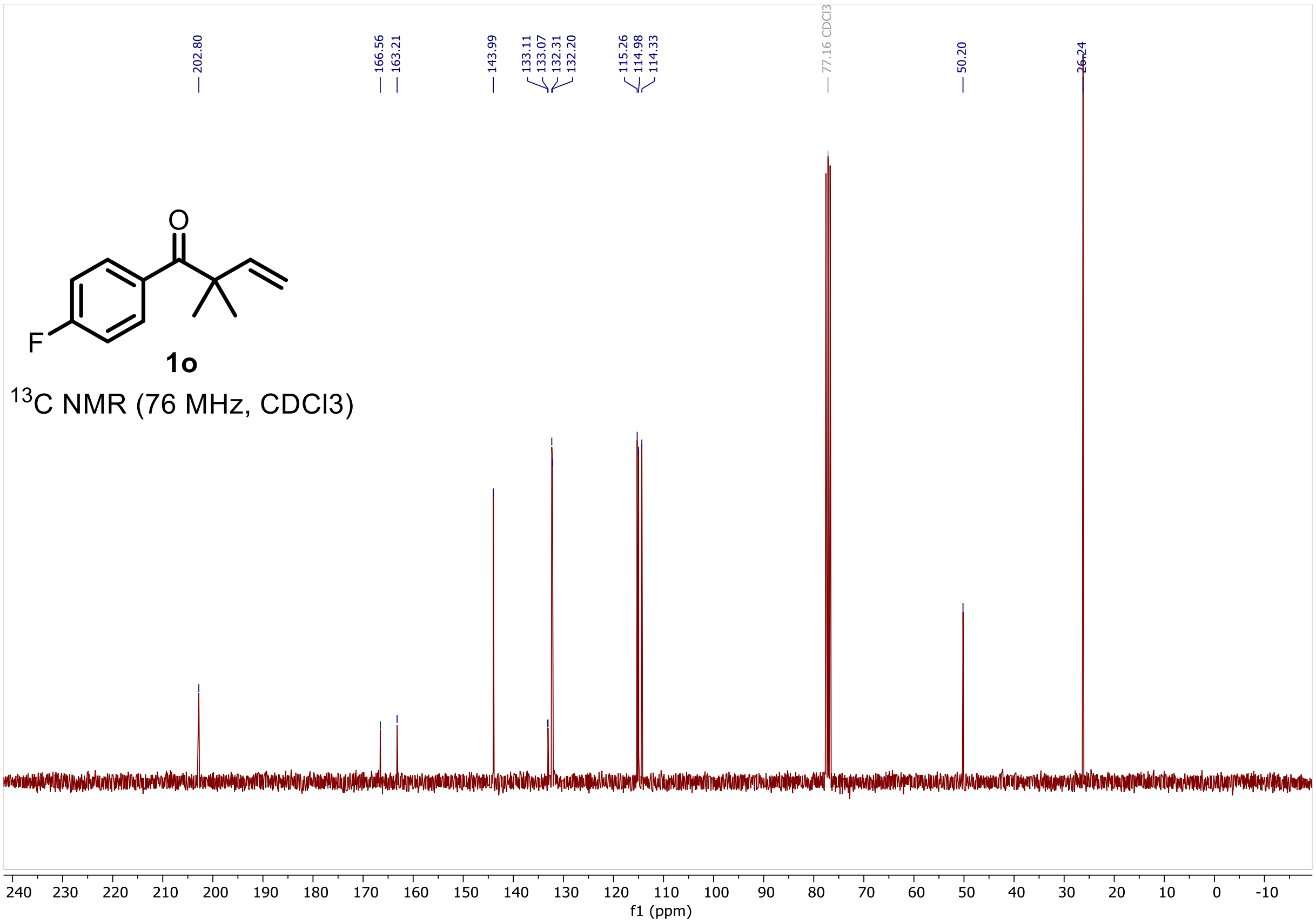
202.80
166.56
163.21
143.99
133.11
133.07
132.31
132.20
115.26
114.98
114.33
77.16 CDCl3
50.20
26.24
O
F
1o
^{13}C NMR (76 MHz, CDCl3)
f1 (ppm)

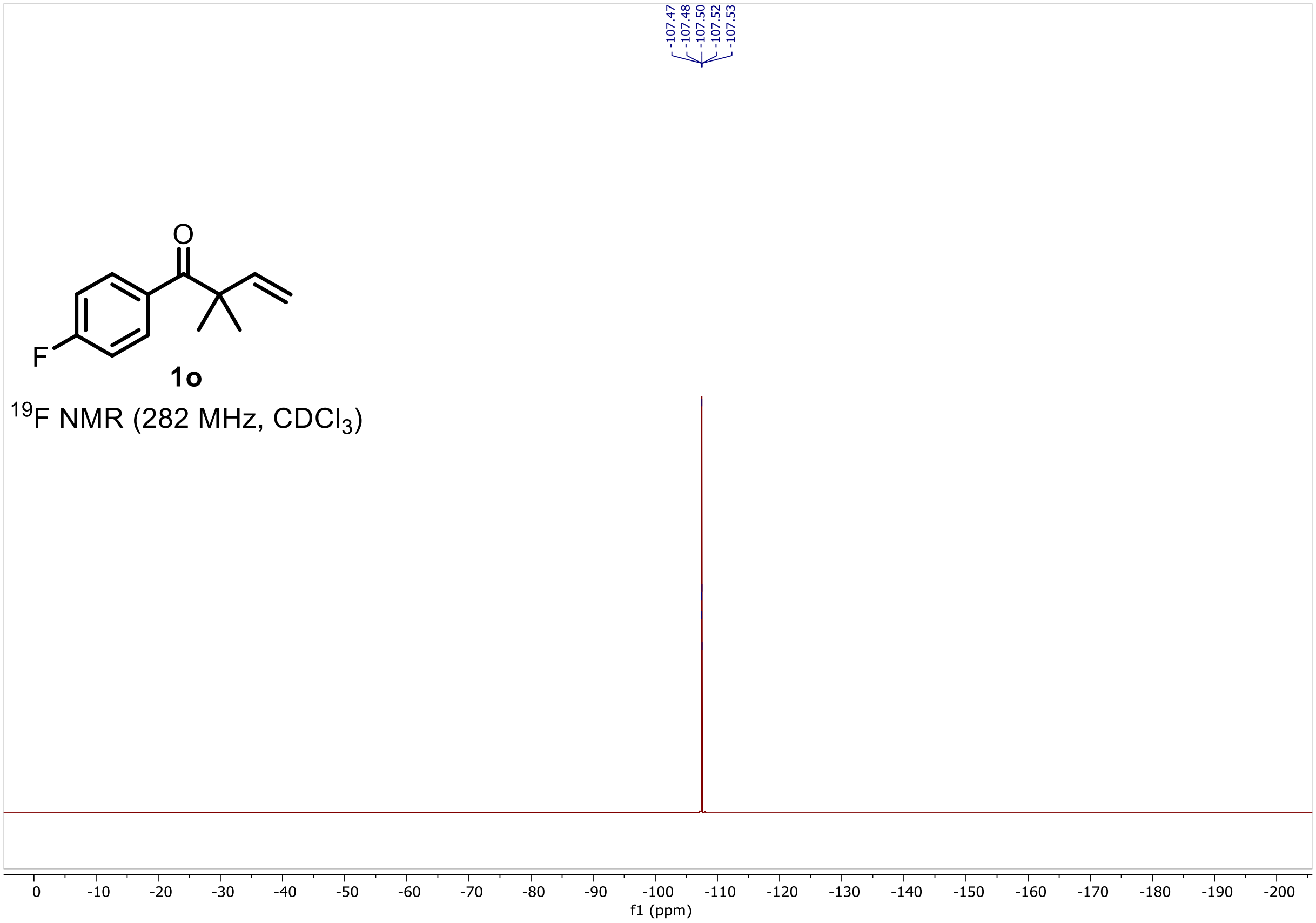

1o

^{19}F NMR (282 MHz, $CDCl_3$)

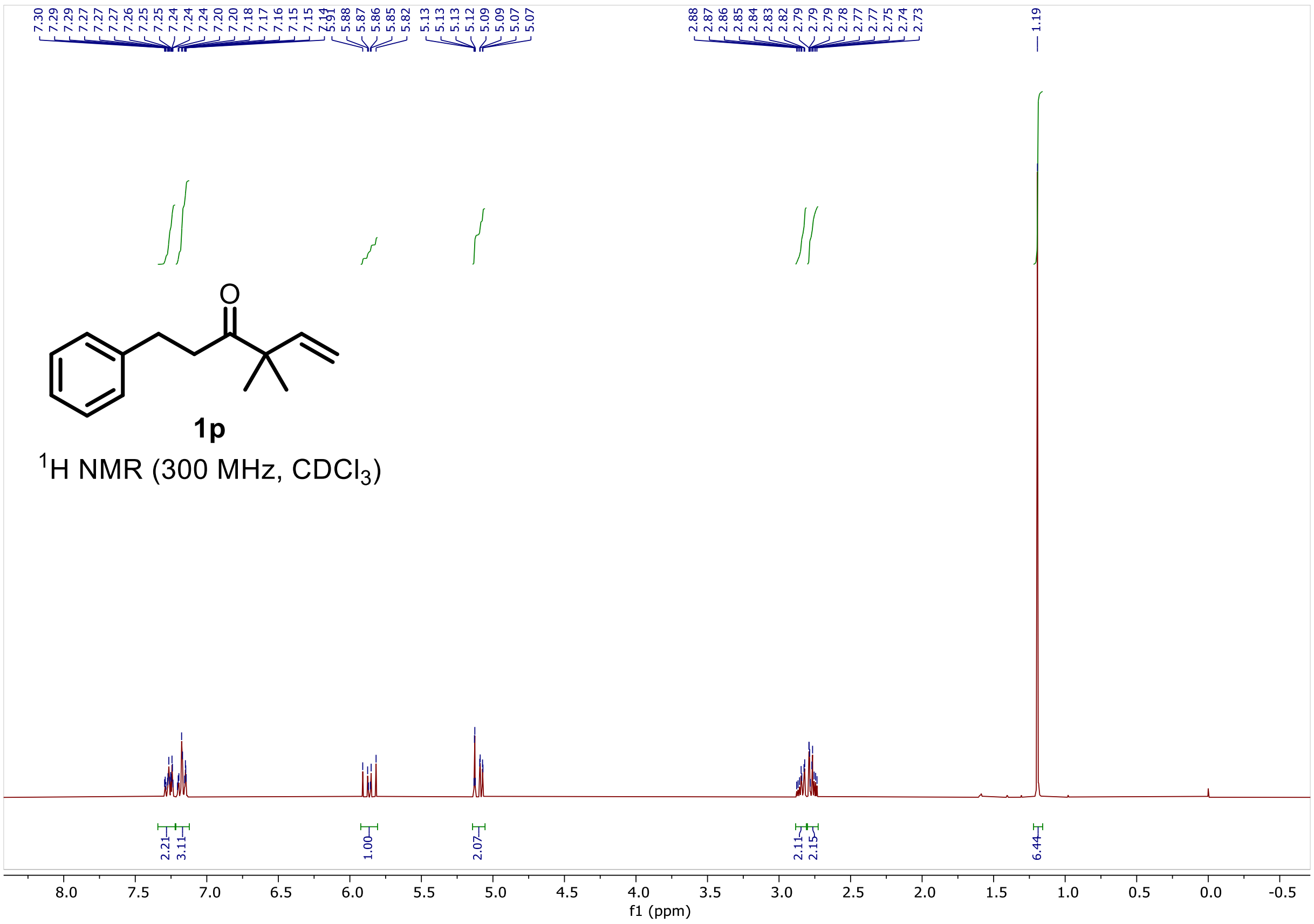

1p

^{1}H NMR (300 MHz, $CDCl_3$)

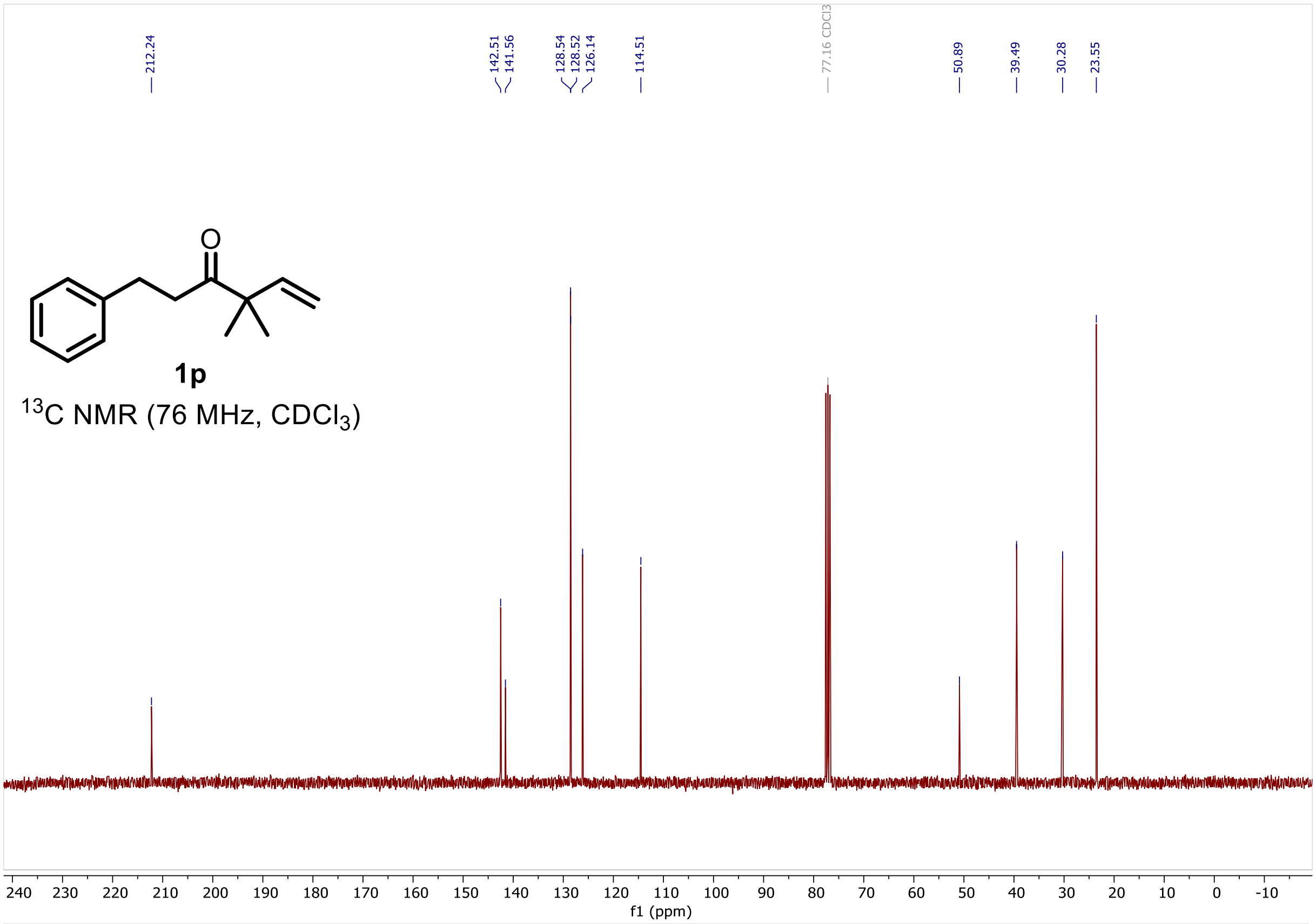

1p

^{13}C NMR (76 MHz, $CDCl_3$)

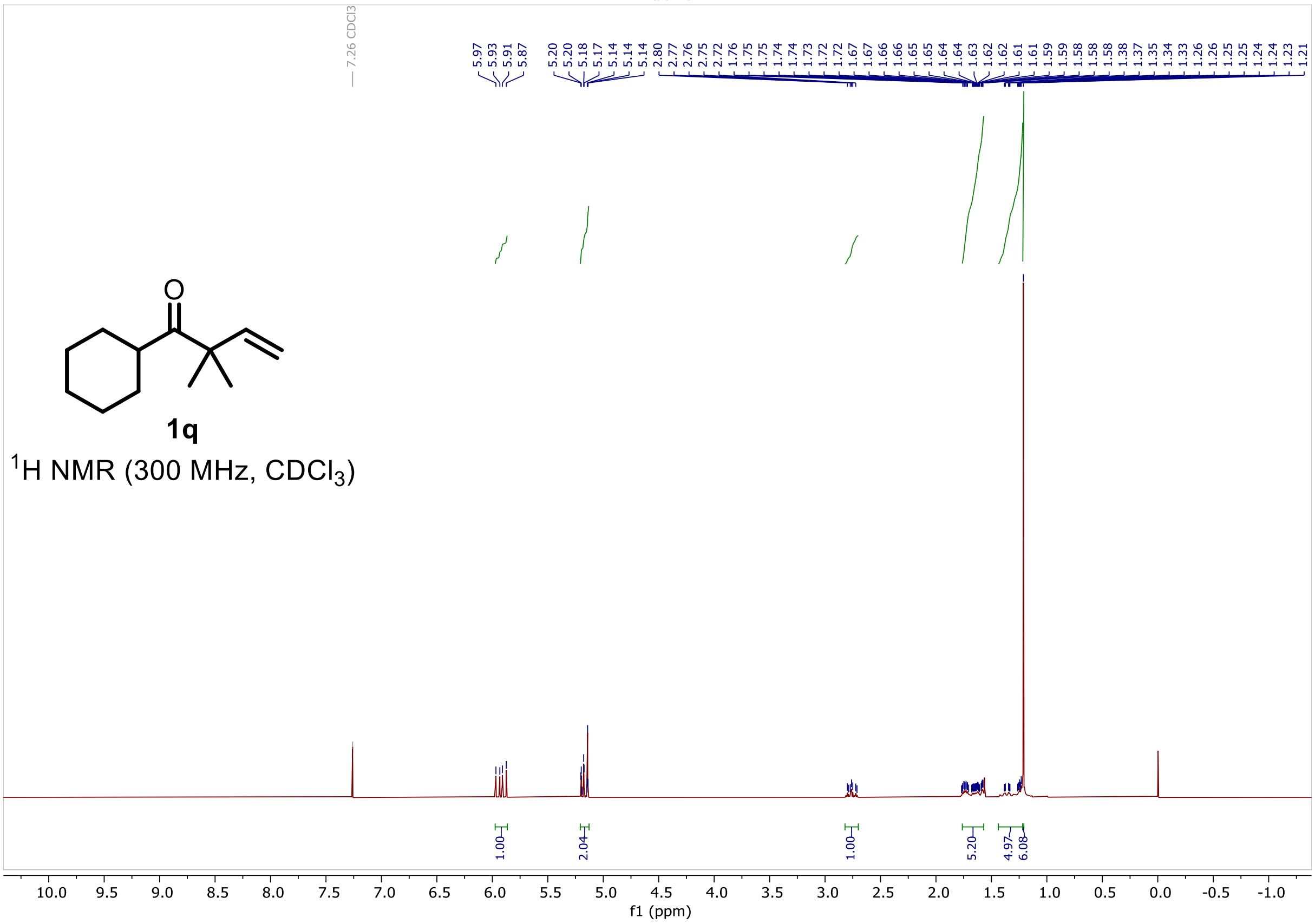

1q

^{1}H NMR (300 MHz, $CDCl_3$)

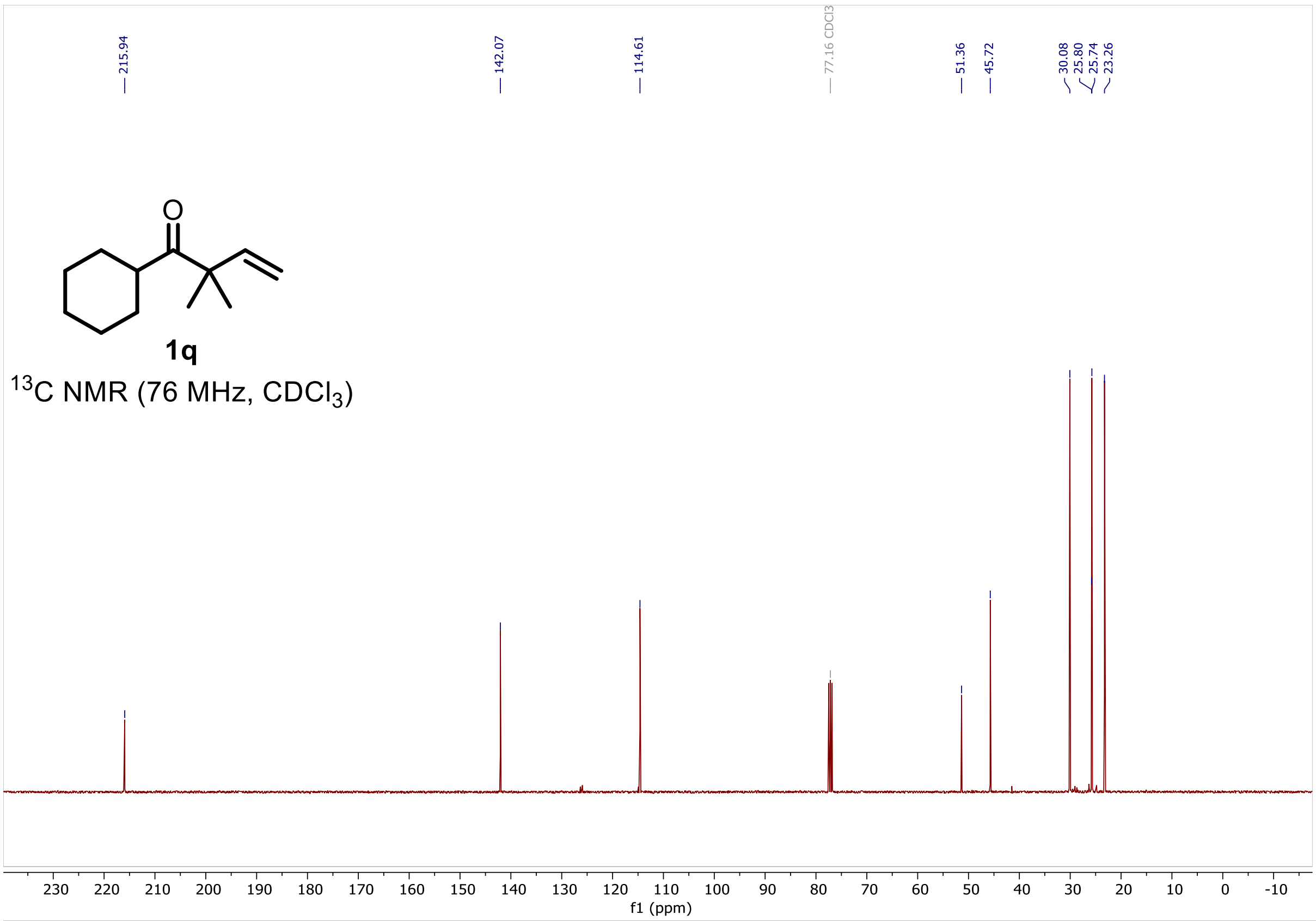
215.94
142.07
114.61
77.16 CDCl3
51.36
45.72
30.08
25.80
25.74
23.26
O
1q
^{13}C NMR (76 MHz, $CDCl_3$)
f1 (ppm)

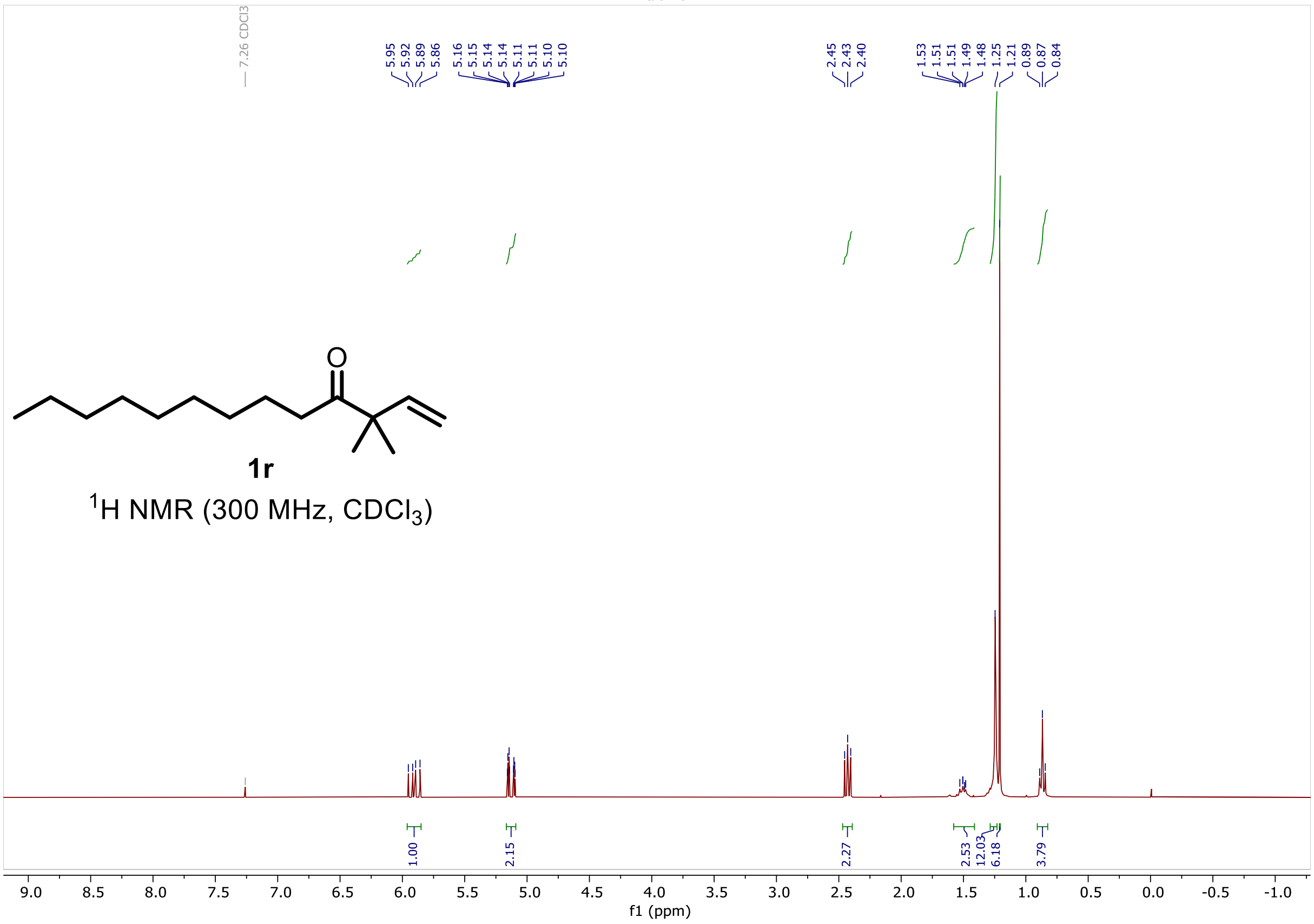
7.26 CDCl3
5.95
5.92
5.89
5.86
5.16
5.15
5.14
5.14
5.11
5.11
5.10
5.10
2.45
2.43
2.40
1.53
1.51
1.51
1.49
1.48
1.25
1.21
0.89
0.87
0.84
O
1r
^{1}H NMR (300 MHz, $CDCl_3$)
1.00
2.15
2.27
2.53
12.03
6.18
3.79
f1 (ppm)

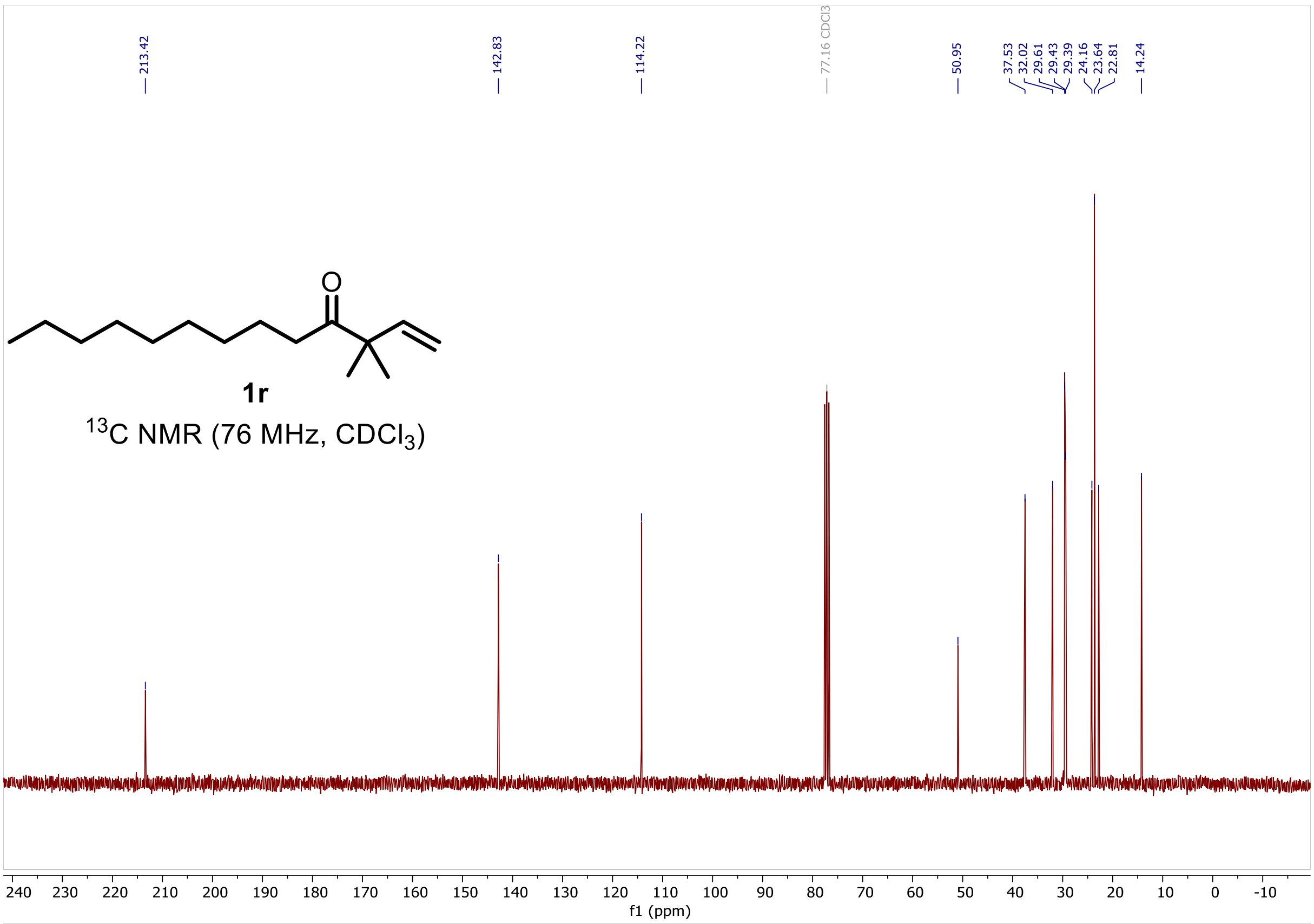

1r

^{13}C NMR (76 MHz, $CDCl_3$)

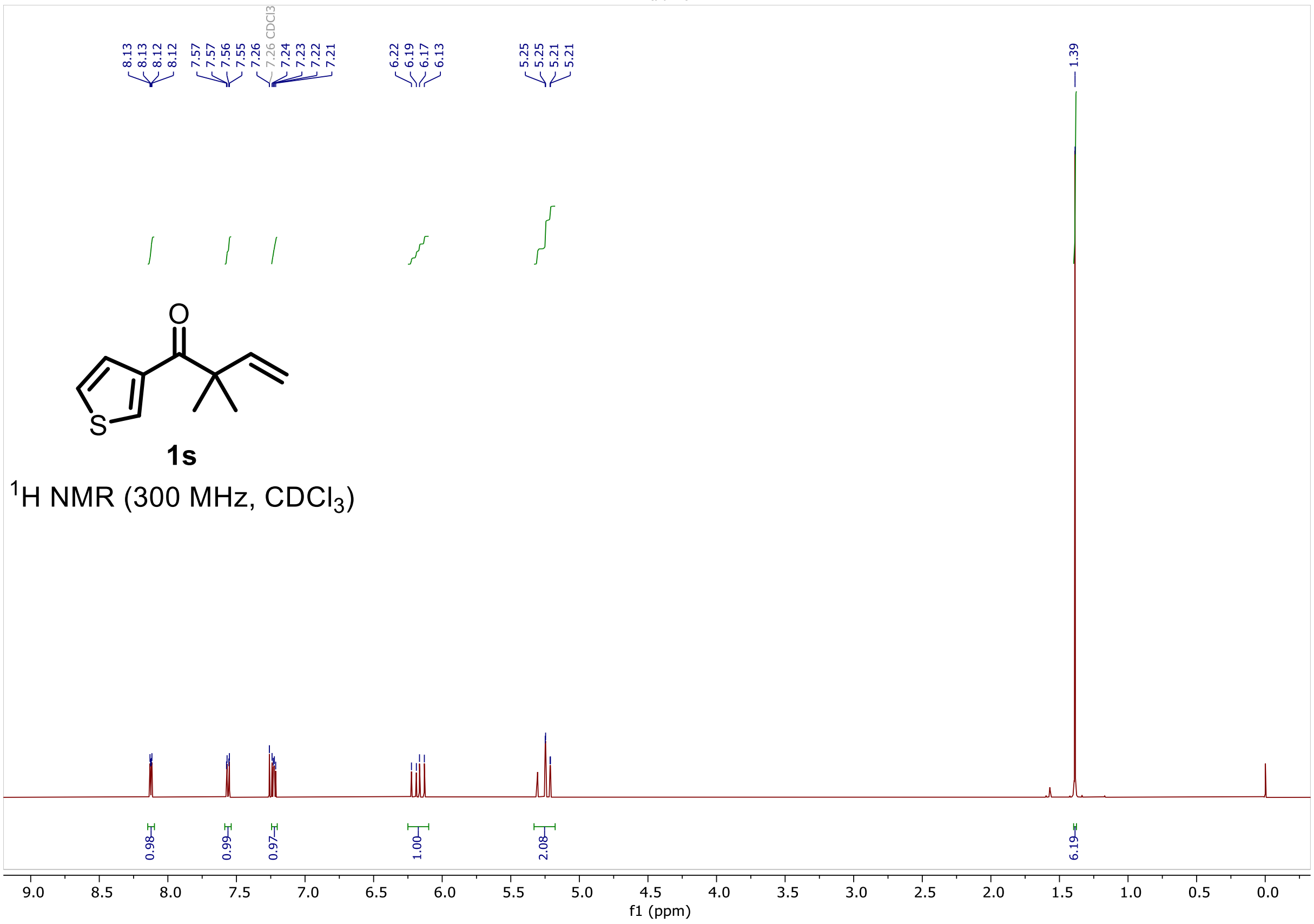

1s

^{1}H NMR (300 MHz, $CDCl_3$)

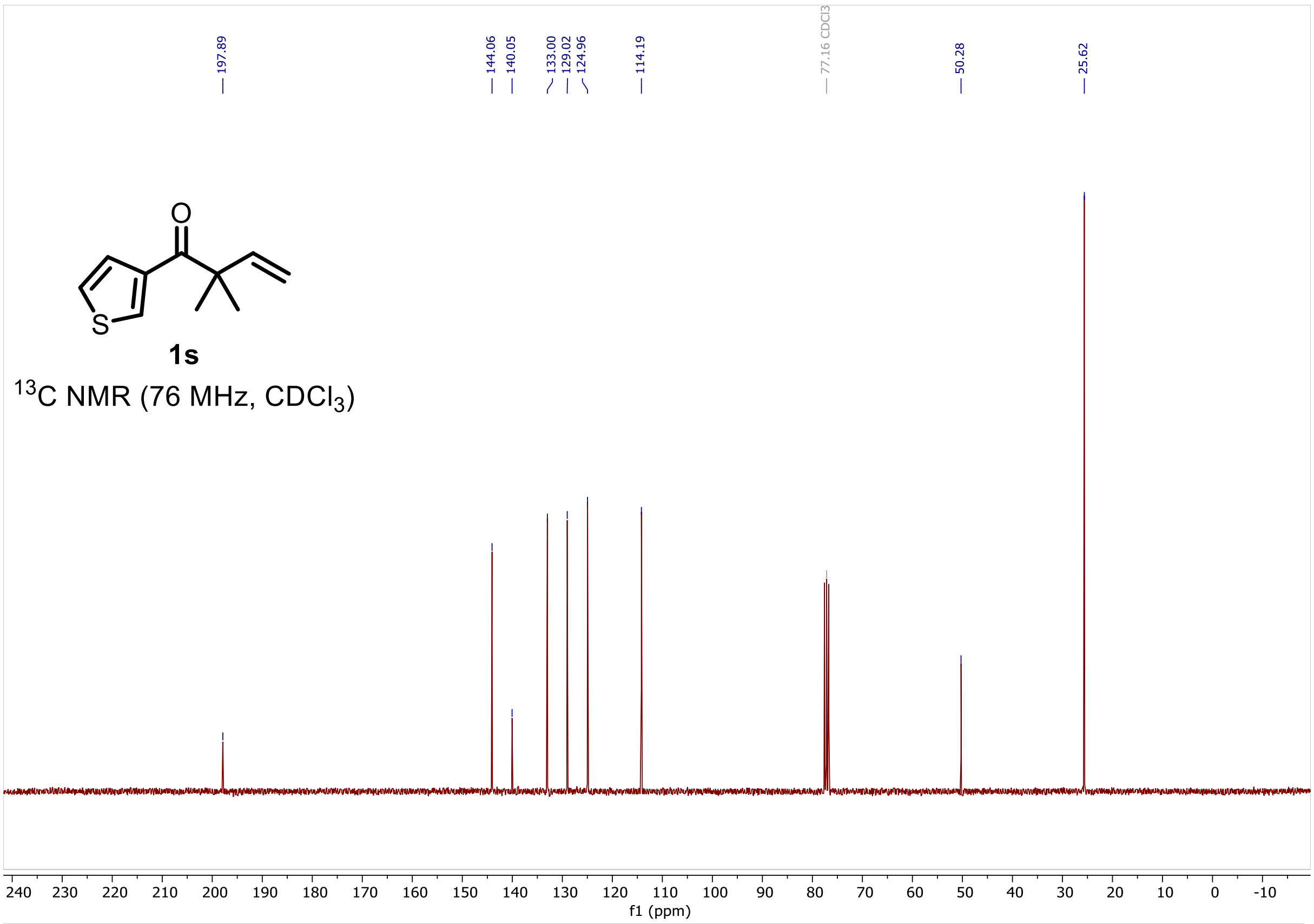
197.89
144.06
140.05
133.00
129.02
124.96
114.19
77.16 CDCl3
50.28
25.62
O
S
1s
^{13}C NMR (76 MHz, CDCl$_3$)
f1 (ppm)

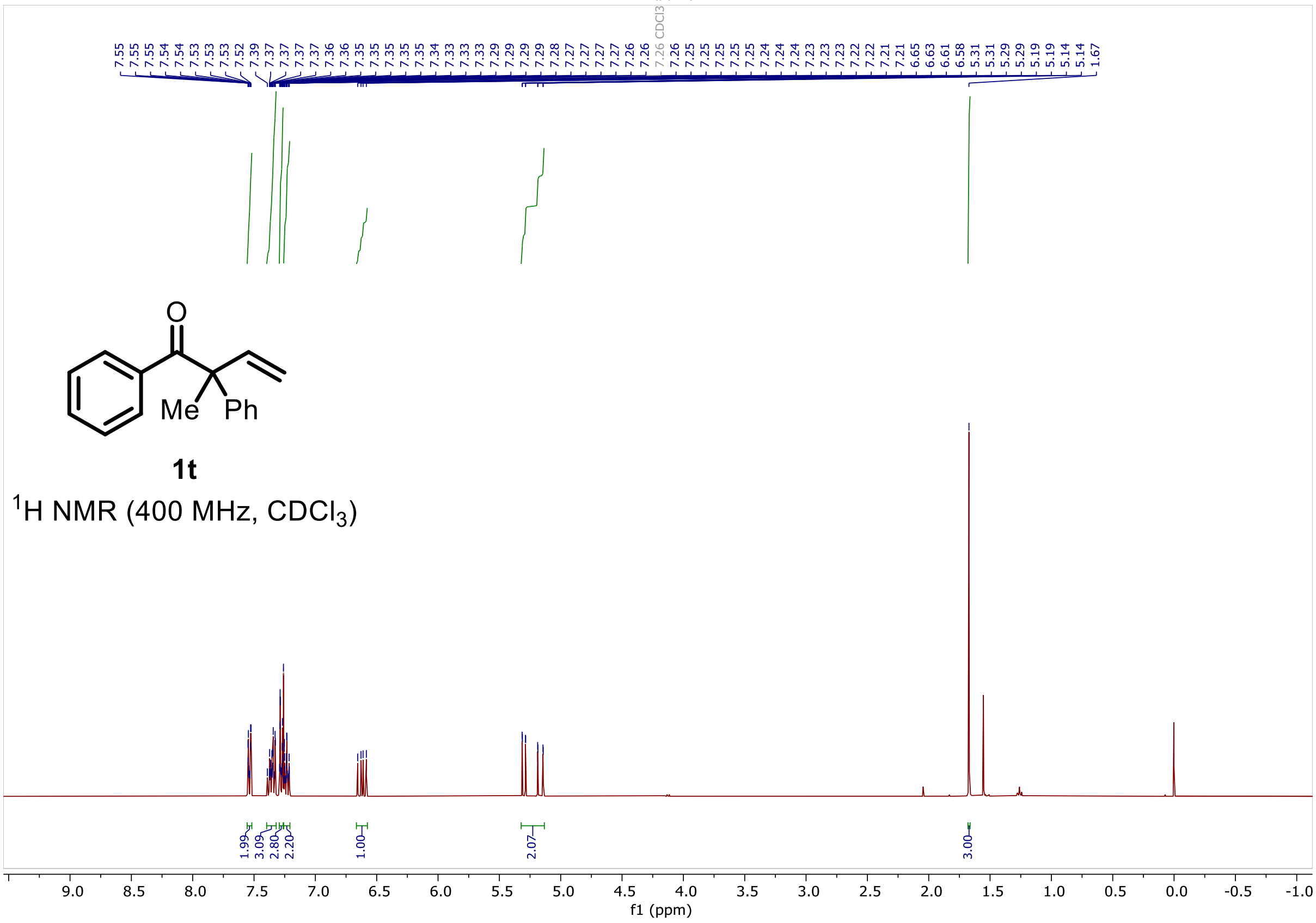
O
Me
Ph
1t
^{1}H NMR (400 MHz, CDCl$_3$)
1.99
3.09
2.80
2.20
1.00
2.07
3.00
f1 (ppm)

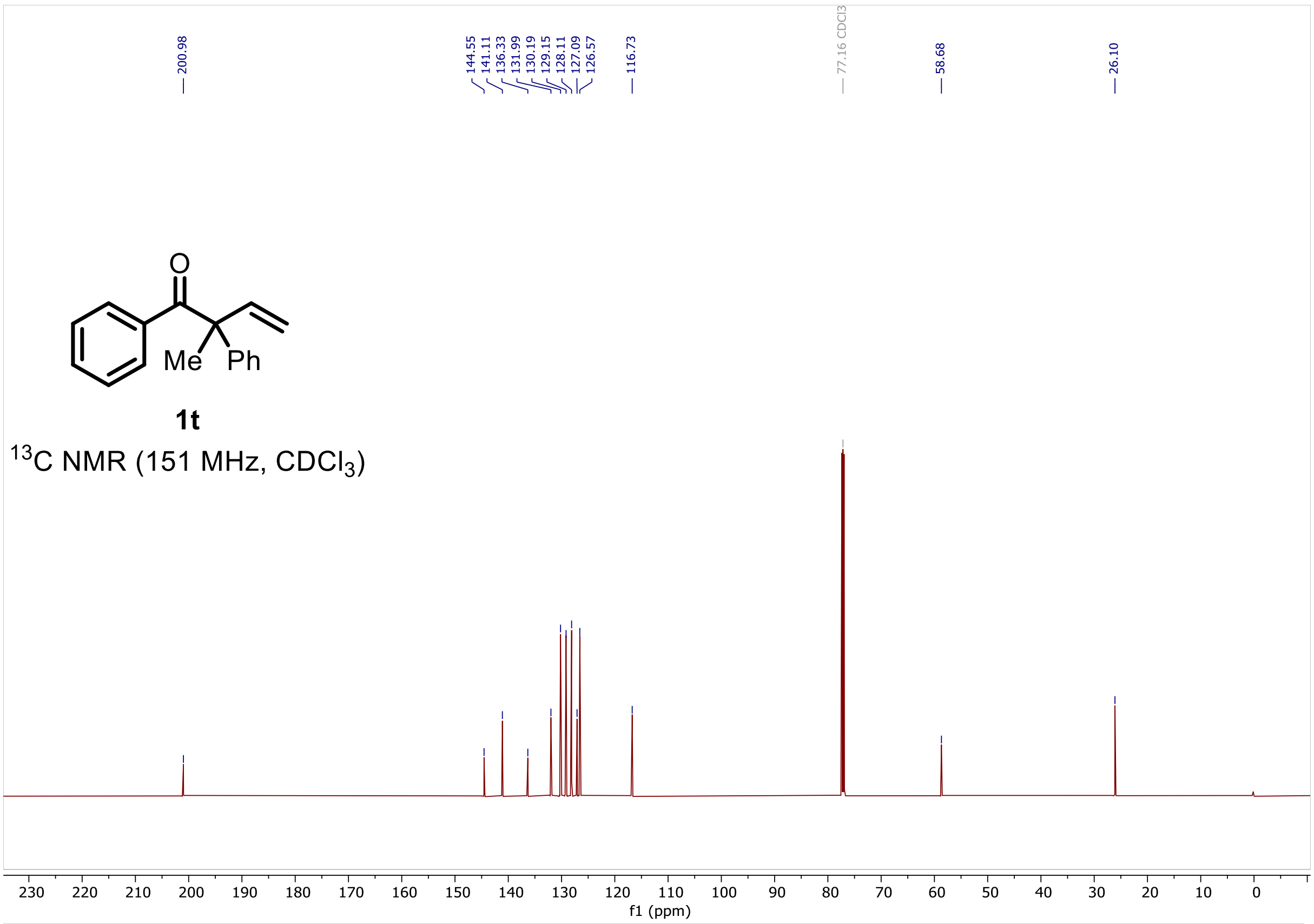
200.98
144.55
141.11
136.33
131.99
130.19
129.15
128.11
127.09
126.57
116.73
77.16 CDCl3
58.68
26.10
1t
13C NMR (151 MHz, CDCl3)
f1 (ppm)

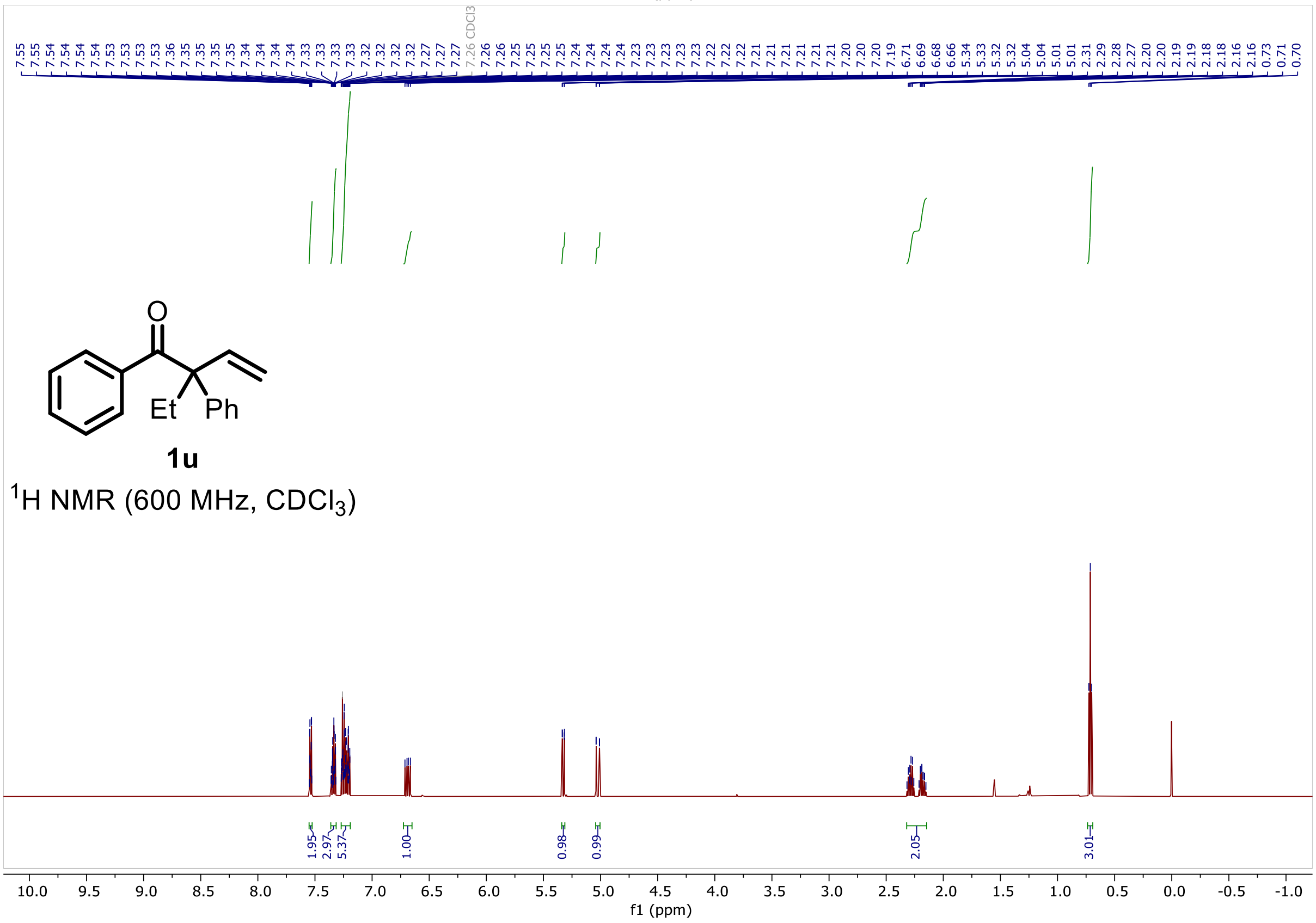
1u
1H NMR (600 MHz, CDCl3)
1.95
2.97
5.37
1.00
0.98
0.99
2.05
3.01
f1 (ppm)

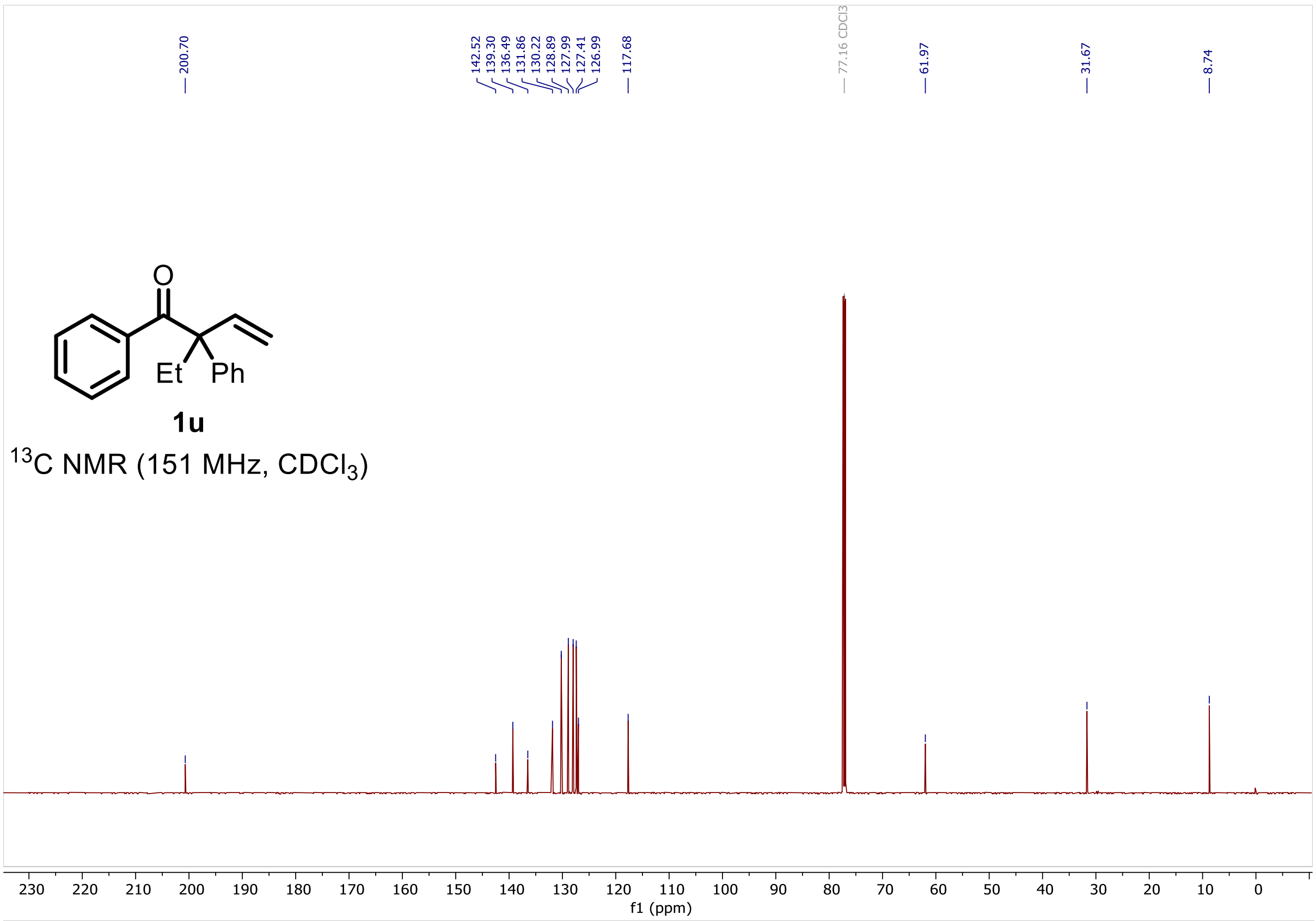
200.70
142.52
139.30
136.49
131.86
130.22
128.89
127.99
127.41
126.99
117.68
77.16 CDCl3
61.97
31.67
8.74
O
Et
Ph
1u
^{13}C NMR (151 MHz, $CDCl_3$)
230 220 210 200 190 180 170 160 150 140 130 120 110 100 90 80 70 60 50 40 30 20 10 0
f1 (ppm)

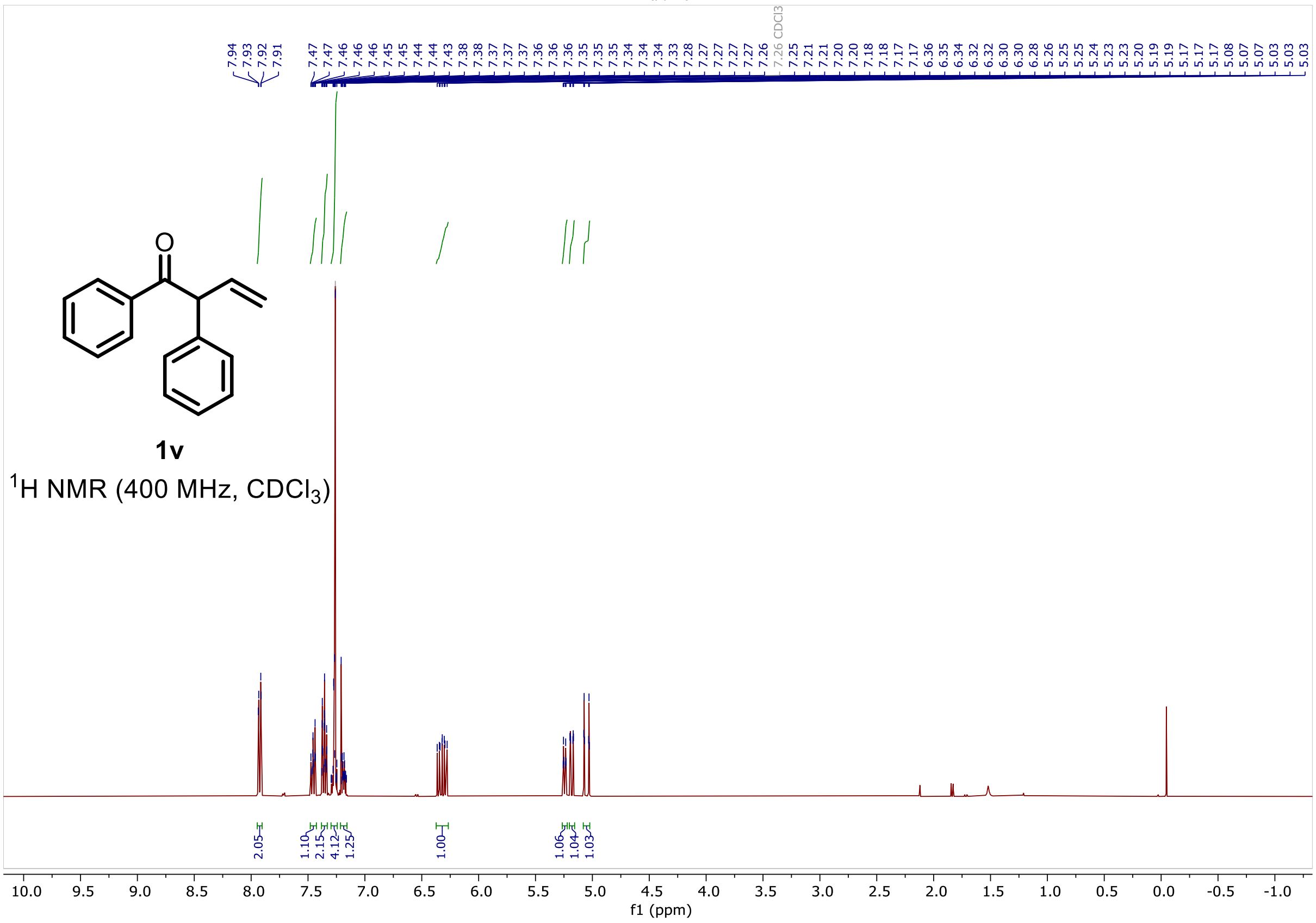
7.94
7.93
7.92
7.91
7.47
7.47
7.46
7.46
7.45
7.45
7.44
7.44
7.43
7.38
7.38
7.37
7.37
7.37
7.36
7.36
7.35
7.35
7.35
7.34
7.34
7.33
7.28
7.27
7.27
7.27
7.27
7.26
7.26 CDCl3
7.25
7.21
7.21
7.20
7.20
7.18
7.17
7.17
6.36
6.35
6.34
6.32
6.32
6.30
6.30
6.28
5.26
5.25
5.25
5.24
5.23
5.23
5.20
5.19
5.19
5.17
5.17
5.17
5.08
5.07
5.07
5.03
5.03
5.03
O
1v
^{1}H NMR (400 MHz, $CDCl_3$)
2.05
1.10
2.15
4.12
1.25
1.00
1.06
1.04
1.03
10.0 9.5 9.0 8.5 8.0 7.5 7.0 6.5 6.0 5.5 5.0 4.5 4.0 3.5 3.0 2.5 2.0 1.5 1.0 0.5 0.0 -0.5 -1.0
f1 (ppm)

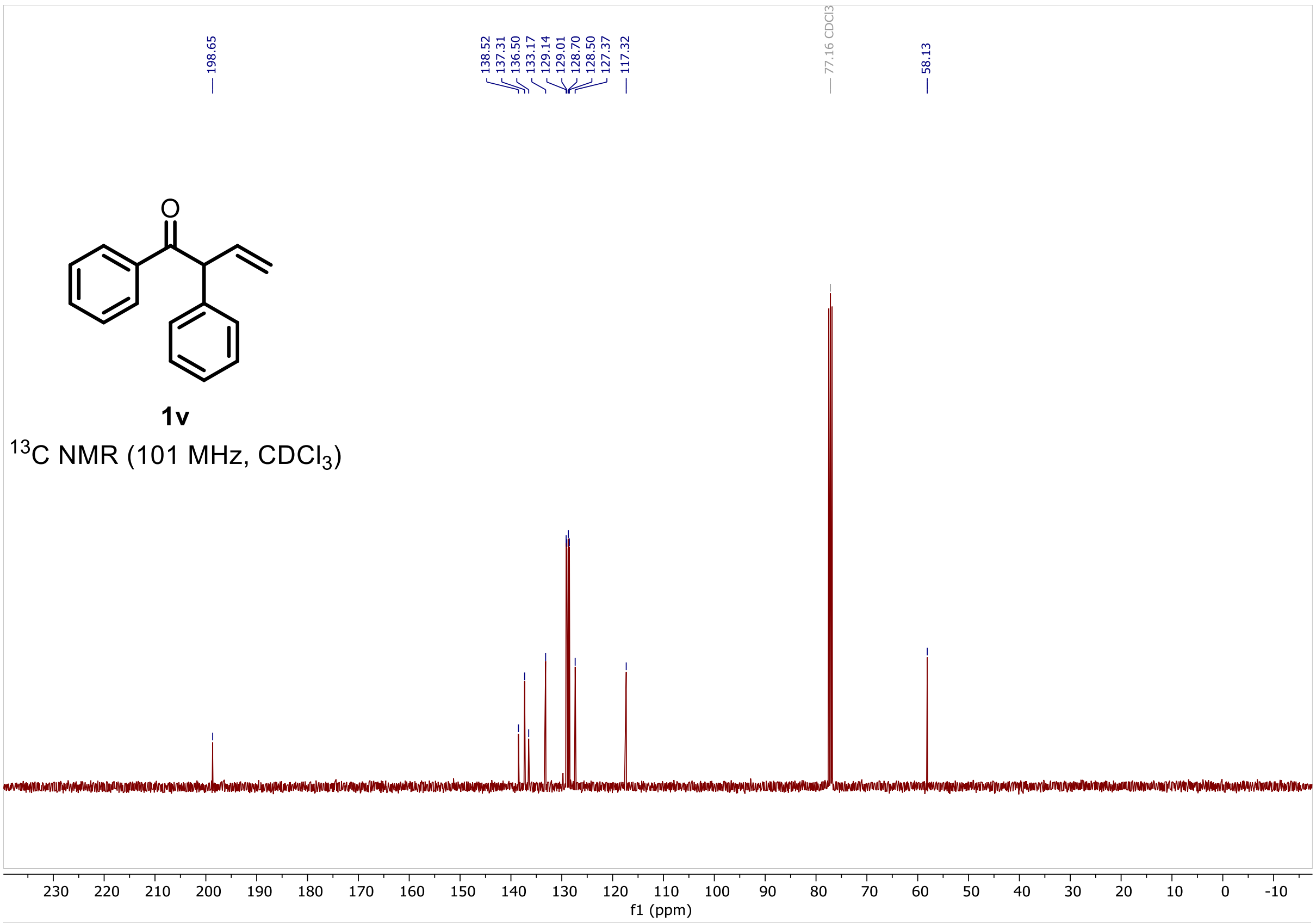
198.65
138.52
137.31
136.50
133.17
129.14
129.01
128.70
128.50
127.37
117.32
77.16 CDCl3
58.13
O
1v
13C NMR (101 MHz, CDCl3)
f1 (ppm)

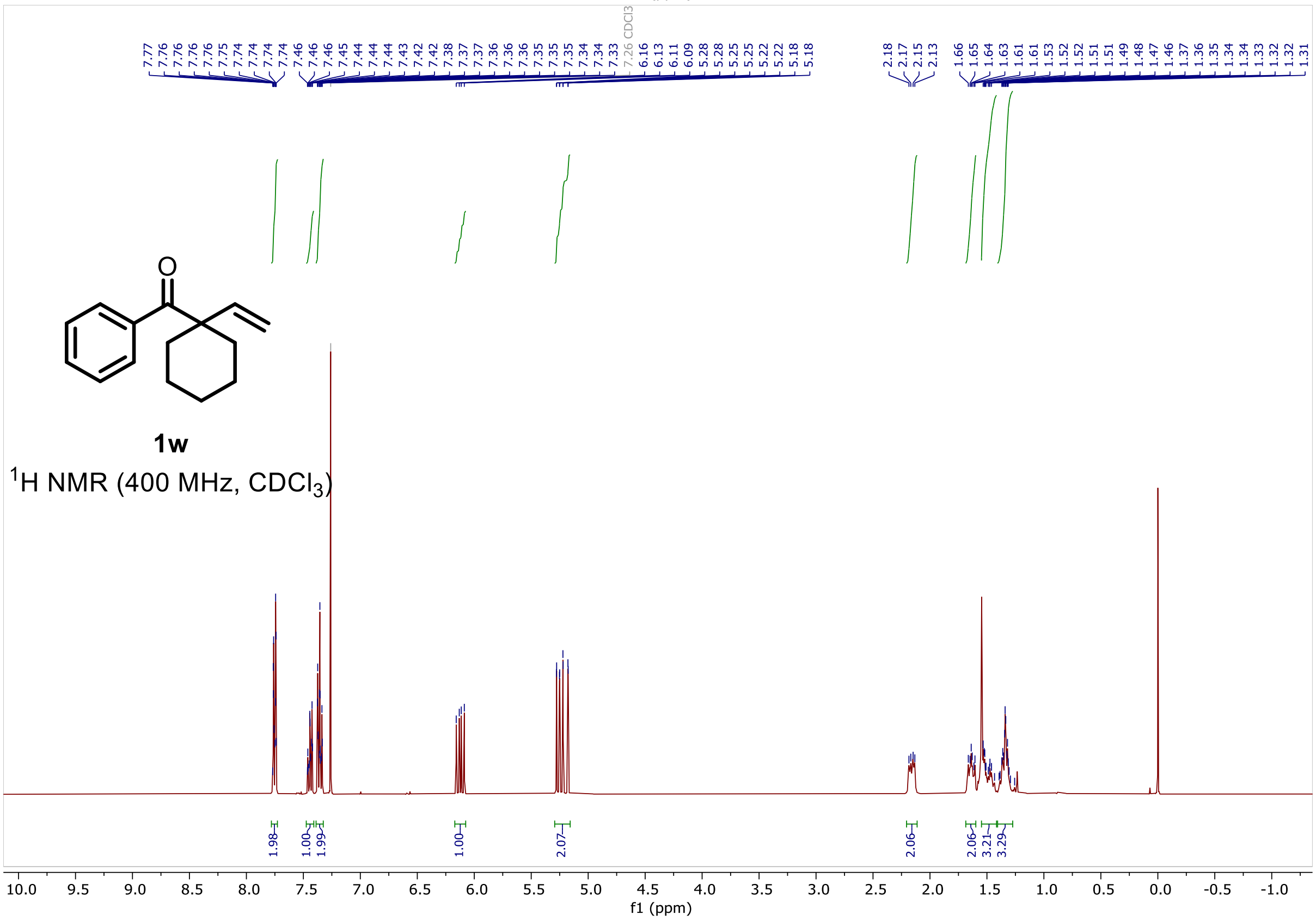
O
1w
1H NMR (400 MHz, CDCl3)
7.26 CDCl3
1.98
1.00
1.99
1.00
2.07
2.06
2.06
3.21
3.29
f1 (ppm)

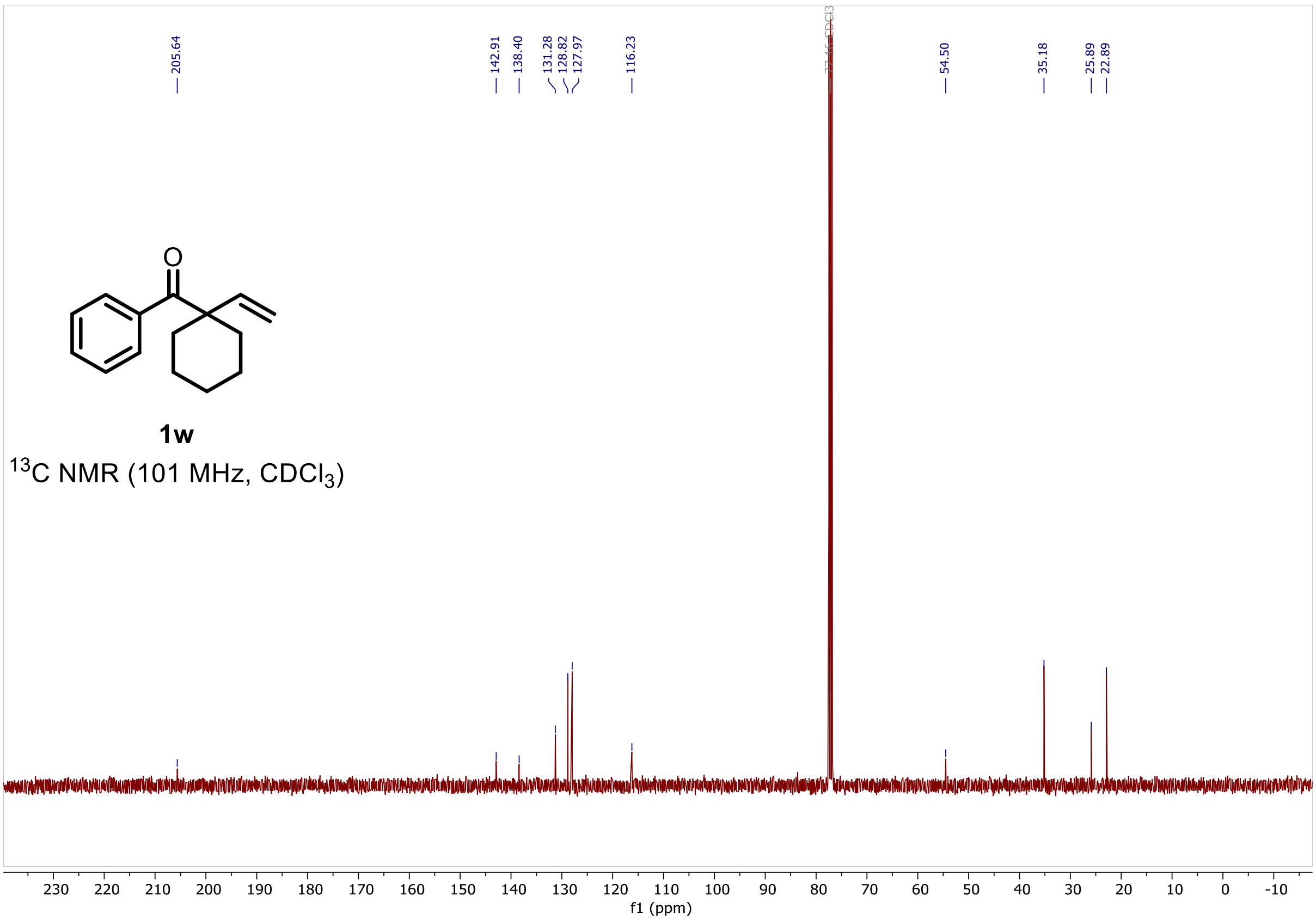

^{13}C NMR (101 MHz, $CDCl_3$)

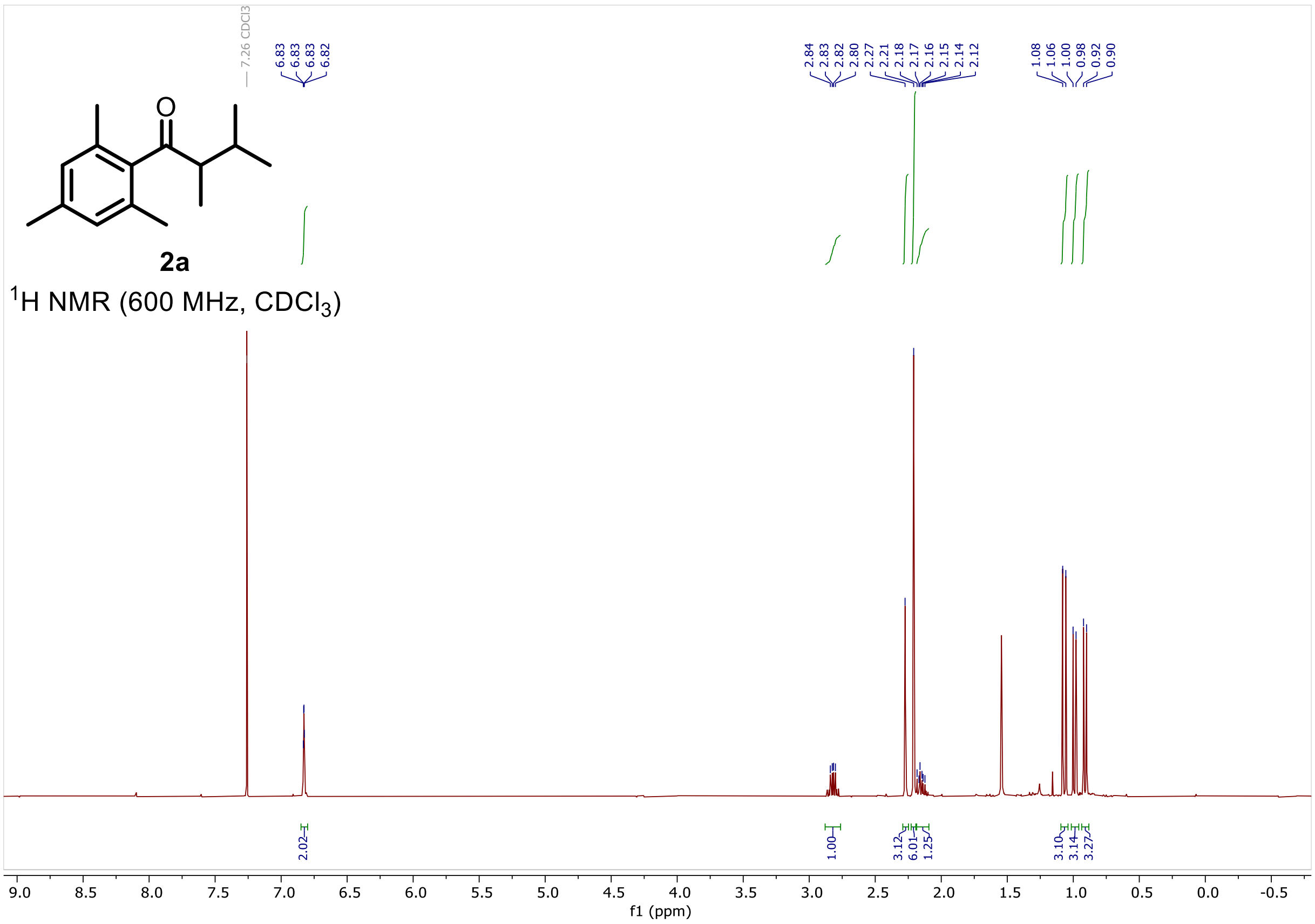

^{1}H NMR (600 MHz, $CDCl_3$)

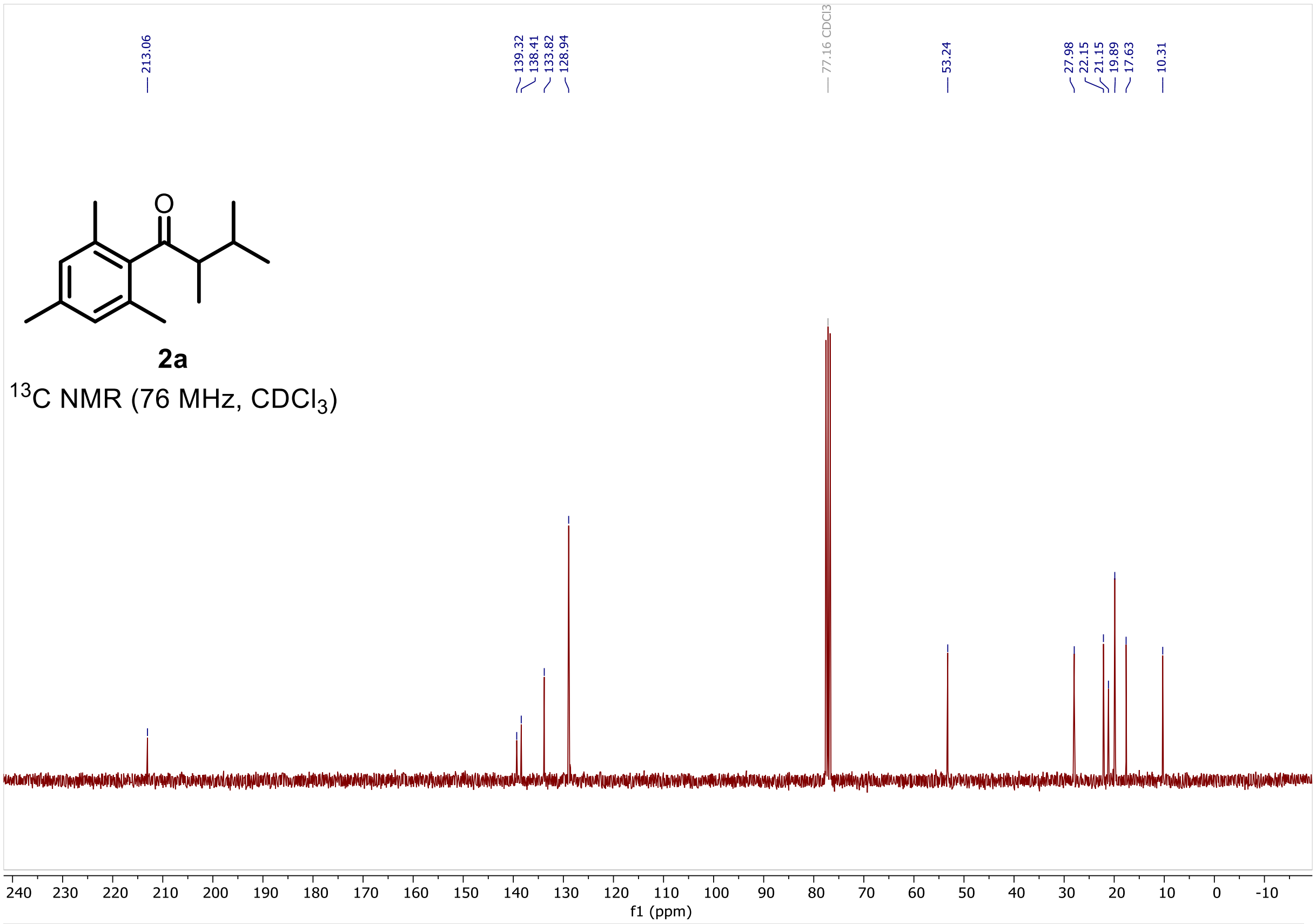

2a

^{13}C NMR (76 MHz, $CDCl_3$)

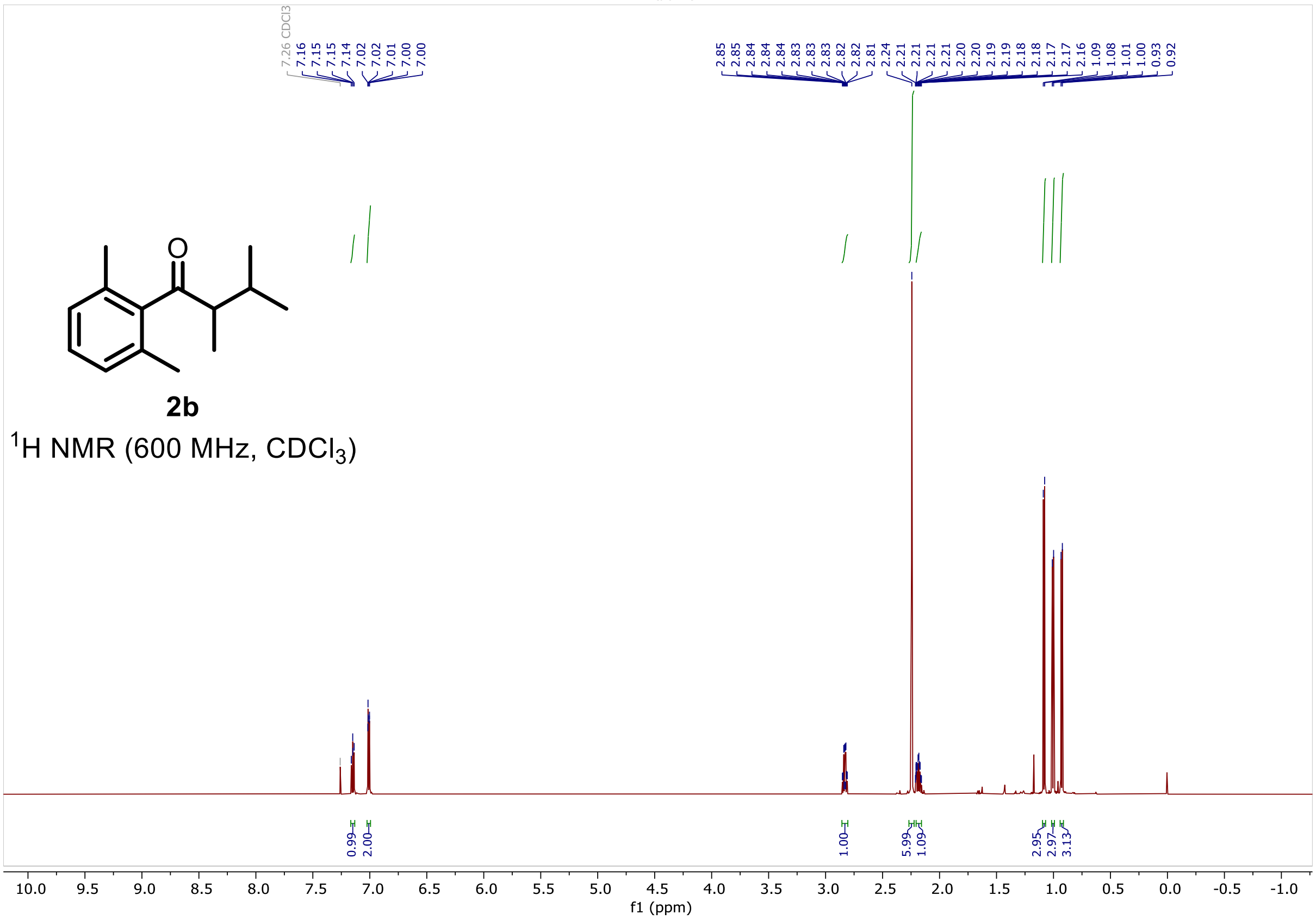

2b

^{1}H NMR (600 MHz, $CDCl_3$)

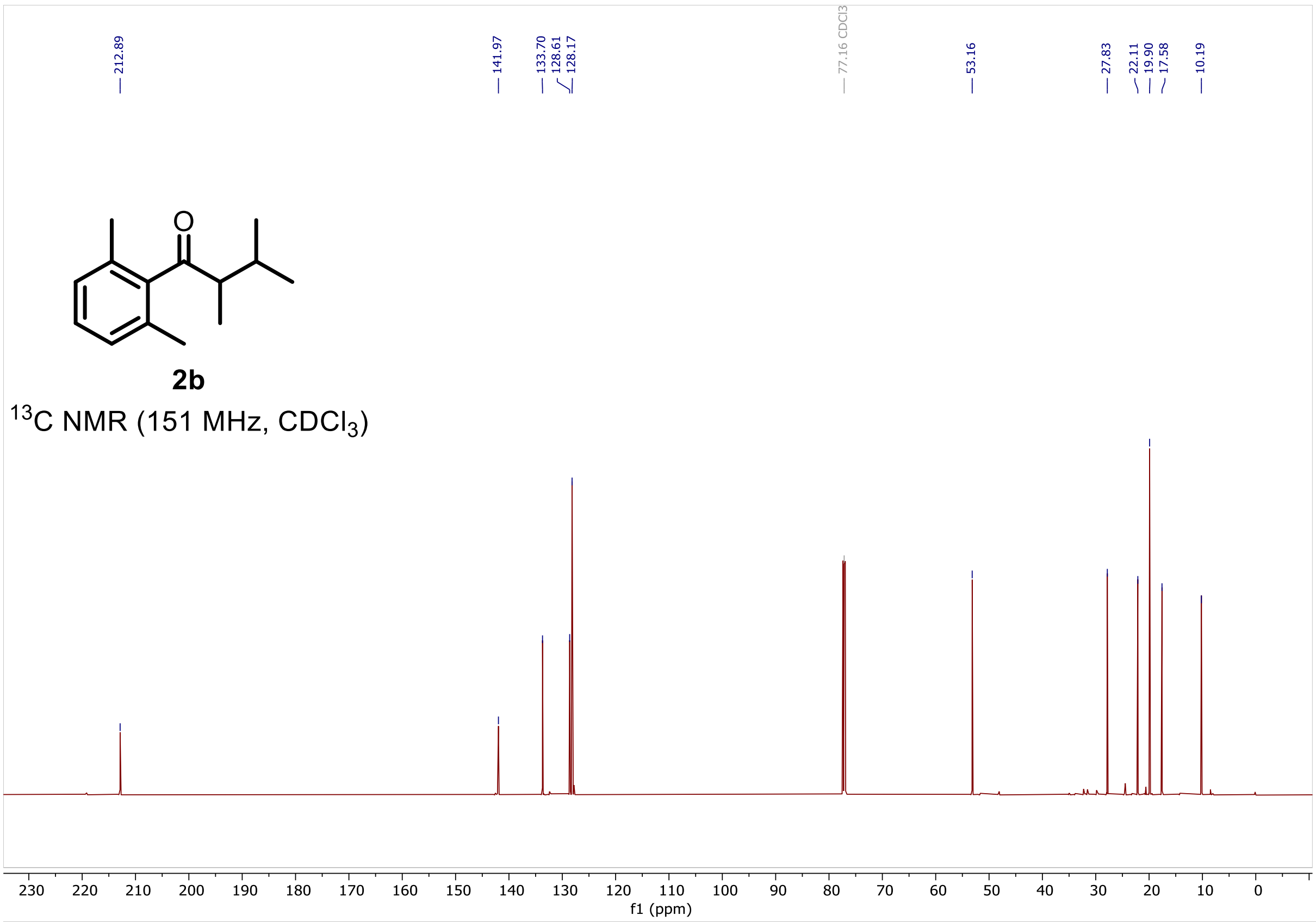
212.89
141.97
133.70
128.61
128.17
77.16 CDCl3
53.16
27.83
22.11
19.90
17.58
10.19
O
2b
13C NMR (151 MHz, CDCl3)
230 220 210 200 190 180 170 160 150 140 130 120 110 100 90 80 70 60 50 40 30 20 10 0
f1 (ppm)

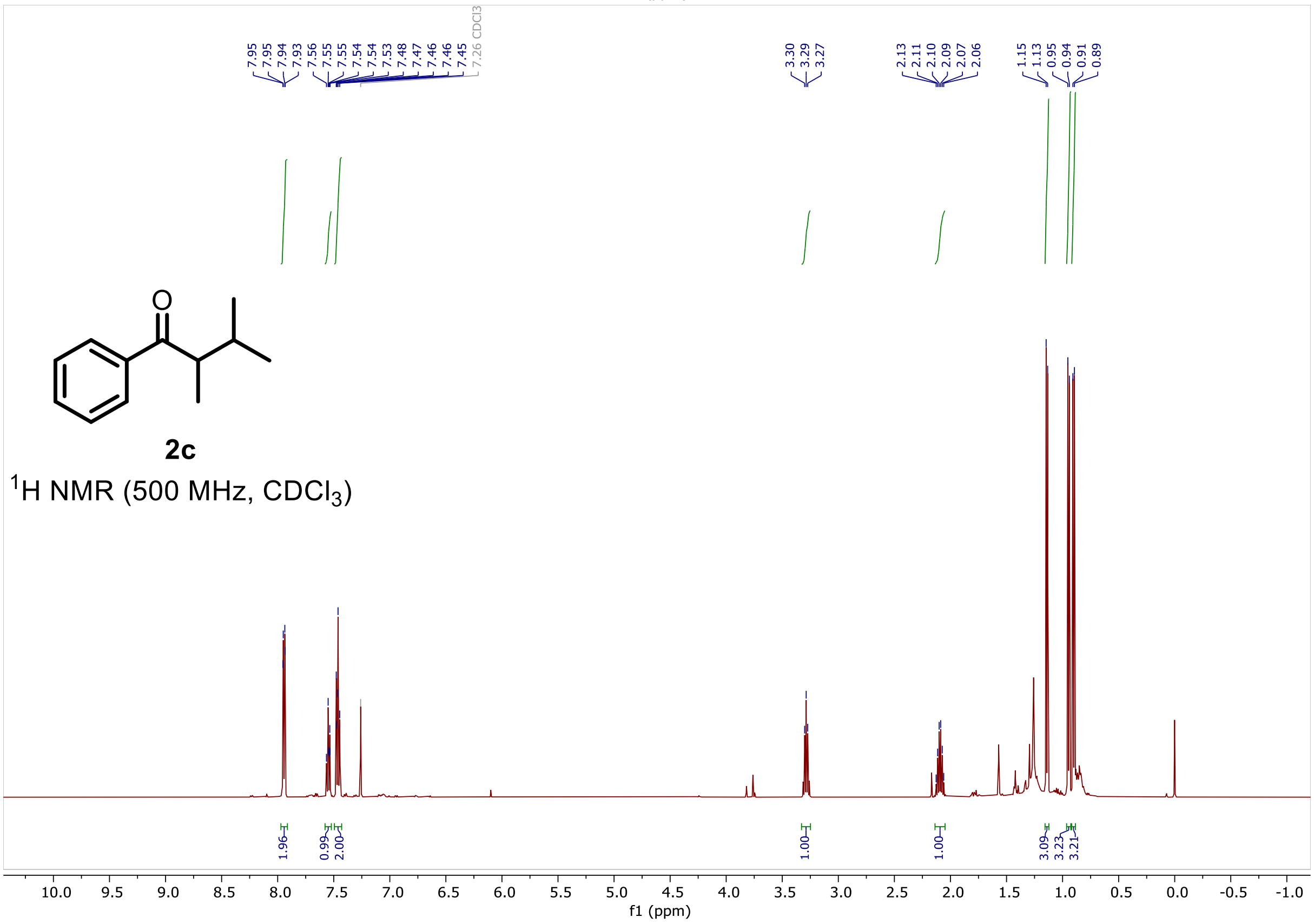
7.95
7.95
7.94
7.93
7.56
7.55
7.55
7.54
7.54
7.53
7.48
7.47
7.46
7.46
7.45
7.26 CDCl3
3.30
3.29
3.27
2.13
2.11
2.10
2.09
2.07
2.06
1.15
1.13
0.95
0.94
0.91
0.89
O
2c
1H NMR (500 MHz, CDCl3)
1.96
0.99
2.00
1.00
1.00
3.09
3.23
3.21
10.0 9.5 9.0 8.5 8.0 7.5 7.0 6.5 6.0 5.5 5.0 4.5 4.0 3.5 3.0 2.5 2.0 1.5 1.0 0.5 0.0 -0.5 -1.0
f1 (ppm)

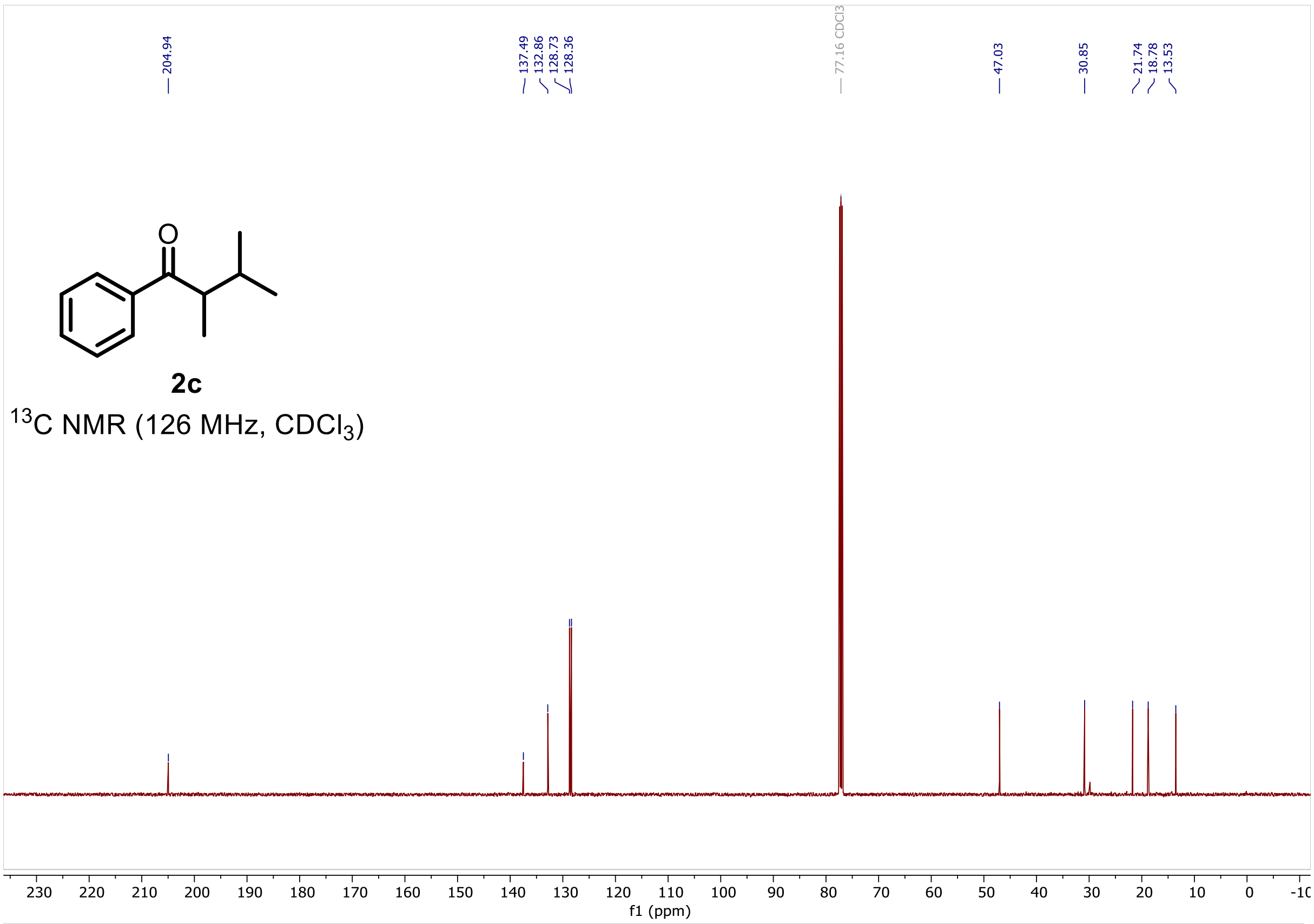

2c

^{13}C NMR (126 MHz, $CDCl_3$)

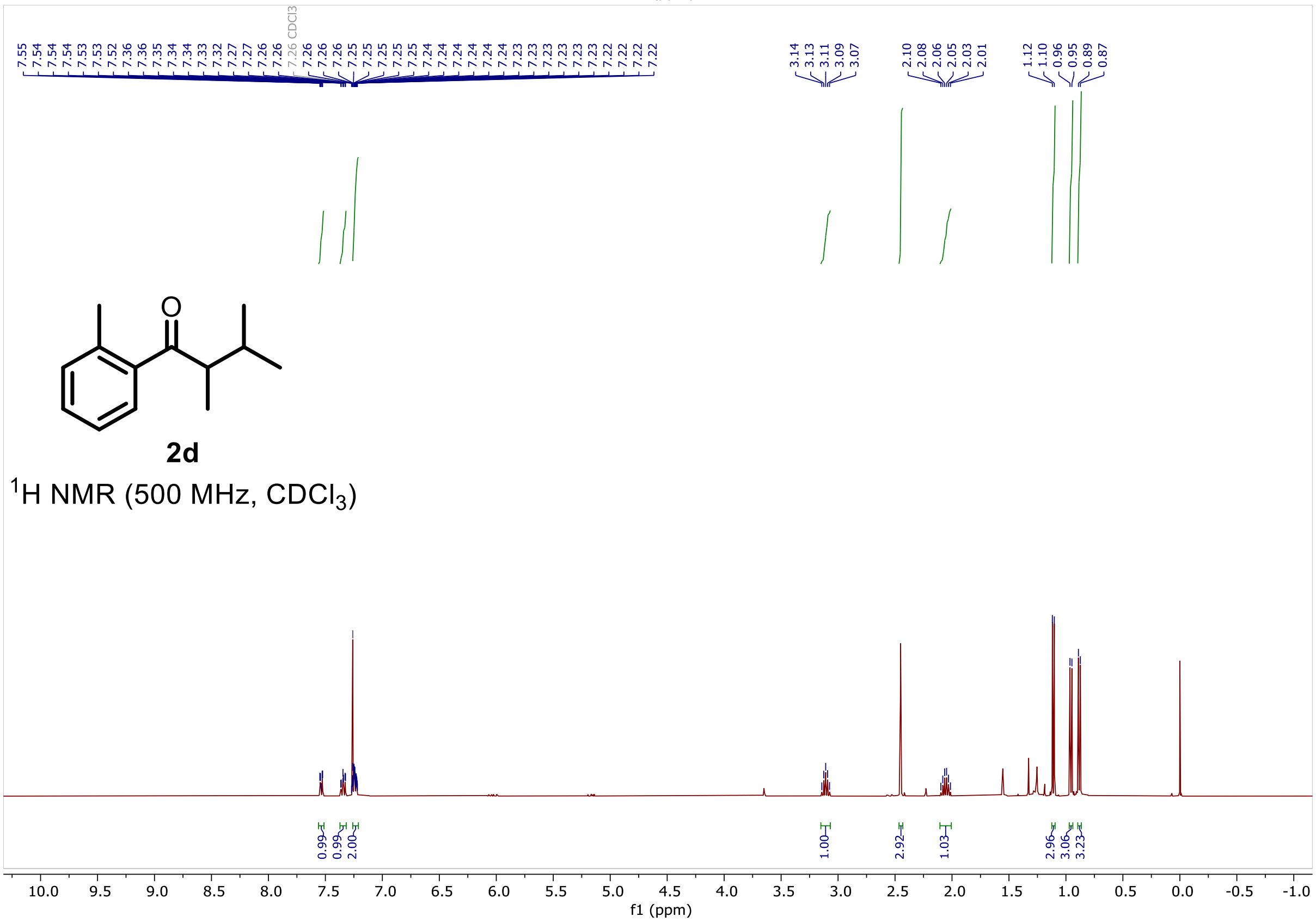

2d

^{1}H NMR (500 MHz, $CDCl_3$)

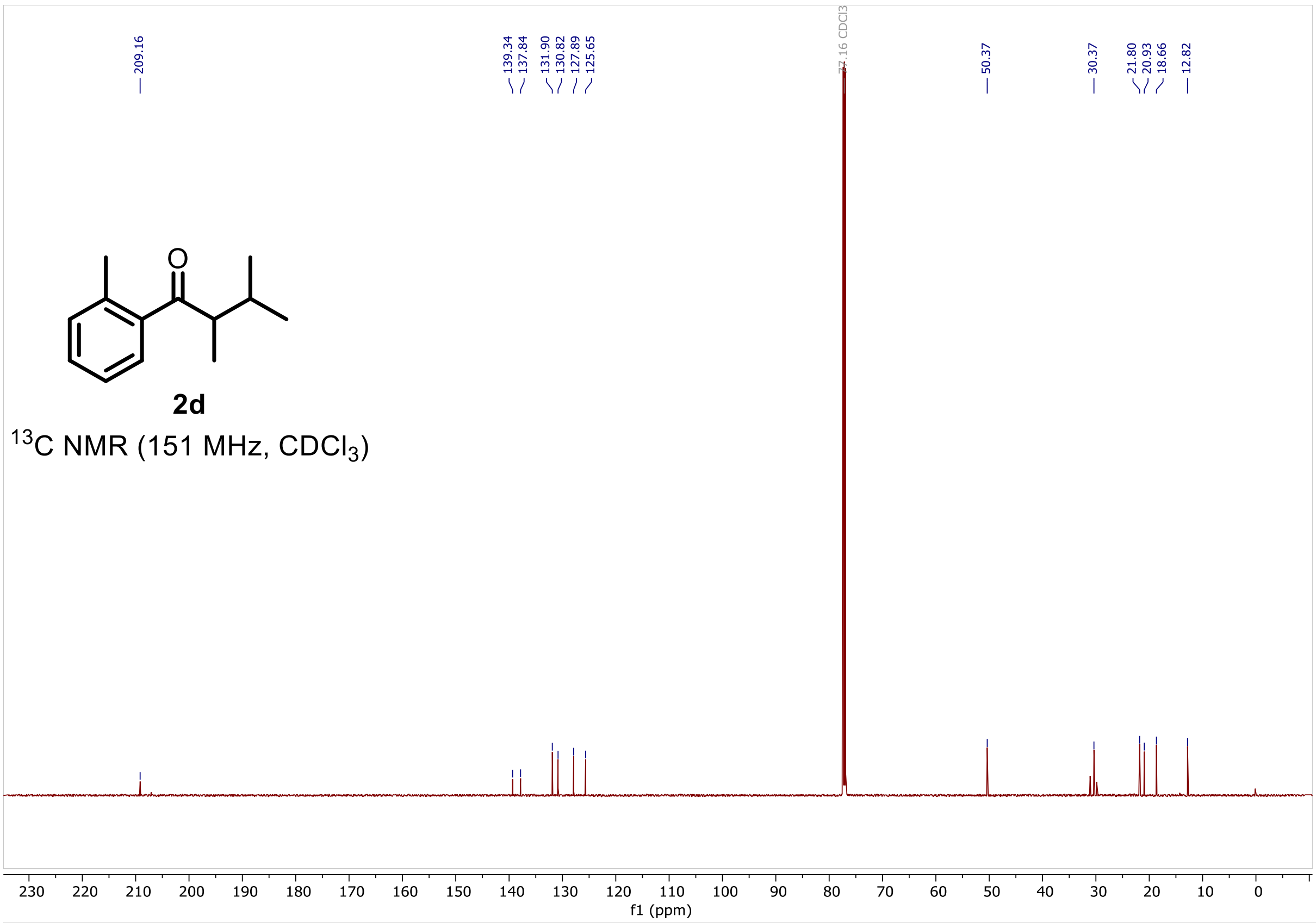

2d

^{13}C NMR (151 MHz, $CDCl_3$)

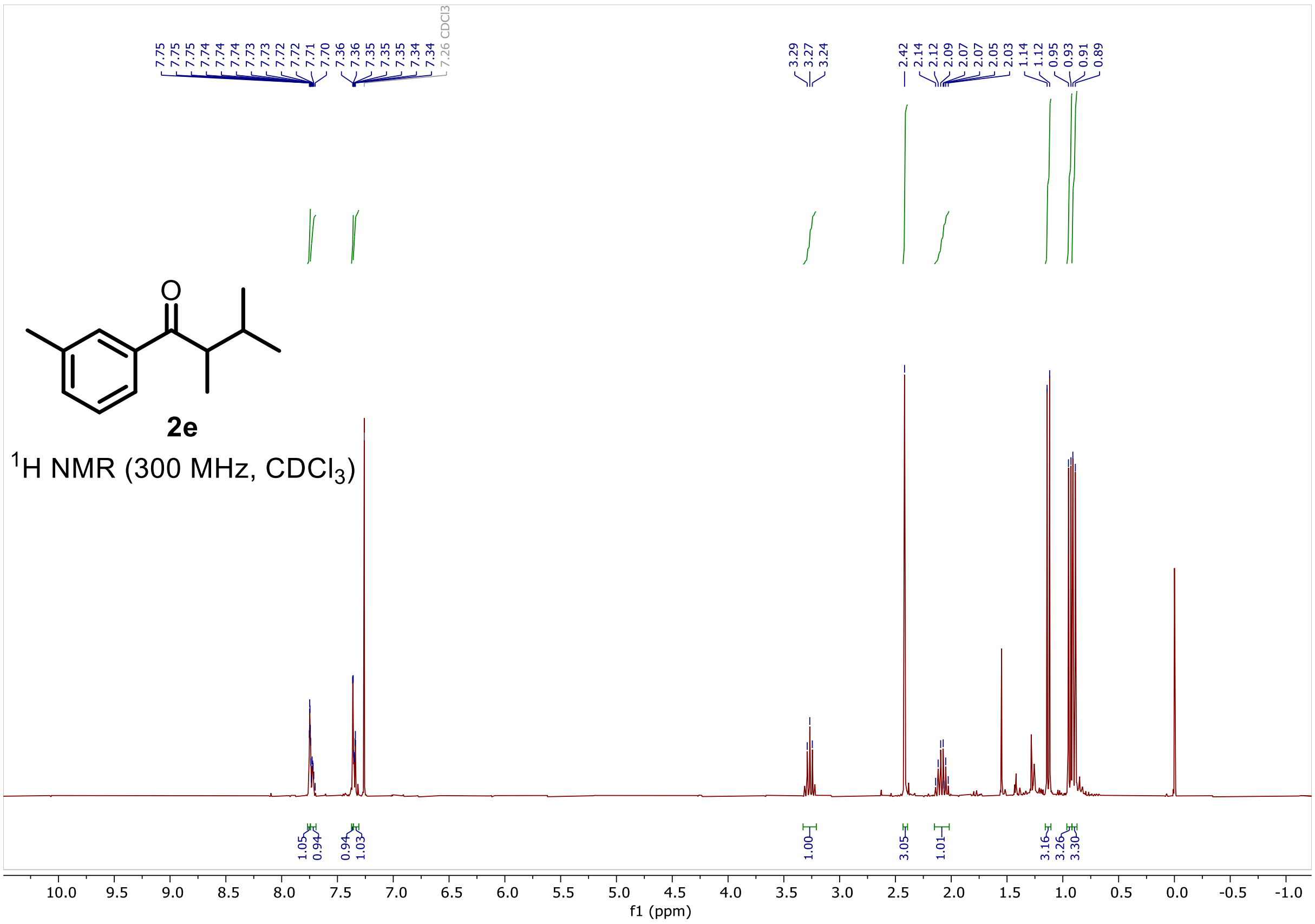

2e

^{1}H NMR (300 MHz, $CDCl_3$)

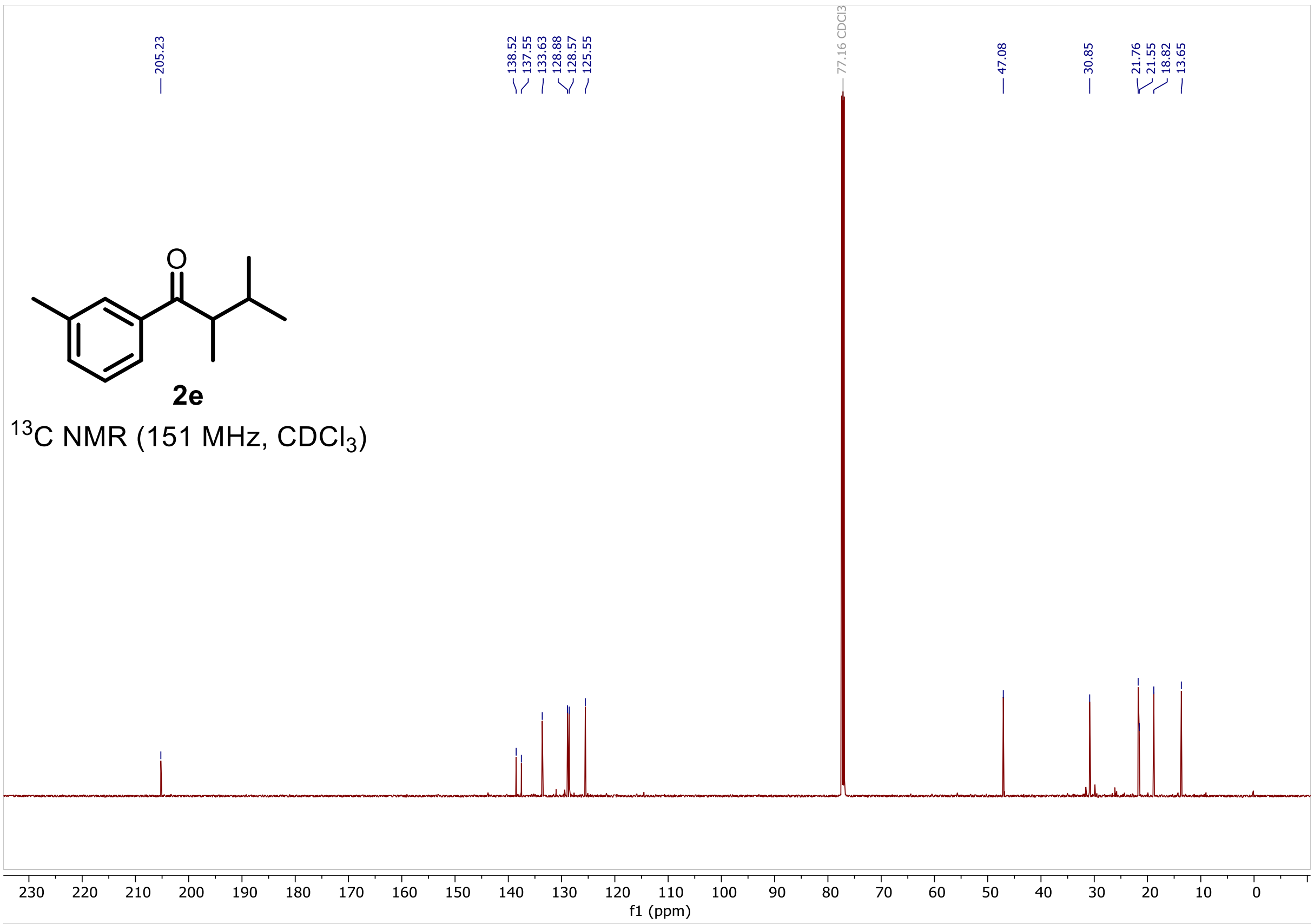
205.23
138.52
137.55
133.63
128.88
128.57
125.55
77.16 CDCl3
47.08
30.85
21.76
21.55
18.82
13.65
O
2e
^{13}C NMR (151 MHz, $CDCl_3$)
230 220 210 200 190 180 170 160 150 140 130 120 110 100 90 80 70 60 50 40 30 20 10 0
f1 (ppm)

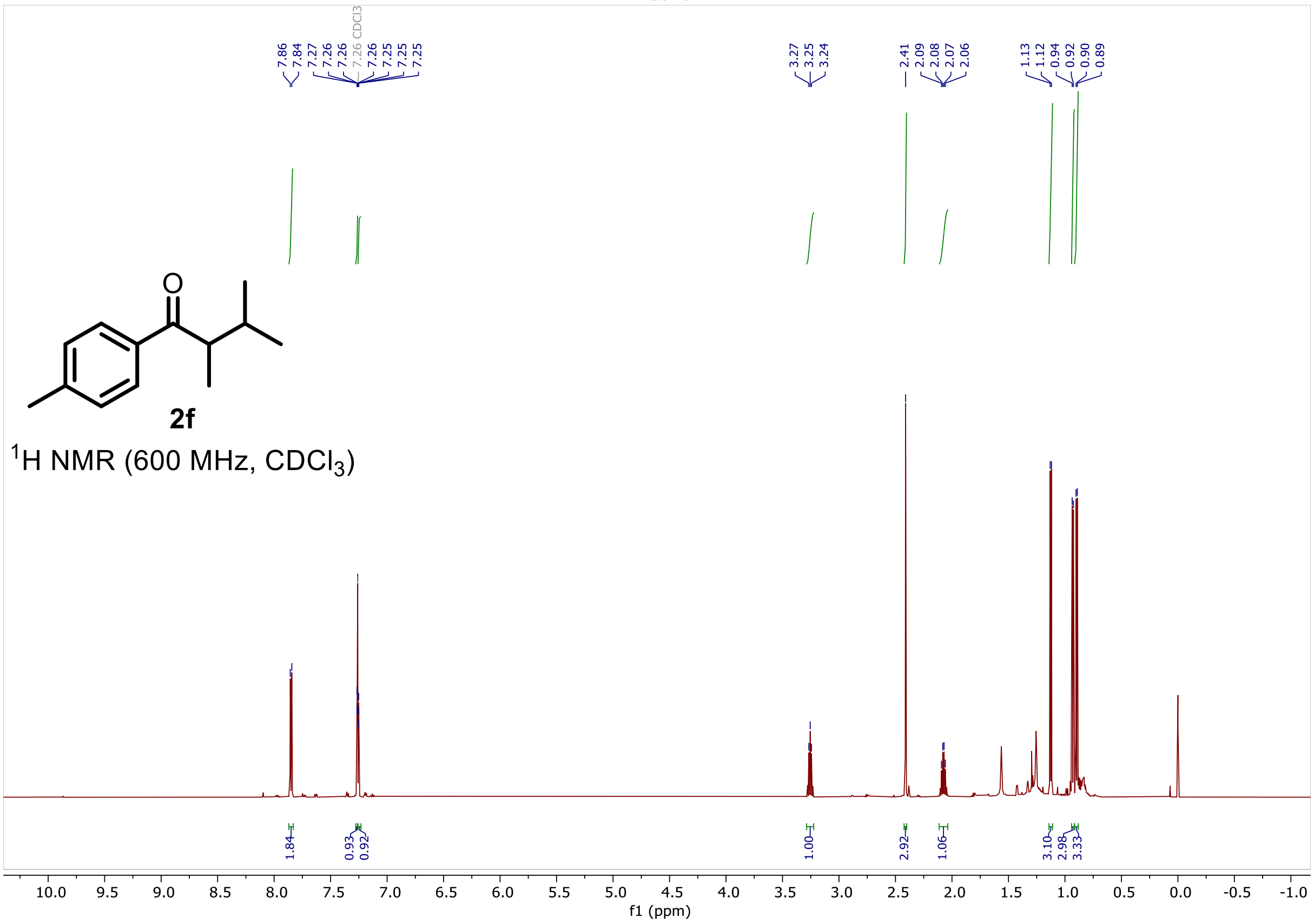
7.86
7.84
7.27
7.26
7.26
7.26 CDCl3
7.26
7.25
7.25
7.25
3.27
3.25
3.24
2.41
2.09
2.08
2.07
2.06
1.13
1.12
0.94
0.92
0.90
0.89
O
2f
^{1}H NMR (600 MHz, $CDCl_3$)
1.84
0.93
0.92
1.00
2.92
1.06
3.10
2.98
3.33
10.0 9.5 9.0 8.5 8.0 7.5 7.0 6.5 6.0 5.5 5.0 4.5 4.0 3.5 3.0 2.5 2.0 1.5 1.0 0.5 0.0 -0.5 -1.0
f1 (ppm)

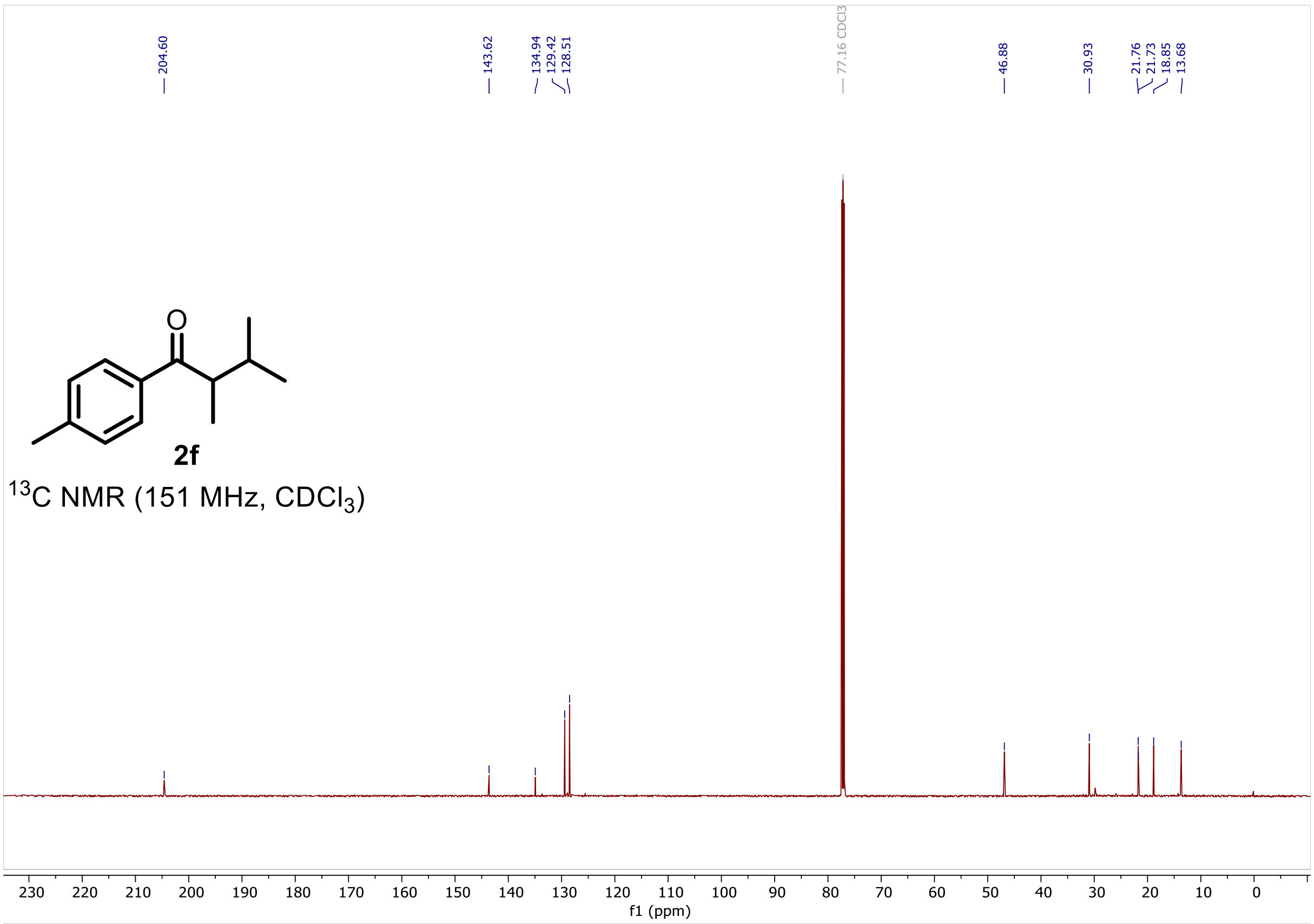
204.60
143.62
134.94
129.42
128.51
77.16 CDCl3
46.88
30.93
21.76
21.73
18.85
13.68
O
2f
13C NMR (151 MHz, CDCl3)
f1 (ppm)

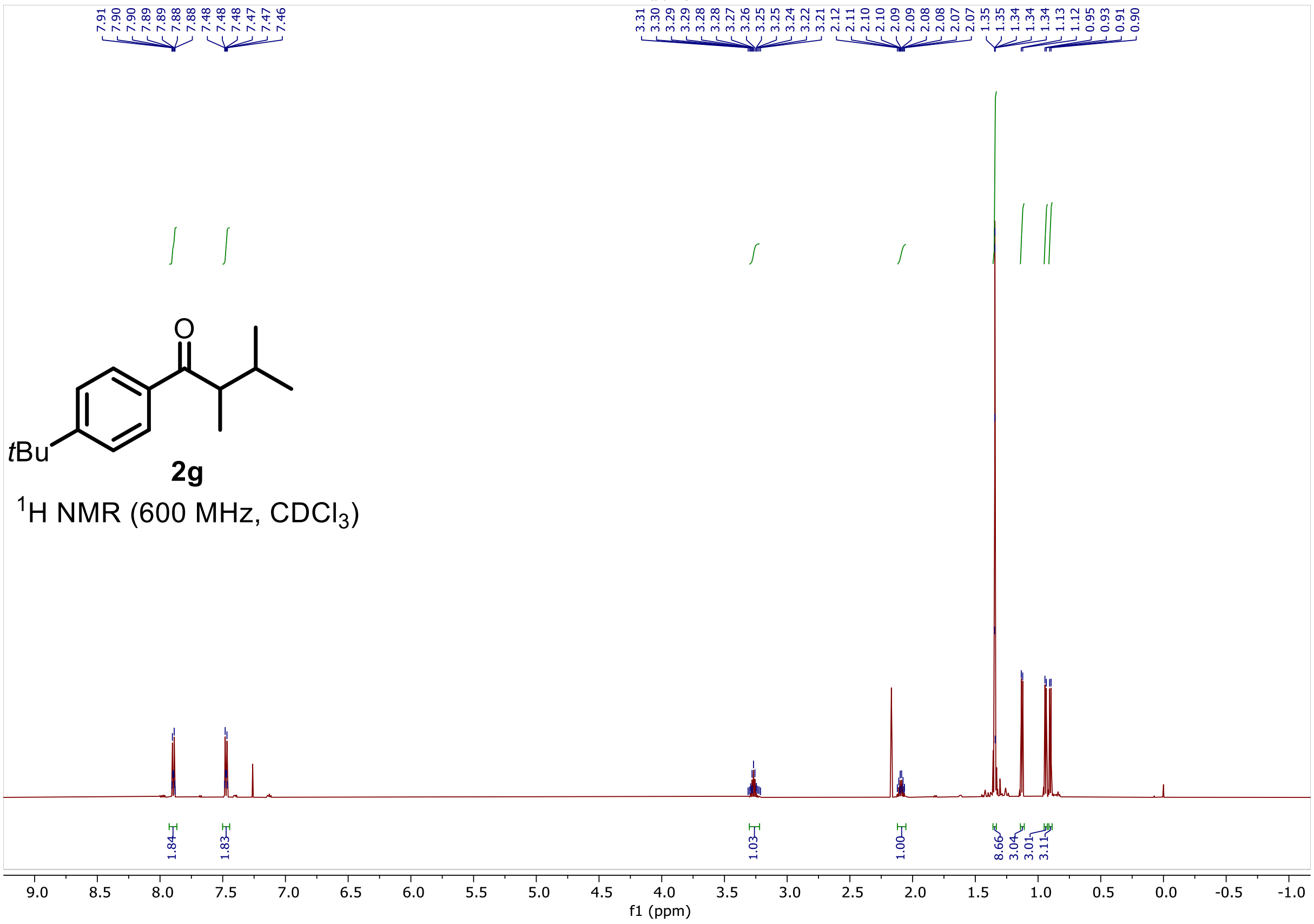
7.91 7.90 7.90 7.89 7.89 7.88 7.88 7.48 7.48 7.48 7.47 7.47 7.46
3.31 3.30 3.29 3.29 3.28 3.28 3.27 3.26 3.25 3.25 3.24 3.22 3.21
2.12 2.11 2.10 2.10 2.09 2.09 2.08 2.08 2.07 2.07
1.35 1.35 1.34 1.34 1.34 1.13 1.12 0.95 0.93 0.91 0.90
O
tBu
2g
1H NMR (600 MHz, CDCl3)
1.84
1.83
1.03
1.00
8.66
3.04
3.01
3.11
f1 (ppm)

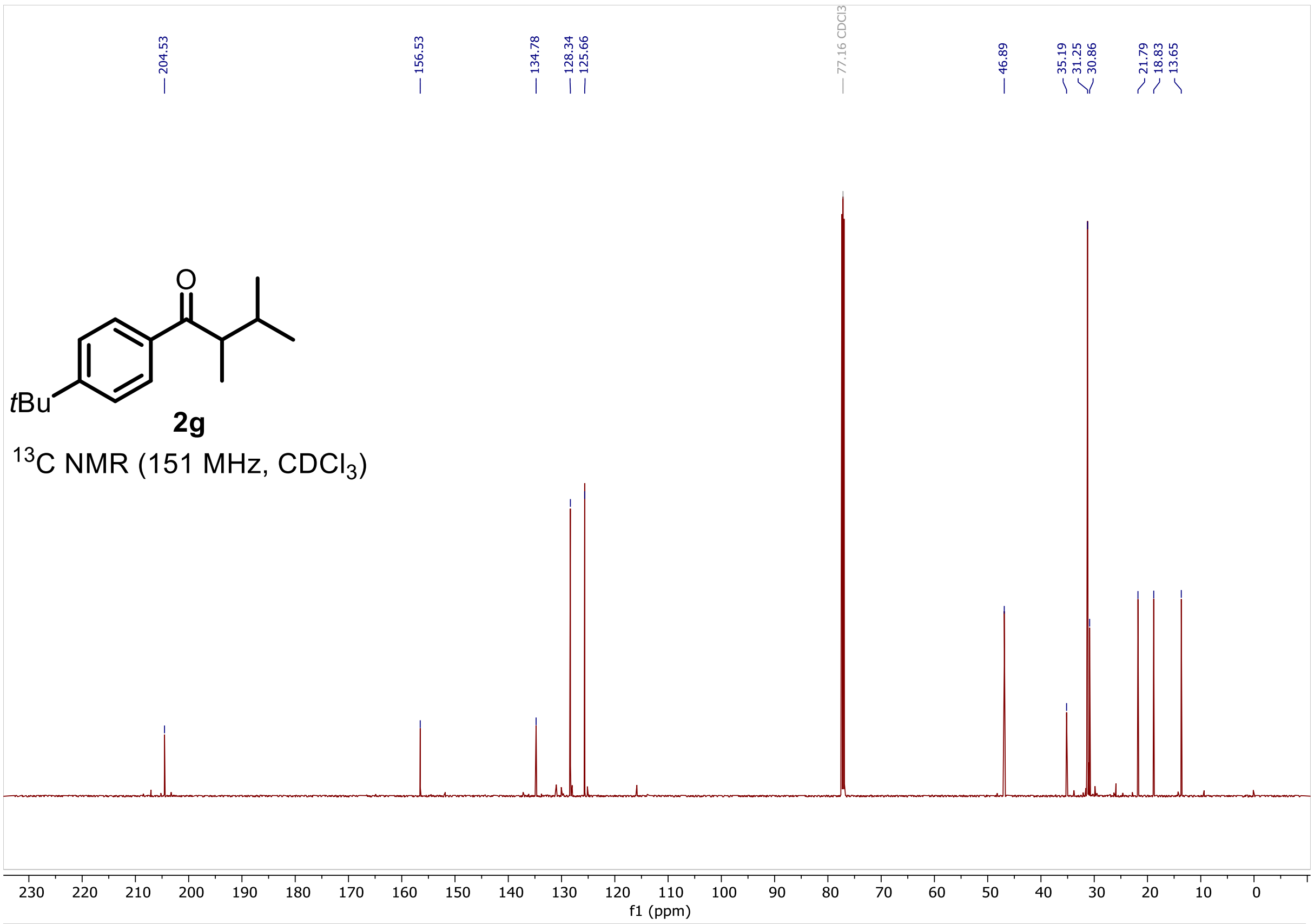

204.53
156.53
134.78
128.34
125.66
77.16 CDCl3
46.89
35.19
31.25
30.86
21.79
18.83
13.65
O
tBu
2g
^{13}C NMR (151 MHz, CDCl$_3$)
f1 (ppm)

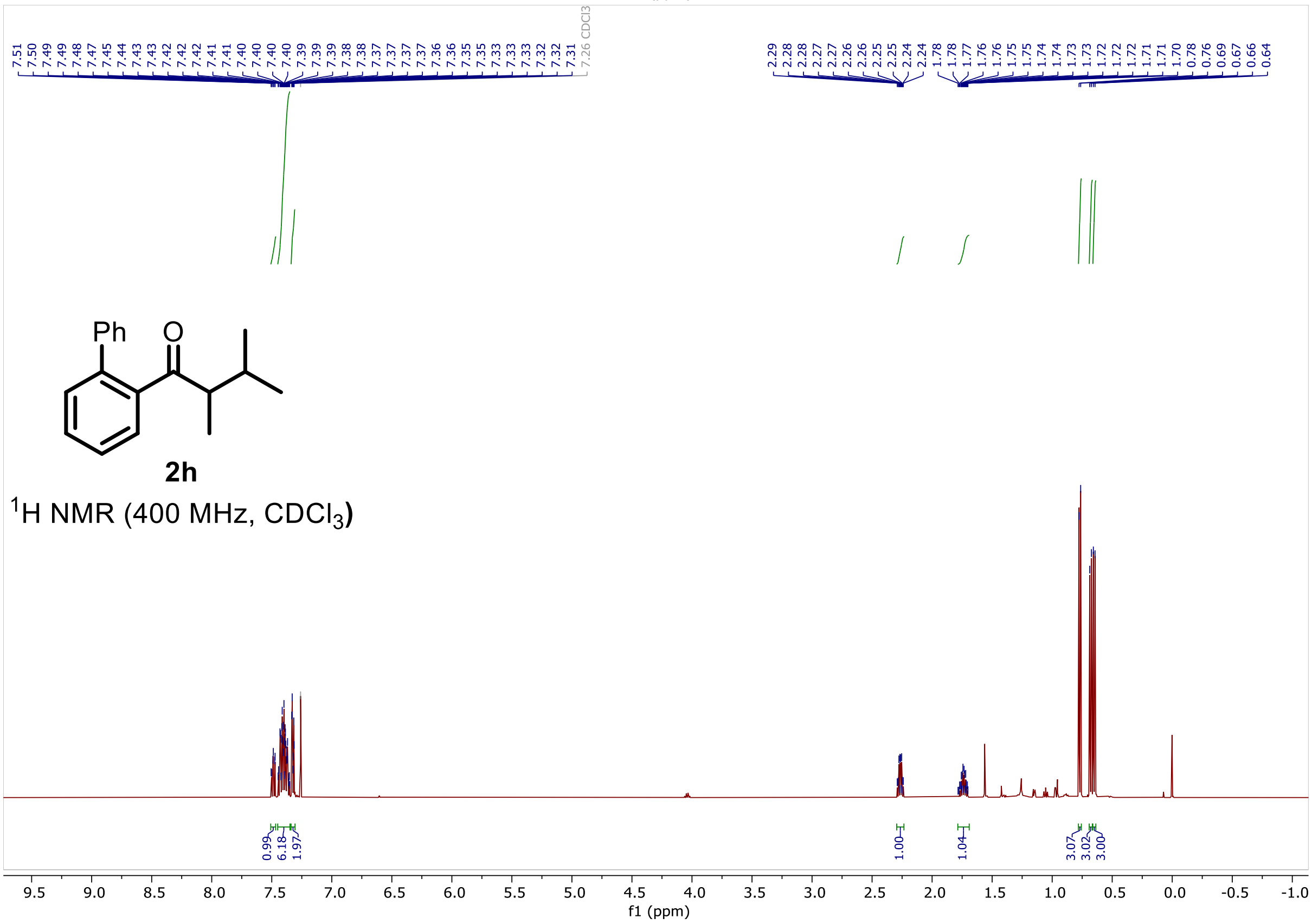

7.26 CDCl3
Ph
O
2h
^{1}H NMR (400 MHz, CDCl$_3$)
0.99
6.18
1.97
1.00
1.04
3.07
3.02
3.00
f1 (ppm)

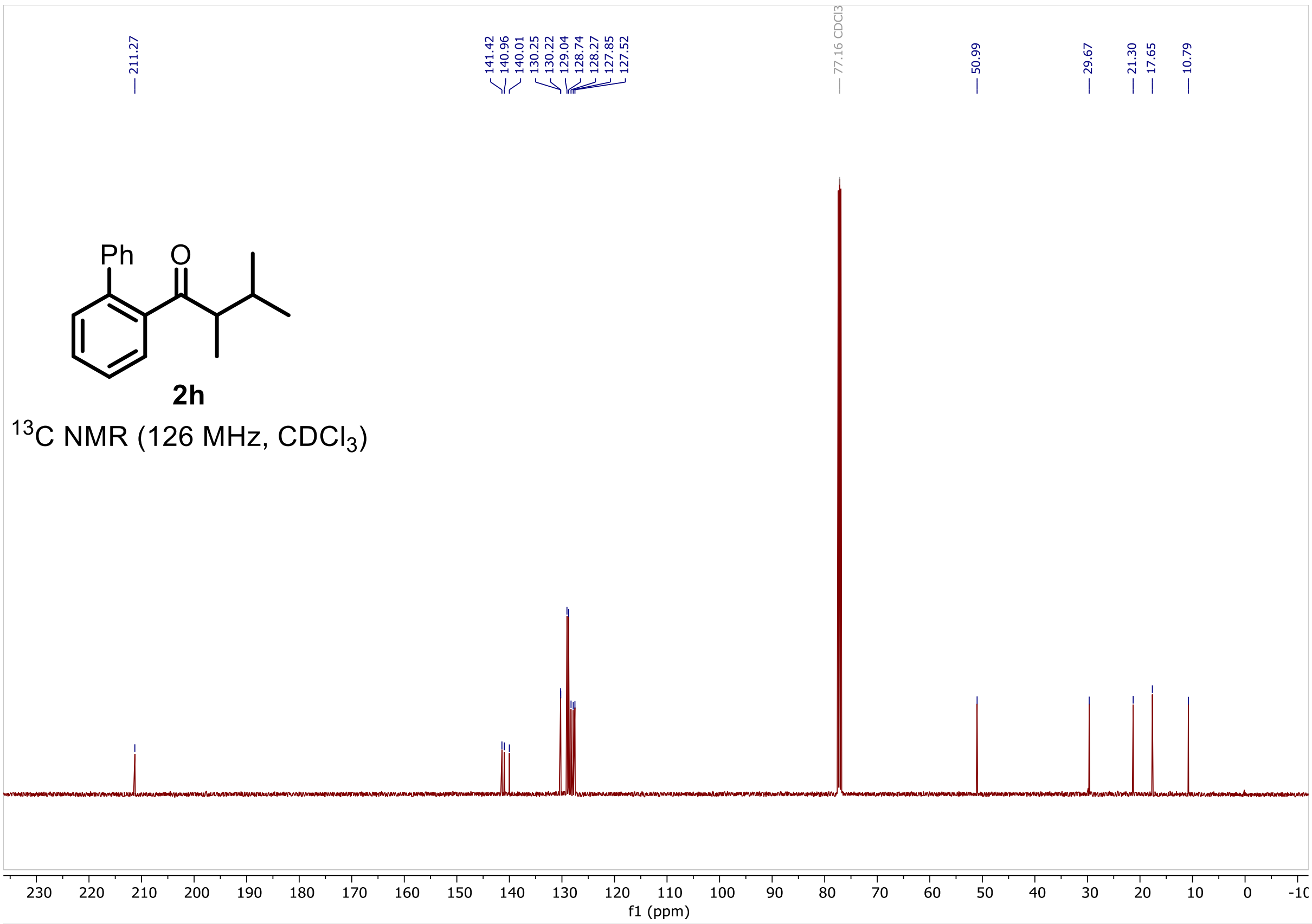

2h

^{13}C NMR (126 MHz, $CDCl_3$)

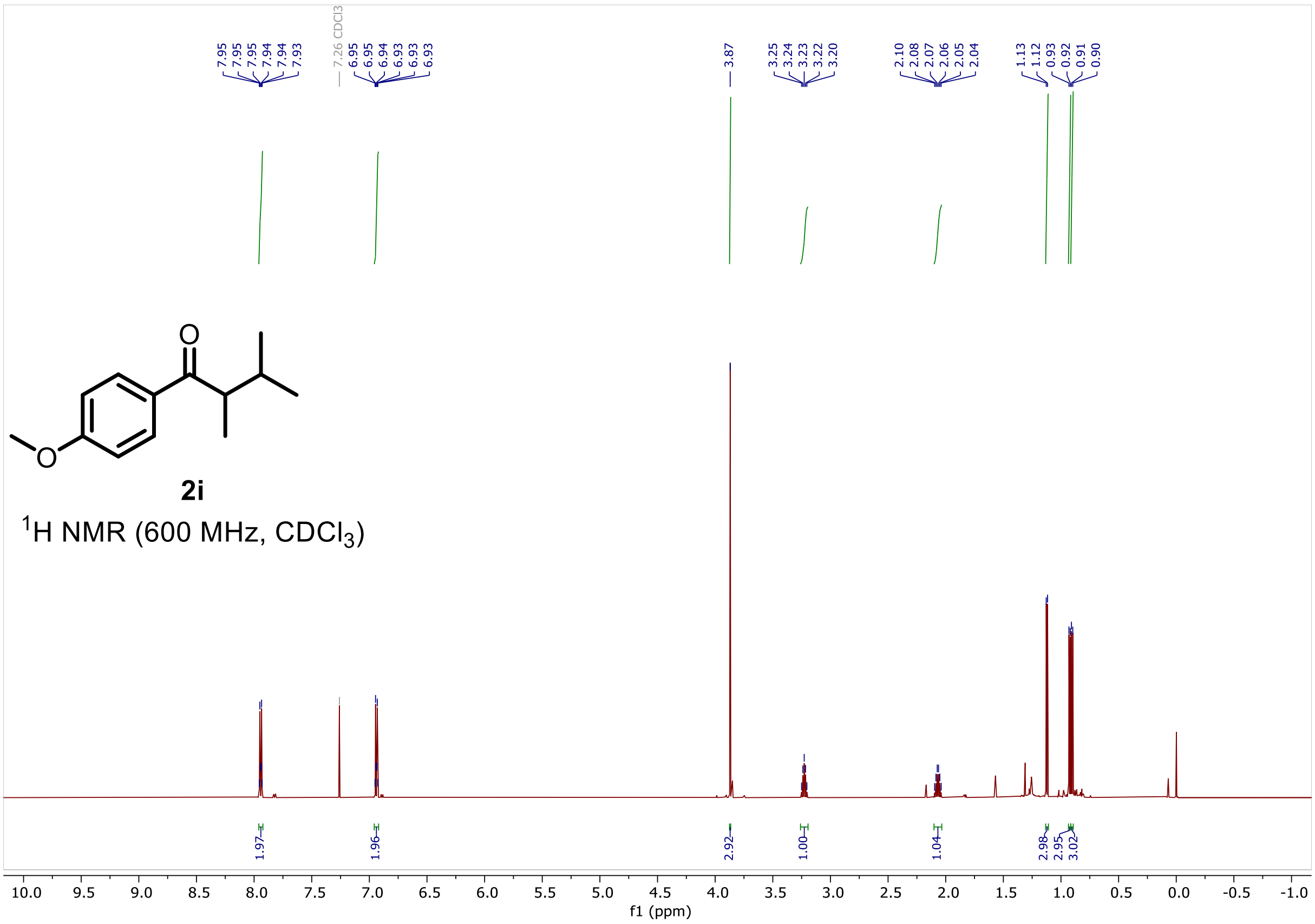

2i

^{1}H NMR (600 MHz, $CDCl_3$)

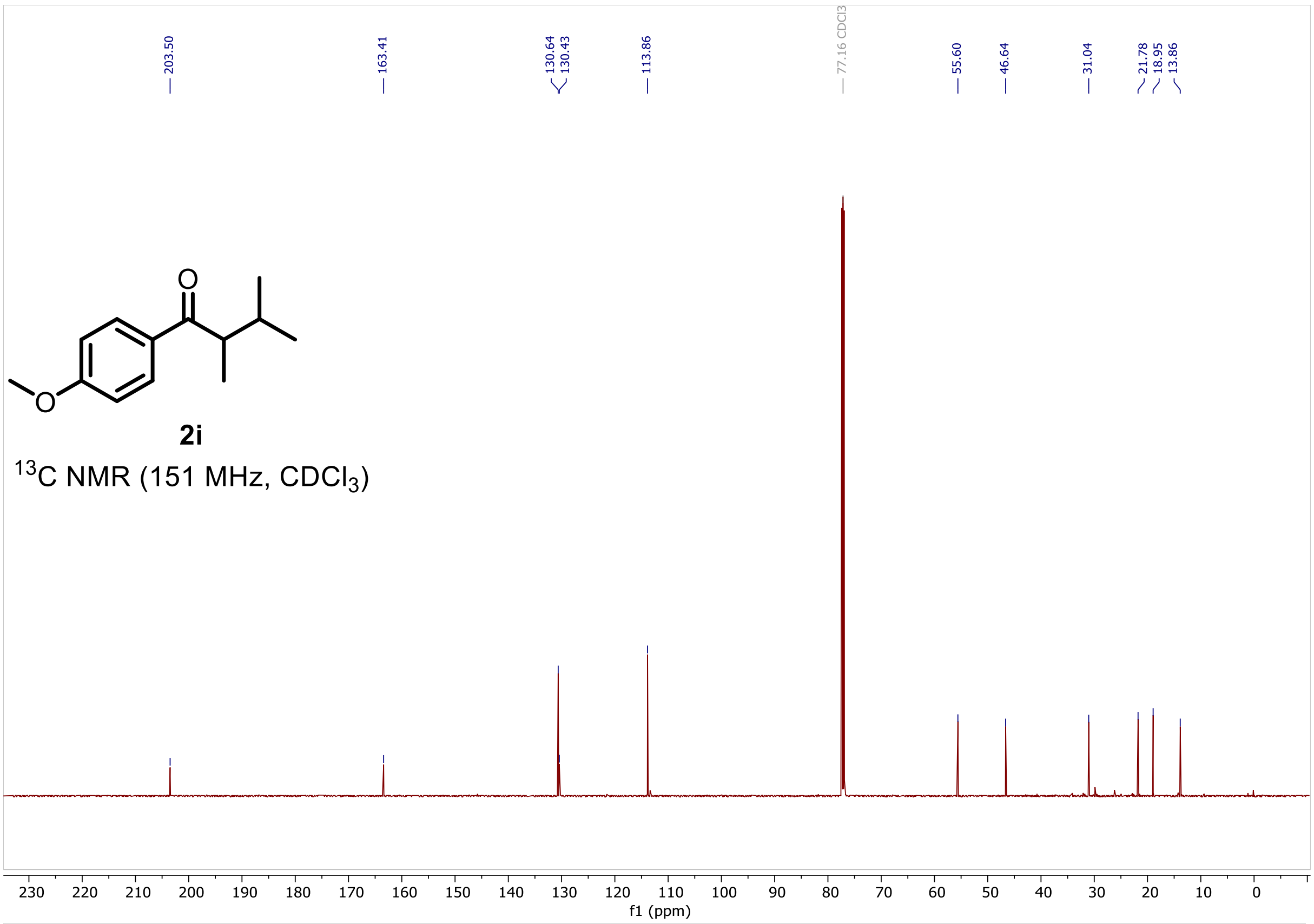

2i

^{13}C NMR (151 MHz, $CDCl_3$)

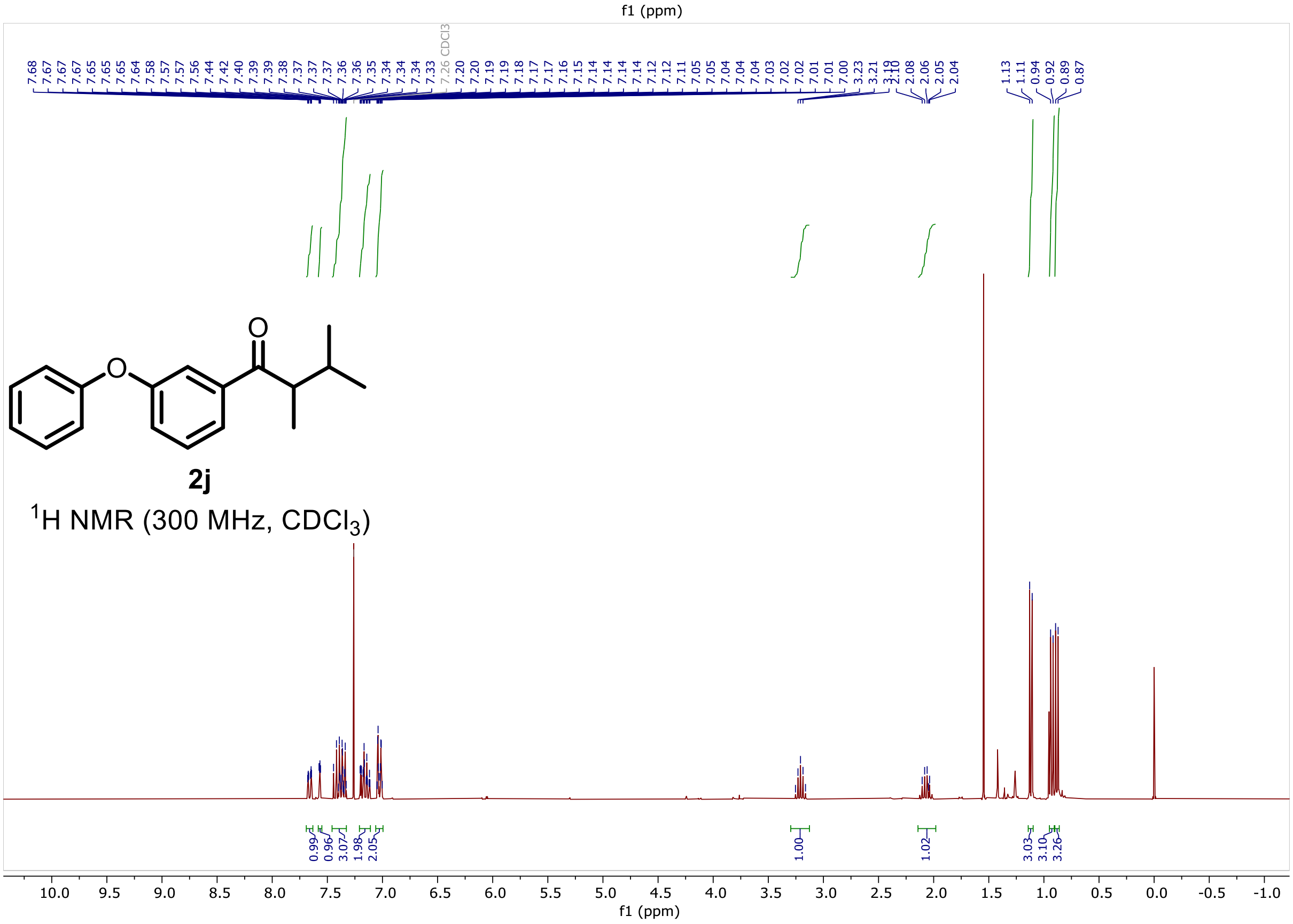

2j

^{1}H NMR (300 MHz, $CDCl_3$)

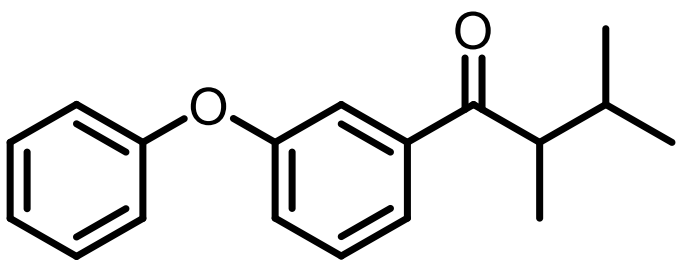

2j

^{13}C NMR (151 MHz, $CDCl_3$)

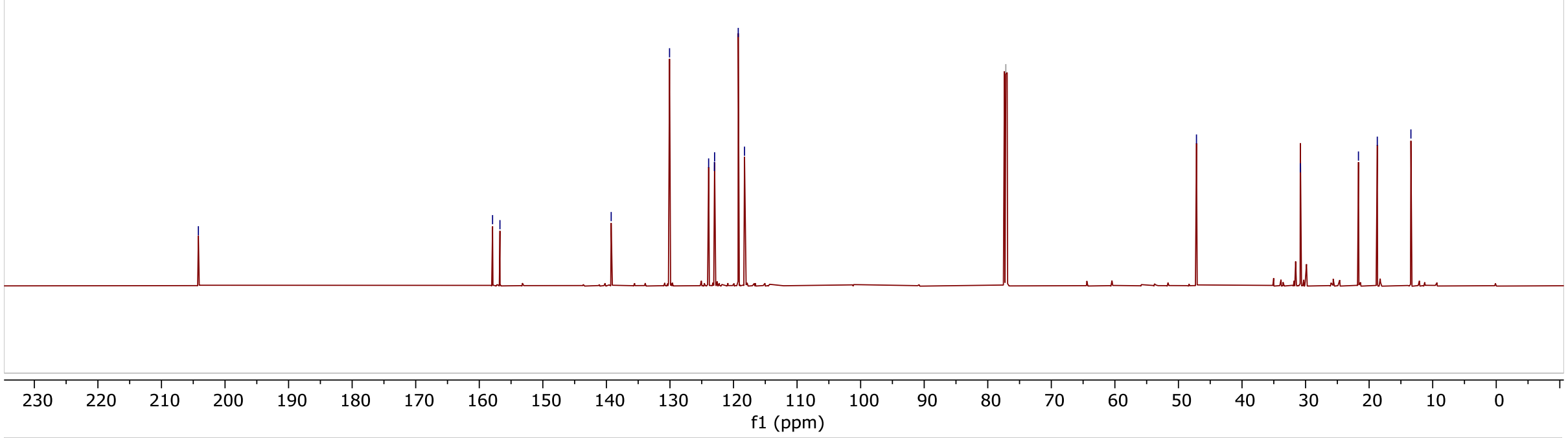

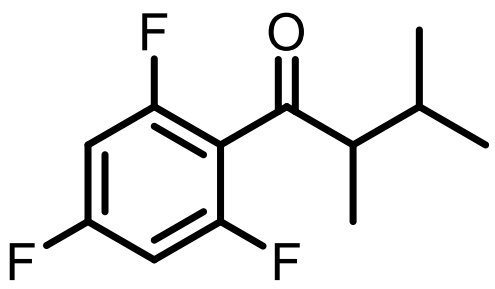

2k

^{1}H NMR (300 MHz, $CDCl_3$)

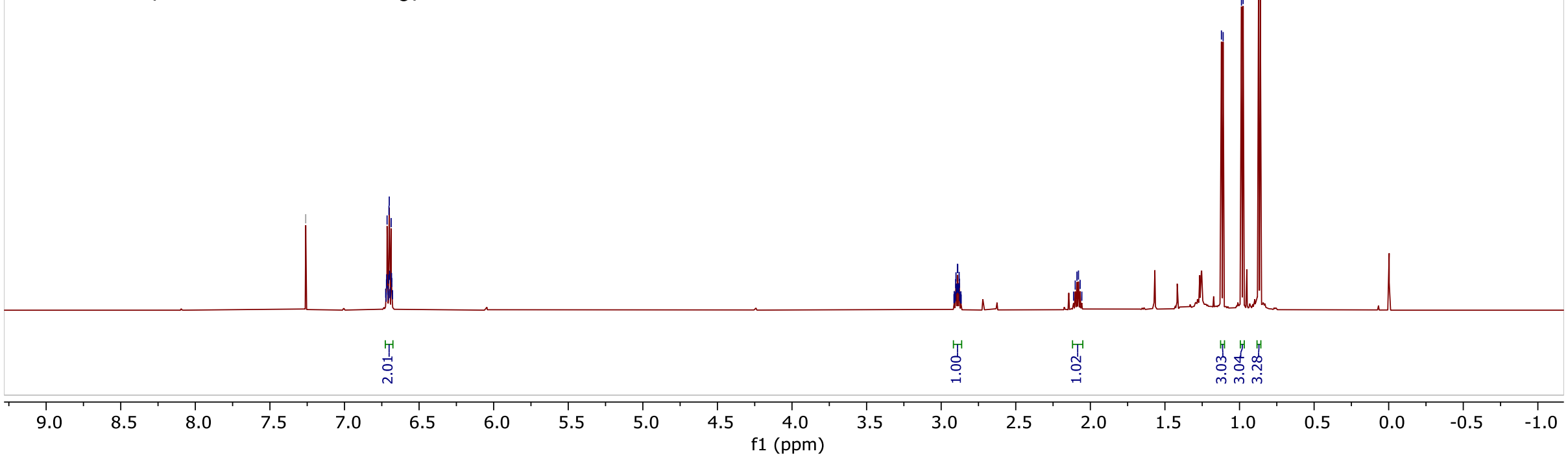

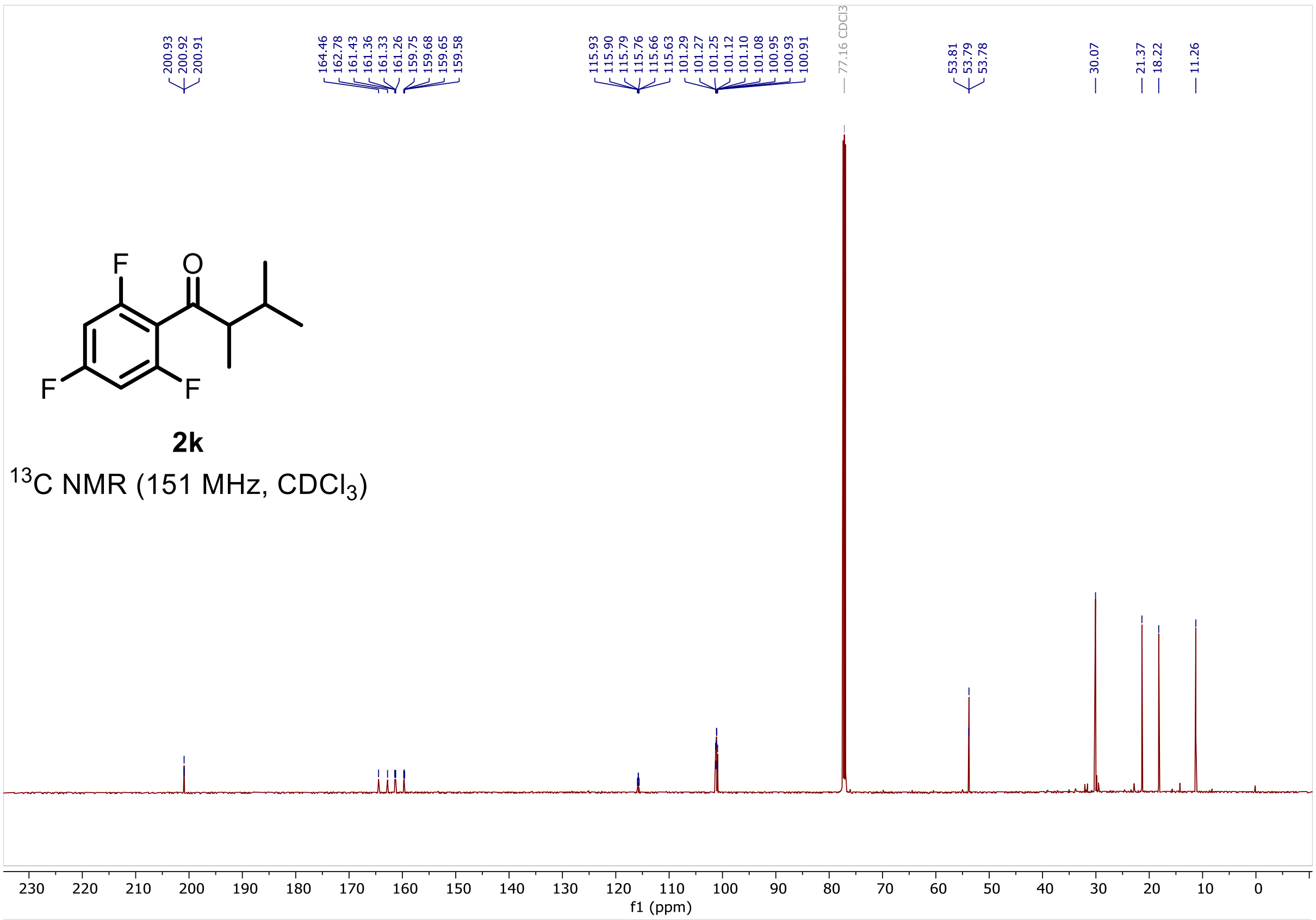

2k

^{13}C NMR (151 MHz, $CDCl_3$)

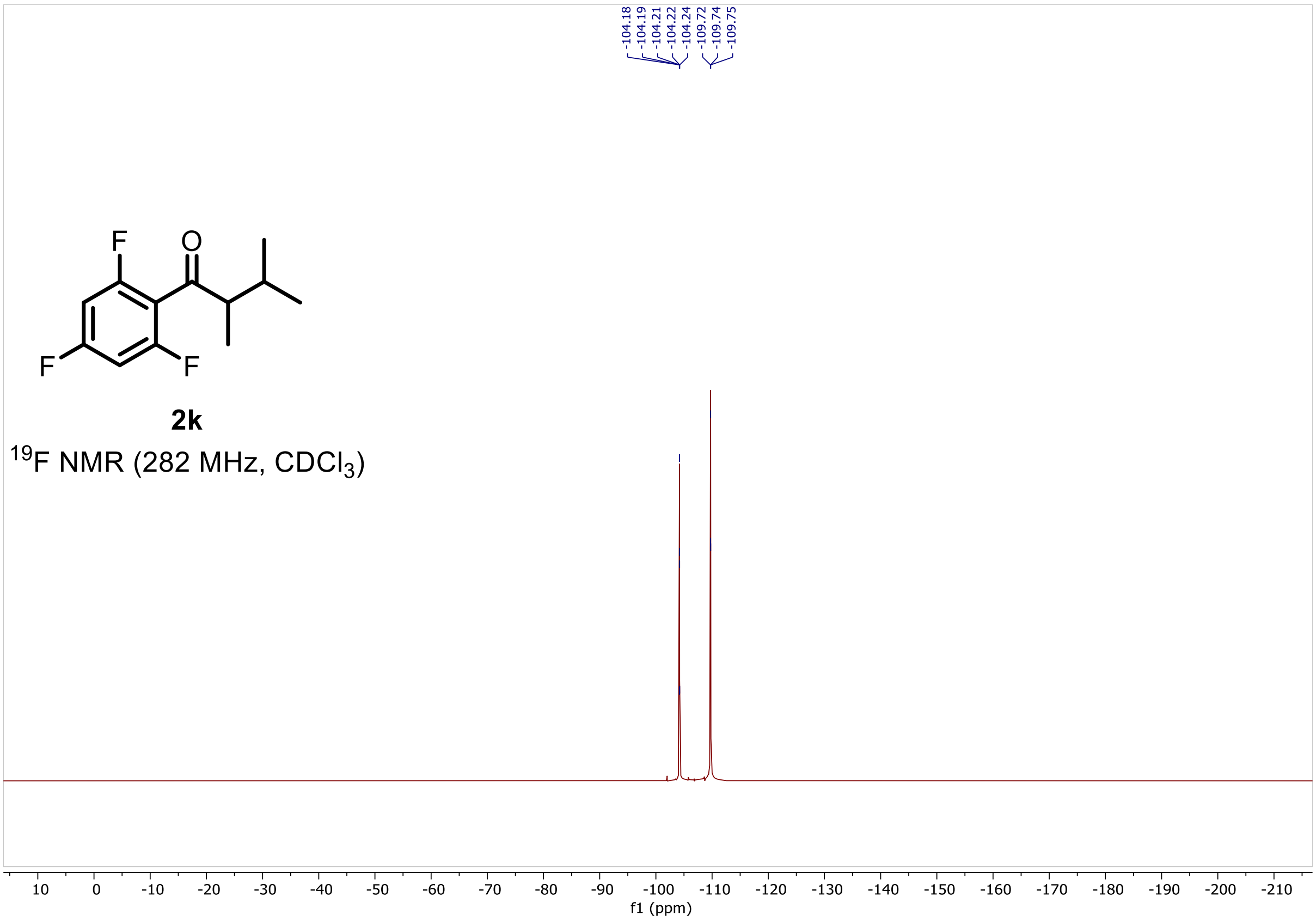

2k

^{19}F NMR (282 MHz, $CDCl_3$)

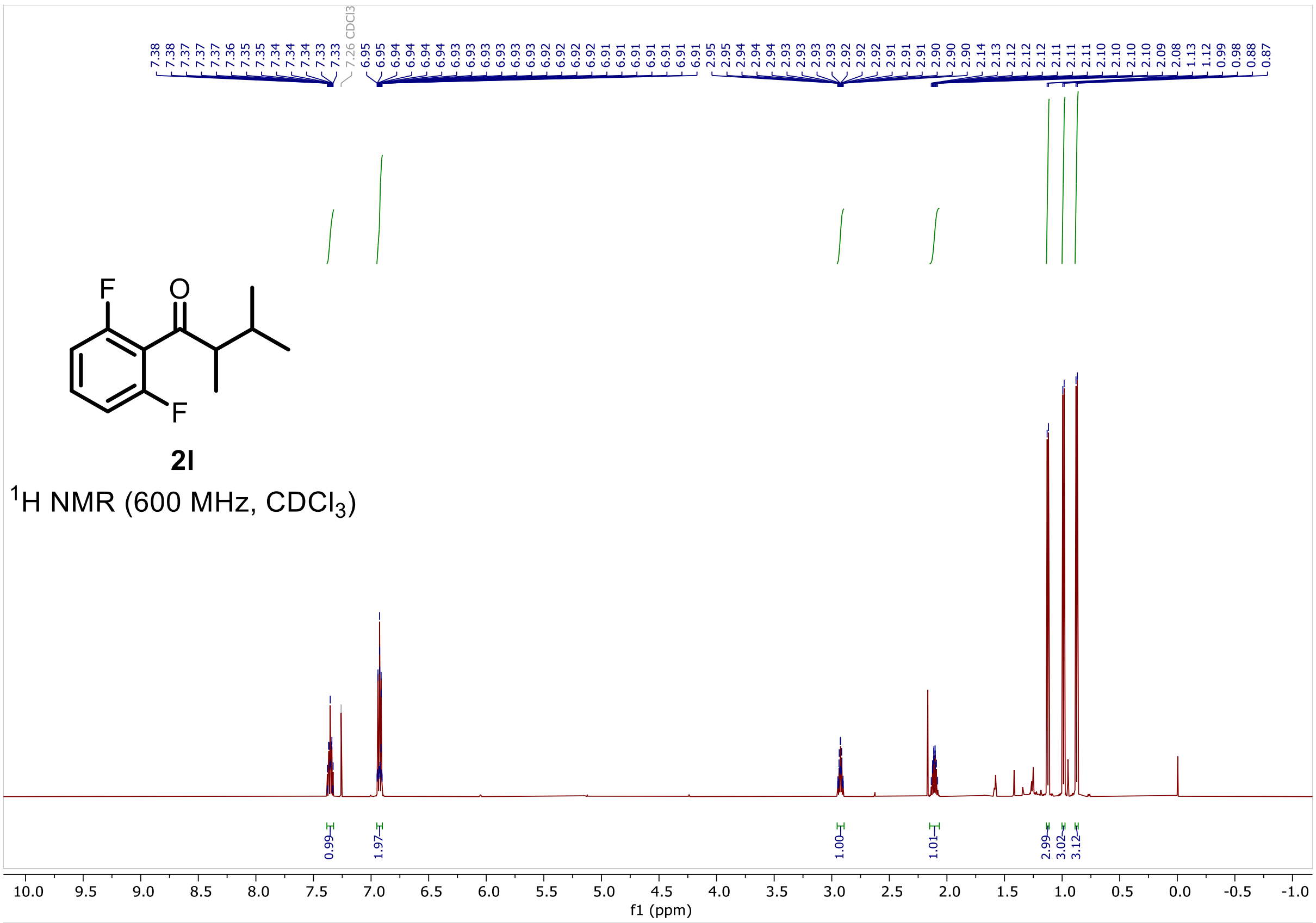
2l
1H NMR (600 MHz, CDCl3)
f1 (ppm)

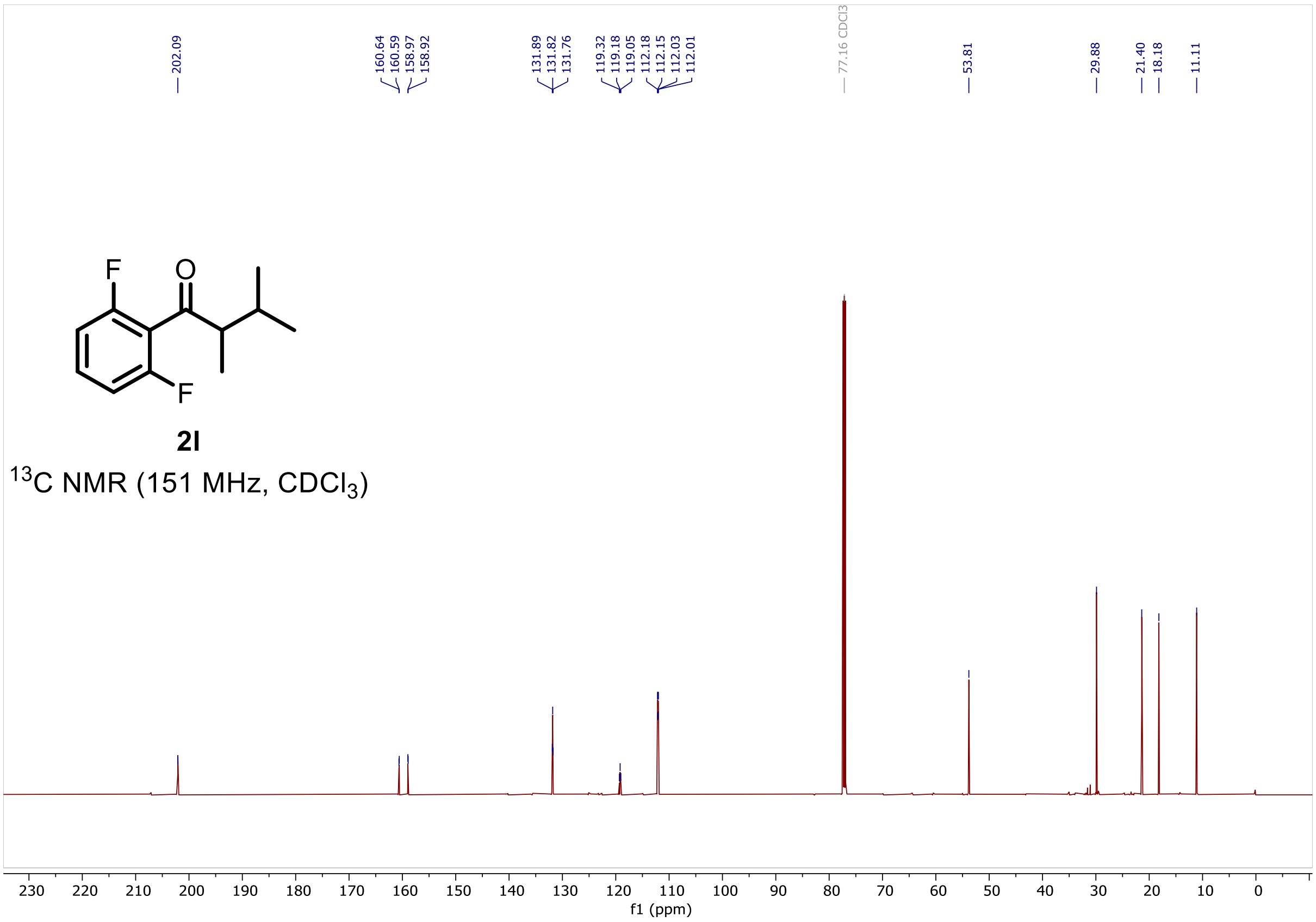
202.09
160.64
160.59
158.97
158.92
131.89
131.82
131.76
119.32
119.18
119.05
112.18
112.15
112.03
112.01
77.16 CDCl3
53.81
29.88
21.40
18.18
11.11
2l
13C NMR (151 MHz, CDCl3)
f1 (ppm)

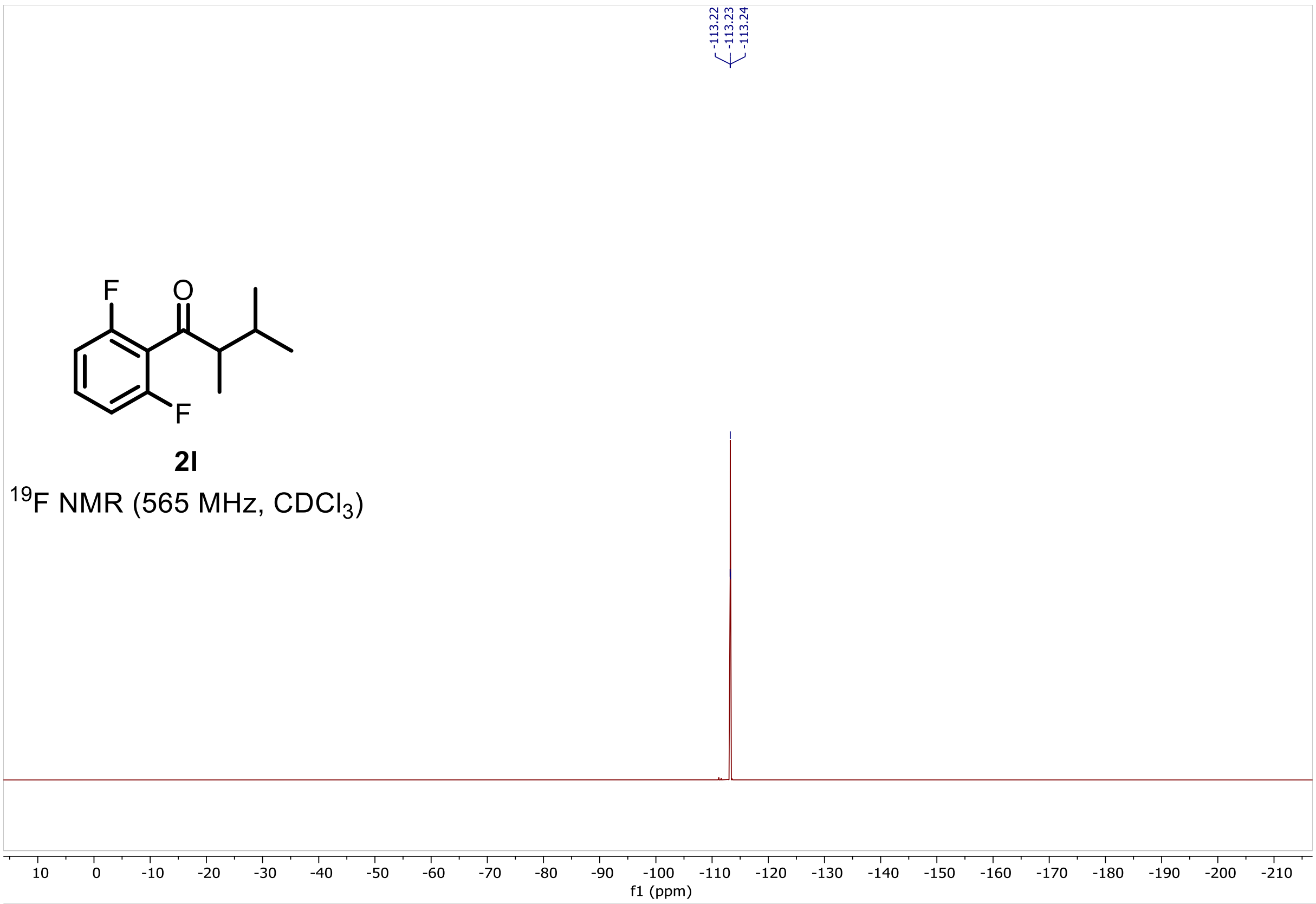

2l

^{19}F NMR (565 MHz, $CDCl_3$)

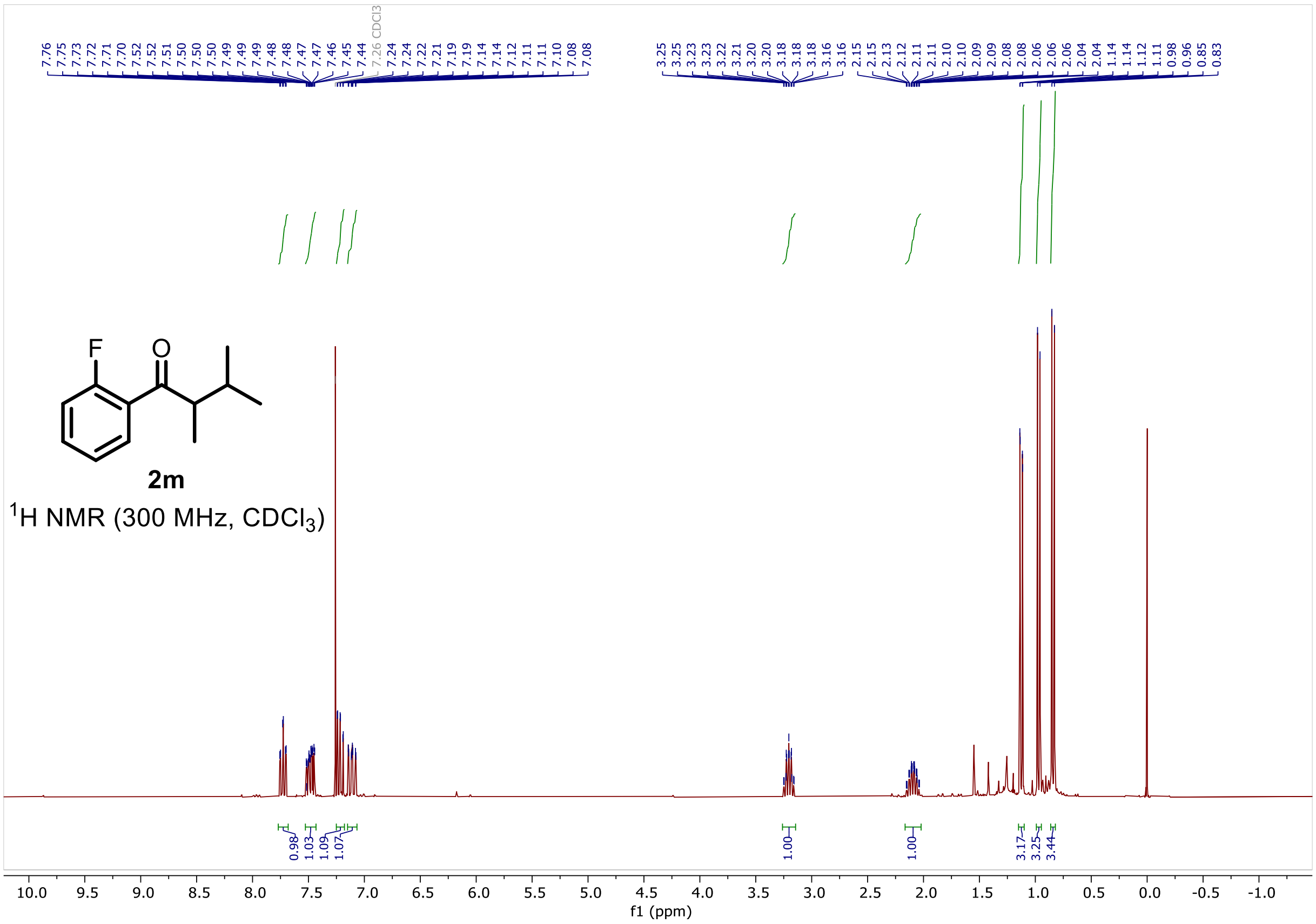

2m

^{1}H NMR (300 MHz, $CDCl_3$)

v

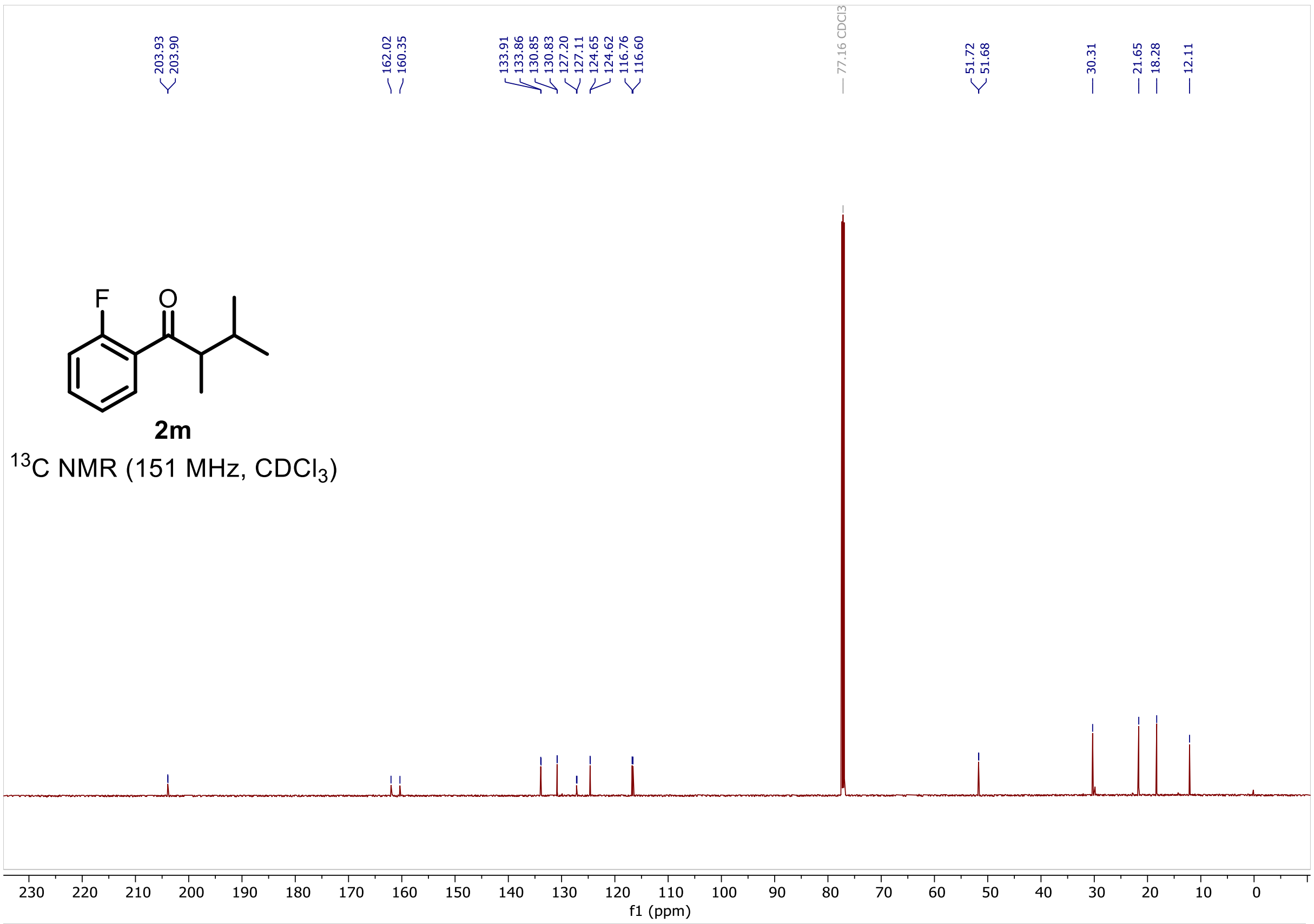
203.93
203.90
162.02
160.35
133.91
133.86
130.85
130.83
127.20
127.11
124.65
124.62
116.76
116.60
77.16 CDCl3
51.72
51.68
30.31
21.65
18.28
12.11
F
O
2m
13C NMR (151 MHz, CDCl3)
230 220 210 200 190 180 170 160 150 140 130 120 110 100 90 80 70 60 50 40 30 20 10 0
f1 (ppm)

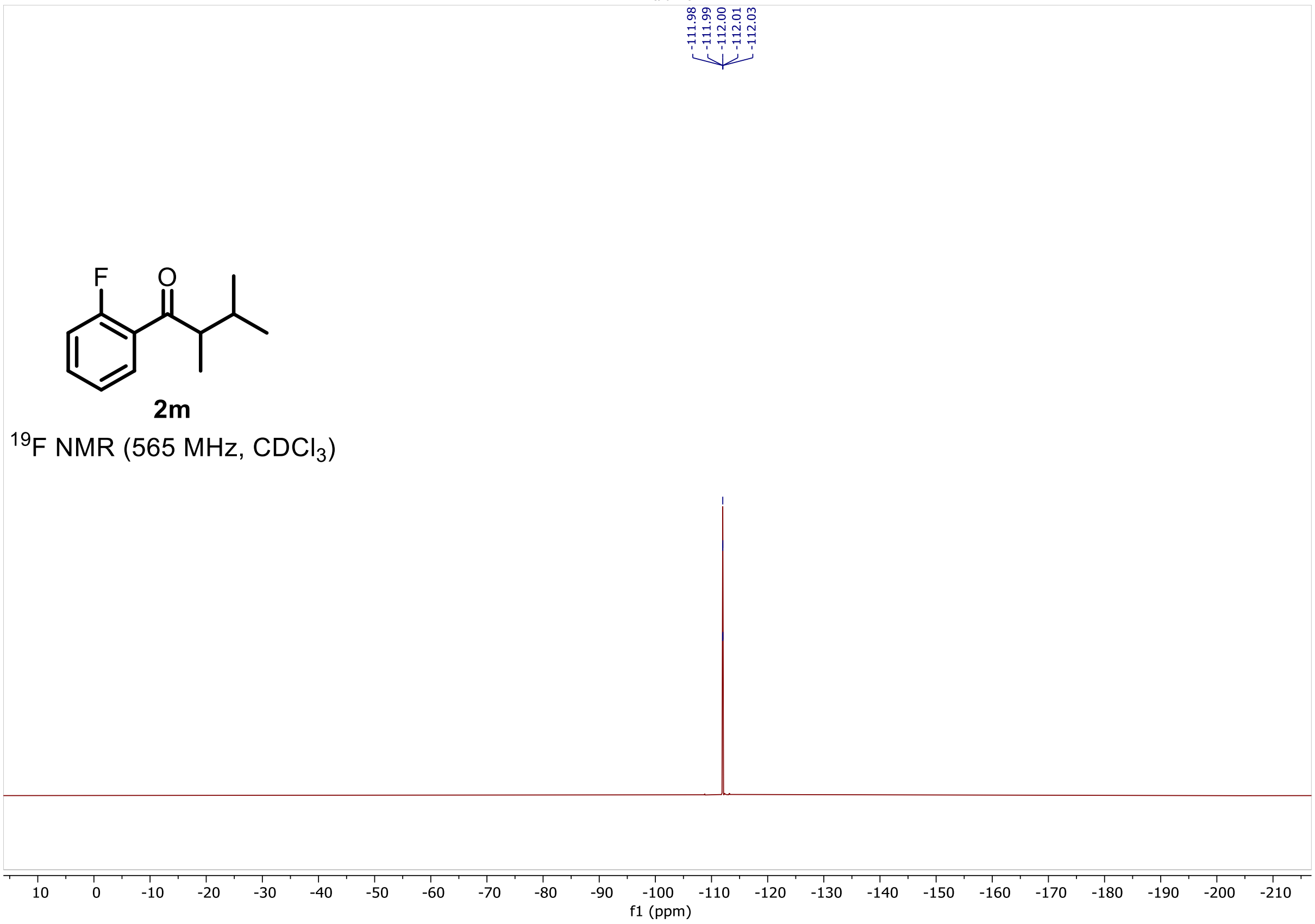
-111.98
-111.99
-112.00
-112.01
-112.03
F
O
2m
19F NMR (565 MHz, CDCl3)
10 0 -10 -20 -30 -40 -50 -60 -70 -80 -90 -100 -110 -120 -130 -140 -150 -160 -170 -180 -190 -200 -210
f1 (ppm)

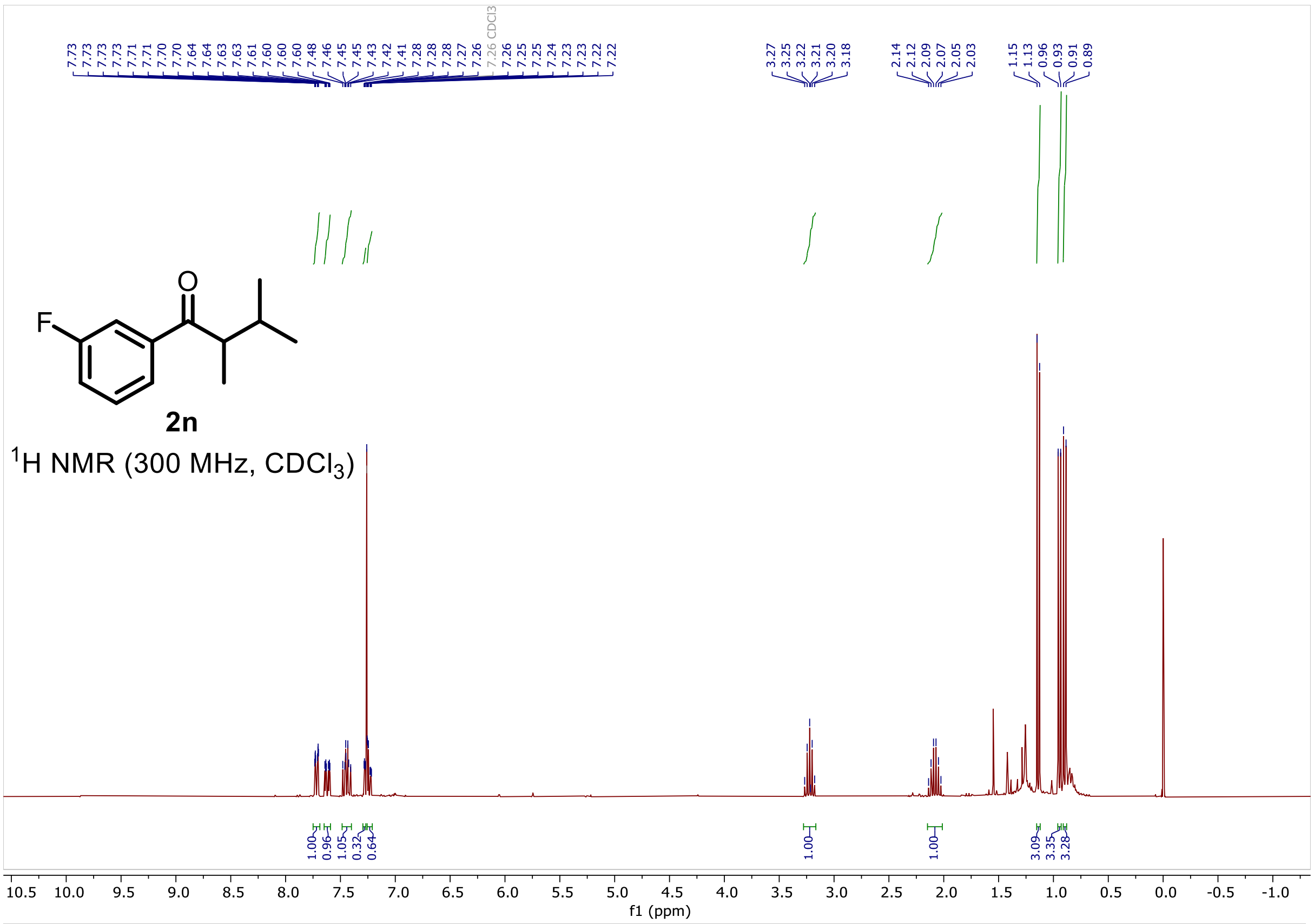
7.73
7.73
7.73
7.71
7.71
7.70
7.64
7.64
7.63
7.63
7.61
7.60
7.60
7.48
7.46
7.45
7.45
7.43
7.42
7.41
7.28
7.28
7.28
7.27
7.26
7.26 CDCl3
7.26
7.25
7.25
7.24
7.23
7.23
7.22
7.22
3.27
3.25
3.22
3.21
3.20
3.18
2.14
2.12
2.09
2.07
2.05
2.03
1.15
1.13
0.96
0.93
0.91
0.89
F
O
2n
^{1}H NMR (300 MHz, CDCl$_3$)
1.00
0.96
1.05
0.32
0.64
1.00
1.00
3.09
3.35
3.28
10.5 10.0 9.5 9.0 8.5 8.0 7.5 7.0 6.5 6.0 5.5 5.0 4.5 4.0 3.5 3.0 2.5 2.0 1.5 1.0 0.5 0.0 -0.5 -1.0
f1 (ppm)

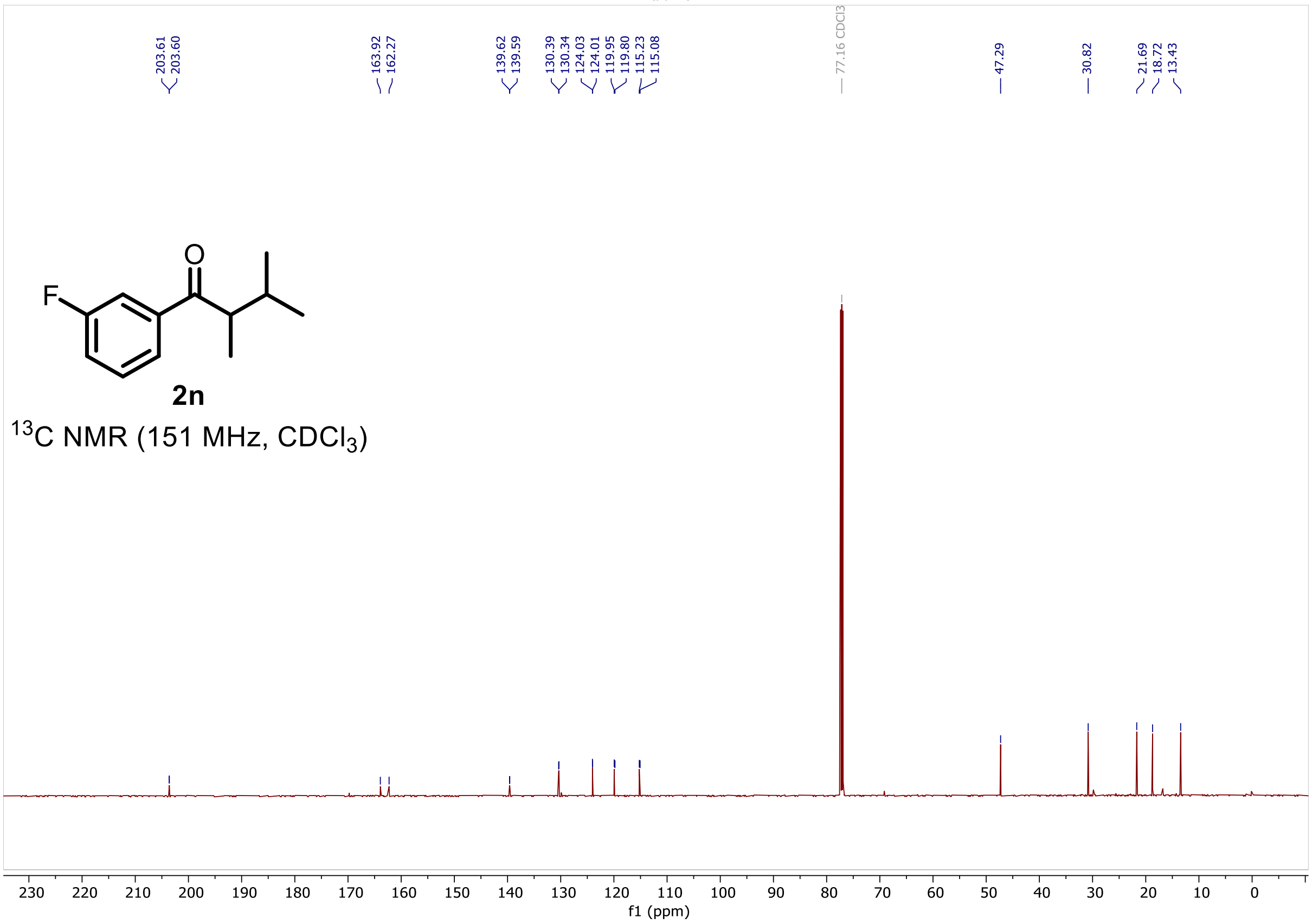
203.61
203.60
163.92
162.27
139.62
139.59
130.39
130.34
124.03
124.01
119.95
119.80
115.23
115.08
77.16 CDCl3
47.29
30.82
21.69
18.72
13.43
F
O
2n
^{13}C NMR (151 MHz, CDCl$_3$)
230 220 210 200 190 180 170 160 150 140 130 120 110 100 90 80 70 60 50 40 30 20 10 0
f1 (ppm)

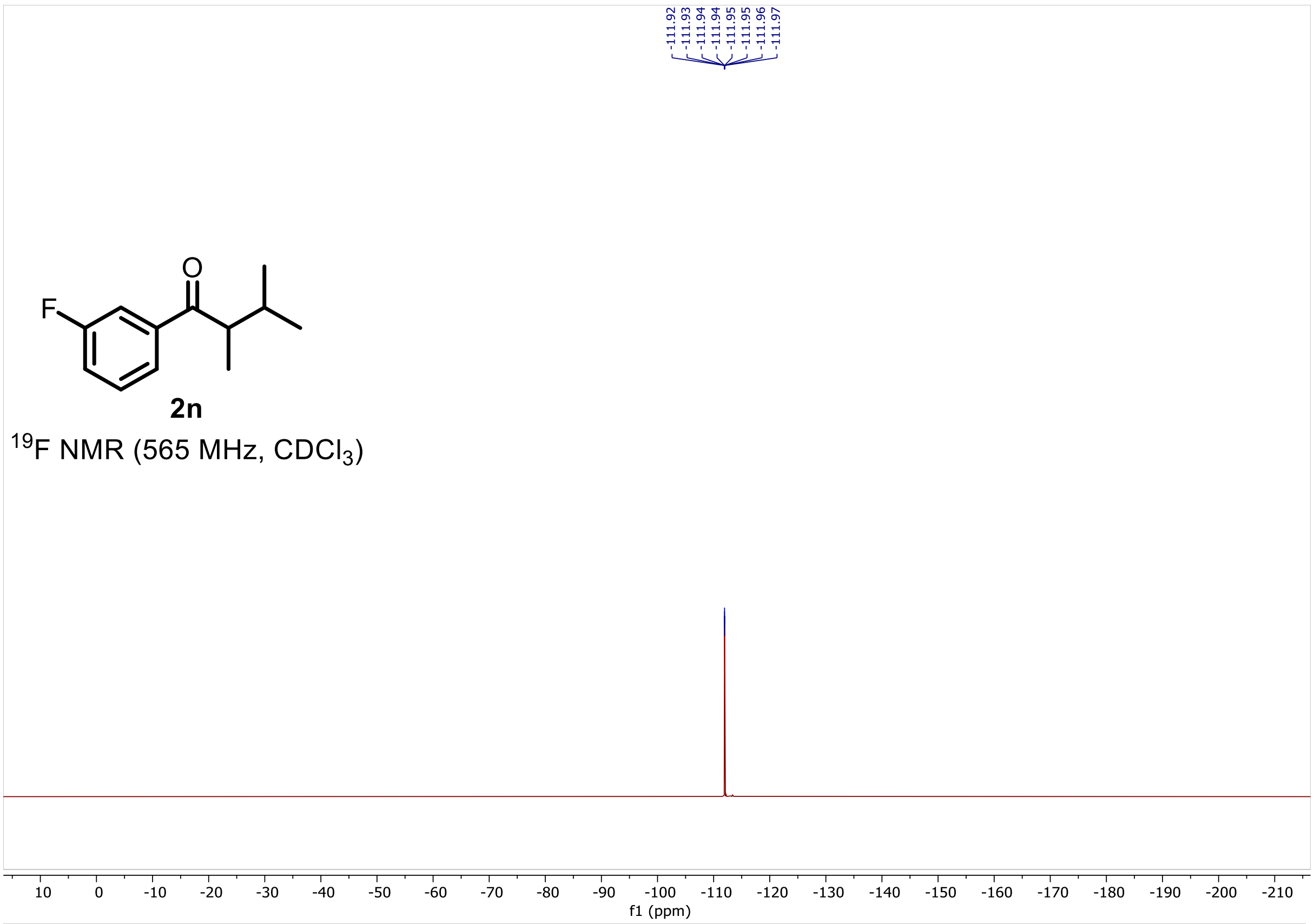

2n

^{19}F NMR (565 MHz, $CDCl_3$)

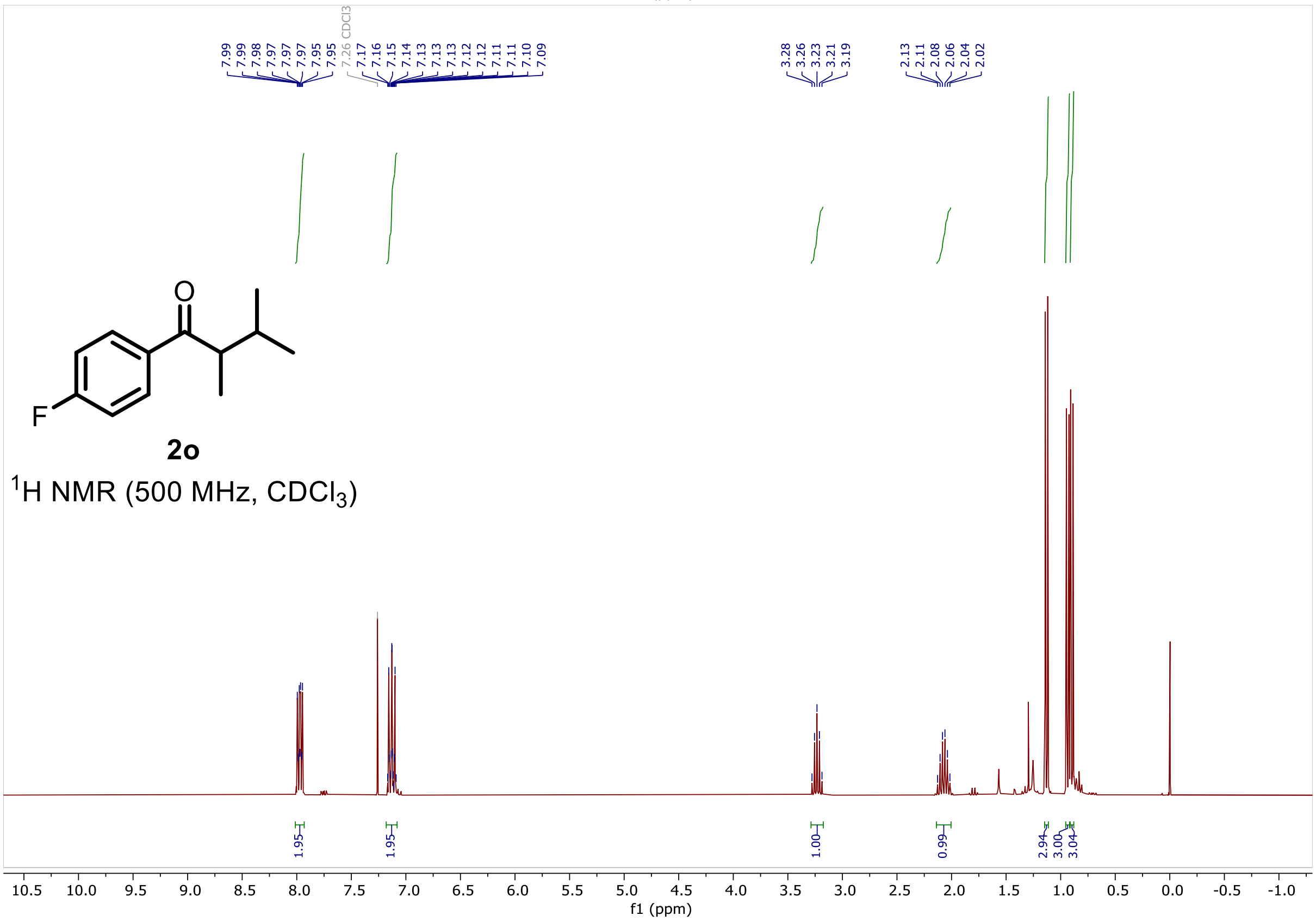

2o

^{1}H NMR (500 MHz, $CDCl_3$)

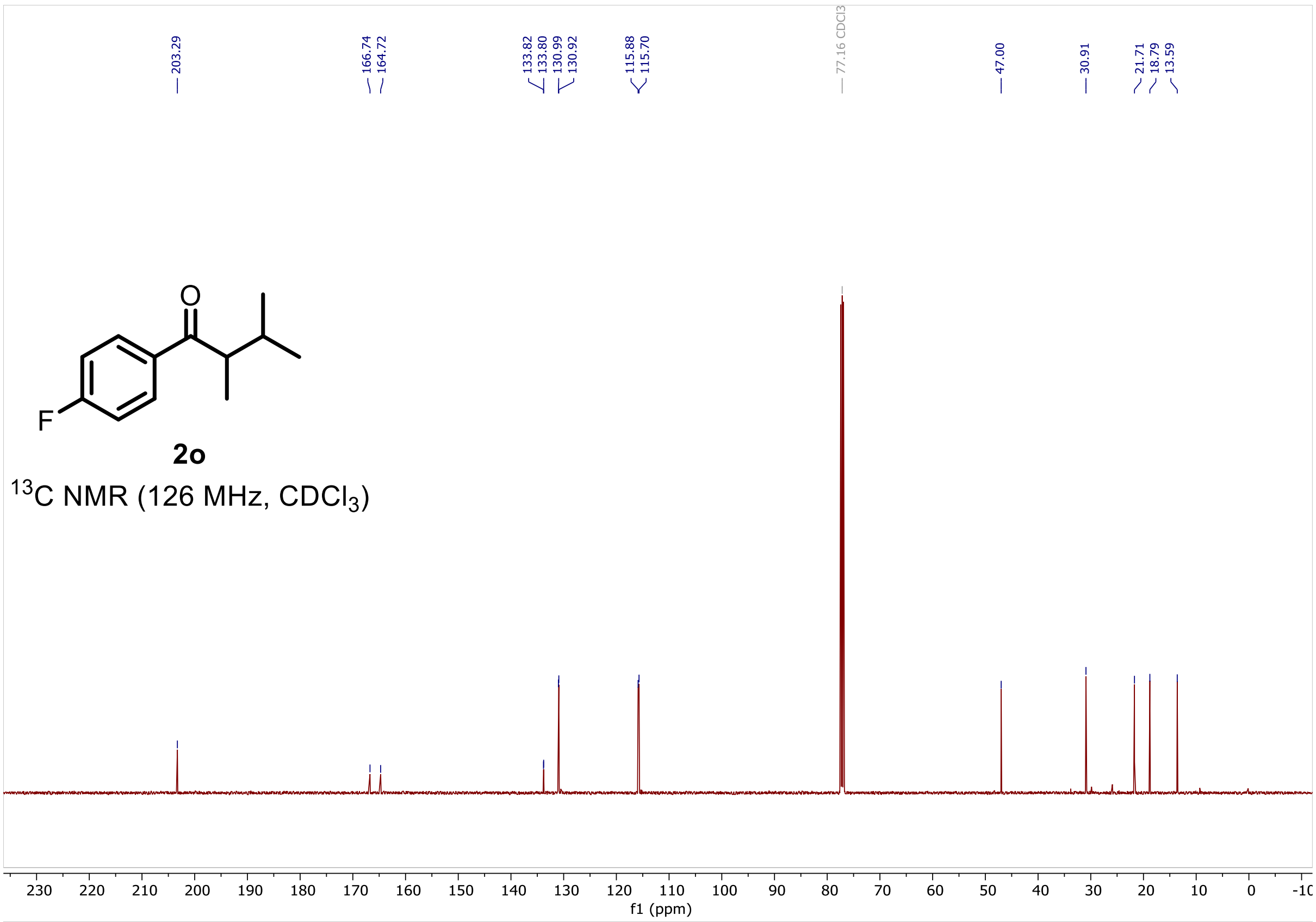
203.29
166.74
164.72
133.82
133.80
130.99
130.92
115.88
115.70
77.16 CDCl3
47.00
30.91
21.71
18.79
13.59
O
F
2o
13C NMR (126 MHz, CDCl3)
f1 (ppm)

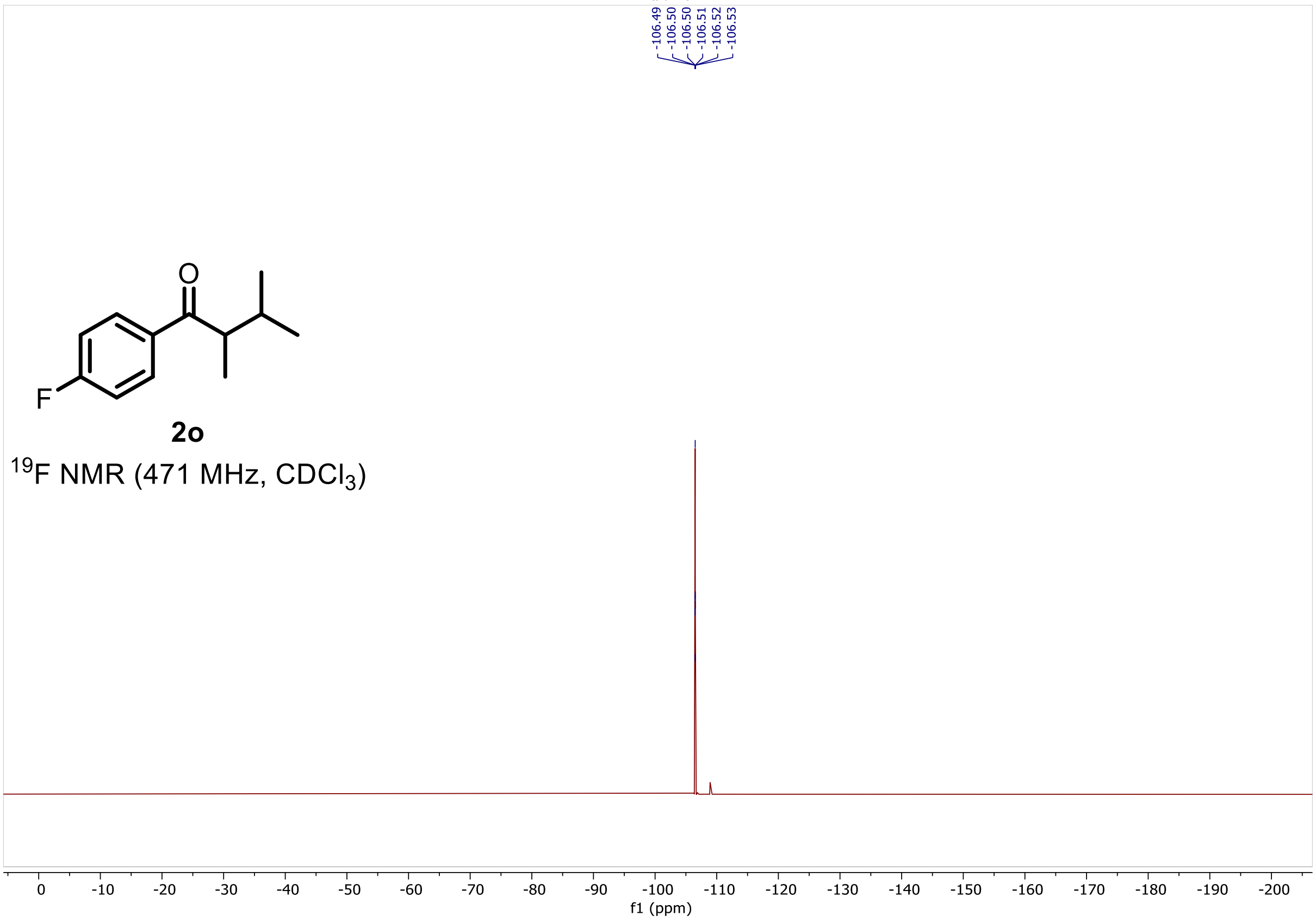
-106.49
-106.50
-106.50
-106.51
-106.52
-106.53
O
F
2o
19F NMR (471 MHz, CDCl3)
f1 (ppm)

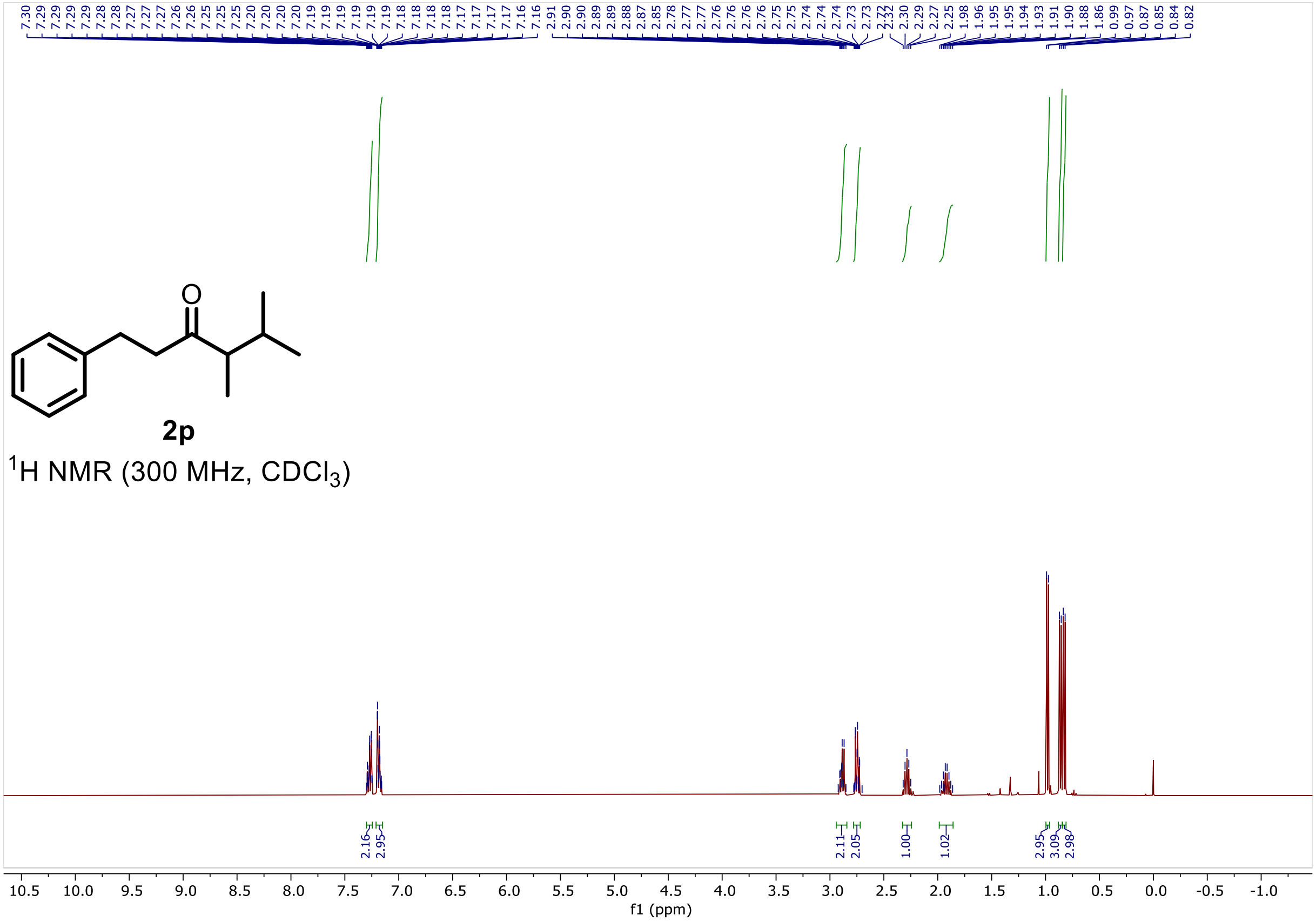

2p

^{1}H NMR (300 MHz, $CDCl_3$)

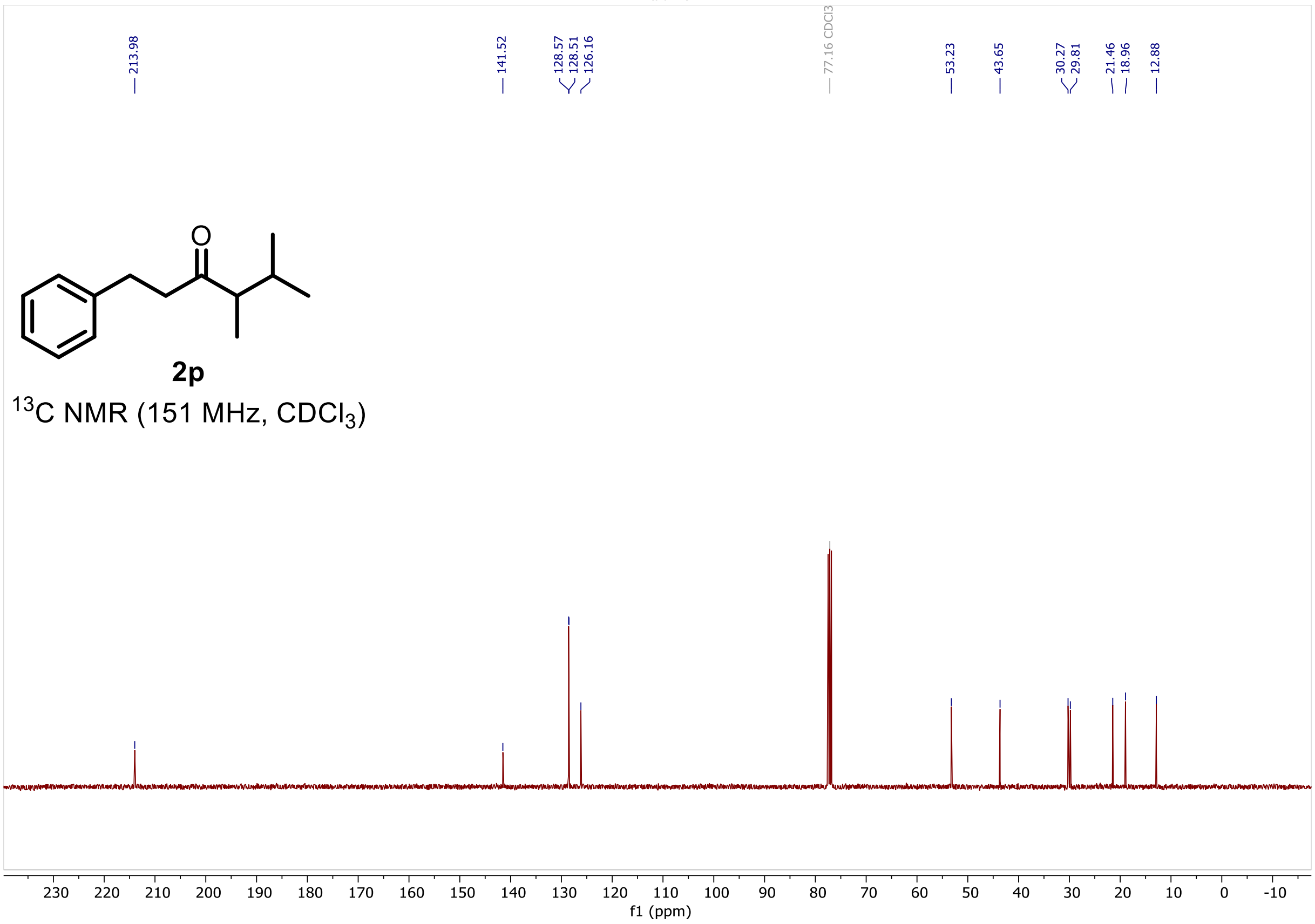

2p

^{13}C NMR (151 MHz, $CDCl_3$)

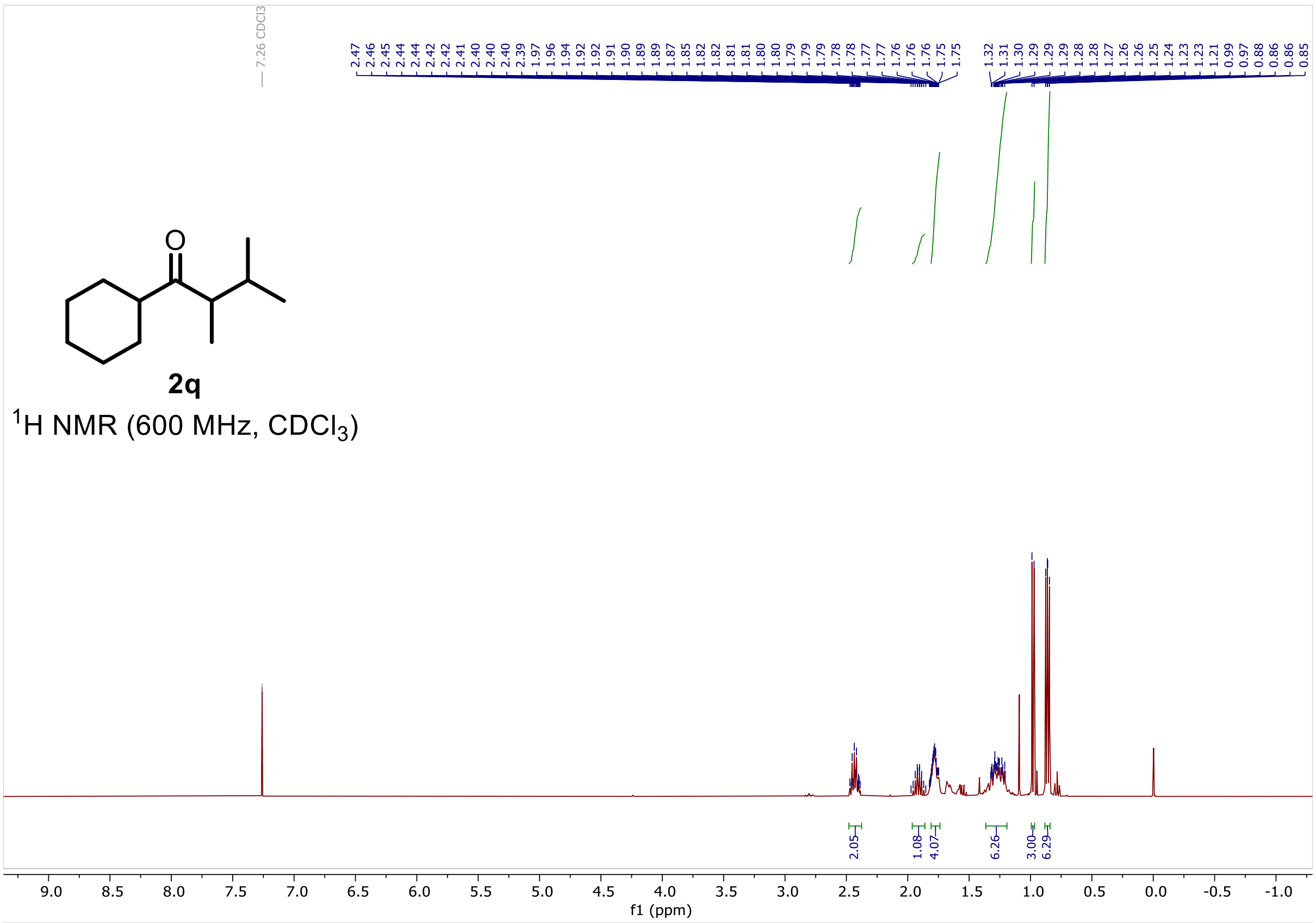
2q
^{1}H NMR (600 MHz, CDCl$_3$)
f1 (ppm)

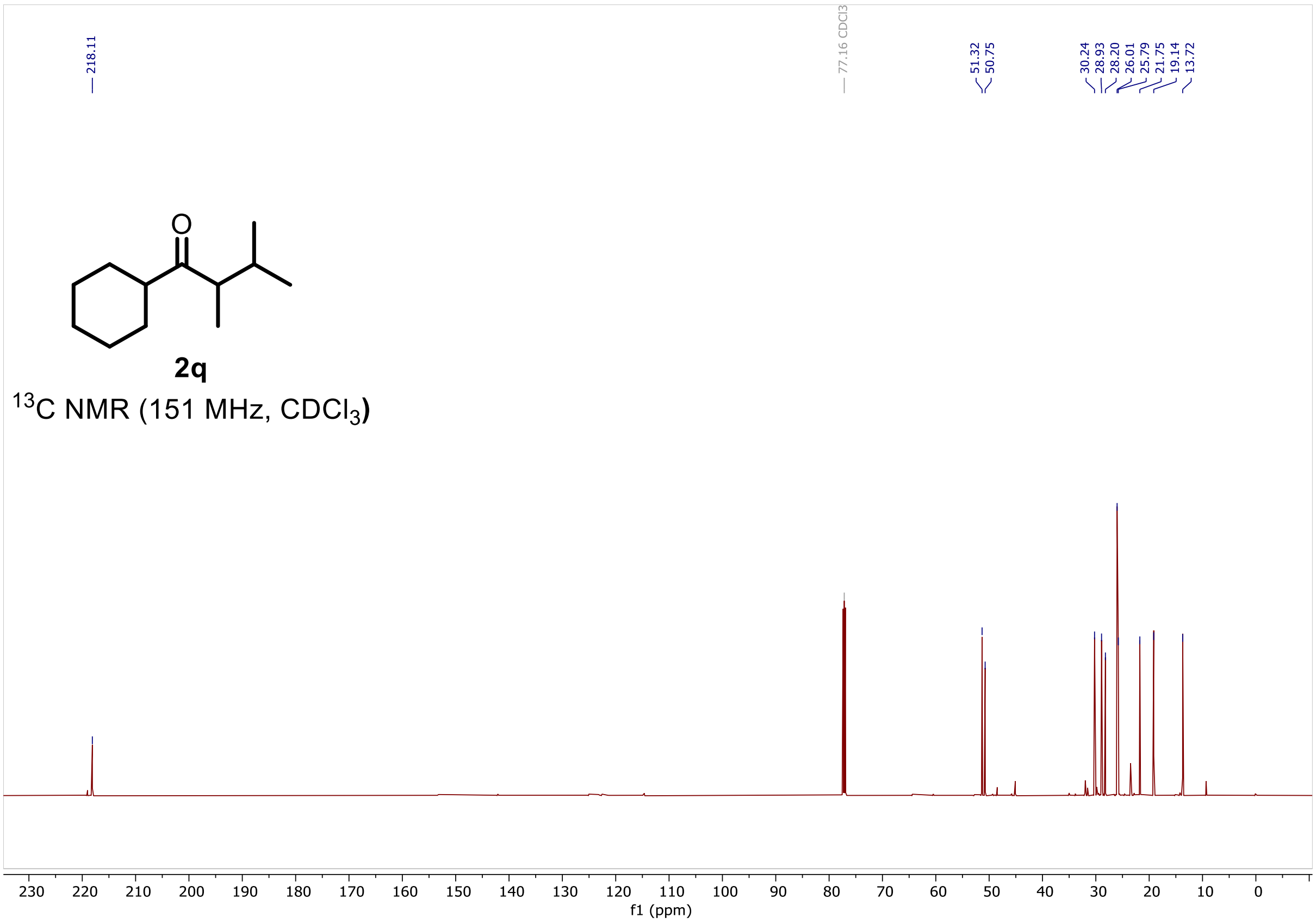
2q
^{13}C NMR (151 MHz, CDCl$_3$)
f1 (ppm)

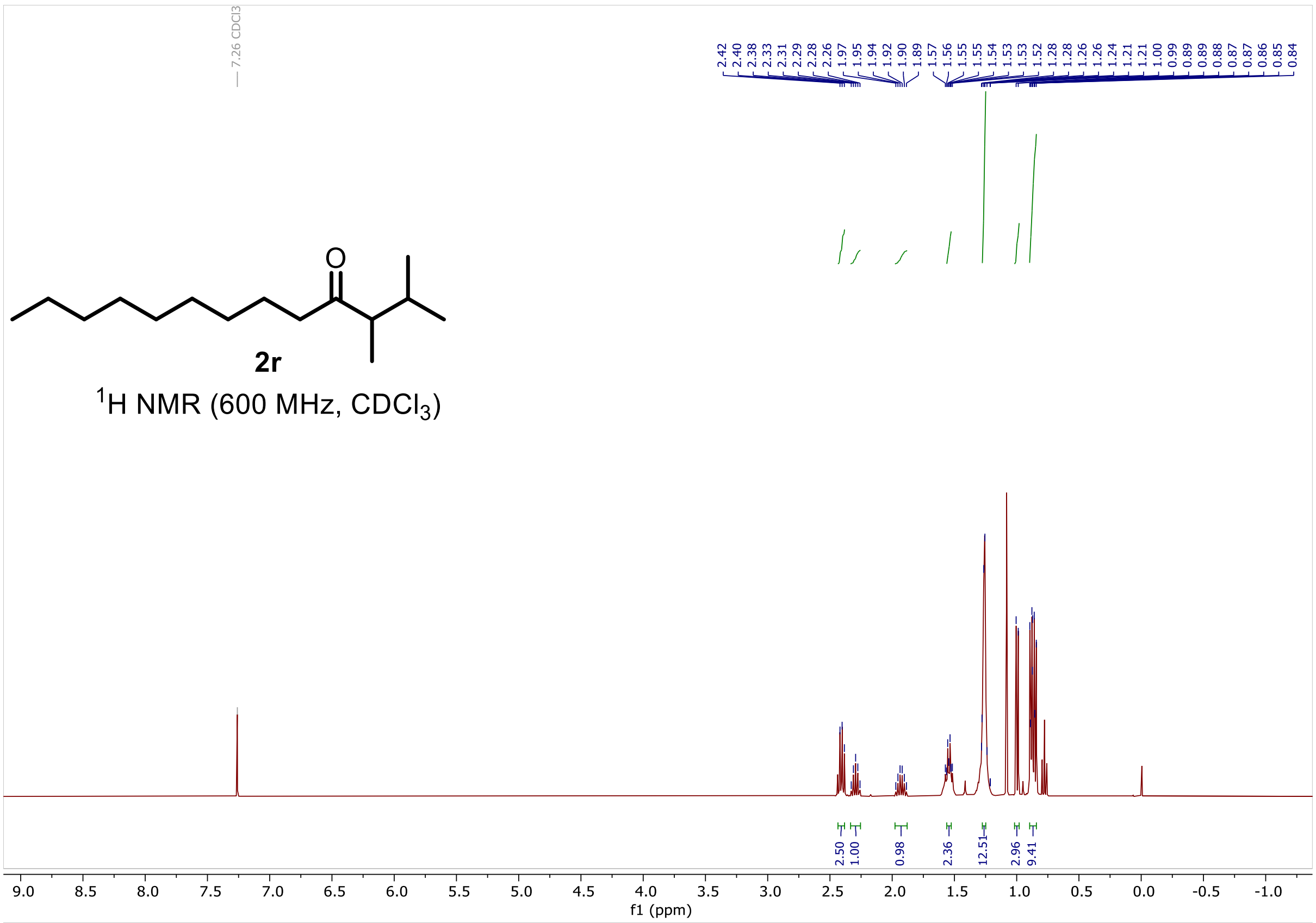
2r
^{1}H NMR (600 MHz, $CDCl_3$)
7.26 CDCl3
f1 (ppm)

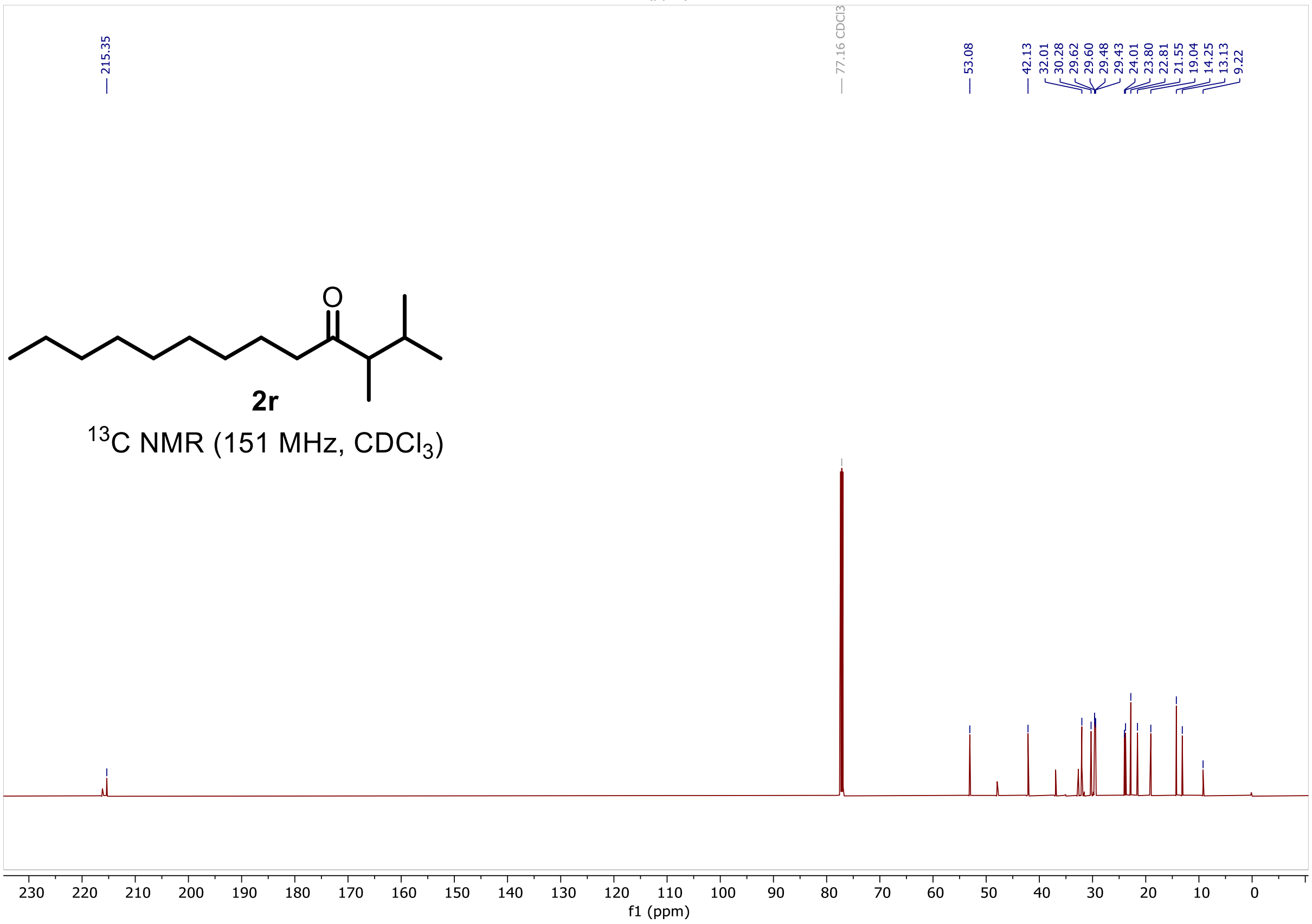
2r
^{13}C NMR (151 MHz, $CDCl_3$)
215.35
77.16 CDCl3
53.08
42.13
32.01
30.28
29.62
29.60
29.48
29.43
24.01
23.80
22.81
21.55
19.04
14.25
13.13
9.22
f1 (ppm)

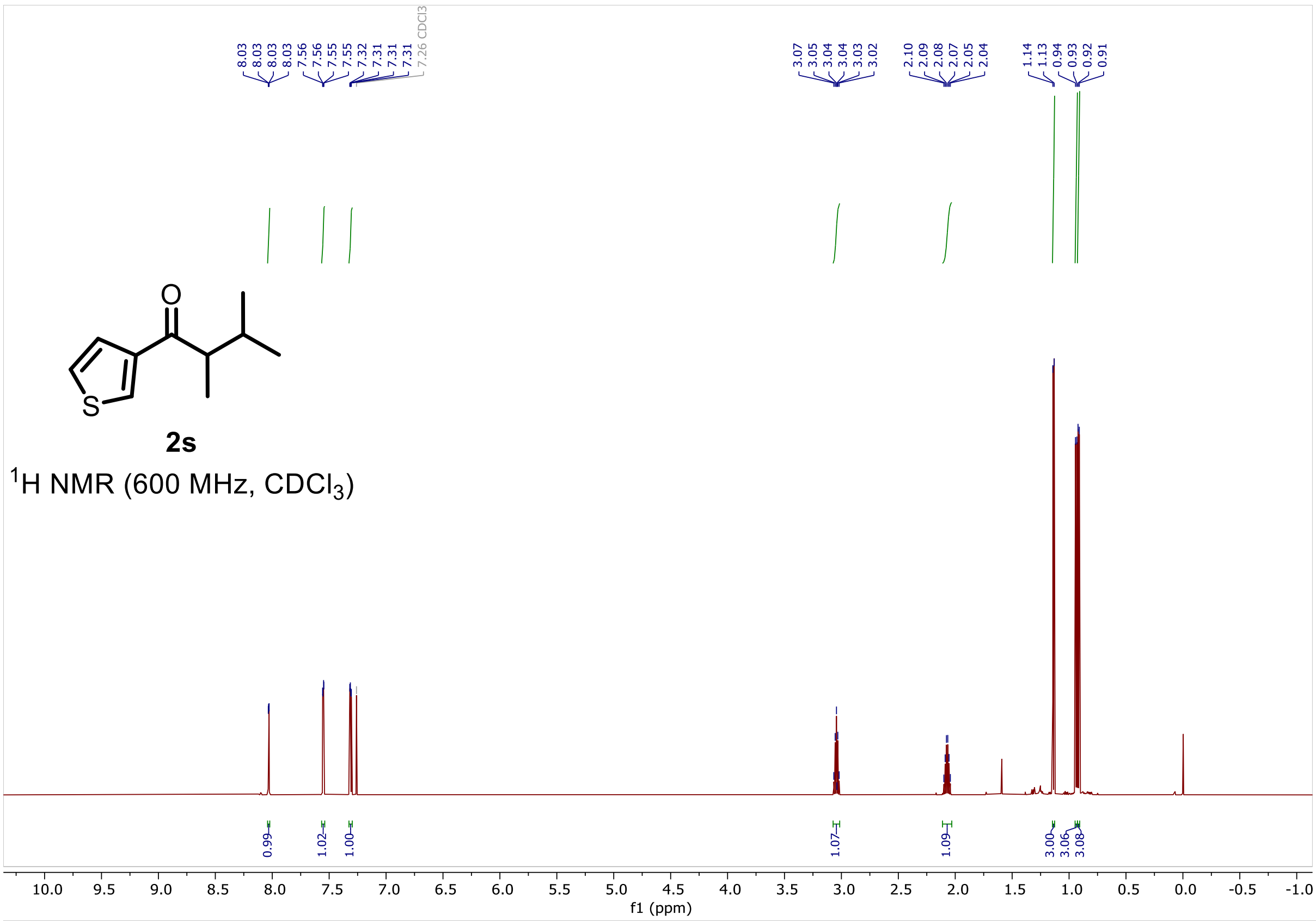

2s

^{1}H NMR (600 MHz, $CDCl_3$)

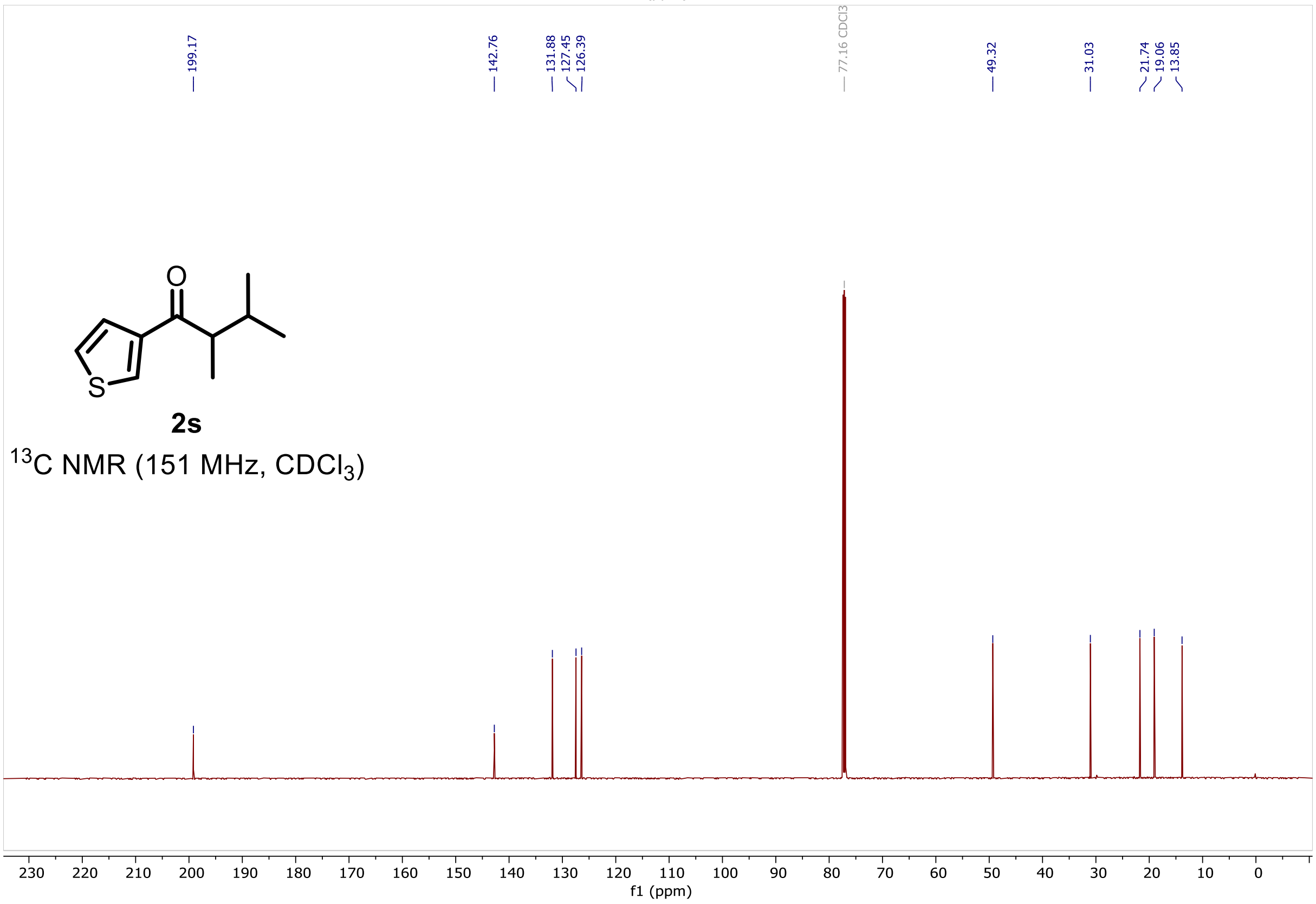

2s

^{13}C NMR (151 MHz, $CDCl_3$)

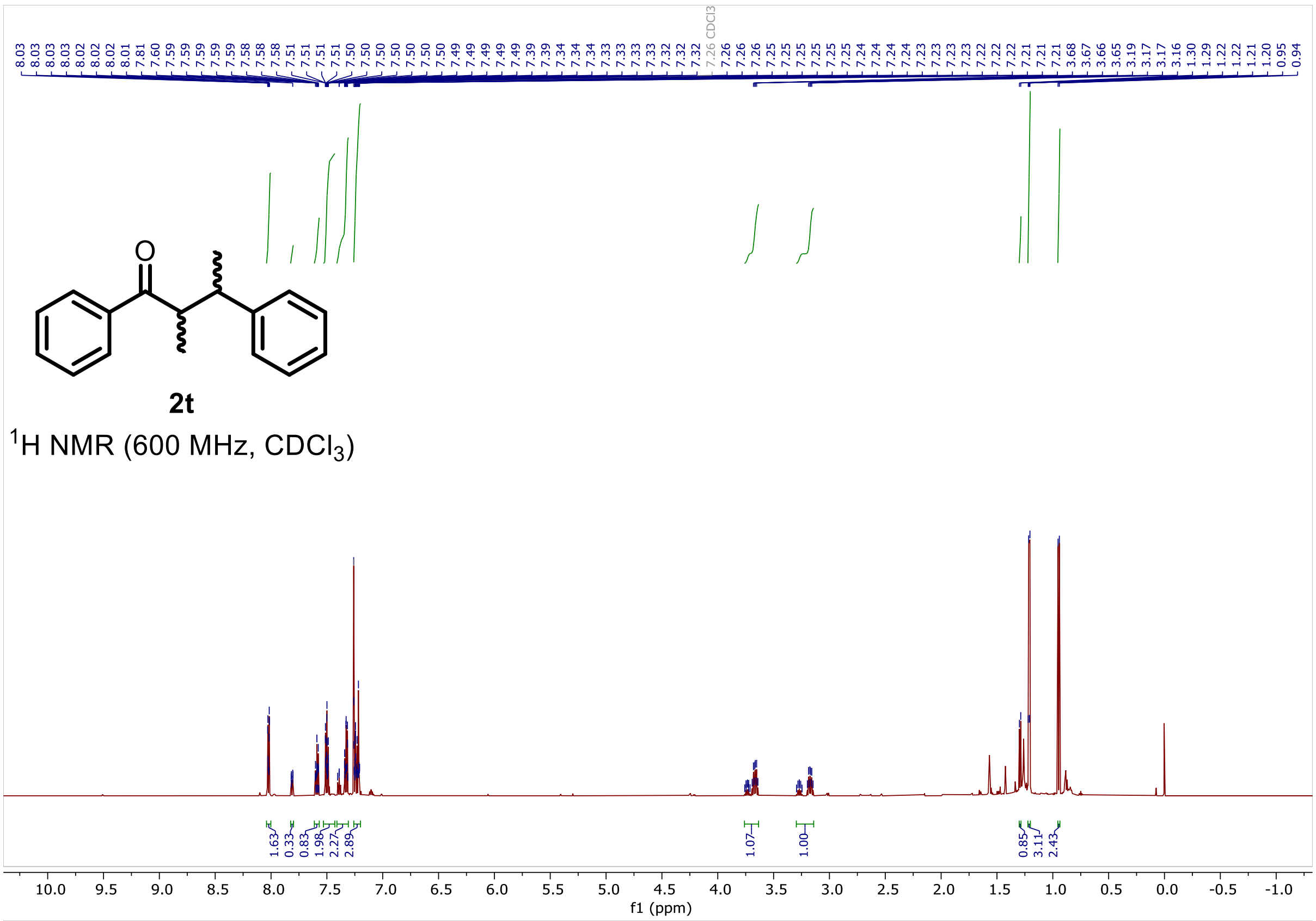

2t

^{1}H NMR (600 MHz, $CDCl_3$)

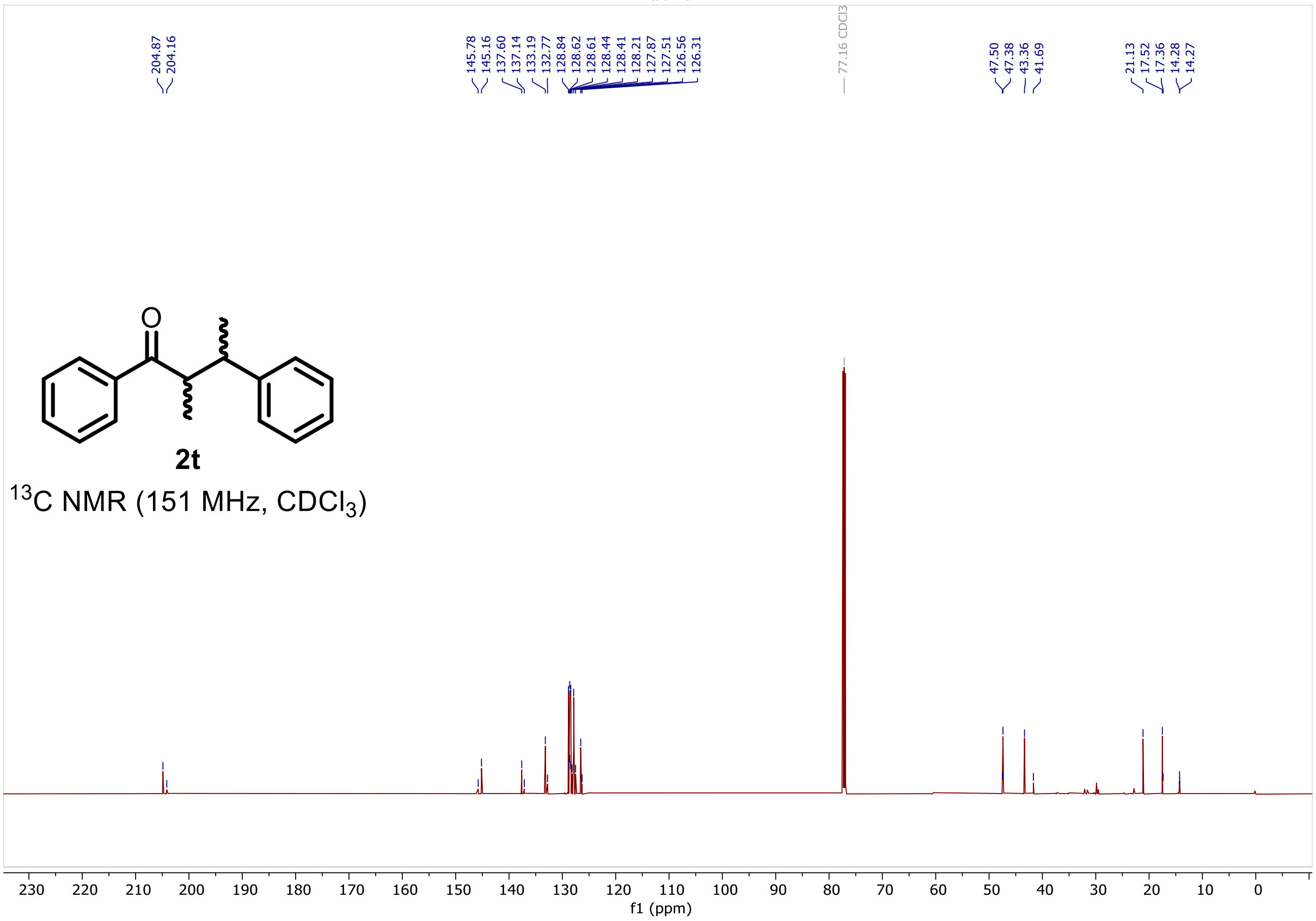

2t

^{13}C NMR (151 MHz, $CDCl_3$)

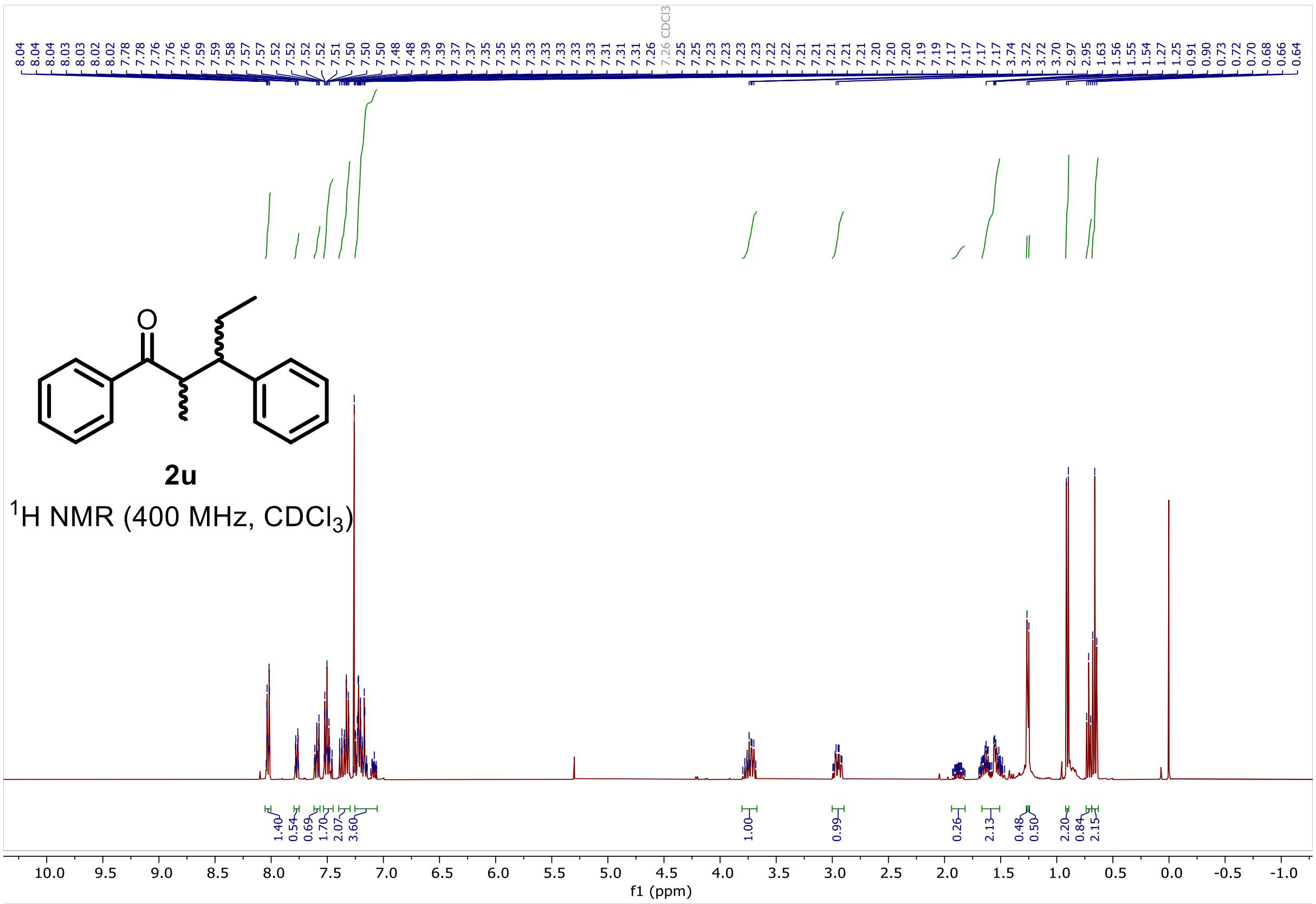
8.04 8.04 8.04 8.03 8.03 8.02 8.02 7.78 7.78 7.76 7.76 7.76 7.59 7.59 7.58 7.57 7.57 7.52 7.52 7.52 7.52 7.51 7.50 7.50 7.50 7.48 7.48 7.39 7.39 7.37 7.37 7.35 7.35 7.35 7.33 7.33 7.33 7.33 7.33 7.31 7.31 7.26 CDCl3 7.25 7.25 7.23 7.23 7.23 7.22 7.22 7.21 7.21 7.21 7.21 7.20 7.20 7.19 7.19 7.17 7.17 7.17 7.17 3.74 3.72 3.72 3.70 2.97 2.95 1.63 1.56 1.55 1.54 1.27 1.25 0.91 0.90 0.73 0.72 0.70 0.68 0.66 0.64
O
2u
^{1}H NMR (400 MHz, CDCl$_3$)
1.40 0.54 0.69 1.70 2.07 3.60 1.00 0.99 0.26 2.13 0.48 0.50 2.20 0.84 2.15
10.0 9.5 9.0 8.5 8.0 7.5 7.0 6.5 6.0 5.5 5.0 4.5 4.0 3.5 3.0 2.5 2.0 1.5 1.0 0.5 0.0 -0.5 -1.0
f1 (ppm)

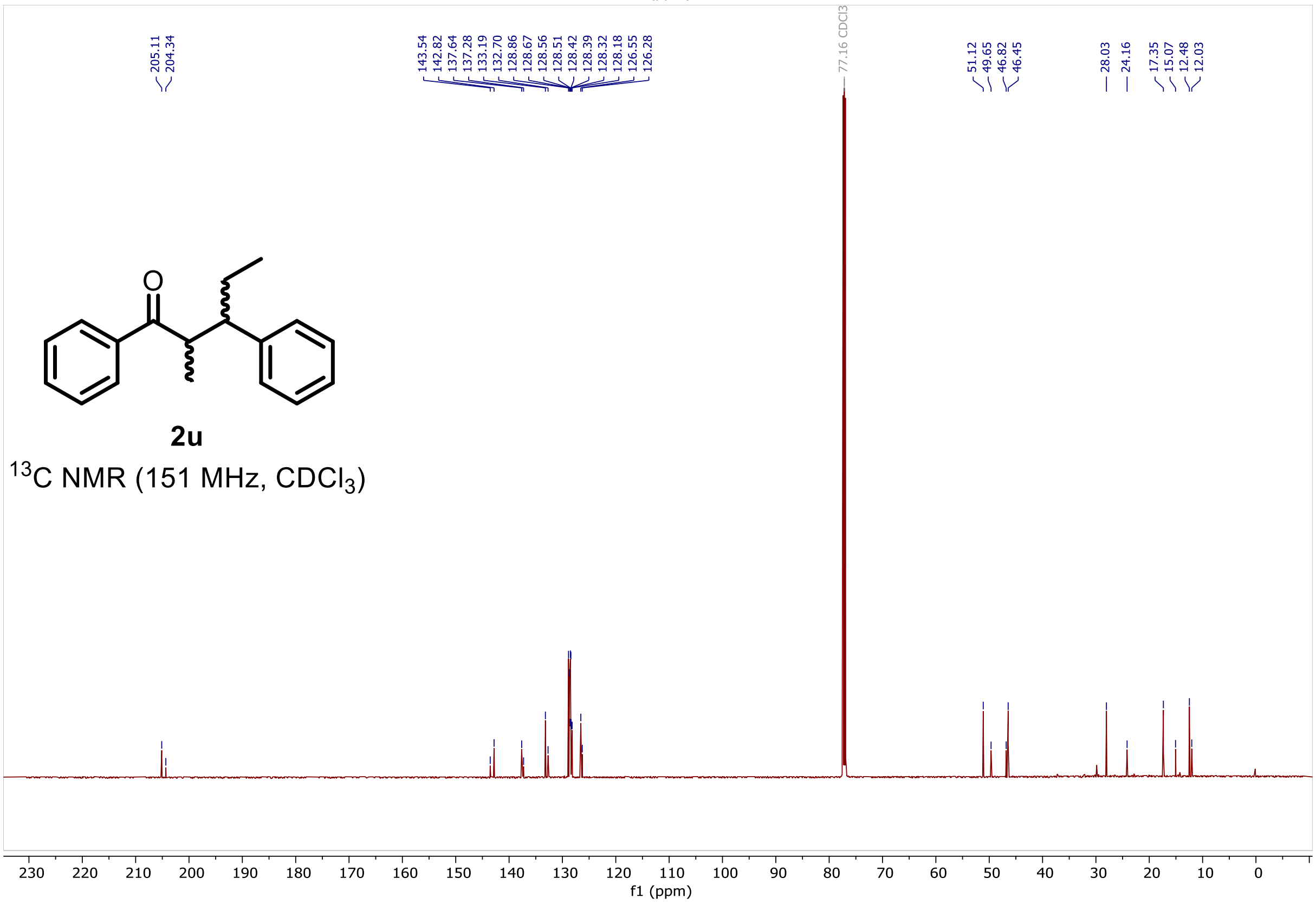
205.11 204.34 143.54 142.82 137.64 137.28 133.19 132.70 128.86 128.67 128.56 128.51 128.42 128.39 128.32 128.18 126.55 126.28 77.16 CDCl3 51.12 49.65 46.82 46.45 28.03 24.16 17.35 15.07 12.48 12.03
O
2u
^{13}C NMR (151 MHz, CDCl$_3$)
230 220 210 200 190 180 170 160 150 140 130 120 110 100 90 80 70 60 50 40 30 20 10 0
f1 (ppm)

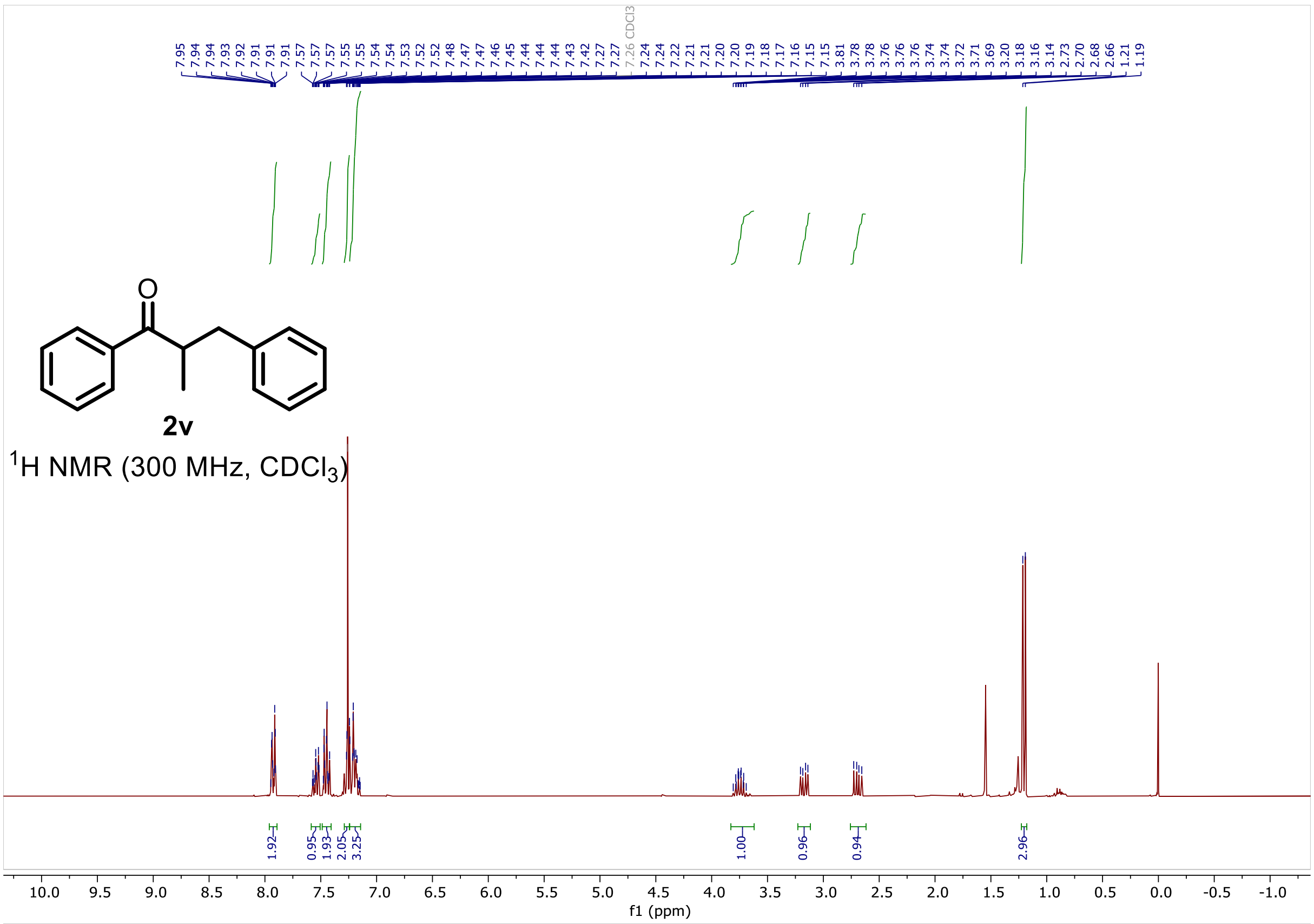

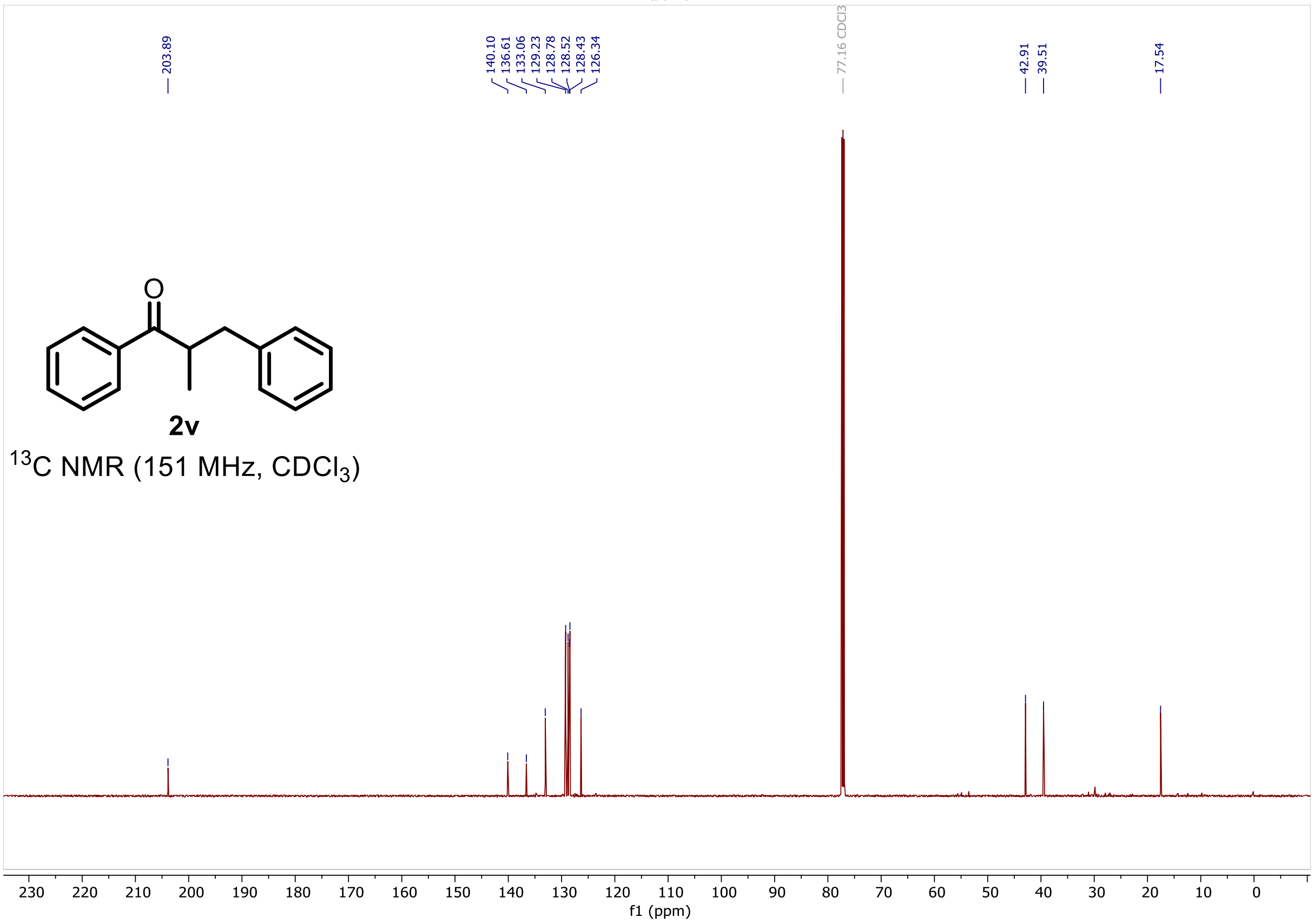

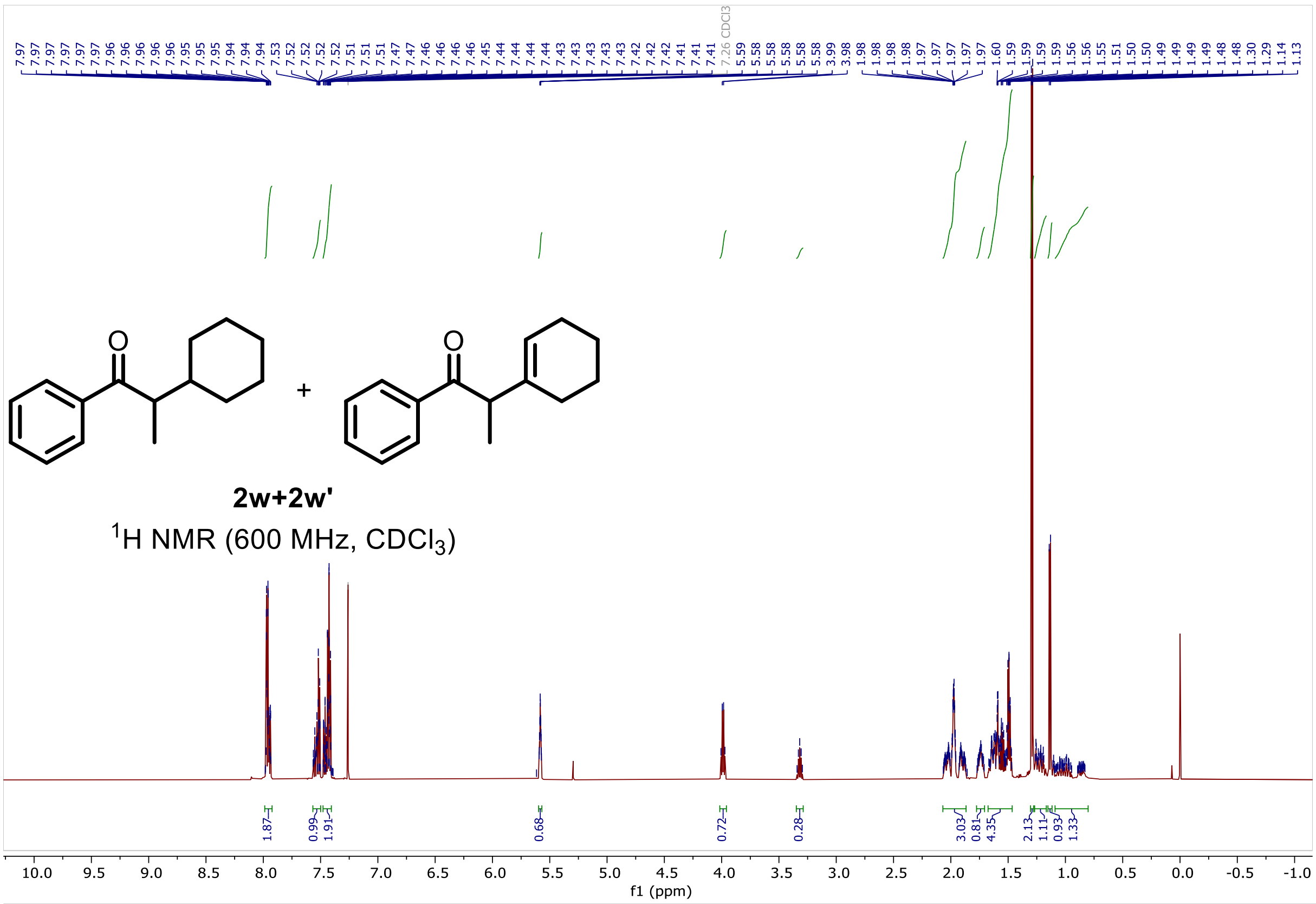
2w+2w'
1H NMR (600 MHz, CDCl3)
f1 (ppm)

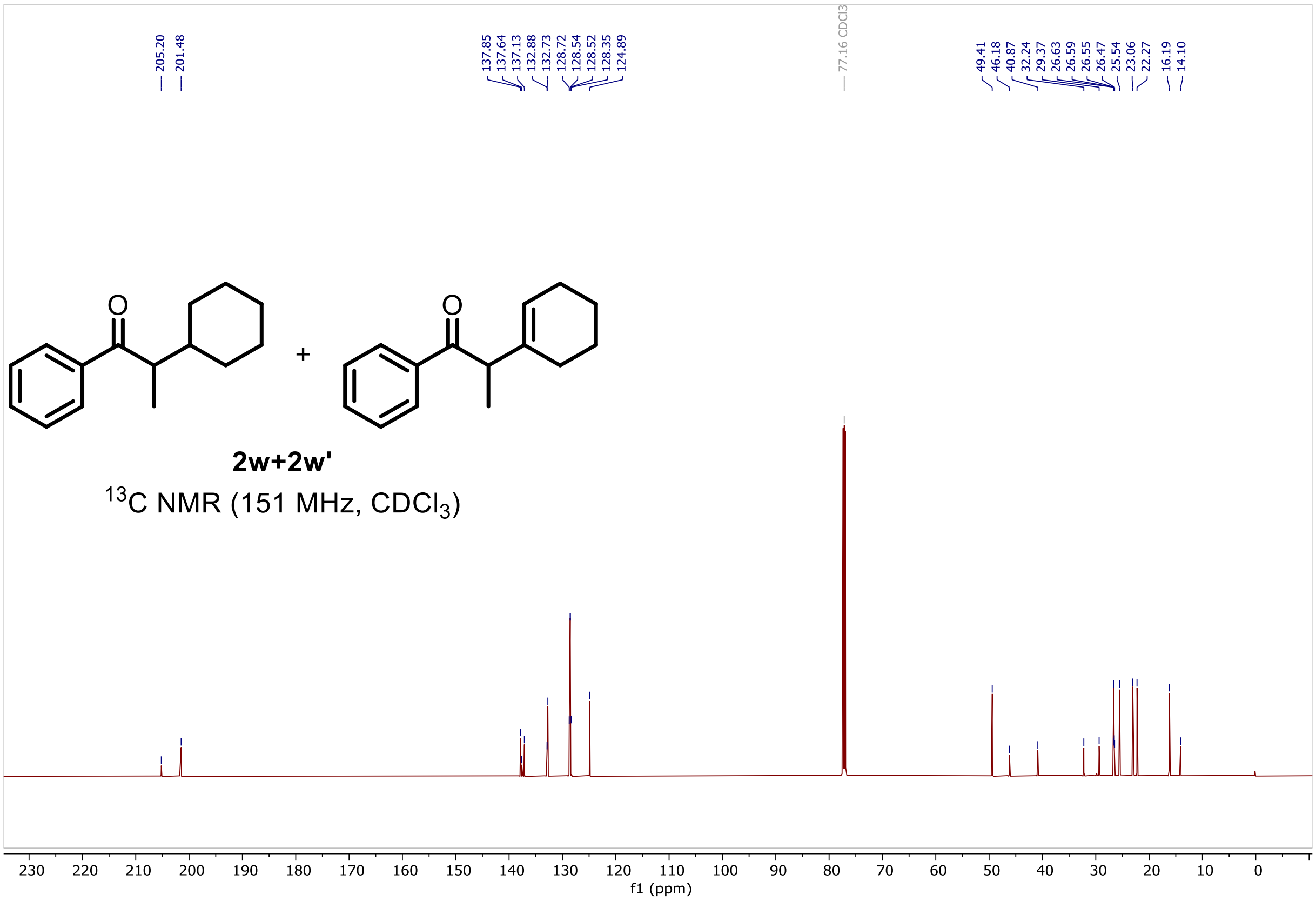
205.20
201.48
137.85
137.64
137.13
132.88
132.73
128.72
128.54
128.52
128.35
124.89
77.16 CDCl3
49.41
46.18
40.87
32.24
29.37
26.63
26.59
26.55
26.47
25.54
23.06
22.27
16.19
14.10
2w+2w'
13C NMR (151 MHz, CDCl3)
f1 (ppm)

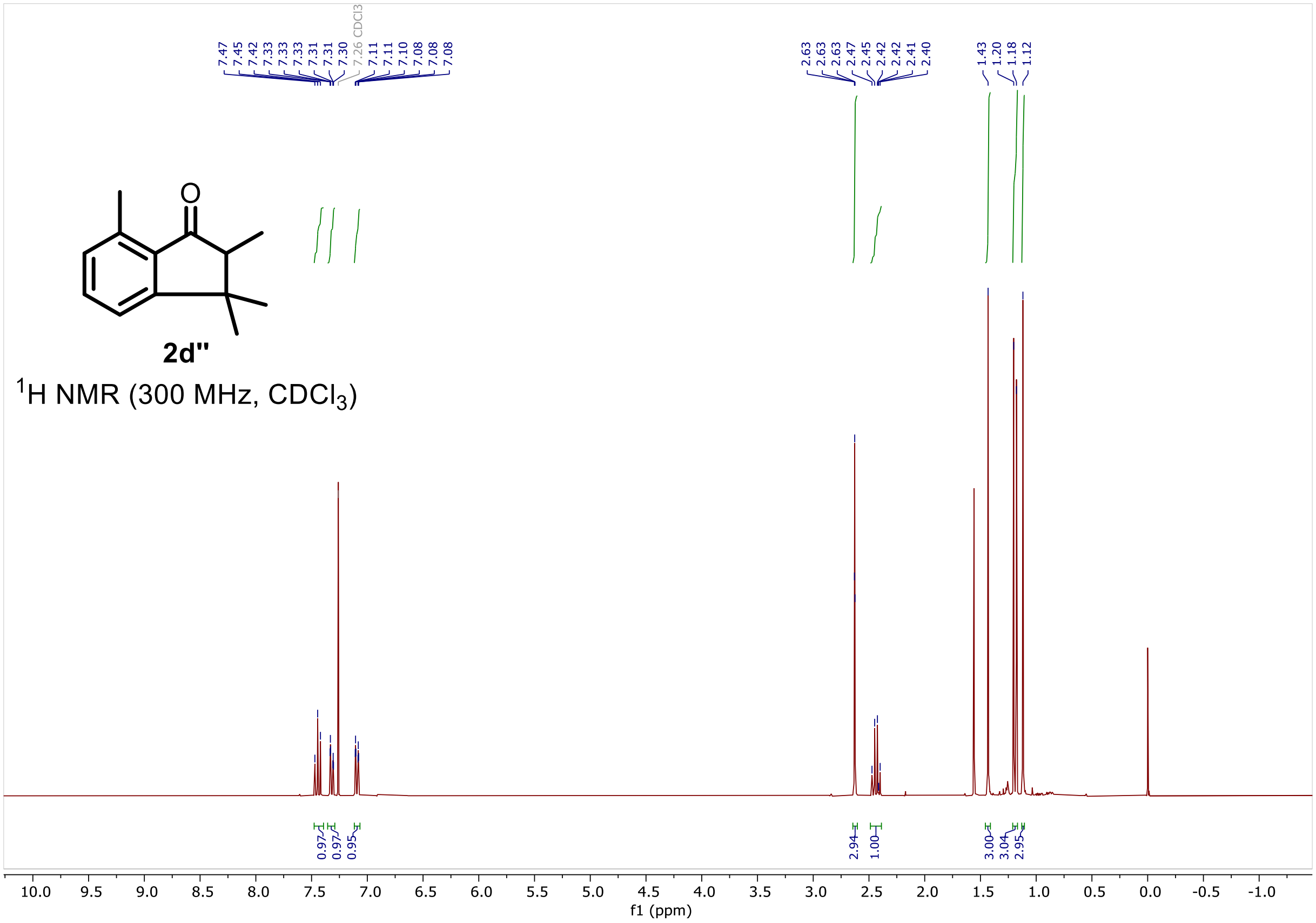
2d''
^{1}H NMR (300 MHz, $CDCl_3$)
7.47 7.45 7.42 7.33 7.33 7.33 7.31 7.31 7.30 7.26 CDCl3 7.11 7.11 7.10 7.08 7.08 7.08
2.63 2.63 2.63 2.47 2.45 2.42 2.42 2.41 2.40
1.43 1.20 1.18 1.12
0.97 0.97 0.95 2.94 1.00 3.00 3.04 2.95
f1 (ppm)

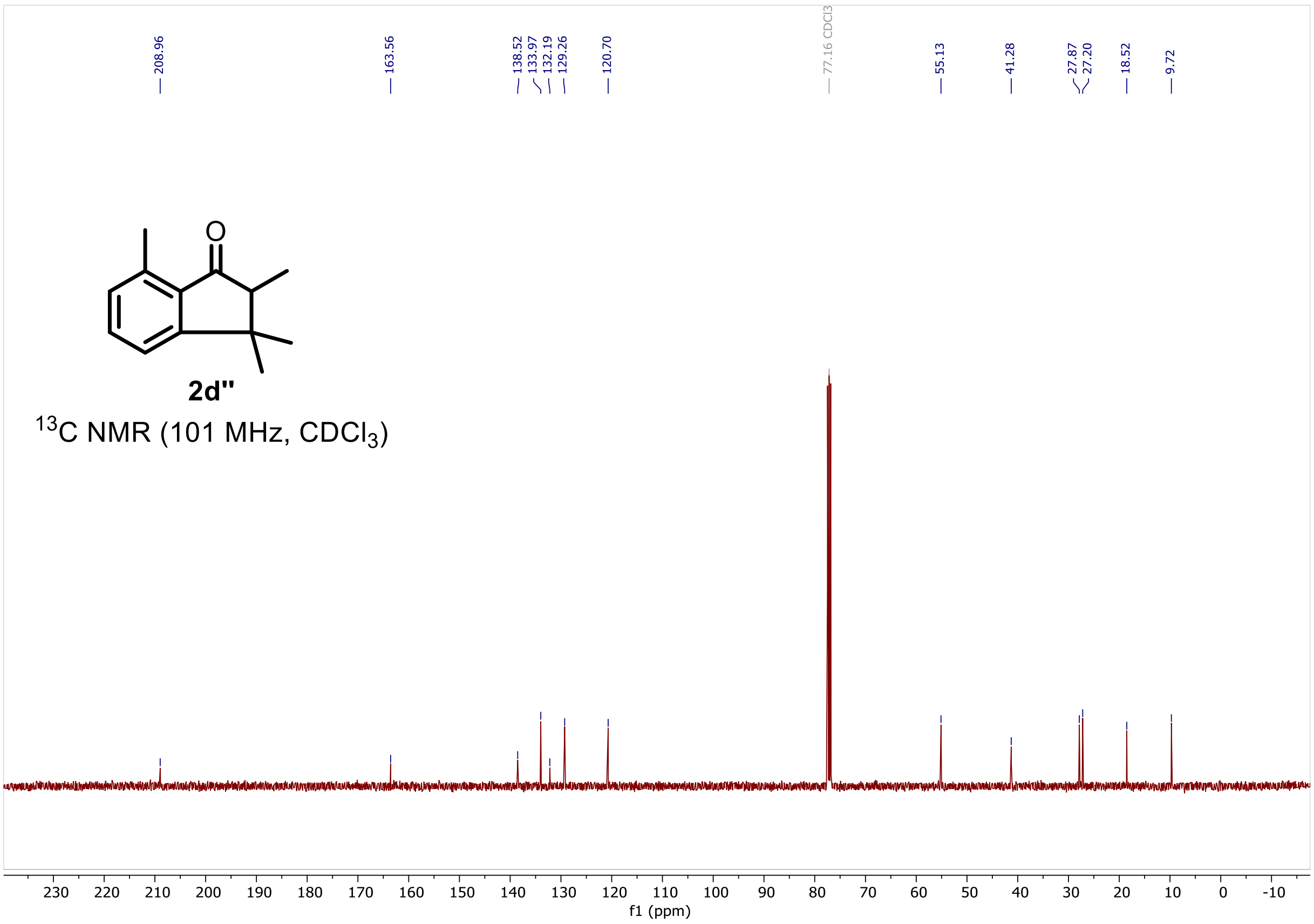
2d''
^{13}C NMR (101 MHz, $CDCl_3$)
208.96 163.56 138.52 133.97 132.19 129.26 120.70 77.16 CDCl3 55.13 41.28 27.87 27.20 18.52 9.72
f1 (ppm)

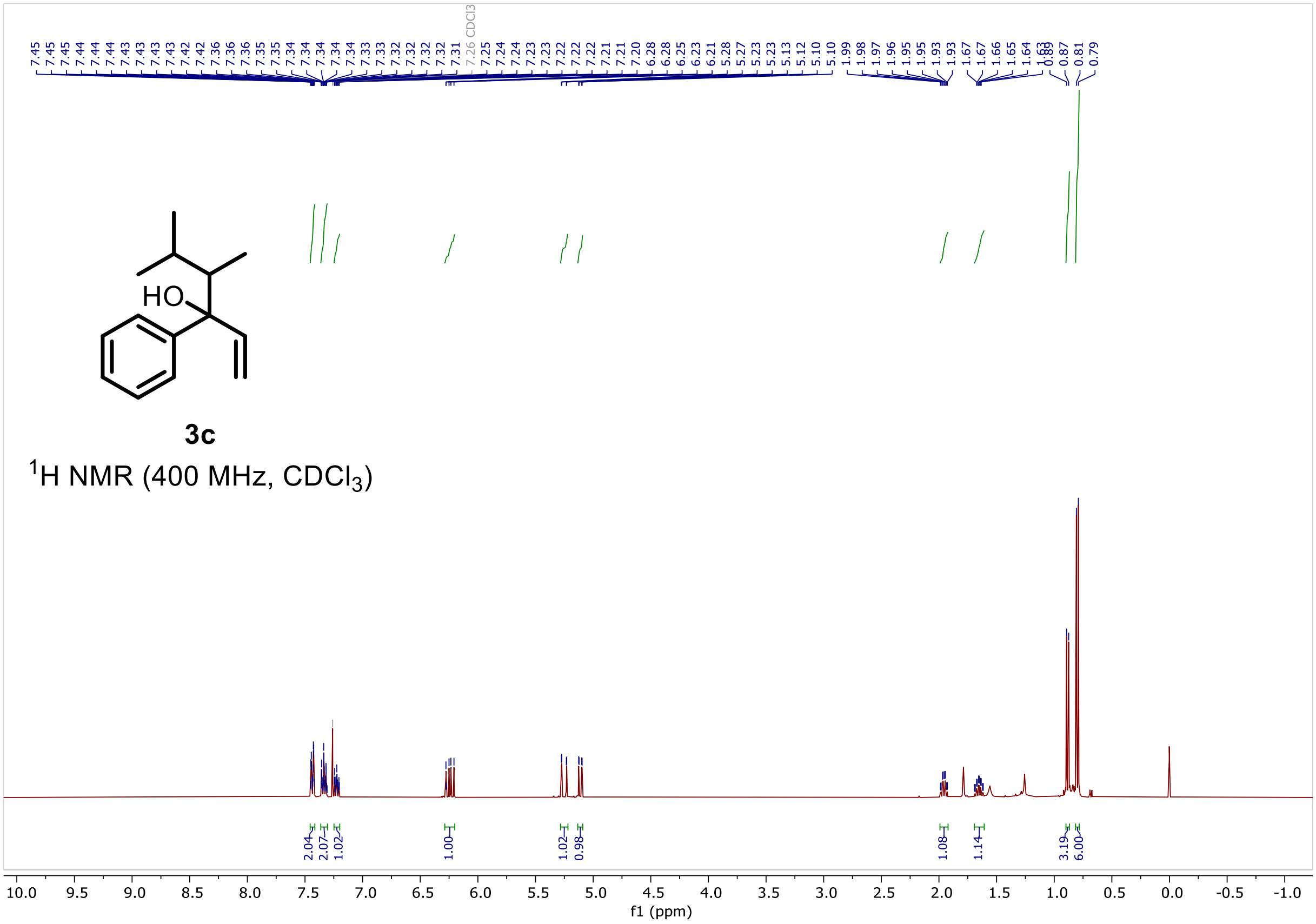
HO
3c
1H NMR (400 MHz, CDCl3)
f1 (ppm)

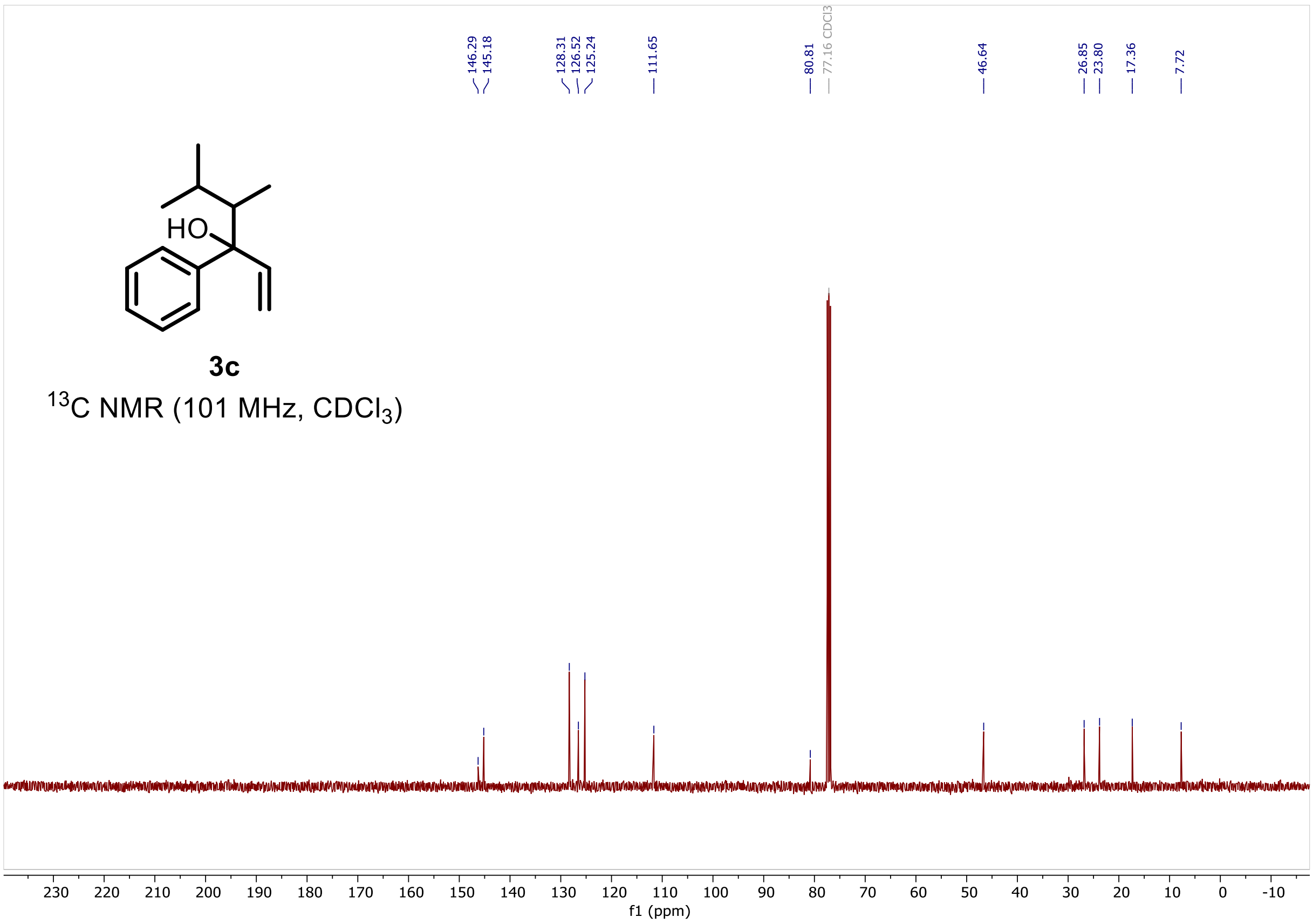
HO
3c
13C NMR (101 MHz, CDCl3)
146.29
145.18
128.31
126.52
125.24
111.65
80.81
77.16 CDCl3
46.64
26.85
23.80
17.36
7.72
f1 (ppm)

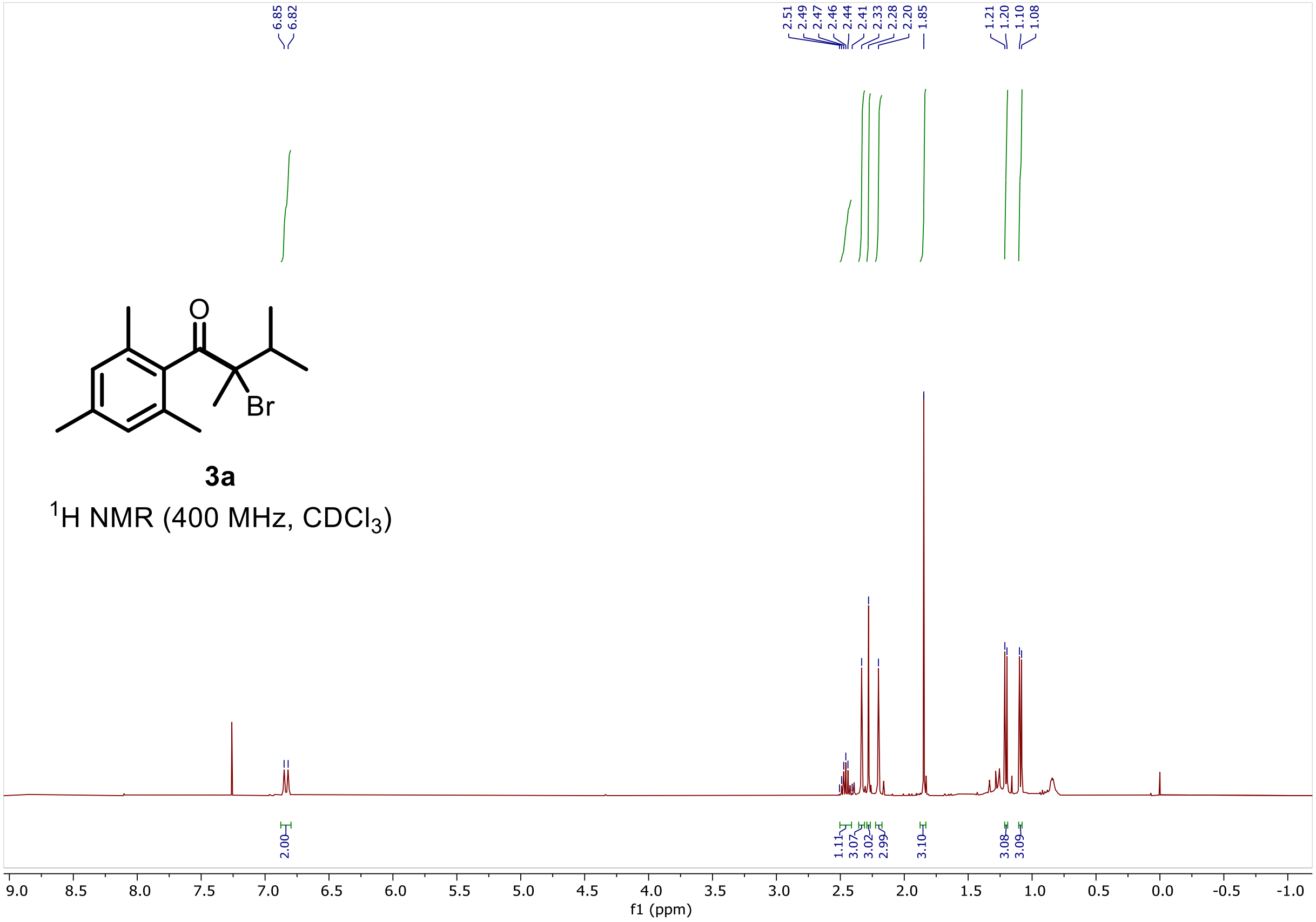
3a
¹H NMR (400 MHz, CDCl3)
f1 (ppm)

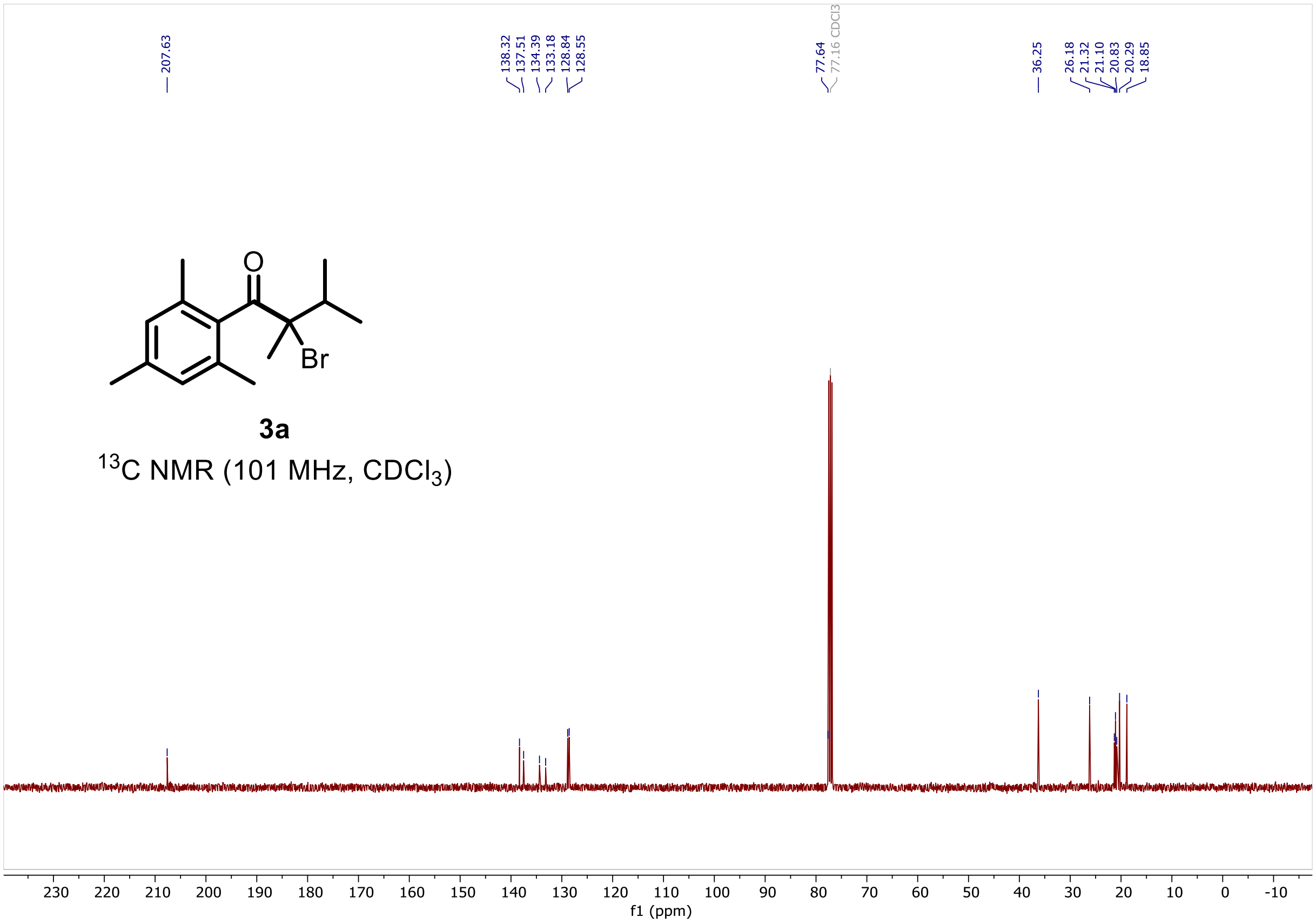
3a
¹³C NMR (101 MHz, CDCl3)
f1 (ppm)
